# Chemoselective Difluoromethylation of Nucleosides

**DOI:** 10.1021/acs.orglett.5c02204

**Published:** 2025-06-17

**Authors:** Otto Linden, Alexander Axer, Andrea Taladriz-Sender, Glenn A. Burley

**Affiliations:** † Department of Pure and Applied Chemistry, 3527 University of Strathclyde, Thomas Graham Building, 295 Cathedral Street, Glasgow G1 1XL, U.K.; ‡ Strathclyde Centre for Molecular Bioscience, University of Strathclyde, Glasgow G1 1XQ, U.K.

## Abstract

A profiling platform to define the chemoselectivity of the carbene-mediated difluoromethylation of nucleosides is described. First, the optimized reaction conditions for the difluoromethylation of silyl-protected nucleosides are established using TMS-CF_2_Br and KOAc to form the difluoromethyl carbene *in situ*. Second, the scope of these reaction conditions to difluoromethylate uridine- and cytidine-based 2′-deoxyribonucleoside and ribonucleoside analogues is established. When uridine analogues are substrates, O-difluoromethylation at the 4-position is observed, whereas O-difluoromethylation at the 2-position of cytidine analogues predominates. S-Difluoromethylation is preferable over O-difluoromethylation when thionucleosides are used. In all cases, no N-difluoromethylation is observed. Finally, silyl deprotection afforded difluoromethylated free nucleosides, thereby enabling the exploration of their utility for broader applications in medicinal chemistry and chemical biology.

## Graphical abstract

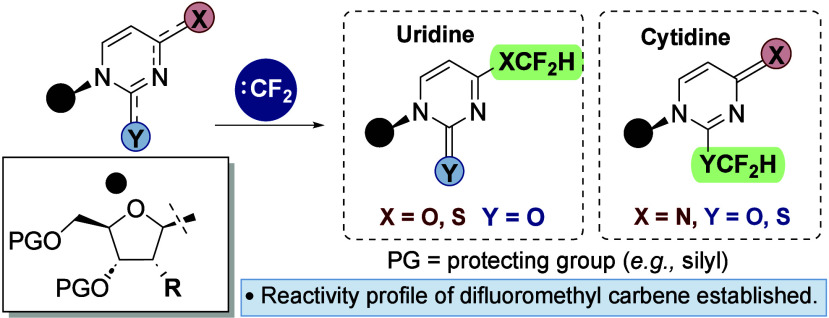

Nucleosides are essential molecules that are used in all living cells and viruses. Their ubiquity is demonstrated by the role of nucleosides forming the basic building blocks of DNA and RNA, as well as their utility as small molecule second messengers and cofactors (e.g., *S*-adenosyl methionine (SAM)).^1^ The ability to prepare nucleoside analogues that incorporate modifications to the nucleobase and sugar moieties is vital for the analysis of nucleic acid structure^2,3^ and forms part of our molecular arsenal to develop therapeutics for the treatment of a range of cancers and viruses (Figure 1A).^1,4,5^ Underpinning these developments is the need for facile and chemoselective synthetic strategies to modify the nucleoside scaffold,^6,7^ particularly at a late stage in a synthetic sequence that can fine-tune physicochemical properties and efficacy.^8,9^ Difluoromethylation of small molecules is an effective strategy for this purpose.^10^ By virtue of the electron-withdrawing nature of the two fluorine atoms, the C–H bond of the -CF_2_H group is polarized.^11^ This polarization renders the difluoromethyl group a lipophilic bioisostere of a hydroxyl group.^12^

**Figure 1:**
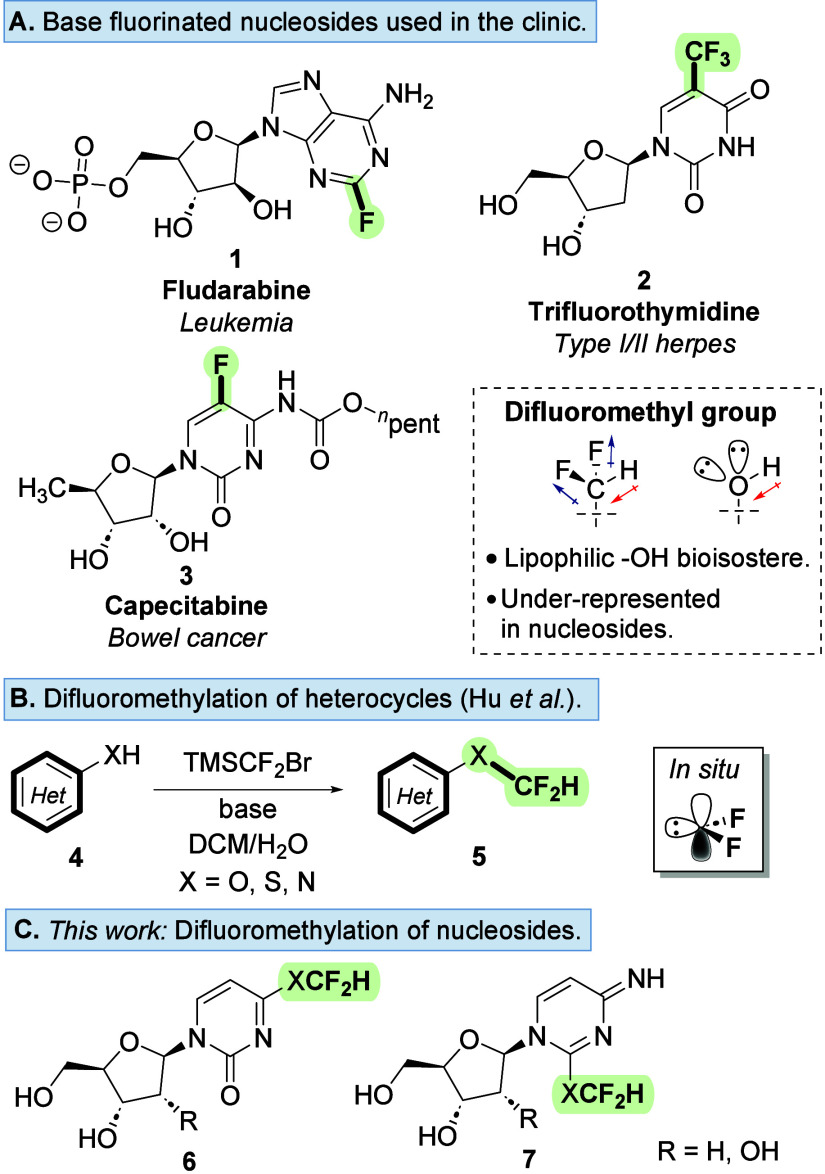
(A) Representative therapeutic fluorinated nucleosides. (B) Difluoromethylation of heterocycles via formation of difluorocarbene *in situ*.^27^ (C) Chemoselectivity reaction profiling platform for late-stage nucleoside difluoromethylation (this work).

While the installation of difluoromethyl groups has been used extensively throughout medicinal chemistry (Figure 1B),^13^ the utility of the difluoromethyl group in the development of novel nucleoside analogues has been limited.^14–16^ One prominent example is the late-stage difluoromethylation of the purine nucleobase via the formation of a difluoromethyl radical by photoredox catalysis.^17^ Whereas the addition of a radical to the purine and pyrimidine nucleobases produces difluoromethylated products via C–C bond formation,^18^ the electron deficient reactivity of difluorocarbenes tends to react with nucleophilic heteroatom sites,^19^ forming N,O,S-difluoromethylated products.^20–22^ A mild, late-stage difluoromethylation approach to modify nucleoside scaffolds would therefore provide a synthetic strategy to alter their overall physicochemical and functional properties.^20–22^

In this Letter, we establish a reactivity profile of the addition of difluoromethyl carbene to a series of nucleoside analogues (Figure 1C). We define the chemoselectivity and scope of difluoromethylation, and provide a rationale for the wider utility of difluoromethylation as a synthetic approach for nucleoside late-stage functionalization.

Reactivity profiling of the difluoromethylation of nucleosides was explored using conditions where the difluorocarbene is generated *in situ* using TMSCF_2_Br and a mild base.^23^ While previous work had established general reactivity of the addition of difluorocarbene to heterocycles, the complexity of the substrate was limited by poor selectivity.^24–26^

Consequently, only one method for the O-difluoromethylation of uridine nucleosides has been reported, but its applicability has been hindered by the requirement of highly toxic Hg­(CF_3_)_2_ as a difluoromethylating agent.^14^ Since the mild conditions of a reported two-phase reaction generate the difluorocarbene at the DCM–H_2_O interface,^20,27–30^ we chose to silyl protect the 3′-OH and 5′-OH groups to ensure solubility in DCM. Silyl-protected **8** was used to probe difluoromethylation of the uridine scaffold, whereas **9** and **10** were used to explore the reactivity of cytidine (Figure 2A).

**Figure 2:**
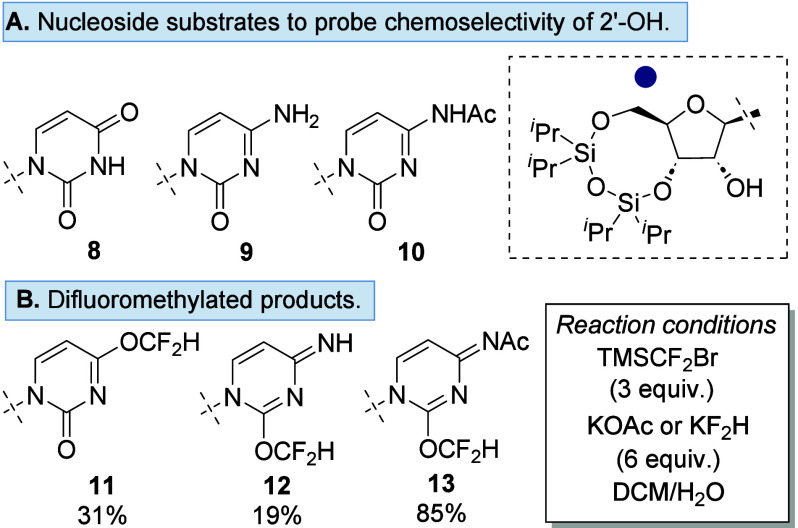
(A) 3′/5′-Silyl-protected nucleoside substrates used to explore chemoselective difluoromethylation. (B) Difluoromethylated nucleoside products.

Using the previously reported biphasic difluoromethylation conditions with **8** resulted in O-difluoromethylation at the 4-position (**11**) in 31% yield (Figure 2B).^23,31^ No O-difluoromethylation was observed on the 2′-OH or the 2-position of the nucleobase, with the remaining mass balance being associated with unreacted starting material. Furthermore, no competition from N3 was observed. In contrast with the 4-position being the desired nucleophilic site in **8**, both cytidine analogues **9** and **10** underwent O-difluoromethylation at the 2-position, affording **12** and **13** in 19% and 85% yields, respectively.^32^ No difluoromethylation was observed at the 2′-OH or N4 position, thus confirming the chemoselective nature of carbene addition. We surmise the difference in the yield of **12** versus acetyl-protected **13** is due to the stronger electron-withdrawing capabilities of the acetate group.

With nucleobase O-difluoromethylation being the preferred site of functionalization established with **9** and **10**, we further explored the chemoselectivity of difluoromethylation using an expanded series of ribonucleosides and 2′-deoxyribonucleosides **14–18** (Figure 3A). Difluoromethylation of oxygen-based uridine scaffolds (**14–16**) afforded O-difluoromethylated products at the 4-position (**19–21**) for both silyl-protected ribonucleosides (**14** and **16**) and 2′-deoxyribonucleosides (**15** (Figure 3B)). Thiouridines **17** and **18** alter the reactivity away from oxygen, forming S-difluoromethylated products **22** (30%) and **23** (35%), respectively.

**Figure 3:**
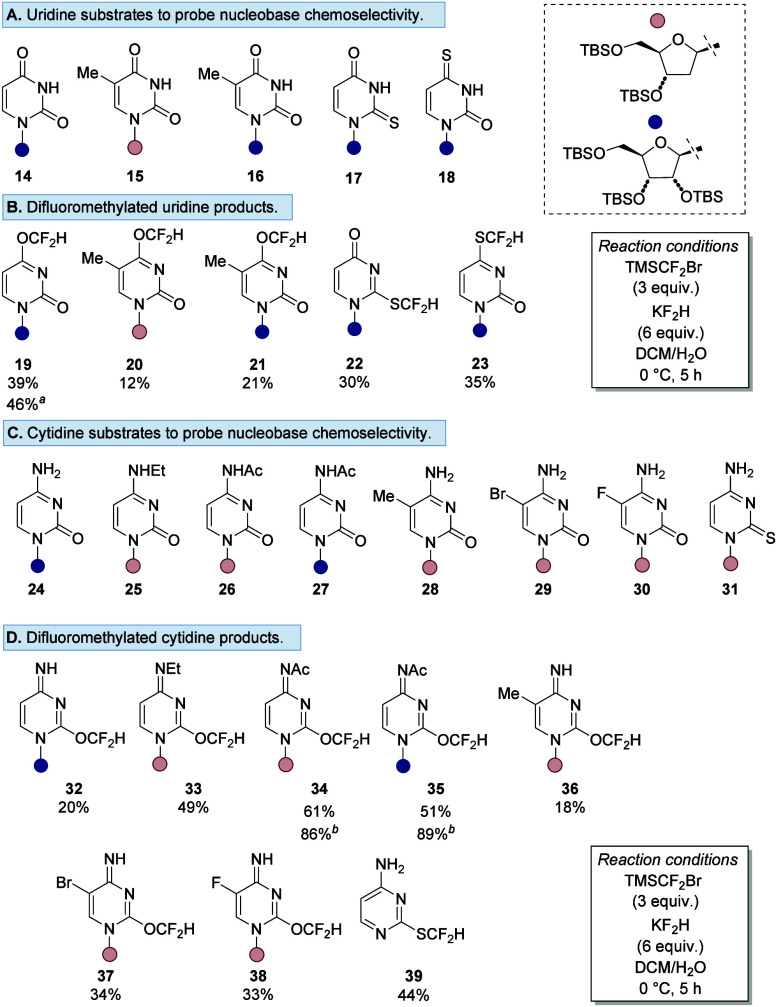
(A) Uridine substrates used to explore the chemoselectivity of the nucleobase and (B) difluoromethylated products. ^*a*^Yield of the 1 mmol scale reaction. (C) Cytidine substrates used to explore the chemoselectivity of the nucleobase and (D) difluoromethylated products. ^*b*^Conversion to the crude product after 1 h.

Further expansion of this series using **24–30** (Figure 3C) afforded O-difluoromethylated products **32–38** (Figure 3D). One exception was the difluoromethylation of **31**, which resulted in the cleavage of the glycosidic bond, affording **41** in 44% yield. Cytidine derivatives bearing N4 acetyl groups (**34**, **35**, and **10**) were formed to full conversion; however, partial deacetylation during column chromatography resulted in lower isolated yields (Figures S1–S4).

Although isolated yields were generally low, substantial recovery of starting materials was achieved, with no major byproducts isolated. Some degradation was observed for 2′-deoxynucleosides, particularly cytidine analogues, likely due to glycosidic bond cleavage. However, no N-difluoromethylated products were detected, suggesting either low reactivity or instability under the reaction and workup conditions.

With the chemoselectivity of difluoromethylation established, we explored the deprotection of a subset of these analogues (Scheme 1A). Using **35** as our cytidine exemplar, we observed the lability of the difluoromethyl group when fluoride reagents were used (entries 1–3) as well as with Dowex resin (entry 4). The use of acidic conditions resulted in the formation of deprotected nucleoside **40** (entries 5 and 6), with TFA/H_2_O identified as the optimal set of conditions, affording the desired product in 45% yield (entry 7). Expansion of these deprotection conditions using substrates **34** and **38** afforded the desired free nucleosides **41** and **42** in 50% and 23% yields, respectively (Scheme 1B).

**Scheme 1:**
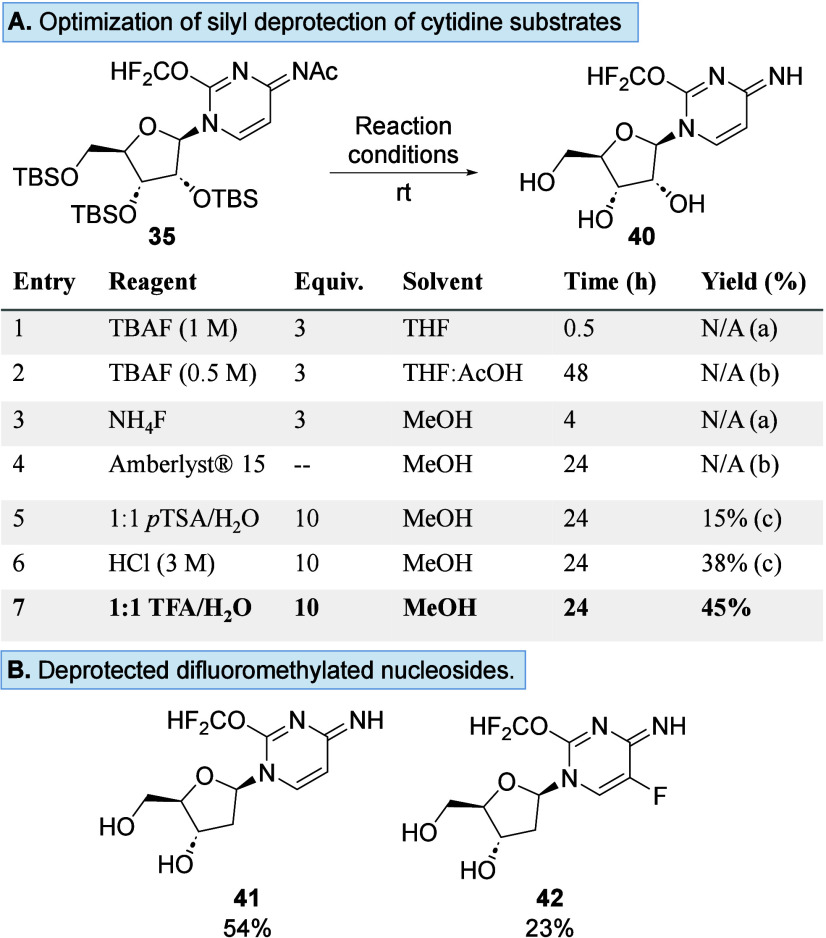
(A) Optimization of the Silyl Deprotection Condition to Form Nucleoside **40**, Where (a) Denotes Partial Hydrolysis of the Difluoromethyl Group, (b) Denotes Incomplete Deprotection, and (c) Denotes Isolation of the Product as a Salt, and (B) Scope of the Silyl Deprotection Condition

Further optimization was required for the silyl deprotection of the difluoromethylated uridine analogues (Scheme 2A). As with compound **35**, the difluoromethyl group in compound **19** was labile when fluoride reagents were used, although this effect was mediated by buffering with AcOH in this instance (entries 1 and 2). Compound **19** also demonstrated sensitivity to acid deprotection conditions (entries 3–5). The TEA/3HF mixture was the optimal silyl deprotection reagent for **19**, affording **43** in 48% yield. These conditions were also used to silyl deprotect **20**, affording **44** in 69% yield (Scheme 2B).

**Scheme 2:**
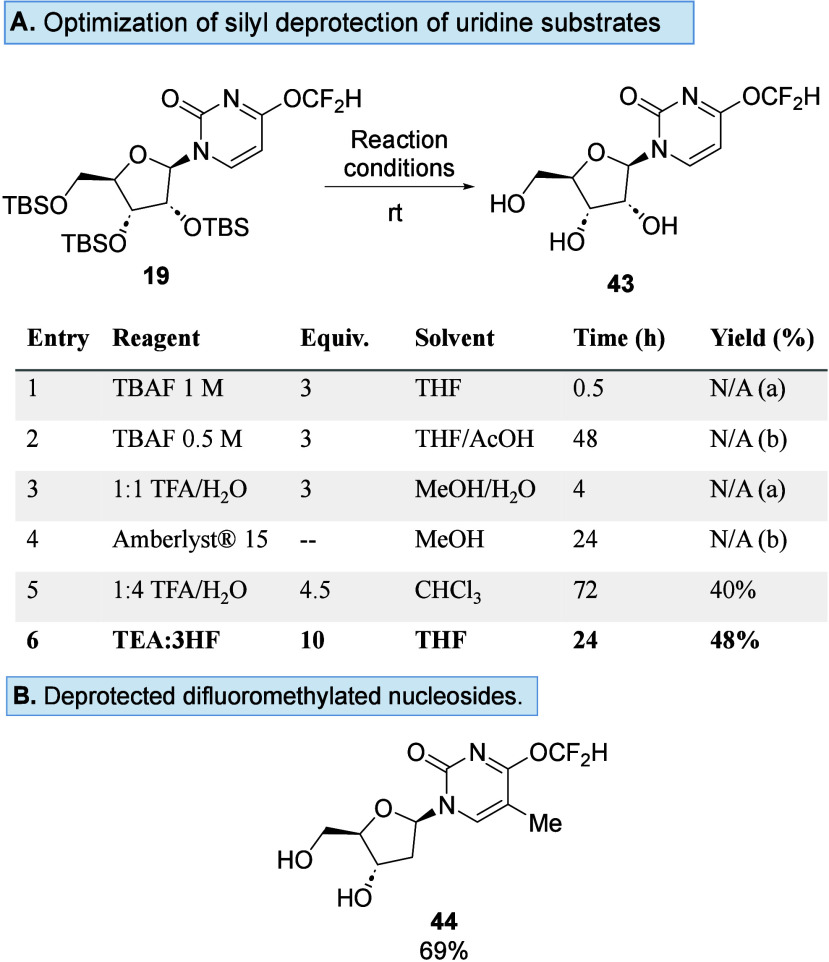
(A) Optimization of the Silyl Deprotection Condition to Form Nucleoside **43** and (B) Scope of the Silyl Deprotection Condition, Where (a) Denotes Partial Hydrolysis of the Difluoromethyl Group and (b) Denotes Incomplete Deprotection

Given the high conversions observed at each step in the synthesis of **41**, a telescoped approach was explored (Scheme 3). When performed on a 1.5 mmol scale with only aqueous workups between steps, **41** was obtained in 30% overall yield, requiring just a single purification across the sequence.

**Scheme 3:**
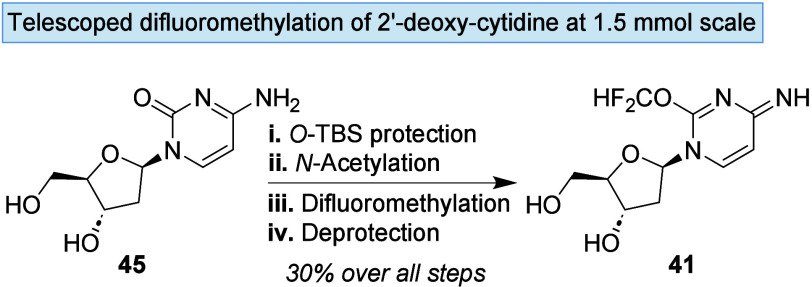
Reaction Conditions for Telescoped Reaction Series^a^

a Conditions: (i) TBS-Cl (4.5 equiv), imidazole (4.0 equiv), DMF, rt, 18 h, Ar; (ii) Ac_2_O (1.2 equiv), DMAP (10 mol %), pyridine, rt, 18 h, Ar; (iii) TMSCF_2_Br (3 equiv), KF_2_H (6 equiv), DCM, H_2_O, 0 °C, 1 h; (iv) TFA/H_2_O (16 equiv), MeOH, 0 °C, 4 h.

In summary, our profiling of difluoromethylation of nucleosides revealed O-difluoromethylation is preferred over N-difluoromethylation in both uridine and cytidine analogues. Reactivity toward S-difluoromethylation predominates when sulfonated analogues are used. When there are two competing nucleophilic oxygen sites (e.g., uridine series), O-difluoromethylation at position 4 is preferred. Silyl deprotection under acidic conditions accesses free difluoromethylated nucleoside products, and the telescoped procedures proved to be both practical and scalable. We envisage that the mild reaction conditions and readily accessible nucleoside starting materials will enable facile exploration of the broader biological properties of difluoromethylated nucleosides.

## Supplementary Material



## Data Availability

The data underlying this study are available in the published article and its Supporting Information and openly available in pure.strath.ac.uk at 10.15129/eb3345bf-8083-4ac1-9c02-fb5d47e67ee7.

## Notes

Experimental details and characterization data (NMR and HRMS) of the isolated products (PDF)
