# Supplementary material for: Chemoselective Difluoromethylation of Nucleosides
Source: Org Lett. 2025 Jun 17;27(25):6906–10. doi: 10.1021/acs.orglett.5c02204 (PMC12210264; doi:10.1021/acs.orglett.5c02204)
Supplement: Supplementary file 1 [file ol5c02204_si_001.pdf]

Supplemental Information

CHEMOSELECTIVE DIFLUOROMETHYLATION OF NUCLEOSIDES

Otto Linden,[1,2] Alexander Axer,[1,2] Andrea Taladriz-Sender,[1,2] Glenn A. Burley[1,2*]

[1] Department of Pure and Applied Chemistry, University of Strathclyde. Thomas Graham Building, 295 Cathedral Street, Glasgow, G1 1XL, U.K. Email: glenn.burley@strath.ac.uk

[2] Strathclyde Centre for Molecular Bioscience

Table of Contents

CHEMOSELECTIVE DIFLUOROMETHYLATION OF NUCLEOSIDES 1

1.1 General information 3

1.1.1 Reagents and Solvents 3

1.1.2 NMR Spectroscopy 3

1.1.3 Mass Spectrometry (MS) 3

1.2 Synthetic procedures 3

1.2.1 Crude NMR spectra following difluoromethylation of N-acetylated cytidines 30

1.3 References 32

1.4 Characterisation data 33

1.1 General information

1.1.1 Reagents and Solvents

All reagents and solvents were used as supplied from commercial sources and used without any further purification unless otherwise specified. Solvents were all HPLC grade and used without any further purification, unless otherwise specified. Thin layer chromatography (TLC) was performed using Merck silica plates coated with fluorescent indicator UV254. TLC plates were analysed under 254 nm UV light or developed in *p*-anisaldehyde solution. Normal-phase column chromatography was carried out manually using Fluorochem Silicagel 60A 40-63u or by Advion Interchim puriFlash® XS 530Plus using SiliaSep Flash Cartridges, Silica, 40 - 63 µm, 60 Å.

1.1.2 NMR Spectroscopy

NMR spectroscopy was carried out using a Bruker AVANCE III HD two channel 400 MHz or AVIII-HD-500 NMR Spectrometer. All chemical shifts (δ) in $CDCl_3$ were referenced at 7.26 ppm (^{1}H) and 77.06 ppm (^{13}C); in DMSO-d_6 at 2.50 ppm (^{1}H) and 39.52 ppm (^{13}C); in CD_3CN at 1.94 ppm (^{1}H) and 1.32 ppm (^{13}C); in CD_3OD at 3.31 ppm (^{1}H) and 49.00 ppm (^{13}C). Chemical shifts are reported in parts per million (ppm) and coupling constants are quoted in hertz (Hz). Abbreviations for splitting patterns are s (singlet), d (doublet), t (triplet), q (quartet) and m (multiplet). App (apparent) denotes signals in which similar *J* values have resulted in false equivalence. Structural assignments were made with additional information from COSY, HSQC, and HMBC experiments. All NMR data was processed using MestRenova 14.3.1 software.

1.1.3 Mass Spectrometry (MS)

HRMS spectra of small molecules were measured on a Bruker microTOFq or a ThermoScientific Exactive™ Plus Orbitrap High Resolution Mass Spectrometer.

1.2 Synthetic procedures

3,5-O-TIPDS-Uridine (8)

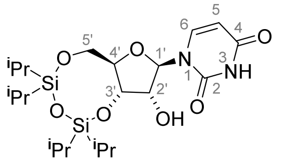

8

To a solution of uridine (250 mg, 1.03 mmol, 1.00 equiv) in anhydrous pyridine (10.0 mL) was added TIPDS-Cl_2 (347 mg, 1.10 mmol, 1.10 equiv) dropwise and the mixture was stirred at 0 °C under an argon atmosphere. After complete addition, the reaction was allowed to warm to rt and was stirred for

24 h, then concentrated and partitioned using DCM (30 mL) and water (30 mL). The organic layer was washed with brine (sat. aq., 20 mL) then dried over Na_2SO_4, filtered, and concentrated *in vacuo.* Purification by column chromatography (silica gel, 20% → 40% EtOAc in n-hexane) afforded **8** as a colourless foam (421 mg, 84%).

^{1}H NMR (500 MHz, $CDCl_3$): δ 8.70 (s, 1H, 3-NH), 7.68 (d, *J* = 8.1 Hz, 1H, 6-CH), 5.73 (s, 1H, 1′-CH), 5.69 (dd, *J* = 8.2, 1.9 Hz, 1H, 5-CH), 4.38 (dd, *J* = 8.7, 4.9 Hz, 1H, 2′-CH), 4.23 - 4.15 (m, 2H, 5′-CH_2), 4.15 - 4.07 (m, 1H, 4′-CH), 4.01 (dd, *J* = 13.2, 2.9 Hz, 1H, 3′-CH), 3.07 (d, *J* = 1.6 Hz, 1H, 2′-OH), 1.15 - 0.96 (m, 28H, 4 × ^{i}Pr).

^{13}C{^{1}H} NMR (126 MHz, $CDCl_3$) δ 163.7 (4-C=O), 150.4 (2-C=O), 140.0 (6-CH), 102.1 (5-CH), 91.1 (1′-CH), 82.0 (4′-CH), 75.3 (2′-CH), 68.9 (3′-CH), 60.3 (5′-CH), 17.6 (CH_3), 17.5 (CH_3), 17.4 (CH_3), 17.4 (CH_3), 17.2 (CH_3), 17.1 (CH_3), 17.0 (CH_3), 16.9 (CH_3), 13.5 (SiCH), 13.1 (SiCH), 13.0 (SiCH), 12.6 (SiCH).

These data are in accordance with literature values.[1]

3′,5′-O-TIPDS-Cytidine (9)

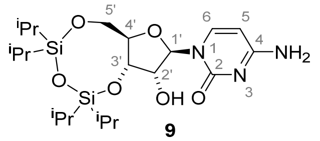

9

To a solution of cytidine (500 mg, 2.06 mmol, 1.00 equiv) in anhydrous pyridine (20.0 mL) was added TIPDS-Cl_2 (895 mg, 2.84 mmol, 1.38 equiv) dropwise under an argon atmosphere, and the mixture was stirred for 4 hours at 0 °C. Once determined complete by TLC, the mixture was concentrated *in vacuo*. Purification of **9** by column chromatography (silica gel, 3% → 7% MeOH in DCM) afforded **9** as a colourless foam (729 mg, 73%).

^{1}H NMR (400 MHz, $CDCl_3$) δ 7.81 (d, *J* = 7.5 Hz, 1H, 6-CH), 5.75 (s, 1H, 1′-CH), 5.68 (d, *J* = 7.5 Hz, 1H, 5-CH), 4.27 (dd, *J* = 8.9, 4.7 Hz, 1H, 3′-CH), 4.25 – 4.18 (m, 2H, 2′-CH, 5′- CH_aH$_b$), 4.15 (app dt, *J* = 9.0, 2.2 Hz, 1H, 4′-CH), 3.99 (dd, *J* = 13.2, 2.7 Hz, 1H, 5′-CH$_a$$H_b$), 3.48 (s, 1H, OH), 1.14 – 0.90 (m, 28H, 4 × ^{i}Pr).

^{13}C{^{1}H} NMR (101 MHz, $CDCl_3$) δ 166.1 (2-C=O), 155.9 (4-C-NH_2), 141.3 (6-CH), 94.0 (5-CH), 91.7 (1′-CH), 81.8 (4′-CH), 75.4 (2′-CH), 68.9 (3′-CH), 60.4 (5′-CH), 17.6 (CH_3), 17.6 (CH_3), 17.5 (CH_3), 17.5 (CH_3), 17.2 (CH_3), 17.2 (CH_3), 17.1 (CH_3), 17.0 (CH_3), 13.5 (SiCH), 13.1(SiCH), 13.0 (SiCH), 12.6 (SiCH).

These data are in accordance with literature values.[2]

4-N-Ac-3',5'-O-TIPDS-Cytidine (10)

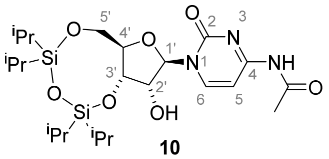

To a solution of **9** (303 mg, 620 μmol, 1.00 equiv) in anhydrous DMF (6.24 mL) was added Ac_2O (300 μL, 3.12 mmol, 5.00 equiv) and the mixture was stirred at rt for 6 h under an argon atmosphere. Once complete, the mixture was co-evaporated with MeOH (30 mL). Purification by column chromatography (silica gel, 3% MeOH in DCM) afforded **10** a colourless solid (227 mg, 69%).

^{1}H NMR (400 MHz, $CDCl_3$) δ 9.99 (s, 1H, NH), 8.18 (d, *J* = 7.5 Hz, 1H, 6-CH), 7.43 (d, *J* = 7.5 Hz, 1H, 5-CH), 5.81 (s, 1H, 1′-CH), 4.30 – 4.23 (m, 2H, 3′-CH, 5′-CH_aH_b), 4.22 – 4.16 (m, 2H, 4′-CH, 2′-CH), 4.01 (dd, *J* = 13.4, 2.7 Hz, 1H, 5′-CH_aH_b), 3.17 (s, 1H, 2′-OH), 2.28 (s, 3H, *CH_3*CO), 1.14 – 0.93 (m, 28H, 4 × ^{i}Pr).

^{13}C{^{1}H} NMR (101 MHz, $CDCl_3$) δ 171.1 (CH_3*C*=O), 163.2 (4-C=O), 155.1 (2-C=O), 144.5 (6-CH), 96.6 (5-CH), 91.7 (1′-CH), 82.2 (4′-CH), 75.3 (2′-CH), 68.7 (3′-CH), 60.1 (5′-CH_2), 25.1 (*CH_3*CO), 17.8 (CH_3), 17.6 (CH_3), 17.5 (CH_3), 17.4 (CH_3), 17.2(CH_3), 17.1 (CH_3), 17.0 (CH_3), 17.0 (CH_3), 13.5 (SiCH), 13.1 (SiCH), 13.1 (SiCH), 12.6 (SiCH).

These data are in accordance with literature values.[3]

4-O-CF_2H-3', 5'-O-TIPDS-Uridine (11)

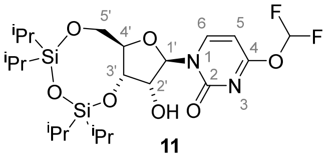

To a mixture of DCM (900 μL) and water (900 μL) was added **8** (726 mg, 1.50 mmol, 1.00 equiv) and KOAc (882 mg, 9.00 mmol, 6.00 equiv) and the mixture stirred vigorously. $TMSCF_2Br$ (690 μL, 4.50 mmol, 3.00 equiv) was added in 5 equal portions at 30 min intervals and allowed to stir for a further 3.5 h at 0 °C. Once complete, the reaction was diluted with DCM (5 mL) and water (50 mL) and separated. The aqueous phase was washed with DCM (3 × 30 mL) and the combined organic phase was dried over Na_2SO_4, filtered, and concentrated *in vacuo*. Purification by column chromatography (silica gel, 10% → 50% EtOAc in n-hexane) afforded **11** as a pale-yellow foam (296 mg, 31%).

^{1}H NMR (500 MHz, $CDCl_3$) δ 8.23 (d, *J* = 7.3 Hz, 1H, 6-CH), 7.51 (t, *J* = 71.0 Hz, 1H, 4-OCF_2H), 5.98 (d, *J* = 7.3 Hz, 1H, 5-CH), 5.78 (s, 1H, 1′-CH), 4.32 – 4.17 (m, 1H, 2′-CH), 4.32 – 4.17 (m, 1H, 4′-CH), 4.32 – 4.17 (m, 1H, 3′-CH), 4.32 – 4.17 (m, 1H, 5′-CH_aH_b), 4.01 (dd, *J* = 13.4, 2.7 Hz, 1H, 5′-CH_aH_b), 3.98 (s, 1H, OH), 1.11 – 0.97 (m, 28H, 4 × ^{i}Pr).

^{13}C{^{1}H} NMR (126 MHz, $CDCl_3$) δ 168.0 (4-*C*-OCF_2H), 154.0 (2-C=O), 145.7 (6-CH), 112.8 (t, J = 259.4 Hz, CF_2H), 94.0 (5-CH), 91.8 (1′-CH), 82.1 (2′-CH), 75.1 (4′-CH), 68.5 (3′ CH), 60.0 (5′-CH), 17.4 (CH_3), 17.4 (CH_3), 17.3 (CH_3), 17.0(CH_3), 17.0 (CH_3), 16.9 (CH_3), 16.8 (CH_3), 16.8 (CH_3), 13.4 (SiCH), 12.9 (SiCH), 12.6 (SiCH), 12.5 (SiCH).

^{19}F NMR (471 MHz, $CDCl_3$) δ −90.61 (dd, J = 176.7, 71.1 Hz, CF_aF_bH), −91.21 (dd, J = 176.8, 71.0 Hz CF_aF_bH).

HRMS-ESI Exact mass calculated for $[M + Na]^+$ ($C_{22}H_{38}F_2N_2O_7Si_2Na$) requires *m/z* 559.2078, found $[M + Na]^+$ *m/z* 559.2078.

2-O-CF₂H-3′,5′-O-TIPDS-Cytidine (12)

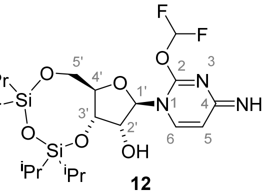

12

To a mixture of DCM (300 µL) and water (300 µL) was added **9** (227 mg, 500 µmol, 1.00 equiv) and KF_2H (234 mg, 3.00 mmol, 6.00 equiv) and the mixture was stirred vigorously. $TMSCF_2Br$ (230 µL, 1.50 mmol, 3.00 equiv) was added dropwise and the biphasic solution was stirred for 8 h at 0 °C. Once complete, the reaction was diluted with DCM (5 mL) and water (20 mL) and separated. Then, the aqueous phase was washed with DCM (3 × 20 mL) and the combined organic phase was dried over Na_2SO_4, filtered, and concentrated *in vacuo*. Purification by column chromatography (silica gel, 20% → 35% EtOAc in n-hexane) afforded **12** as a yellow oil (43 mg, 19%).

^{1}H NMR (400 MHz, $CDCl_3$) δ 9.10 (s, 1H, NH), 7.85 (t, J = 57.4 Hz, 1H, CF_2H), 7.58 (d, J = 8.3 Hz, 1H, 6-CH), 6.21 (d, J = 8.3 Hz, 1H, 5-CH), 5.74 (s, 1H, 1′-CH), 4.31 (dd, J = 8.8, 4.8 Hz, 1H, 3′-CH), 4.20 (dd, J = 13.4, 2.0 Hz, 1H, 5′-CH_aH_b), 4.16 (d, J = 4.8 Hz, 1H, 2′-CH), 4.11 (app dt, J = 8.8, 2.3 Hz, 1H, 4′-CH), 4.00 (dd, J = 13.4, 2.8 Hz, 1H, 5′-CH_aH_b), 2.86 (s, 1H, 2′-OH), 1.14 – 0.97 (m, 28H, 4 × ^{i}Pr).

^{13}C{^{1}H} NMR (101 MHz, $CDCl_3$) δ 154.9 (4-C=N), 146.7 (2-C-OCF_2H), 138.0 (6-CH), 108.55 (t, J = 256.9 Hz, CF_2H), 96.7 (5-CH), 91.0 (1′-CH), 82.3 (4′-CH), 75.3 (2′-CH), 69.0 (3′-CH), 60.1 (5′-CH_2), 17.5 (CH_3), 17.5 (CH_3), 17.4 (CH_3), 17.4 (CH_3), 17.2 (CH_3), 17.1 (CH_3), 17.0 (CH_3), 17.0 (CH_3), 13.5 (SiCH), 13.0 (SiCH), 12.7 (SiCH), 12.6 (SiCH).

^{19}F NMR (376 MHz, $CDCl_3$) δ −109.10 (dd, J = 230.3, 57.5 Hz, CF_aF_b), −109.78 (dd, J = 230.9, 57.7 Hz, CF_aF_b).

HRMS-ESI Exact mass calculated for $[M + H]^+$ ($C_{22}H_{40}F_2N_3O_6Si_2$) requires m/z 536.2418 found $[M + H]^+$ m/z 536.2435.

4-N-Ac-2-O-CF₂H-3',5'-O-TIPDS-Cytidine (13)

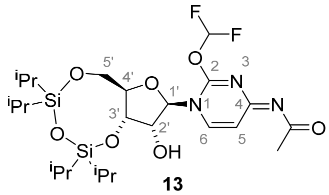

13

To a mixture of DCM (300 µL) and water (300 µL) was added **10** (227 mg, 460 µmol, 1.00 equiv) and KF_2H (227 mg, 2.75 mmol, 6.00 equiv) and the mixture stirred vigorously at 0 °C. $TMSCF_2Br$ (210 µL, 1.37 mmol, 3.00 equiv) was added dropwise and the biphasic solution was stirred at 0 °C for 1 h then, diluted with DCM (5 mL) and water (20 mL) and separated. The aqueous phase was washed with DCM (3 × 20 mL) and the combined organic phase was dried over Na_2SO_4, filtered, and concentrated *in vacuo*. Purification by column chromatography (silica gel, 15% → 20% EtOAc in n-hexane) afforded **13** as a colourless foam (212 mg, 85%).

^{1}H NMR (400 MHz, $CDCl_3$) δ 7.73 (t, *J* = 57.5 Hz, 1H, CF_2H), 7.39 (d, *J* = 8.3 Hz, 1H, 6-CH), 6.04 (d, *J* = 8.3 Hz, 1H, 5-CH), 5.72 (d, *J* = 0.7 Hz, 1H, 1′-CH), 4.32 (dd, *J* = 8.7, 4.9 Hz, 1H, 3′-CH), 4.17 (dd, *J* = 13.2, 2.2 Hz, 1H, 5′-CH_aH$_b$), 4.13 (d, *J* = 5.1 Hz, 1H, 2′-CH), 4.07 (app dt, *J* = 8.7, 2.5 Hz, 1H, 4′-CH), 3.99 (dd, *J* = 13.2, 2.9 Hz, 1H, 5′-CH$_a$$H_b$), 2.84 (s, 1H, OH), 2.22 (s, 3H, *CH₃*CO), 1.13 – 0.95 (m, 28H, 4 × ^{i}Pr).

^{13}C{^{1}H} NMR (101 MHz, $CDCl_3$) δ 183.1 (CH_3*C*=O) 149.6 (4-C=NH), 146.9 (2-*C*-OCF_2H), 136.1 (6-CH), 108.31 (t, *J* = 255.4 Hz, CF_2H), 96.6 (5-CH), 90.6 (1′-CH), 81.8 (4′-CH), 75.0 (2′-CH), 68.9 (3′-CH), 60.0 (5′-CH_2), 26.7 (CH_3CO), 17.2 (CH_3), 17.2 (CH_3), 17.1 (2 × CH_3), 16.9 (CH_3), 16.8 (CH_3), 16.7 (CH_3), 16.7 (CH_3), 13.2 (SiCH), 12.7 (2 × SiCH), 12.4 (SiCH).

^{19}F NMR (376 MHz, $CDCl_3$) δ −109.10 (dd, *J* = 230.3, 57.5 Hz, CF_aF$_b$), −109.78 (dd, *J* = 230.9, 57.7 Hz, CF$_a$$F_b$).

HRMS-ESI Exact mass calculated for $[M + Na]^+$ ($C_{24}H_{41}F_2N_3O_7Si_2Na$) requires m/z 600.2343, found $[M + Na]^+$ m/z 600.2343.

2',3',5'-tri-O-TBDMS-uridine (14)

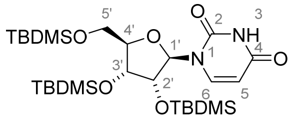

14

TBDMS-Cl (600 mg, 12.0 mmol, 6.00 equiv) was added to a stirred solution of uridine (488 mg, 2.00 mmol, 1.00 equiv) and imidazole (550 mg, 8.00 mmol, 4.00 equiv) in anhydrous DMF (20.0 mL) and the mixture stirred at rt for 18 h under an argon atmosphere. Once complete, the reaction mixture was concentrated *in vacuo* then partitioned between EtOAc (30 mL) and water (30 mL) and separated. The

organic phase was washed with water (3 × 40 mL) and LiCl (5%, aq., 20 mL), dried over Na_2SO_4, filtered, and concentrated *in vacuo*. Purification by column chromatography (silica gel, 15% → 50% EtOAc in n-hexane) afforded **14** as a colourless foam (113 mg, 24%).

^{1}H NMR (400 MHz, $CDCl_3$) δ 8.51 (s, 1H, NH), 8.01 (d, *J* = 8.2 Hz, 1H, 6-CH), 5.87 (d, *J* = 3.5 Hz, 1H, 1′-CH), 5.67 (dd, *J* = 8.1, 2.3 Hz, 1H, 5-CH), 4.12 – 4.04 (m, 3H, 2′-CH, 3′-CH, 4′-CH), 3.98 (dd, *J* = 11.9, 1.7 Hz, 1H, 5′-CH_aH_b), 3.76 (dd, *J* = 11.7, 1.1 Hz, 1H, 5′-CH_aH_b), 0.94 (s, 9H, Si^{t}Bu), 0.91 (s, 9H, Si^{t}Bu), 0.89 (s, 9H, Si^{t}Bu), 0.13 (s, 3H, $SiCH_3$), 0.12 (s, 3H, $SiCH_3$), 0.09 (s, 3H, $SiCH_3$), 0.08 (s, 3H, $SiCH_3$), 0.08 (s, 3H, $SiCH_3$), 0.07 (s, 3H, $SiCH_3$).

^{13}C{^{1}H} NMR (101 MHz, $CDCl_3$) δ 163.2 (4-C=O), 150.3 (2-C=O), 140.5 (6-CH), 102.0 (6-CH), 89.0 (1′-CH), 84.7 (4′-CH), 76.3 (2′-CH), 71.0 (3′-CH), 62.0 (5′-CH_2), 26.2 (SiC*C*H_3), 26.2 (SiC*C*H_3), 26.2 (SiC*C*H_3), 26.0 (SiC*C*H_3), 26.0 (SiC*C*H_3), 26.0 (SiC*C*H_3), 25.9 (SiC*C*H_3), 25.9 (SiC*C*H_3), 25.9 (SiC*C*H_3), 18.6 (SiC), 18.2 (SiC), 18.1 (SiC), −4.1 ($SiCH_3$), −4.4 ($SiCH_3$), −4.6 ($SiCH_3$), −4.7 ($SiCH_3$), −5.2 ($SiCH_3$), −5.4 ($SiCH_3$).

These data are in accordance with literature values.[4]

3′,5′-di-O-TBDMS-thymidine (15)

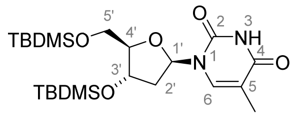

15

TBDMS-Cl (274 mg, 1.82 mmol, 2.20 equiv) was added to a stirred solution of cytidine (200 mg, 830 μmol, 1.00 equiv) and imidazole (225 mg, 3.30 mmol, 4.00 equiv) in anhydrous DMF (1.5 mL) and the mixture stirred at rt for 18 h under an argon atmosphere. Once complete, the reaction mixture was concentrated *in vacuo* then partitioned between EtOAc (10 mL) and water (10 mL) and separated. The organic phase was washed with water (3 × 10 mL) and LiCl (5%, aq., 10 mL), dried over Na_2SO_4, filtered, and concentrated *in vacuo*. Purification by column chromatography (silica gel, 1% → 4% MeOH in DCM) afforded **15** as a colourless foam (350 mg, 90%).

^{1}H NMR (400 MHz, $CDCl_3$) δ 9.53 (s, 1H, 3-NH), 7.44 (d, *J* = 1.2 Hz, 1H, 6-CH), 6.32 (dd, *J* = 7.9, 5.8 Hz, 1H, 1′-CH), 4.38 (app dt, *J* = 5.5, 2.5 Hz, 1H, 3′-CH), 3.91 (app q, *J* = 2.5 Hz, 1H, 4′-CH), 3.85 (dd, *J* = 11.2, 2.5 Hz, 1H, 5′-CH_aH_b), 3.74 (app dt, *J* = 11.2, 1.7 Hz, 1H, 5′-CH_aH_b), 2.23 (ddd, *J* = 13.1, 5.9, 2.7 Hz, 1H, 2′-CH_aH_b), 1.98 (ddd, *J* = 12.7, 7.8, 6.1 Hz, 1H, 2′-CH_aH_b), 1.89 (app t, *J* = 1.2 Hz, 3H, 5-CH_3), 0.90 (s, 9H, Si^{t}Bu), 0.87 (s, 9H, Si^{t}Bu), 0.09 (s, 6H, 3 × $SiCH_3$), 0.06 (s, 3H, $SiCH_3$), 0.05 (s, 3H, $SiCH_3$).

^{13}C{^{1}H} NMR (101 MHz, $CDCl_3$) δ 164.2 (4-C=O), 150.6 (2-C=O), 135.5 (6-CH), 110.9 (5-C), 87.9 (4′-CH), 84.9 (1′-CH), 72.3 (3′-CH), 63.0 (5′-CH_2), 41.5 (2′-CH_2), 26.0 (3 × SiC*C*H_3), 25.8 (3 × SiC*C*H_3), 18.5 (SiC), 18.1 (SiC), 12.6 (5-CH_3), −4.6 ($SiCH_3$), −4.8 ($SiCH_3$), −5.3 ($SiCH_3$), −5.4 ($SiCH_3$).

These data are in accordance with literature values.[5]

2',3',5'-tri-O-TBDMS-5-methyl-uridine (16)

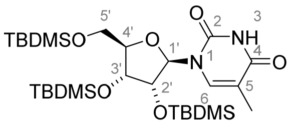

16

TBDMS-Cl (695 mg, 4.61 mmol, 12.0 equiv) was added to a stirred solution of 5-methyl-uridine (99.0 mg, 380 µmol, 1.00 equiv) and imidazole (105 mg, 1.54 mmol, 4.00 equiv) in anhydrous DMF (930 µL) and the mixture stirred at rt for 18 h under an argon atmosphere. Once complete, the reaction mixture was concentrated *in vacuo* then partitioned between EtOAc (10 mL) and water (10 mL) and separated. The organic phase was washed with water (3 × 10 mL) and LiCl (5%, aq., 10 mL), dried over Na_2SO_4, filtered, and concentrated *in vacuo*. Purification by column chromatography (silica gel, 15% → 50% EtOAc in n-hexane) afforded **16** as a colourless foam (61 mg, 26%).

^{1}H NMR (400 MHz, $CDCl_3$) δ 8.61 (s, 1H, 3-NH), 7.46 (d, *J* = 1.3 Hz, 1H, 6-CH), 6.02 (d, *J* = 6.4 Hz, 1H, 1′-CH), 4.12 (dd, *J* = 6.5, 4.6 Hz, 1H, 2′-CH), 4.06 (dd, *J* = 4.6, 2.3 Hz, 1H, 3′-CH), 4.02 (app q, *J* = 2.3 Hz, 1H, 4′-CH), 3.89 (dd, *J* = 11.6, 2.3 Hz, 1H, 5′-CH_aH_b), 3.73 (dd, *J* = 11.5, 2.1 Hz, 1H, 5′-CH_aH_b), 1.92 (d, *J* = 1.2 Hz, 3H, 5-CH_3), 0.96 (s, 9H, Si^{t}Bu), 0.91 (s, 9H, Si^{t}Bu), 0.85 (s, 9H, Si^{t}Bu), 0.14 (s, 3H, $SiCH_3$), 0.13 (s, 3H, $SiCH_3$), 0.09 (s, 3H, $SiCH_3$), 0.07 (s, 3H, $SiCH_3$), 0.01 (s, 3H, $SiCH_3$), −0.05 (s, 3H, $SiCH_3$).

^{13}C{^{1}H} NMR (101 MHz, $CDCl_3$) δ 163.8 (4-C=O), 150.5 (2-C=O), 135.9 (6-CH), 111.0 (5-CH), 87.5 (1′-CH), 86.1 (4′-CH), 75.4 (2′-CH), 72.5 (3′-CH), 63.3 (5′-CH_2), 26.2 (3 × SiC*C*H$_3$), 26.0 (3 × SiC*C*H$_3$), 25.8 (3 × SiC*C*H$_3$), 18.7 (SiC), 18.2 (SiC), 18.1 (SiC), 12.5 (5-CH_3), −4.3 ($SiCH_3$), −4.4 ($SiCH_3$), −4.6 ($SiCH_3$), −4.7 ($SiCH_3$), −5.2 ($SiCH_3$), −5.2 ($SiCH_3$).

HRMS-ESI Exact mass calculated for $[M+Na]^+$ ($C_{28}H_{56}N_2O_6Si_3Na$) requires *m/z* 623.3344, found $[M + Na]^+$ *m/z* 623.3326.

2',3',5'-tri-O-TBDMS-2-thio-uridine (17)

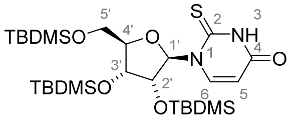

17

TBDMS-Cl (521 mg, 3.46 mmol, 9.00 equiv) was added to a stirred solution of 2-thio-uridine (488 mg, 380 µmol, 1.00 equiv) and imidazole (105 mg, 1.54 mmol, 4.00 equiv) in anhydrous DMF (3.84 mL) and the mixture stirred at rt for 18 h under an argon atmosphere. Once complete, the reaction mixture was concentrated *in vacuo* and partitioned between EtOAc (10 mL) and water (10 mL) and separated. The organic phase was washed with water (3 × 10 mL) and LiCl (5%, aq., 10 mL), dried over Na_2SO_4,

filtered, and concentrated *in vacuo*. Purification by column chromatography (silica gel, 15% → 50% EtOAc in n-hexane) afforded **17** as a yellow foam (77.0 mg, 34%).

^{1}H NMR (400 MHz, $CDCl_3$) δ 9.39 (s, 1H, 3-NH), 8.31 (d, *J* = 8.2 Hz, 1H, 6-CH), 6.44 (d, *J* = 2.8 Hz, 1H, 1′-CH), 5.92 (d, *J* = 8.2 Hz, 1H, 5-CH), 4.21 (dd, *J* = 3.9, 2.9 Hz, 1H, 2′-CH), 4.16 – 4.10 (m, 1H, 4′-CH), 4.12 – 4.01 (m, 2H, 3′-CH, 5′-CH_aH$_b$), 3.80 (dd, *J* = 11.9, 1.4 Hz, 1H, 5′-CH$_a$$H_b$), 0.95 (s, 9H, Si^{t}Bu), 0.91 (s, 9H, Si^{t}Bu), 0.91 (s, 9H, Si^{t}Bu), 0.16 (s, 3H, $SiCH_3$), 0.14 (s, 3H, $SiCH_3$), 0.13 (s, 3H, $SiCH_3$), 0.11 (s, 3H, $SiCH_3$), 0.10 (s, 3H, $SiCH_3$), 0.08 (s, 3H, $SiCH_3$).

^{13}C{^{1}H} NMR (101 MHz, $CDCl_3$) δ 175.8 (4-C=O), 159.3 (2-C=S) 141.2 (6-CH), 106.3 (5-CH), 93.3 (1′-CH), 84.3 (4′-CH), 77.09 (2′-CH), 70.5 (3′-CH), 61.4 (5′-CH_2), 26.2 (4 × SiC*C*H_3), 26.1 (5 × SiC*C*H_3), 18.7 (2 × SiC), 18.3 (SiC), −3.7 ($SiCH_3$), −3.9 ($SiCH_3$), −4.5 ($SiCH_3$), −4.7 ($SiCH_3$), −5.1 ($SiCH_3$), −5.5 ($SiCH_3$).]

HRMS-ESI Exact mass calculated for [M − TBDMS]$^-$ ($C_{21}H_{39}N_2O_5SSi_2$) requires *m/z* 487.2124, found [M − TBDMS]$^-$ *m/z* 487.2115.

2′,3′,5′-tri-O-TBDMS-4-thio-uridine (18)

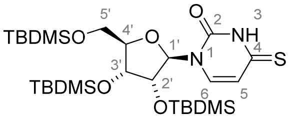

18

TBDMS-Cl (521 mg, 3.46 mmol, 9.00 equiv) was added to a stirred solution of 4-thio-uridine (100 mg, 380 µmol, 1.00 equiv) and imidazole (105 mg, 1.54 mmol, 4.00 equiv) in anhydrous DMF (930 µL) and the mixture stirred at rt for 18 h under an argon atmosphere. Once complete, the reaction mixture was concentrated *in vacuo* and partitioned between EtOAc (10 mL) and water (10 mL) and separated. The organic phase was washed with water (3 × 10 mL) and LiCl (5%, aq., 10 mL), dried over Na_2SO_4, filtered, and concentrated *in vacuo*. Purification by column chromatography (silica gel, 15% → 50% EtOAc in n-hexane) afforded **18** as a yellow solid foam (185 mg, 82%).

^{1}H NMR (400 MHz, $CDCl_3$) δ 9.33 (s, 1H, NH), 7.69 (d, *J* = 7.7 Hz, 1H, 6-CH), 6.38 (dd, *J* = 7.7, 2.0 Hz, 1H, 5-CH), 5.92 (d, *J* = 4.3 Hz, 1H, 1′-CH), 4.31 – 4.24 (m, 1H, 3′-CH), 4.04 (app ddt, *J* = 6.8, 4.7, 2.3 Hz, 2H, 2′-CH, 4′-CH), 3.96 (ddd, *J* = 11.5, 9.1, 2.3 Hz, 1H, 5′-CH_aH$_b$), 3.75 (dd, *J* = 11.7, 1.8 Hz, 1H, 5′-CH_aH$_b$), 0.93 (s, 28H, 3 × Si^{t}Bu), 0.15 (s, 3H, $SiCH_3$), 0.14 (s, 3H, $SiCH_3$), 0.12 (s, 12H, 3 × $SiCH_3$).

^{13}C{^{1}H} NMR (101 MHz, $CDCl_3$) δ 165.7 (4-C=S), 147.6 (2-C=O), 134.8 (6-CH), 113.3 (5-CH), 89.5 (1′-CH), 85.3 (4′-CH), 75.8 (2′-CH), 71.1 (3′-CH), 62.2 (5′-CH_2), 29.9 (3 × SiC*C*H_3), 26.1 (6 × SiC*C*H_3), 25.8 (3 × SiC*C*H_3), 18.5 (SiC), 18.2 (2 × SiC), −4.5 ($SiCH_3$), −4.5 ($SiCH_3$), −4.6 ($SiCH_3$), −4.6 ($SiCH_3$), −5.3 ($SiCH_3$), −5.3 ($SiCH_3$).

HRMS-ESI Exact mass calculated for [M − TBDMS]$^-$ ($C_{21}H_{39}N_2O_5SSi_2$) requires *m/z* 487.2124, found [M − TBDMS]$^-$ *m/z* 487.2115.

2',3',5'-tri-O-TBDMS-4-O-difluoromethyl-uridine (19)

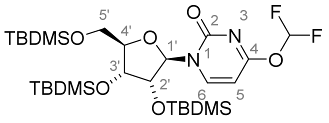

19

To a mixture of DCM (300 µL) and water (300 µL) was added **14** (300 mg, 500 µmol, 1.00 equiv) and KF_2H (231 mg, 3.00 mmol, 6.00 equiv) and the mixture was stirred vigorously. $TMSCF_2Br$ (230 µL, 1.50 mmol, 3.00 equiv) was added dropwise and the biphasic solution stirred for 5 h at 0 °C then diluted with DCM (5 mL) and water (20 mL) and separated. The aqueous phase was extracted with DCM (3 × 20 mL) and the combined organic phase was dried over Na_2SO_4, filtered, and concentrated *in vacuo*. Purification by column chromatography (silica gel, 5% → 20% EtOAc in n-hexane) afforded **19** as a colourless foam (124 mg, 39%).

Reaction at 1 mmol scale:

To a mixture of DCM (600 µL) and water (600 µL) was added **14** (587 mg, 1.00 mmol, 1.00 equiv) and KF_2H (463 mg, 6.00 mmol, 6.00 equiv) and the mixture was stirred vigorously. $TMSCF_2Br$ (467 µL, 3.00 mmol, 3.00 equiv) was added dropwise and the biphasic solution stirred for 5 h at 0 °C then diluted with DCM (10 mL) and water (40 mL) and separated. The aqueous phase was extracted with DCM (3 × 40 mL) and the combined organic phase was dried over Na_2SO_4, filtered, and concentrated *in vacuo*. Purification by column chromatography (silica gel, 5% → 20% EtOAc in n-hexane) afforded **19** as a colourless foam (287 mg, 46%).

^{1}H NMR (400 MHz, $CDCl_3$) δ 8.66 (d, J = 7.3 Hz, 1H, (6-CH), 7.50 (t, J = 71.1 Hz, 1H, 2-OCF_2H), 5.92 (d, J = 7.3 Hz, 1H, 5-CH), 5.70 (s, 1H, 1′-CH), 4.20 – 4.07 (m, 3H, 2′-CH, 4′-CH, 5′-C$H_a$$H_b$), 4.01 (dd, J = 8.3, 3.8 Hz, 1H, 3′-CH), 3.79 (dd, J = 11.9, 1.3 Hz, 1H, 5′-C$H_a$$H_b$), 0.94 (s, 9H, Si^{t}Bu), 0.91 (s, 9H, Si^{t}Bu), 0.24 (s, 9H, Si^{t}Bu), 0.13 (s, 3H, $SiCH_3$), 0.12 (s, 3H, $SiCH_3$), 0.11 (s, 3H, $SiCH_3$), 0.04 (s, 3H, $SiCH_3$), 0.03 (s, 3H, $SiCH_3$).

^{13}C{^{1}H} NMR (101 MHz, $CDCl_3$) δ 167.8 (t, 3.1 Hz, 4-*C*-OCF_2H), 154.4 (2-C=O), 146.4 (6-CH), 112.9 (t, J = 259.0 Hz, OCF_2H), 93.6 (6-CH), 91.6 (1′-CH), 83.0 (4′-CH), 76.2 (2′-CH), 68.6 (3′-CH), 60.5 (5′-CH_2), 26.2 (SiC*C*H_3), 26.2 (SiC*C*H_3), 26.2 (SiC*C*H_3), 26.0 (SiC*C*H_3), 26.0 (SiC*C*H_3), 26.0 (SiC*C*H_3), 25.9 (SiC*C*H_3), 25.9 (SiC*C*H_3), 25.9 (SiC*C*H_3), 18.7 (SiC), 18.2 (SiC), −3.9 ($SiCH_3$), −4.1 ($SiCH_3$), −5.0 ($SiCH_3$), −5.0 ($SiCH_3$), −5.1 ($SiCH_3$), −5.1 ($SiCH_3$), −5.5 ($SiCH_3$).

^{19}F NMR (376 MHz, $CDCl_3$) δ −90.8 (d, J = 71.2 Hz, C$F_a$$F_b$H), −91.1 (d, J = 71.0 Hz, C$F_a$$F_b$H).

HRMS-ESI Exact mass calculated for $[M+Na]^+$ ($C_{28}H_{54}F_2N_2O_6Si_3Na$) requires *m/z* 659.3155, found $[M + Na]^+$ *m/z* 659.3140.

3′,5′-di-O-TBDMS-4-O-difluoromethyl-thymidine (20)

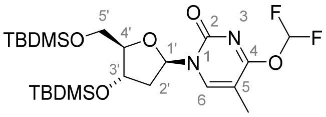

20

To a mixture of DCM (120 µL) and water (120 µL) was added **15** (94.0 mg, 200 µmol, 1.00 equiv) and KF_2H (94.0 mg, 1.20 mmol, 6.00 equiv) and the mixture was stirred vigorously. $TMSCF_2Br$ (94.0 µL, 600 µmol, 3.00 equiv) was added dropwise and the biphasic solution was stirred for 5 h at 0 °C then diluted with DCM (2 mL) and water (10 mL) and separated. The aqueous phase was extracted with DCM (3 × 10 mL) and the combined organic phase was dried over Na_2SO_4, filtered, and concentrated *in vacuo*. Purification by column chromatography (silica gel, 5% → 40% EtOAc in n-hexane) afforded **20** as a yellow foam (13.0 mg, 12%).

^{1}H NMR (400 MHz, $CDCl_3$) δ 8.00 (d, J = 1.0 Hz, 1H, 6-CH), 7.55 (t, J = 71.3 Hz, 1H, 4-OCF_2H), 6.26 (app t, J = 6.3 Hz, 1H, 1′-CH), 4.37 (app dt, J = 6.7, 3.6 Hz, 1H, 4′-CH), 3.99 (app dt, J = 3.5, 2.5 Hz, 1H, 3′-CH), 3.93 (dd, J = 11.5, 2.5 Hz, 1H, 5′-CH_aH_b), 3.77 (dd, J = 11.5, 2.5 Hz, 1H, 5′-CH_aH_b), 2.53 (ddd, J = 13.4, 6.2, 3.8 Hz, 1H, 2′-CH_aH_b), 2.01 (d, J = 1.0 Hz, 3H, 5-Me), 2.01 (app dt, J = 12.9, 6.4 Hz, 1H, 2′-CH_aH_b), 0.92 (s, 9H, Si^{t}Bu), 0.89 (s, 9H, Si^{t}Bu), 0.11 (s, 3H, $SiCH_3$), 0.10 (s, 3H, $SiCH_3$), 0.08 (s, 3H, $SiCH_3$), 0.07 (s, 3H, $SiCH_3$).1

^{13}C{^{1}H} NMR (101 MHz, $CDCl_3$) δ 166.8 (t, 4.6 Hz, 4-*C*-OCF_2H), 154.4 (2-C=O), 143.0 (6-CH), 113.2 (t, J = 258.6 Hz, 4-OCF_2H), 103.1 (5-CH), 88.5 (4′-CH), 87.3 (1′-CH), 71.7 (3′-CH), 62.7 (5′-CH_2), 42.6 (2′-CH_2), 26.1 (3 × SiC*C*H_3), 25.9 (3 × SiC*C*H_3), 18.6 (SiC), 18.2 (SiC), 12.0 (5-CH_3), −4.4 ($SiCH_3$), −4.7 ($SiCH_3$), −5.2 (2 × $SiCH_3$).

^{19}F NMR (376 MHz, $CDCl_3$) δ −90.42 (dd, J = 176.2, 71.4 Hz, CF_aF_bH), −91.15 (dd, J = 176.2, 71.2 Hz, CF_aF_bH).

HRMS-ESI Exact mass calculated for $[M + Na]^+$ ($C_{23}H_{42}F_2N_2O_5Si_2Na$) requires *m/z* 543.2498, found $[M + Na]^+$ *m/z* 542.2512.

2′,3′,5′-O-TBDMS-4-O-difluoromethyl-5-methyl-uridine (21)

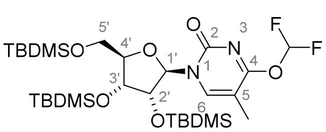

21

To a mixture of DCM (60.0 µL) and water (60.0 µL) was added **16** (61.0 mg, 100 µmol, 1.00 equiv) and KF_2H (47.0 mg, 600 µmol, 6.00 equiv) and the mixture was stirred vigorously. $TMSCF_2Br$ (47.0 µL, 300 µmol, 3.00 equiv) was added dropwise and the biphasic solution was stirred for 5 h at 0 °C then diluted with DCM (1 mL) and water (5 mL) and separated. The aqueous phase was extracted with DCM (3 × 5 mL) and the combined organic phase was dried over Na_2SO_4, filtered, and concentrated *in vacuo*.

Purification by column chromatography (silica gel, 5% → 20% EtOAc in n-hexane) afforded **21** as a colourless foam (13.8 mg, 21%).

^{1}H NMR (400 MHz, $CDCl_3$) δ 8.07 (d, *J* = 1.1 Hz, 1H, 6-CH), 7.54 (t, *J* = 71.2 Hz, 1H, CF_2H), 5.83 (d, *J* = 2.8 Hz, 1H, 1′-CH), 4.17 – 4.12 (m, 2H, 2′-CH, 4′-CH), 4.07 (dd, *J* = 11.8, 2.3 Hz, 1H, 5′-C CH_aH_b), 4.00 (dd, *J* = 6.5, 4.2 Hz, 1H, 3′-CH), 3.80 (dd, *J* = 11.8, 2.1 Hz, 1H, 5′-CH_aH_b), 2.02 (d, *J* = 1.0 Hz, 3H 5-C-CH_3), 0.97 (s, 9H, Si^{t}Bu), 0.90 (Si^{t}Bu, s, 9H), 0.90 (Si^{t}Bu, s, 9H), 0.16 (s, 3H, $SiCH_3$), 0.14 (s, 3H, $SiCH_3$), 0.14 (s, 3H, $SiCH_3$), 0.09 (s, 3H, $SiCH_3$), 0.07 (s, 3H, $SiCH_3$), 0.06 (s, 3H, $SiCH_3$).

^{13}C{^{1}H} NMR (101 MHz, $CDCl_3$) δ 166.8 (t, *J* = 3 Hz, 4-*C*-OCF_2H), 154.5 (2-C=O), 143.2 (6-CH), 113.2 (t, *J* = 259.0 Hz, OCF_2H), 103.1 (5-C), 90.9 (1′-CH), 84.1 (4′-CH), 76.4 (2′-CH), 70.1 (3′-CH), 61.7 (5′-CH), 26.4 (3 × SiC*C*H_3), 26.0 (6 × SiC*C*H_3), 19.0 (2 × SiC), 18.2 (SiC), 11.9 (5-CH_3), −3.9 ($SiCH_3$), −4.2 ($SiCH_3$), −4.7 ($SiCH_3$), −4.8($SiCH_3$), −4.9 ($SiCH_3$), −5.3 ($SiCH_3$).

^{19}F NMR (376 MHz, $CDCl_3$) δ −90.46 (dd, *J* = 176.2, 71.4 Hz, CF_aF_bH), −91.25 (dd, *J* = 176.2, 71.1 Hz, CF_aF_bH).

HRMS-ESI Exact mass calculated for $[M + H]^+$ ($C_{29}H_{57}F_2N_2O_6Si_3$) requires *m/z* 651.3492, found $[M + H]^+$ *m/z* 651.3482.

2′,3′,5′-tri-O-TBDMS-2-S-difluoromethyl-2-thio-uridine (22)

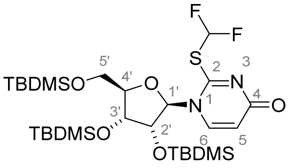

22

To a mixture of DCM (80 µL) and water (80 µL) was added **17** (77.0 mg, 130 µmol, 1.00 equiv) and KF_2H (59.0 mg, 770 µmol, 6.00 equiv) and the mixture was stirred vigorously. $TMSCF_2Br$ (59.6 µL, 380 µmol, 3.00 equiv) was added dropwise and the biphasic solution was stirred for 5 h at 0 °C then diluted with DCM (1 mL) and water (5 mL) and separated. The aqueous phase was extracted with DCM (3 × 5 mL) and the combined organic phase was dried over Na_2SO_4, filtered, and concentrated *in vacuo*. Purification by column chromatography (silica gel, 5% → 20% EtOAc in n-hexane) afforded **22** as a colourless foam (25.4 mg, 30%).

^{1}H NMR (400 MHz, $CDCl_3$) δ 7.95 (d, *J* = 7.8 Hz, 1H, 6-CH), 7.83 (t, *J* = 55.2 Hz, 1H, SCF_2H), 6.13 (d, *J* = 7.8 Hz, 1H, 5-CH), 5.63 (d, *J* = 7.1 Hz, 1H, 1′-CH), 4.14 (dd, *J* = 7.1, 4.4 Hz, 1H, 3′-CH), 4.11 – 4.02 (m, 2H, 4′-CH, 2′-CH), 3.87 (dd, *J* = 11.7, 2.3 Hz, 1H, 5′-CH_aH_b), 3.73 (dd, *J* = 11.7, 1.7 Hz, 1H, 5′-CH_aH_b), 0.94 (s, 9H, Si^{t}Bu), 0.93 (s, 9H, Si^{t}Bu), 0.87 (s, 9H, Si^{t}Bu), 0.13 (s, 3H, $SiCH_3$), 0.13 (s, 3H, $SiCH_3$), 0.12 (s, 3H, $SiCH_3$), 0.08 (s, 3H, $SiCH_3$), -0.01 (s, 3H, $SiCH_3$), -0.15 (s, 3H, $SiCH_3$).

^{13}C{^{1}H} NMR (101 MHz, $CDCl_3$) δ 167.1 (4-C=O), 158.1 (t, *J* = 5.3 Hz, 2-S-CF_2H), 138.6 (6-CH), 120.8 (t, *J* = 174.4 Hz, SCF_2H), 111.4 (5-CH), 91.2 (1′-CH), 88.1 (4′-CH), 77.0 (2′-CH), 73.5 (3′-CH),

63.6 (5′-CH_2), 26.1 (3 × SiCCH_3), 25.9 (6 × SiCCH_3), 18.5 (SiC), 18.2 (SiC), 18.0 (SiC), −4.3 ($SiCH_3$), −4.4 ($SiCH_3$), −4.4 ($SiCH_3$), −4.9 ($SiCH_3$), −5.4 ($SiCH_3$), −5.4 ($SiCH_3$).

^{19}F NMR (376 MHz, $CDCl_3$) δ −96.74 (dd, J = 248.6, 55.7 Hz, C$F_a$$F_b$H), −99.79 (dd, J = 248.7, 54.8 Hz, C$F_a$$F_b$H).

HRMS-ESI Exact mass calculated for $[M + Na]^+$ ($C_{28}H_{54}F_2N_2O_5SSi_3Na$) requires *m/z* 675.2927, found $[M + Na]^+$ *m/z* 675.2915.

2′,3′,5′-tri-O-TBDMS-4-S-difluoromethyl-4-thio-uridine (23)

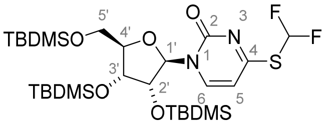

23

To a mixture of DCM (120 µL) and water (120 µL) was added **18** (123 mg, 200 µmol, 1.00 equiv) and KF_2H (94.0 mg, 1.20 mmol, 6.00 equiv) and the mixture was stirred vigorously. $TMSCF_2Br$ (94.0 µL, 600 µmol, 3.00 equiv) was added dropwise and the biphasic solution was stirred for 5 h at 0 °C then diluted with DCM (2 mL) and water (10 mL) and separated. The aqueous phase was extracted with DCM (3 × 10 mL) and the combined organic phase was dried over Na_2SO_4, filtered, and concentrated *in vacuo*. Purification by column chromatography (silica gel, 15% → 50% EtOAc in n-hexane) afforded **23** as a yellow foam (46.0 mg, 35%).

^{1}H NMR (400 MHz, $CDCl_3$) δ 8.52 (d, J = 7.0 Hz, 1H, 6-CH), 7.91 (t, J = 55.1 Hz, 1H 4-C-SCF_2H), 6.09 (d, J = 7.0 Hz, 1H, 5-CH), 5.66 (s, 1H, 1′-CH), 4.18 – 4.09 (m, 3H, 2′-CH, 4′-CH, 5′-C$H_a$$H_b$), 4.01 (dd, J = 8.6, 3.8 Hz, 1H, 3′-CH), 3.80 (dd, J = 11.9, 1.3 Hz, 1H, 5′-C$H_a$$H_b$), 0.94 (s, 9H, Si^{t}Bu), 0.92 (s, 9H, Si^{t}Bu), 0.87 (s, 9H, Si^{t}Bu), 0.28 (s, 3H $SiCH_3$), 0.14 (s, 3H, $SiCH_3$), 0.14 (s, 3H, $SiCH_3$), 0.11 (s, 3H, $SiCH_3$), 0.05 (s, 3H, $SiCH_3$), 0.04 (s, 3H, $SiCH_3$).

^{13}C{^{1}H} NMR (101 MHz, $CDCl_3$) δ 172.7 (t, J = 3.0 Hz, 4-*C*-SCF_2H), 153.1 (2-C=O), 142.8 (6-CH), 109.5 (t, J = 272.0 Hz, SCF_2H), 102.5 (5-CH), 91.6 (1′-CH), 82.9 (4′-CH), 75.1 (2′-CH), 68.4 (3′-CH), 60.4 (5′-CH_2), 26.2 (3 × SiCCH_3), 26.0 (3 × SiCCH_3), 18.7 (SiC), 18.2 (SiC), 18.2 (SiC), −3.8 ($SiCH_3$), −4.0 ($SiCH_3$), −5.0 ($SiCH_3$), −5.0 ($SiCH_3$), −5.1($SiCH_3$), −5.5($SiCH_3$).

^{19}F NMR (376 MHz, $CDCl_3$) δ −98.68 (dd, J = 254.2, 55.2 Hz, C$F_a$$F_b$H), −99.68 (dd, J = 254.0, 55.0 Hz, C$F_a$$F_b$H).

HRMS-ESI Exact mass calculated for $[M + H]^+$ ($C_{28}H_{55}F_2N_2O_5SSi_3$) requires *m/z* 653.3108, found $[M + H]^+$ *m/z* 653.3096.

2′,3′,5′-tri-O-TBDMS-cytidine (24)

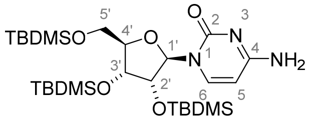

24

TBDMS-Cl (3.09 g, 20.5 mmol, 10.0 equiv) was added to a stirred solution of cytidine (498 mg, 2.05 mmol, 1.00 equiv) and imidazole (558 mg, 8.19 mmol, 4.00 equiv) in anhydrous DMF (20.5 mL) and the mixture stirred at rt for 18 h under an argon atmosphere. Once complete, the reaction mixture was concentrated *in vacuo* and partitioned between EtOAc (30 mL) and water (30 mL) and separated. The organic phase was washed with water (3 × 40 mL) and LiCl (5%, aq., 40 mL), dried over Na_2SO_4, filtered, and concentrated *in vacuo*. Purification by column chromatography (silica gel, 1% → 4% MeOH in DCM) afforded **24** as a colourless foam (787 mg, 67%).

^{1}H NMR (400 MHz, $CDCl_3$) δ 8.09 (d, *J* = 7.5 Hz, 1H, 6-CH), 5.80 (d, *J* = 2.2 Hz, 1H, 1′-CH), 5.74 (d, *J* = 7.5 Hz, 1H, 5-CH), 4.16 – 3.99 (m, 4H, 2′-CH, 3′-CH, 4′-CH, 5′-CH_aH$_b$), 3.87 – 3.67 (m, 1H, 5′-CH$_a$$H_b$), 0.93 (s, 9H, Si^{t}Bu), 0.88 (s, 9H, Si^{t}Bu), 0.88 (s, 9H, Si^{t}Bu), 0.16 (s, 3H, $SiCH_3$), 0.12 (s, 3H, $SiCH_3$), 0.10 (s, 3H, $SiCH_3$), 0.08 (s, 3H, $SiCH_3$), 0.05 (s, 3H, $SiCH_3$), 0.04 (s, 3H, $SiCH_3$).

^{13}C{^{1}H} NMR (101 MHz, $CDCl_3$) δ 165.1 (4-C-NH_2), 155.1 (2-C=O), 141.7 (6-CH), 94.3 (5-CH), 90.1 (1′-CH), 83.4 (4′-CH), 76.4 (2′-CH), 69.8 (3′-CH), 61.3 (5′-CH_2), 26.2 (3 × SiC*C*H_3), 26.0 (3 × SiC*C*H_3), 18.7 (SiC), 18.2 (SiC), 18.2 (SiC), −4.0 ($SiCH_3$), −4.1 ($SiCH_3$), −4.9 ($SiCH_3$), −4.9 ($SiCH_3$), −5.2 ($SiCH_3$), −5.4 ($SiCH_3$).

HRMS-ESI Exact mass calculated for $[M + H]^+$ ($C_{27}H_{56}N_3O_5Si_3$) requires *m/z* 586.3528, found $[M + Na]^+$ *m/z* 586.3516.

3′,5′-di-O-TBDMS-4-N-ethyl-2′-deoxycytidine (25)

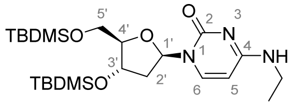

25

TBDMS-Cl (283 mg, 1.88 mmol, 8 equiv) was added to a stirred solution of 4-*N*-ethyl-2′-deoxycytidine (60.0 mg, 0.240 mmol, 1.00 equiv) and imidazole (64.0 mg, 0.940 mmol, 4.00 equiv) in anhydrous DMF (570 μL) and the mixture stirred at rt for 18 h under an argon atmosphere. Once complete, the reaction mixture was concentrated *in vacuo* then partitioned between EtOAc (10 mL) and water (10 mL) and separated. The organic phase was washed with water (3 × 10 mL) and LiCl (5%, aq., 10 mL), dried over Na_2SO_4, filtered, and concentrated *in vacuo* which afforded a colourless foam (105 mg, 92%).

^{1}H NMR (400 MHz, $CDCl_3$) δ 7.89 (d, *J* = 5.0 Hz, 1H, 6-CH), 6.29 (app t, *J* = 5.7 Hz, 1H, 1′-CH), 5.50 (d, *J* = 5.0 Hz, 1H, 5-CH), 4.94 (s, 1H, NH), 4.36 (dd, *J* = 6.0, 5.1 Hz, 1H, 3′-CH), 3.91 (dd, *J* = 11.3, 2.6 Hz, 1H, 5′-CH_aH$_b$), 3.86 (app br s, 1H, 4′-CH), 3.75 (dd, *J* = 11.3, 2.1 Hz, 1H, 5′-CH$_a$$H_b$), 3.52 (app br s, 2H, 4-*N*-CH_2CH$_3$), 2.40 (app dt, *J* = 12.7, 6.3 Hz, 1H, 2′-CH_aH$_b$), 2.08 (ddd, *J* = 13.3, 6.5, 4.9 Hz, 1H, 2′-CH$_a$$H_b$), 1.21 (app br s, 3H, 4-*N*-CH$_2$CH_3), 0.92 (s, 9H, Si^{t}Bu), 0.87 (s, 9H, Si^{t}Bu), 00.10 (s, 3H, $SiCH_3$), 0.09 (s, 3H, $SiCH_3$), 0.04 (s, 3H, $SiCH_3$), 0.04 (s, 3H, $SiCH_3$).

^{13}C NMR (101 MHz, $CDCl_3$) δ 163.7 (4-C-NH_2), 156.3 (2-C=O), 140.1 (6-CH), 94.5 (5-CH), 87.3 (4′-CH), 85.9 (1′-CH), 70.3 (3′-CH), 62.0 (5′-CH_2), 42.3 (2′-CH_2), 35.9 (4-*N*-CH_2CH$_3$), 26.05 (Si^{t}Bu), 25.9 (Si^{t}Bu), 18.5 (SiC), 18.1 (SiC), 14.8 (4-*N*-CH$_2$CH_3), -4.4 ($SiCH_3$), -4.8 ($SiCH_3$), -5.3 ($SiCH_3$), -5.4 ($SiCH_3$).

HRMS-ESI Exact mass calculated for $[M + H]^+$ ($C_{23}H_{46}N_3O_4Si_2$) requires *m/z* 484.3027, found $[M + H]^+$ *m/z* 484.3013.

3′,5′-di-O-TBDMS-4-N-Ac-cytidine (26)

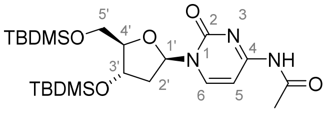

26

TBDMS-Cl (299 mg, 1.98 mmol, 9.00 equiv) was added to a stirred solution of deoxycytidine (100 mg, 440 µmol, 1.00 equiv) and imidazole (120 mg, 1.76 mmol, 4.00 equiv) in anhydrous DMF (1.06 mL) and the mixture stirred at rt for 18 h under an argon atmosphere. Once complete, the reaction mixture was concentrated *in vacuo* then partitioned between EtOAc (10 mL) and water (10 mL) and separated. The organic phase was washed with water (3 × 10 mL) and LiCl (5%, aq., 10 mL), dried over Na_2SO_4, filtered, and concentrated *in vacuo* to afford a colourless solid foam. This solid (198 mg) was dissolved in anhydrous pyridine (2.13 mL) and DMAP (4.48 mg, 43.9 µmol, 10.0 mol%) and acetic anhydride (49.8 µL, 527 µmol, 1.20 equiv) were added sequentially at 0 °C under an argon atmosphere. The suspension was allowed to stir vigorously overnight at rt then water (1 mL) was added to quench the reaction and then the mixture was concentrated *in vacuo*. The crude mixture was partitioned between DCM (5 mL) and $NaHCO_3$ (sat. aq., 5 mL) and separated. The organic phase was washed with water (3 × 4 mL), dried over Na_2SO_4, filtered, and concentrated *in vacuo,* affording **26** a colourless foam.

^{1}H NMR (400 MHz, $CDCl_3$) δ 9.51 (s, 1H, NH), 8.36 (d, *J* = 7.5 Hz, 1H, 6-CH), 7.36 (d, *J* = 7.5 Hz, 1H, 5-CH), 6.21 (dd, *J* = 6.5, 4.8 Hz, 1H, 1′-CH), 4.37 (td, *J* = 6.0, 4.6 Hz, 1H, 3′-CH), 3.98 – 3.90 (m, 2H 4′-CH, 5′-CH_aH$_b$), 3.77 (dd, *J* = 12.1, 2.8 Hz, 1H, 5′-CH$_a$$H_b$), 2.49 (ddd, *J* = 13.4, 6.5, 5.8 Hz, 1H, 2′-CH_aH$_b$), 2.25 (s, 3H, CH_3CO), 2.15 – 2.04 (m, 1H, 2′-CH$_a$$H_b$), 0.92 (s, 9H, Si^{t}Bu), 0.87 (s, 9H, Si^{t}Bu), 0.11 (s, 3H, $SiCH_3$), 0.10 (s, 3H, $SiCH_3$), 0.04 (s, 6H, 2 × $SiCH_3$).

^{13}C{^{1}H} NMR (101 MHz, $CDCl_3$) δ 170.8 (CH_3*C*=O), 162.6 (4-C-NHAc), 155.2 (2-C=O), 144.8 (6-CH), 96.3 (5-CH), 87.9 (4′-CH), 86.9 (1′-CH), 70.1 (3′-CH), 61.9 (5′-CH_2), 42.4 (2′-CH_2), 26.0 (3 ×

Si^{t}Bu), 25.8 (3 × Si^{t}Bu), 25.0 (*CH3*CO), 18.5 (SiC), 18.1 (SiC), −4.5 ($SiCH_3$), −4.8 ($SiCH_3$), −5.4 ($SiCH_3$), −5.4 ($SiCH_3$).

These data are in accordance with literature values.[6]

2',3',5'-tri-O-TBDMS-4-N-Ac-cytidine (27)

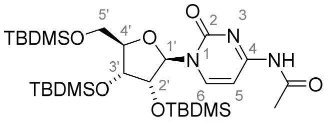

27

To a solution of **24** (290 mg, 495 µmol, 1.00 equiv) and DMAP (5.10 mg, 50.0 µmol, 10.0 mol%) in anhydrous pyridine (2.40 mL) was added acetic anhydride (56.1 µL, 594 µmol, 1.20 equiv) dropwise at 0 °C under an argon atmosphere. This suspension was allowed to stir vigorously overnight at room temperature then water (2 mL) was added to quench the reaction and then the mixture was concentrated *in vacuo*. Crude mixture was partitioned between DCM (10 mL) and $NaHCO_3$ (sat. aq., 10 mL) and separated. The organic phase was washed with water (3 × 8 mL), dried over Na_2SO_4, filtered, and concentrated *in vacuo*. Purification by column chromatography (silica gel, 1% → 3% MeOH in DCM) afforded **27** as a colourless foam (276 mg, 89%).

^{1}H NMR (400 MHz, $CDCl_3$) δ 10.55 (s, 1H, NH), 8.53 (d, *J* = 7.5 Hz, 1H, 6-CH), 7.40 (d, *J* = 7.5 Hz, 1H, 5-CH), 5.76 (d, *J* = 1.4 Hz, 1H, 1′-CH), 4.17 – 4.00 (m, 4H, 2′-CH, 3′-CH, 4′-CH, 5′-CH_aH$_b$), 3.82 – 3.72 (m, 1H, 5′-CH$_a$$H_b$), 2.30 (s, 3H, *CH3*CO), 0.96 (s, 9H, Si^{t}Bu), 0.90 (s, 9H, Si^{t}Bu), 0.87 (s, 9H, Si^{t}Bu), 0.21 (s, 3H, $SiCH_3$), 0.14 (s, 3H, $SiCH_3$), 0.13 (s, 3H, $SiCH_3$), 0.09 (s, 3H, $SiCH_3$), 0.04 (s, 3H, $SiCH_3$), 0.03 (s, 3H, $SiCH_3$).

^{13}C{^{1}H} NMR (101 MHz, $CDCl_3$) δ 171.8 (CH$_3$*C*=O), 163.3 (4-C-NHAc), 155.2 (2-C=O), 145.1 (6-CH), 96.5 (5-CH), 91.0 (1′-CH), 83.1 (4′-CH), 76.4 (2′-CH), 69.0 (3′-CH), 60.7 (5′-CH_2), 26.3 (3 × SiC*C*H$_3$), 25.9 (6 × SiC*C*H$_3$), 25.0 (*CH3*CO 18.7 (SiC), 18.2 (2 × SiC), −3.9 ($SiCH_3$), −4.0 ($SiCH_3$), −4.9 ($SiCH_3$), −5.0 ($SiCH_3$), −5.1 ($SiCH_3$), −5.4 ($SiCH_3$).

HRMS-ESI Exact mass calculated for $[M + H]^+$ ($C_{29}H_{58}N_3O_6Si_3$) requires *m/z* 628.3633, found $[M + Na]^+$ *m/z* 628.3624.

3',5'-di-O-TBDMS-5-methyl-2'-deoxycytidine (28)

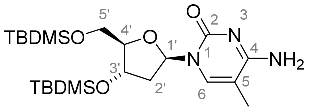

28

TBDMS-Cl (288 mg 1.91 mmol, 4.50 equiv) was added to a stirred solution of 5-methyl-2′-deoxy-cytidine (102 mg, 433 µmol, 1 equiv) and imidazole (116 mg, 1.70 mmol, 4.00 equiv) in anhydrous DMF (1.03 mL) and the mixture stirred at rt for 18 h under an argon atmosphere. Once complete, the

reaction mixture was concentrated *in vacuo* then partitioned between EtOAc (10 mL) and water (10 mL) and separated. The organic phase was washed with water (3 × 10 mL) and LiCl (5%, aq., 10 mL), dried over Na_2SO_4, filtered, and concentrated *in vacuo*. Purification by column chromatography (silica gel, 1% → 4% MeOH in DCM) afforded **28** as a colourless foam (156 mg, 78%).

^{1}H NMR (400 MHz, $CDCl_3$) δ 7.57 – 7.49 (m, 1H, 4-NH), 7.26 (d, *J* = 1.0 Hz, 1H, 6-CH), 6.29 (app t, *J* = 6.5 Hz, 1H, 1′-CH), 4.34 (app dt, *J* = 6.3, 3.2 Hz, 1H, 3′-CH), 3.91 (dd, *J* = 2.9, 2.9 Hz, 1H, 4′-CH), 3.86 (dd, *J* = 11.3, 2.8 Hz, 1H, 5′-CH_aH$_b$), 3.74 (dd, *J* = 11.2, 2.7 Hz, 1H, 5′-CH$_a$$H_b$), 2.36 (ddd, *J* = 13.3, 6.1, 3.4 Hz, 1H, 2′-CH_aH$_b$), 2.00 – 1.87 (m, 4H, 5-Me, 2′-CH$_a$$H_b$), 0.90 (s, 9H, Si^{t}Bu), 0.87 (s, 9H, Si^{t}Bu), 0.09 (s, 3H, $SiCH_3$), 0.09 (s, 3H, $SiCH_3$), 0.05 (s, 3H, $SiCH_3$), 0.05 (s, 3H, $SiCH_3$).

^{13}C{^{1}H} NMR (101 MHz, $CDCl_3$) δ 165.6 (4-C-NH_2), 155.9 (2-C=O), 138.0 (6-CH), 101.6 (5-C), 87.7 (4′-CH), 86.0 (1′-CH), 72.0 (3′-CH), 62.9 (5′-CH_2), 42.3 (2′-CH_2), 26.0 (3 × Si*C*CH_3), 25.9 (3 × Si*C*CH_3), 18.5 (Si*C*CH_3), 18.1 (Si*C*CH_3), −4.5 ($SiCH_3$), −4.8 ($SiCH_3$), −5.2 ($SiCH_3$), −5.3 ($SiCH_3$). These data are in accordance with literature values.[7]

3′,5′-di-O-TBDMS-5-bromo-2′-deoxycytidine (29)

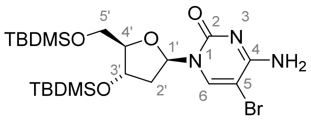

29

TBDMS-Cl (407 mg, 2.70 mmol, 4.50 equiv) was added to a stirred solution of 5-bromo-deoxycytidine (184 mg, 600 μmol, 1.00 equiv) and imidazole (163 mg, 2.40 mmol, 4.00 equiv) in anhydrous DMF (1.45 mL) and the mixture stirred at rt for 18 h under an argon atmosphere. Once complete, the reaction mixture was concentrated *in vacuo* then partitioned between EtOAc (10 mL) and water (10 mL) and separated. The organic phase was washed with water (3 × 10 mL) and LiCl (5%, aq., 10 mL), dried over Na_2SO_4, filtered, and concentrated *in vacuo*. Purification by column chromatography (silica gel, 1% → 4% MeOH in DCM) afforded **29** as a colourless foam (273 mg, 85%).

^{1}H NMR (400 MHz, $CDCl_3$) δ 8.71 (br s, 1H, 4-NH_aH$_b$), 8.02 (d, *J* = 1.9 Hz, 1H, 6-CH), 6.22 (app t, *J* = 6.3 Hz, 1′-CH), 5.68 (s, 1H, 4-NH$_a$$H_b$), 4.34 (app dt, *J* = 6.4, 3.3 Hz, 1H, 3′-CH), 3.95 (app d, *J* = 2.8 Hz, 1H, 4′-CH), 3.92 – 3.86 (m, 1H, 5′-CH_aH$_b$), 3.74 (app dt, *J* = 11.4, 1.6 Hz, 1H, 5′-CH$_a$$H_b$), 2.43 (dddd, *J* = 13.4, 5.6, 3.6, 1.6 Hz, 1H, 2′-CH_aH$_b$), 2.02 – 1.93 (m, 1H, 2′-CH$_a$$H_b$), 0.91 (s, 9H, Si^{t}Bu), 0.87 (s, 9H, Si^{t}Bu), 0.12 (s, 3H, $SiCH_3$), 0.11 (s, 3H, $SiCH_3$), 0.05 (s, 3H, $SiCH_3$), 0.05 (s, 3H, $SiCH_3$).

^{13}C{^{1}H} NMR (101 MHz, $CDCl_3$) δ 162.5 (4-C-NH_2), 154.6 (2-C=O), 141.3 (6-CH), 88.2 (4′-CH), 87.4 (5-CBr), 86.8 (1′-CH), 72.0 (3′-CH), 62.8 (5′-CH_2), 42.7 (2′-CH_2), 26.2 (SiC*C*H_3), 26.2 (SiC*C*H_3), 26.2 (SiC*C*H_3), 25.8 (SiC*C*H_3), 25.8 (SiC*C*H_3), 25.8 (SiC*C*H_3), 18.5 (SiC), 18.1 (SiC), −4.5 ($SiCH_3$), −4.8 ($SiCH_3$), −5.2 ($SiCH_3$), −5.2 ($SiCH_3$).

HRMS-ESI Exact mass calculated for $[2M + H]^+$ ($C_{42}H_{81}{}^{79}Br{}^{81}BrN_6O_8Si_4$) requires *m/z* 1069.3539, found $[2M + H]^+$ *m/z* 1069.3545.

3′,5′-di-O-TBDMS-5-fluoro-2′-deoxycytidine (30)

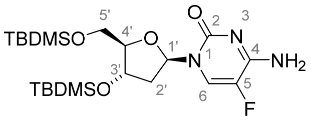

30

TBDMS-Cl (553 mg, 3.67 mmol, 4.50 equiv) was added to a stirred solution of 5-fluoro-deoxycytidine (200 mg, 820 μmol, 1.00 equiv) and imidazole (222 mg, 3.26 mmol, 4.00 equiv) in anhydrous DMF (570 μL) and the mixture stirred at rt for 18 h under an argon atmosphere. Once complete, the reaction mixture was concentrated *in vacuo* then partitioned between EtOAc (10 mL) and water (10 mL) and separated. The organic phase was washed with water (3 × 10 mL) and LiCl (5%, aq., 10 mL), dried over Na_2SO_4, filtered, and concentrated *in vacuo*. Purification by column chromatography (silica gel, 1% → 4% MeOH in DCM) afforded **30** as a colourless foam (350 mg, 91%).

^{1}H NMR (400 MHz, $CDCl_3$) δ 8.74 (s, 1H, 4-N*H$_a$*H$_b$), 8.00 (d, *J* = 6.5 Hz, 1H, 6-CH), 6.18 (ddd, *J* = 6.3, 5.9, 1.8 Hz, 1H, 1′-CH), 5.70 (s, 1H, 4-NH$_a$*H$_b$*), 4.34 (dd, *J* = 6.2, 4.8 Hz, 1H, 3′-CH), 3.91 (dd, *J* = 11.2, 2.5 Hz, 1H, 5′-C*H$_a$*H$_b$), 3.87 (ddd, *J* = 4.5, 2.2 Hz, 1H, 4′-CH), 3.74 (dd, *J* = 11.3, 1.9 Hz, 1H, 5′-CH$_a$*H$_b$*), 2.37 (ddd, *J* = 13.3, 6.3, 5.2 Hz, 1H, 2′-C*H$_a$*H$_b$), 2.03 (app dt, *J* = 13.5, 5.9 Hz, 1H, 2′-CH$_a$*H$_b$*), 0.90 (s, 9H, Si^{t}Bu), 0.85 (s, 9H, Si^{t}Bu), 0.09 (s, 3H, $SiCH_3$), 0.08 (s, 3H, $SiCH_3$), 0.03 (s, 3H, $SiCH_3$), 0.03 (s, 3H, $SiCH_3$).

^{13}C{^{1}H} NMR (101 MHz, $CDCl_3$) δ 157.7 (d, *J* = 14.2 Hz, 4-C-NH$_2$), 153.5 (2-C=O), 136.0 (d, *J* = 240.5 Hz, 5-C-F), 124.6 (d, *J* = 32.2 Hz, 6-CH), 87.0 (4′-CH), 85.5 (1′-CH), 70.0 (3′-CH), 61.6 (5′-CH$_2$), 41.7 (2′-CH$_2$), 25.4 (SiC*C*H$_3$), 25.4 (SiC*C*H$_3$), 25.4 (SiC*C*H$_3$), 25.2 (SiC*C*H$_3$), 25.2 (SiC*C*H$_3$), 25.2 (SiC*C*H$_3$), 17.9 (SiC), 17.4 (SiC), −5.1 ($SiCH_3$), −5.4 ($SiCH_3$), −6.1 ($SiCH_3$), −6.1 ($SiCH_3$).

^{19}F NMR (376 MHz, $CDCl_3$) δ −168.61 (dd, *J* = 6.7, 1.8 Hz, 5-F).

HRMS-ESI Exact mass calculated for $[2M + H]^+$ ($C_{42}H_{81}F_2N_6O_8Si_4$) requires *m/z* 947.5161, found $[2M + H]^+$ *m/z* 947.5157.

3′,5′-di-O-TBDMS-2-thio-2′-deoxycytidine (31)

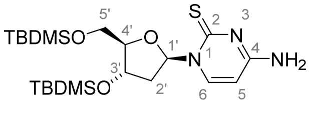

31

TBDMS-Cl (283 mg, 1.88 mmol, 8.00 equiv) was added to a stirred solution of 2-thio-deoxycytidine (57.2 mg, 240 μmol, 1.00 equiv) and imidazole (64.0 mg, 940 μmol, 4.00 equiv) in anhydrous DMF (570 μL) and the mixture stirred at rt for 18 h under an argon atmosphere. Once complete, the reaction mixture was concentrated *in vacuo* then partitioned between EtOAc (10 mL) and water (10 mL) and separated. The organic phase was washed with water (3 × 10 mL) and LiCl (5%, aq., 10 mL), dried over

Na_2SO_4, filtered, and concentrated *in vacuo*. Purification by column chromatography (silica gel, 1% → 4% MeOH in DCM) afforded **31** as a colourless foam (90.0 mg, 81%).

^{1}H NMR (400 MHz, $CDCl_3$) δ 8.89 (s, 1H, 4-NH_aH$_b$), 8.19 (d, *J* = 7.5 Hz, 1H, 6-CH), 6.87 (dd, *J* = 6.8, 4.0 Hz, 1H, 1′-CH), 5.98 (d, *J* = 7.5 Hz, 1H, 5-CH), 5.84 (s, 1H, 4-NH$_a$$H_b$), 4.33 (dd, *J* = 6.7, 5.5 Hz, 1H, 3′-CH), 3.95 (dd, *J* = 11.5, 2.4 Hz, 1H, 5′-CH_aH$_b$), 3.87 (app dt, *J* = 5.8, 2.2 Hz, 1H, 4′-CH), 3.76 (dd, *J* = 11.5, 1.9 Hz, 1H, 5′-CH$_a$$H_b$), 2.57 (ddd, *J* = 13.5, 6.7 Hz, 1H, 2′-CH_aH$_b$), 2.17 (ddd, *J* = 13.6, 6.7, 4.0 Hz, 1H, 2′-CH$_a$$H_b$), 0.90 (s, 9H, $Si^{t}Bu$), 0.85 (s, 9H, $Si^{t}Bu$), 0.09 (s, 3H, $SiCH_3$), 0.08 (s, 3H, $SiCH_3$), 0.04 (s, 3H, $SiCH_3$), 0.03 (s, 3H, $SiCH_3$).

^{13}C{^{1}H} NMR (101 MHz, $CDCl_3$) δ 179.6 (2-C=S), 160.4 (4-C-NH_2), 141.5 (6-CH), 98.3 (5-CH), 90.0 (1′-CH), 87.5 (4′-CH), 69.3 (3′-CH), 61.4 (5′-CH_2), 42.1 (2′-CH_2), 26.0 (SiC*C*H_3), 26.0 (SiC*C*H_3), 26.0 (SiC*C*H_3), 25.8 (SiC*C*H_3), 25.8 (SiC*C*H_3), 25.8 (SiC*C*H_3), 18.4 (SiC), 18.0 (SiC), −4.4 ($SiCH_3$), −4.9 ($SiCH_3$), −5.4 ($SiCH_3$), −5.4 ($SiCH_3$).

HRMS-ESI Exact mass calculated for $[M + H]^+$ ($C_{21}H_{42}N_3O_3SSi_2$) requires *m/z* 472.2485, found $[M + H]^+$ *m/z* 472.2472.

2′,3′,5′-tri-O-TBDMS-2-O-difluoromethyl-cytidine (32)

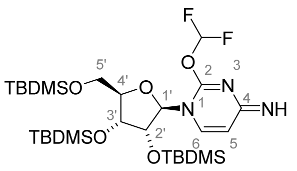

32

To a mixture of DCM (300 μL) and water (300 μL) was added **24** (293 mg, 500 μmol, 1.00 equiv) and KF_2H (94.0 mg, 3.00 mmol, 6.00 equiv) and the mixture was stirred vigorously. $TMSCF_2Br$ (230 μL, 1.50 mmol, 3.00 equiv) was added dropwise and the biphasic solution was stirred for 5 h at 0 °C then diluted with DCM (2 mL) and water (10 mL) and separated. The aqueous phase was extracted with DCM (3 × 10 mL) and the combined organic phase was dried over Na_2SO_4, filtered, and concentrated *in vacuo*. Purification by column chromatography (silica gel, 15% → 50% EtOAc in n-hexane) afforded **32** as a yellow foam (64.0 mg, 20%).

^{1}H NMR (400 MHz, $CDCl_3$) δ 7.77 (t, *J* = 58.0 Hz, 1H, 2-OCF_2H), 7.39 (d, *J* = 8.3 Hz, 1H, 6-CH), 5.95 – 5.82 (m, 1H, 1′-CH), 5.58 (d, *J* = 8.3 Hz, 1H, 5-CH), 4.07 – 4.00 (m, 3H, 3′-CH, 4′-CH, 2′-CH), 3.93 (dd, *J* = 11.6, 2.0 Hz, 1H, 5′-CH_aH$_b$), 3.73 (dd, *J* = 11.6, 1.4 Hz, 1H, 5′-CH$_a$$H_b$), 0.94 (s, 9H, $Si^{t}Bu$), 0.91 (s, 9H, $Si^{t}Bu$), 0.88 (s, 9H, $Si^{t}Bu$), 0.12 (s, 3H, $SiCH_3$), 0.11 (s, 3H, $SiCH_3$), 0.09 (s, 3H, $SiCH_3$), 0.08 (s, 3H, $SiCH_3$), 0.06 (s, 3H, $SiCH_3$), 0.05 (s, 3H, $SiCH_3$).

^{13}C{^{1}H} NMR (101 MHz, $CDCl_3$) δ 153.3 (4-C=NH), 148.7 (2-OCF_2H), 132.2 (6-CH), 109.8 (t, *J* = 250.9 Hz, 2-OCF2H), 103.3 (5-CH), 88.5 (1′-CH), 85.1 (4′-CH), 75.8 (2′-CH), 71.7 (3′-CH), 62.5 (5′-CH), 26.2 (3 × SiC*C*H_3), 26.0 (3 × SiC*C*H_3), 25.9 (3 × SiC*C*H_3), 18.6 (SiC), 18.2 (SiC), 18.1 (SiC), −4.2 ($SiCH_3$), −4.5 ($SiCH_3$), −4.5 ($SiCH_3$), −4.6 ($SiCH_3$), −5.3 ($SiCH_3$), −5.4 ($SiCH_3$).

^{19}**F NMR** (376 MHz, $CDCl_3$) δ −108.56 (dd, *J* = 233.3, 57.7 Hz, C*F*$_a$F$_b$H), −109.70 (dd, *J* = 233.3, 57.5 Hz, CF$_a$*F*$_b$H).

HRMS-ESI Exact mass calculated for $[M + H]^+$ ($C_{28}H_{56}F_2N_3O_5Si_3$) requires *m/z* 636.3496, found $[M + H]^+$ *m/z* 636.3485.

3′,5′-di-O-TBDMS-2-O-difluoromethyl-4-N-ethyl-2′-deoxycytidine (33)

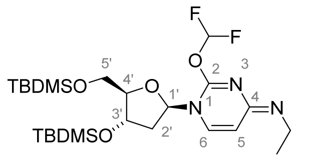

33

To a mixture of DCM (180 µL) and water (120 µL) was added **25** (96.8 mg 200 µmol, 1.00 equiv.) and KF_2H (94.0 mg, 1.20 mmol, 6.00 equiv) and the mixture was stirred vigorously. $TMSCF_2Br$ (94.0 µL, 600 µmol, 3.00 equiv.) was added dropwise and the biphasic solution was stirred for 5 h at 0 °C then diluted with DCM (2 mL) and water (10 mL) and separated. The aqueous phase was extracted with DCM (3 × 10 mL) and the combined organic phase was dried over Na_2SO_4, filtered, and concentrated *in vacuo*. Purification by column chromatography (silica gel, 5% → 40% EtOAc in n-hexane) afforded **33** as a colourless oil (53.0 mg, 49%).

^{1}**H NMR** (400 MHz, $CDCl_3$) δ 7.85 (t, *J* = 58.4 Hz, 1H, 2-OCF_2H), 7.28 (d, *J* = 8.6 Hz, 1H, 6-CH), 6.28 (dd, *J* = 6.5 Hz, 1H, 1′-CH), 5.68 (d, *J* = 8.6 Hz, 1H, 5-CH), 4.38 (app dt, *J* = 6.4, 3.5 Hz, 1H, 3′-CH), 3.88 (app q, *J* = 2.7 Hz, 1H, 4′-CH), 3.84 (dd, *J* = 11.3, 2.8 Hz, 1H, 5′-C*H*$_a$H$_b$), 3.73 (dd, *J* = 11.3, 2.2 Hz, 1H, 5′-CH$_a$*H*$_b$), 3.28 (q, *J* = 7.3 Hz, 1H, 4-N-C*H*$_2$CH$_3$), 2.24 (ddd, *J* = 13.2, 6.1, 3.7 Hz, 1H, 2′-C*H*$_a$H$_b$), 2.00 (ddd, *J* = 13.1, 7.0, 6.1 Hz, 1H, 2′-CH$_a$*H*$_b$), 1.17 (t, *J* = 7.3 Hz, 3H, 4-N-C*H*$_2$CH$_3$), 0.92 (s, 9H, Si^{t}Bu), 0.88 (s, 9H, Si^{t}Bu), 0.10 (s, 3H, $SiCH_3$), 0.10 (s, 3H, $SiCH_3$), 0.07 (s, 3H, $SiCH_3$), 0.06 (s, 3H, $SiCH_3$).

^{13}**C{^{1}H} NMR** (101 MHz, $CDCl_3$) δ 148.4 (2-*C*-OCF_2H), 147.7 (4-C=NEt), 132.6 (6-CH), 109.0 (t, *J* = 251.7, 2-OCF_2H), 94.0 (5-CH), 87.6 (4′-CH), 84.8 (1′-CH), 71.7 (3′-CH), 62.8 (5′-CH_2), 43.0 (4-N-*CH*$_2$CH$_3$), 41.3 (2′-CH_2), 26.0 (3 × SiC*C*H$_3$), 25.8 (3 × SiC*C*H$_3$), 18.5 (SiC), 18.1 (SiC), 16.2 (4-N-CH$_2$*CH*$_3$), −4.5 ($SiCH_3$), −4.7 ($SiCH_3$), −5.3 ($SiCH_3$), −5.4 ($SiCH_3$).

^{19}**F NMR** (376 MHz, $CDCl_3$) δ −108.97 (dd, *J* = 228.4, 58.4 Hz, C*F*$_a$F$_b$H), −109.83 (dd, *J* = 228.4, 58.5 Hz, CF$_a$*F*$_b$H).

HRMS-ESI Exact mass calculated for $[M + H]^+$ ($C_{24}H_{46}F_2N_3O_4Si_2$) requires *m/z* 534.2995, found $[M + H]^+$ *m/z* 534.2981.

3′,5′-tri-O-TBDMS-4-N-Ac-2-O-difluoromethyl-2′-deoxycytidine (34)

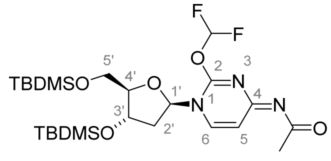

34

To a mixture of DCM (180 µL) and water (120 µL) was added **26** (200 µmol, 1.00 equiv) and KF_2H (94 mg, 1.20 mmol, 6.0 equiv) and the mixture was stirred vigorously. $TMSCF_2Br$ (94.0 µL, 600 µmol, 3.00 equiv) was added dropwise and the biphasic solution was stirred for 5 h at 0 °C then diluted with DCM (2 mL) and water (10 mL) and separated. The aqueous phase was extracted with DCM (3 × 10 mL) and the combined organic phase was dried over Na_2SO_4, filtered, and concentrated *in vacuo*. Purification by column chromatography (silica gel, 5% → 20% EtOAc in n-hexane) afforded **34** as a yellow foam (54.0 mg, 61%).

^{1}H NMR (400 MHz, $CDCl_3$) δ 7.76 (t, *J* = 57.7 Hz, 1H, 2-OCF_2H), 7.63 (d, *J* = 8.3 Hz, 1H, 6-CH), 6.25 (app t, *J* = 6.2 Hz, 1H, 1′-CH), 6.04 (d, *J* = 8.3 Hz, 1H, 5-CH), 4.38 (app dt, *J* = 6.1, 3.8 Hz, 1H, 3′-CH), 3.95 – 3.90 (m, 1H,), 3.87 (dd, *J* = 11.4, 2.6 Hz, 1H, 5′-CH_aH_b), 3.79 – 3.70 (m, 1H, 5′-CH_aH_b), 2.32 (ddd, *J* = 13.3, 6.1, 4.1 Hz, 1H, 2′-CH_aH_b), 2.22 (s, 3H, CH_3CO), 2.02 (app dt, *J* = 13.4, 6.2 Hz, 1H, 2′-CH_aH_b), 0.91 (s, 9H, Si^{t}Bu), 0.88 (s, 9H, Si^{t}Bu), 0.09 (s, 3H, $SiCH_3$), 0.09 (s, 3H, $SiCH_3$), 0.07 (s, 3H, $SiCH_3$), 0.07 (s, 3H, $SiCH_3$).

^{13}C NMR (101 MHz, $CDCl_3$) δ 183.5 (CH_3C=O), 150.5 (4-C=NEt), 147.3 (2-*C*-OCF_2H), 137.0 (6-CH), 108.7 (t, *J* = 255.0 Hz), 97.0 (5-CH), 88.0 (4′-CH), 85.7 (1′-CH), 71.3 (3′-CH), 62.5 (5′-CH_2), 41.9 (2′-CH_2), 27.1 (CH_3CO), 26.0 (2 × $SiCCH_3$), 25.8 (2 × $SiCCH_3$), 18.5 (SiC), 18.1 (SiC), −4.5 ($SiCH_3$), −4.7 ($SiCH_3$), −5.3 ($SiCH_3$), −5.4 ($SiCH_3$).

^{19}F NMR (376 MHz, $CDCl_3$) δ −109.15 (dd, *J* = 230.3, 57.6 Hz, CF_aF_bH), −109.85 (dd, *J* = 230.3, 57.6 Hz, CF_aF_bH).

HRMS-ESI Exact mass calculated for $[M + Na]^+$ ($C_{24}H_{43}F_2N_3O_5Si_2Na$) requires *m/z* 570.2607, found $[M + Na]^+$ *m/z* 570.2595.

2′,3′,5′-tri-O-TBDMS-4-N-Ac-2-O-difluoromethyl-cytidine (35)

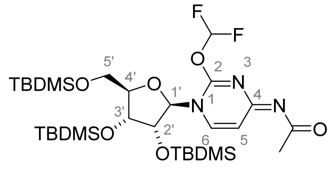

35

To a mixture of DCM (100 µL) and water (100 µL) was added **27** (100 mg, 160 µmol, 1.00 equiv) and KF_2H (74.0 mg, 960 µmol, 6.00 equiv) and the mixture was stirred vigorously. $TMSCF_2Br$ (74.3 µL, 480 µmol, 3.00 equiv) was added dropwise and the biphasic solution was stirred for 5 h at 0 °C then

diluted with DCM (2 mL) and water (8 mL) and separated. The aqueous phase was extracted with DCM (3 × 8 mL) and the combined organic phase was dried over Na_2SO_4, filtered, and concentrated *in vacuo*. Purification by column chromatography (silica gel, 5% → 20% EtOAc in n-hexane) afforded **35** as a yellow foam (54.0 mg, 51%).

^{1}H NMR (400 MHz, $CDCl_3$) δ 7.74 (t, *J* = 57.6 Hz, 1H, 2-OCF_2H), 7.71 (d, *J* = 8.3 Hz, 1H, 6-CH), 6.02 (d, *J* = 8.3 Hz, 1H, 5-CH), 5.89 (d, *J* = 3.8 Hz, 1H, 1′-CH), 4.06 – 4.01 (m, 3H, 4′-CH, 3′-CH, 2′-CH), 3.94 (dd, *J* = 11.8, 2.0 Hz, 1H, 5′-CH_aH_b), 3.73 (dd, *J* = 11.8, 1.3 Hz, 1H, 5′-CH_aH_b), 2.22 (s, 3H, CH_3CO), 0.93 (s, 9H, Si^{t}Bu), 0.90 (s, 9H, Si^{t}Bu), 0.88 (s, 9H, Si^{t}Bu), 0.11 (s, 3H, $SiCH_3$), 0.10 (s, 3H, $SiCH_3$), 0.09 (s, 3H, $SiCH_3$), 0.07 (s, 3H, $SiCH_3$), 0.07 (s, 3H, $SiCH_3$), 0.06 (s, 3H, $SiCH_3$).

^{13}C{^{1}H} NMR (101 MHz, $CDCl_3$) δ 183.4 (CH_3*C*=O), 150.1 (4-C=NAc), 147.4 (2-*C*-OCF_2H), 137.1 (6-CH), 108.7 (t, *J* = 255.0 Hz, OCF_2H), 96.7 (5-CH), 88.9 (1′-CH), 85.0 (4′-CH), 76.1 (2′-CH), 71.2 (3′-CH), 62.1 (5′-CH_2), 27.1 (*CH_3*CO), 26.2 (3 × SiC*C*H_3), 25.9 (6 × SiC*C*H_3),18.6 (SiC), 18.2 (SiC), 18.1 (SiC), −4.1 ($SiCH_3$), −4.4 ($SiCH_3$), −4.6 ($SiCH_3$), −4.7 ($SiCH_3$), −5.2 ($SiCH_3$), −5.3 ($SiCH_3$).

^{19}F NMR (376 MHz, $CDCl_3$) δ −108.93 (dd, *J* = 230.2, 57.7 Hz, CF_aF_bH), −110.11 (dd, *J* = 230.1, 57.5 Hz, CF_aF_bH).

HRMS-ESI Exact mass calculated for $[M - Ac + 2H]^+$ ($C_{28}H_{56}F_2N_3O_5Si_3$) requires *m/z* 636.3496, found $[M - Ac + 2H]^+$ *m/z* 636.3485.

3′,5′-di-O-TBDMS-2-O-difluoromethyl-5-methyl-2′-deoxycytidine (36)

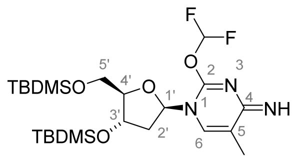

36

To a mixture of DCM (180 µL) and water (120 µL) was added **28** (94.0 mg 200 µmol, 1.00 equiv) and KF_2H (94.0 mg, 1.20 mmol, 6.00 equiv) and the mixture was stirred vigorously. $TMSCF_2Br$ (94.0 µL, 600 µmol, 3.00 equiv) was added dropwise and the biphasic solution was stirred for 5 h at 0 °C then diluted with DCM (2 mL) and water (10 mL) and separated. The aqueous phase was extracted with DCM (3 × 10 mL) and the combined organic phase was dried over Na_2SO_4, filtered, and concentrated *in vacuo*. Purification by column chromatography (silica gel, 5% → 40% EtOAc in n-hexane) afforded **36** as a colourless foam (19.0 mg, 18%).

^{1}H NMR (400 MHz, $CDCl_3$) δ 7.60 (t, *J* = 57.7 Hz, 1H, 2-OCF_2H), 7.52 (d, *J* = 1.3 Hz, 1H, 6-CH), 6.31 (dd, *J* = 7.9, 5.7 Hz, 1H, 1′-CH), 4.39 (app dt, *J* = 5.4, 2.4 Hz, 1H, 3′-CH), 3.96 (app q, *J* = 2.5 Hz, 1H, 4′-CH), 3.87 (dd, *J* = 11.4, 2.5 Hz, 1H, 5′-CH_aH_b), 3.76 (dd, *J* = 11.4, 2.4 Hz, 1H, 5′-CH_aH_b), 2.29 (ddd, *J* = 13.1, 5.7, 2.5 Hz, 1H, 2′-CH_aH_b), 2.04 – 1.94 (m, 1H, 2′-CH_aH_b), 1.92 (d, *J* = 1.2 Hz, 3H, 5-C-CH_3), 0.93 (s, 9H, Si-^{t}Bu), 0.89 (s, 9H, Si-^{t}Bu), 0.11 (s, 3H, $SiCH_3$), 0.11 (s, 3H, $SiCH_3$), 0.08 (s, 3H, $SiCH_3$), 0.07 (s, 3H, $SiCH_3$).

$^{13}C\{^1H\}$ NMR (101 MHz, $CDCl_3$) δ 161.7 (4-C=N), 148.4 (2-*C*-OCF_2H), 135.4 (6-CH), 110.1 (5-C), 108.8 (t, *J* = 253.4 Hz, OCF_2H), 88.3 (4′-CH), 85.7 (1′-CH), 72.4 (3′-CH), 63.1 (5′-CH_2), 41.7 (2′-CH_2), 26.1 (3 × SiC*C*H_3), 25.9 (3 × SiC*C*H_3), 18.5 (SiC), 18.1 (SiC), 13.0 (5-CH_3), −4.5 ($SiCH_3$), −4.7 ($SiCH_3$), −5.2 ($SiCH_3$), −5.3 ($SiCH_3$).

^{19}F NMR (376 MHz, $CDCl_3$) δ −108.73 (dd, *J* = 233.3, 42.8 Hz, CF_aF_bH), −109.47 (dd, *J* = 233.3, 42.8 Hz, CF_aF_bH).

HRMS-ESI Exact mass calculated for $[M + H]^+$ ($C_{23}H_{43}F_2N_3O_4Si_2Na$) requires *m/z* 542.2658, found $[M + H]^+$ *m/z* 542.2646.

3′,5′-di-O-TBDMS-2-O-difluoromethyl-5-bromo-2′-deoxycytidine (37)

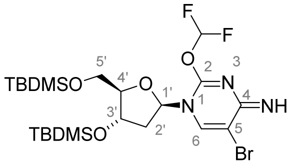

37

To a mixture of DCM (180 µL) and water (120 µL) was added **29** (107 mg, 200 µmol, 1.00 equiv) and KF_2H (94.0 mg, 1.20 mmol, 6.00 equiv) and the mixture was stirred vigorously. $TMSCF_2Br$ (94.0 µL, 600 µmol, 3.00 equiv) was added dropwise and the biphasic solution was stirred for 5 h at 0 °C then diluted with DCM (2 mL) and water (10 mL) and separated. The aqueous phase was extracted with DCM (3 × 10 mL) and the combined organic phase was dried over Na_2SO_4, filtered, and concentrated *in vacuo*. Purification by column chromatography (silica gel, 5% → 40% EtOAc in n-hexane) afforded **37** as a colourless foam (40.0 mg, 34%).

^1H NMR (400 MHz, $CDCl_3$) δ 8.12 (s, 1H, 6-CH), 7.58 (t, *J* = 57.4 Hz, 1H, 2-OCF_2H), 6.27 (dd, *J* = 7.6, 5.7 Hz, 1H, 1′-CH), 4.48 – 4.36 (m, 1H, 2′-CH), 4.01 (app q, *J* = 2.3 Hz, 1H, 4′-CH), 3.91 (dd, *J* = 11.6, 2.3 Hz, 1H, 5′-CH_aH_b), 3.77 (dd, *J* = 11.5, 2.2 Hz, 1H, 5′-CH_aH_b), 2.36 (ddd, *J* = 13.3, 5.8, 2.6 Hz, 1H, 2′-CH_aH_b), 2.02 (ddd, *J* = 13.2, 7.6, 5.7 Hz, 1H, 2′-CH_aH_b), 0.94 (s, 9H, Si^tBu), 0.89 (s, 9H, Si^tBu), 0.15 (s, 3H, $SiCH_3$), 0.14 (s, 3H, $SiCH_3$), 0.09 (s, 3H, $SiCH_3$), 0.08 (s, 3H, $SiCH_3$).

$^{13}C\{^1H\}$ NMR (101 MHz, $CDCl_3$) δ 157.1 (4-C=N), 147.5 (2-*C*-OCF2H), 139.5 (6-CH), 108.9 (t, *J* = 256.3 Hz, 2-*O*-CF_2H), 96.0 (5-CBr), 88.9 (4′-CH), 86.7 (1′-CH), 72.6 (3′-CH), 63.1 (5′-CH_2), 42.3 (2′-CH_2), 26.2 (3 × SiC*C*H_3), 25.8 (3 × SiC*C*H_3), 18.6 (SiC), 18.1 (SiC), −4.5 ($SiCH_3$), −4.7 ($SiCH_3$), −5.1 ($SiCH_3$), −5.2 ($SiCH_3$).

^{19}F NMR (376 MHz, $CDCl_3$) δ −108.30 (dd, *J* = 232.2, 57.4 Hz, CF_aF_bH), −108.98 (dd, *J* = 232.2, 57.4 Hz, CF_aF_bH).

HRMS-ESI Exact mass calculated for $[M + H]^+$ ($C_{22}H_{41}{}^{79}BrF_2N_3O_4Si_2$) requires *m/z* 584.1787, found $[M + H]^+$ *m/z* 584.1777.

3′,5′-di-O-TBDMS-2-O-difluoromethyl-5-fluoro-2′-deoxycytidine (38)

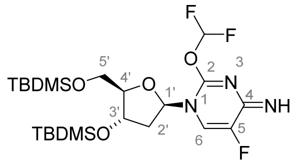

38

To a mixture of DCM (180 µL) and water (120 µL) was added **30** (95.0 mg 200 µmol, 1.00 equiv) and KF_2H (94.0 mg, 1.20 mmol, 6.00 equiv) and the mixture was stirred vigorously. $TMSCF_2Br$ (94.0 µL, 600 µmol, 3.00 equiv) was added dropwise and the biphasic solution was stirred for 5 h at 0 °C then diluted with DCM (2 mL) and water (10 mL) and separated. The aqueous phase was extracted with DCM (3 × 10 mL) and the combined organic phase was dried over Na_2SO_4, filtered, and concentrated *in vacuo*. Purification by column chromatography (silica gel, 5% → 40% EtOAc in n-hexane) afforded **38** as a colourless foam (35.0 mg, 34%).

^{1}H NMR (400 MHz, $CDCl_3$) δ 7.80 (t, *J* = 57.7 Hz, 1H, OCF_2H), 7.53 (d, *J* = 6.1 Hz, 1H, 6-CH), 6.29 (ddd, *J* = 7.9, 6.2, 2.0 Hz, 1H, 1′-CH), 4.38 (app dt, *J* = 6.2, 3.3 Hz, 1H, 3′-CH), 3.97 – 3.84 (m, 2H, 4′-CH, 5′-CH_aH$_b$), 3.80 – 3.70 (m, 1H, 5′-CH$_a$$H_b$), 2.25 (ddd, *J* = 13.2, 6.0, 3.5 Hz, 1H, 2′-CH_aH$_b$), 2.01 (app dt, *J* = 13.1, 6.5 Hz, 1H, 2′-CH$_a$$H_b$), 0.93 (s, 9H, Si^{t}Bu), 0.89 (s, 9H Si^{t}Bu), 0.12 (s, 1H, $SiCH_3$), 0.12 (s, 3H, $SiCH_3$), 0.08 (s, 3H, $SiCH_3$), 0.07 (s, 3H, $SiCH_3$).

^{13}C{^{1}H} NMR (101 MHz, $CDCl_3$) δ 141.6 (C), 126.3 (C), 115.4 (6-CH), 87.4 (4′-CH), 84.8 (1′-CH), 71.2 (3′-CH), 63.0 (5′-CH_2), 40.8 (2′-CH_2), 25.4 (3 × SiCCH_3), 25.2 (3 × SiCCH_3), 18.6 (SiC), 18.1 (SiC), −5.1 ($SiCH_3$), −5.4 ($SiCH_3$), −6.1 ($SiCH_3$), −6.1 ($SiCH_3$).

^{19}F NMR (376 MHz, $CDCl_3$) δ −108.40 – −110.36 (m).

Assignment not possible due to low intensity carbon spectra, likely due to splitting caused by the 5-F atom.

HRMS-ESI Exact mass calculated for $[M + H]^+$ ($C_{22}H_{41}F_3N_3O_4Si_2$) requires *m/z* 524.2588, found $[M + H]^+$ *m/z* 524.2579.

2-S-difluoromethyl-2-thio-cytosine (39)

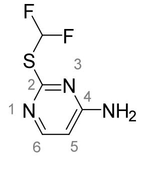

39

To a mixture of DCM (180 µL) and water (120 µL) was added **31** (94.4 mg, 200 µmol, 1.00 equiv) and KF_2H (94.0 mg, 1.20 mmol, 6.00 equiv) and the mixture was stirred vigorously. $TMSCF_2Br$ (94.0 µL, 600 µmol, 3.00 equiv) was added dropwise and the biphasic solution was stirred for 5 h at 0 °C then diluted with DCM (2 mL) and water (10 mL) and separated. The aqueous phase was extracted with

DCM (3 × 10 mL) and the combined organic phase was dried over Na_2SO_4, filtered, and concentrated *in vacuo*. Purification by column chromatography (silica gel, 5% → 40% EtOAc in n-hexane) afforded **39** as a yellow oil (16 mg, 48%)

^{1}H NMR (400 MHz, $CDCl_3$) δ 8.05 (d, J = 5.8 Hz, 1H, 6-CH), 7.74 (t, J = 56.0 Hz, 1H, 2-SCF_2H), 6.21 (d, J = 5.8 Hz, 1H, 6-CH), 5.07 (s, 2H, 4-NH_2).

^{13}C{^{1}H} NMR (101 MHz, $CDCl_3$) δ 166.8 (2-C-SCF_2H), 162.7 (4-C-NH_2), 156.3 (6-CH), 121.2 (t, J = 269.1 Hz, CF_2H), 102.6 (5-CH).

^{19}F NMR (376 MHz, $CDCl_3$) δ −98.80 (d, J = 56.0 Hz, CF_2H).

HRMS-ESI Exact mass calculated for $[M + H]^+$ ($C_5H_6F_2N_3S$) requires *m/z* 178.0250, found $[M + H]^+$ *m/z* 178.0241.

2-O-difluoromethyl-cytidine (40)

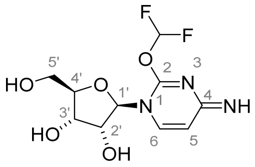

40

To a solution of **35** (235 mg, 350 µmol, 1.00 equiv) in methanol (680 µL) was added TFA (270 µL, 3.50 mmol, 10.0 equiv) and water (63.0 µL, 3.50 mmol, 10.0 equiv) at 0 °C. The mixture was stirred at rt for 4 h then concentrated *in vacuo*. Purification by column chromatography (silica gel, 4 → 8% MeOH in DCM) afforded **40** as a colourless oil (45.6 mg, 45%).

^{1}H NMR (400 MHz, CD_3CN) δ 7.74 (t, J = 57.4 Hz, 1H, 2-OCF_2H), 7.65 (d, J = 8.4 Hz, 1H, 6-CH), 5.94 (d, J = 8.2 Hz, 1H, 5-CH), 5.75 (d, J = 3.7 Hz, 1H, 1′-CH), 4.14 – 4.06 (m, 2H, 2′-CH, 3′-CH), 3.94 (app dt, J = 5.0, 2.8 Hz, 1H, 4′-CH), 3.79 (dd, J = 12.3, 2.7 Hz, 1H, 5′-CH_aH_b), 3.67 (dd, J = 12.3, 2.9 Hz, 1H, 5′-CH_aH_b).

^{13}C{^{1}H} NMR (101 MHz, CD_3CN) δ 150.14 (4-C=N), 148.91 (2-*C*-OCF2H), 138.15 (6-CH), 110.09 (t, J = 252.2 Hz, OCF2H), 97.59 (5-CH), 90.68 (1′-CH), 85.69 (4′-CH), 75.30 (2′-CH), 70.51 (3′-CH), 61.59 (5′-CH_2).

^{19}F NMR (376 MHz, CD_3CN) δ −109.86 (dd, J = 230.8, 57.5 Hz, CF_aF_bH), −110.83 (dd, J = 230.8, 57.5 Hz, CF_aF_bH).

HRMS-ESI Exact mass calculated for $[M + Na]^+$ ($C_{10}H_{13}F_2N_3O_5Na$) requires *m/z* 316.0721, found $[M + Na]^+$ *m/z* 316.0714.

2-O-difluoromethyl-2'-deoxycytidine (41)

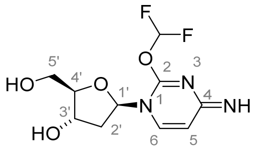

41

Compound **34** (40.0 mg, 73.0 µmol, 1.00 equiv) was dissolved in methanol (120 µL) was added TFA (56.0 uL, 730 µmol, 10.0 equiv) and water (13.0 µL, 730 µmol, 10.0 equiv) at 0 °C. The mixture was stirred at rt for 4 h then concentrated *in vacuo*. Purification by column chromatography (silica gel, 4 → 8% MeOH in DCM) afforded **41** as a colourless oil (11.0 mg, 54%).

1.5 mmol telescoped procedure from cytidine:

TBDMS-Cl (1.02 g, 6.75 mmol, 4.50 equiv) was added to a stirred solution of deoxycytidine (341 mg, 1.50 mmol, 1.00 equiv) and imidazole (408 mg, 6.00 mmol, 4.00 equiv) in anhydrous DMF (3.63 mL) and the mixture was stirred at rt for 18 h under an argon atmosphere. Once complete, the reaction mixture was concentrated *in vacuo* then partitioned between EtOAc (40 mL) and water (40 mL) and separated. The organic phase was washed with water (3 × 40 mL) and LiCl (5%, aq., 40 mL), dried over Na_2SO_4, filtered, and concentrated *in vacuo* to afford a colourless solid foam (534 mg, 78% crude yield) This solid (534 mg) was dissolved in anhydrous pyridine (5.69 mL) and DMAP (12.0 mg, 117 µmol, 10.0 mol%) and acetic anhydride (133 µL, 1.41 mmol, 1.20 equiv) were added sequentially at 0 °C under an argon atmosphere. The suspension was allowed to stir vigorously overnight at rt then water (1 mL) was added to quench the reaction and then the mixture was concentrated *in vacuo*. The crude mixture was partitioned between DCM (30 mL) and $NaHCO_3$ (sat. aq., 30 mL) and separated. The organic phase was washed with water (3 × 20 mL), dried over Na_2SO_4, filtered, and concentrated *in vacuo* affording a colourless solid (492 mg, 83% crude yield)

This crude solid (492 mg, 0.988 mmol, 1.00 equiv.) and KF_2H (469 mg, 6.00 mmol, 6.00 equiv) were dissolved in a mixture of DCM (600 µL) and water (600 µL) and was stirred vigorously. $TMSCF_2Br$ (467 µL, 3.00 mmol, 3.00 equiv) was then added dropwise and the solution was stirred for **1.5 h** at 0 °C, diluted with DCM (10 mL) and water (50 mL) and separated. The aqueous phase was extracted with DCM (3 × 50 mL) and the combined organic phase was dried over Na_2SO_4, filtered, and concentrated *in vacuo* to afford a yellow oil (470 mg, 86% crude yield).

This oil was dissolved in methanol (1.37 mL) then TFA (1.05 mL, 13.7 mmol, 16.0 equiv) and water (247 µL, 13.7 µmol, 16.0 equiv) were added at 0 °C. The mixture was stirred at rt for 4 h then concentrated *in vacuo*. Purification by column chromatography (silica gel, 4 → 8% MeOH in DCM) afforded **41** as a colourless oil (125 mg, 30%).

^{1}H NMR (400 MHz, CD_3OD) δ 8.08 (d, J = 8.3 Hz, 1H, 6-CH), 7.64 (t, J = 57.4 Hz, 1H, 2-OCF_2H), 6.28 (app t, J = 6.6 Hz, 1H, 1′-CH), 5.78 (d, J = 8.3 Hz, 1H, 5-CH), 4.42 (app dt, J = 6.6, 3.5 Hz, 1H, 2′-CH), 3.98 (d, J = 3.4 Hz, 1H, 4′-CH), 3.82 (dd, J = 12.1, 3.2 Hz, 1H, 5′-CH_aH_b), 3.75 (dd, J = 12.1,

3.7 Hz, 1H, 5′-CH$_a$*H$_b$*), 2.37 (ddd, *J* = 13.7, 6.2, 3.7 Hz, 1H, 2′-C*H$_a$*H$_b$), 2.26 (ddd, *J* = 13.6, 7.0, 6.2 Hz, 1H, 2′-CH$_a$*H$_b$*).

^{13}C{^{1}H} NMR (101 MHz, CD$_3$OD) δ 162.6 (4-C=NH), 149.9 (2-*C*-OCF$_2$H), 142.3 (6-CH), 110.1 (t, *J* = 251.9 Hz, OCF$_2$H), 102.0 (6-CH), 89.2 (4′-CH), 87.3 (1-CH), 72.1 (3′-CH), 62.7 (5′-CH$_2$), 41.4 (2′-CH$_2$).

^{19}F NMR (376 MHz, CD$_3$OD) δ −110.34 (dd, *J* = 234.4, 57.4 Hz, C*F$_a$*F$_b$H), −111.06 (dd, *J* = 234.1, 58.0, CF$_a$*F$_b$*H).

HRMS-ESI Exact mass calculated for [M + Na]$^+$ ($C_{10}H_{13}F_2N_3O_4Na$) requires *m/z* 300.0772, found [M + Na]$^+$ *m/z* 300.0765.

2-O-difluoromethyl-5-fluoro-2'-deoxycytidine (42)

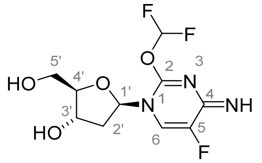

42

To a solution of **38** (44.0 mg, 77.0 μmol, 1.00 equiv) in methanol (150 μL) was added TFA (59.0 μL, 770 μmol, 10.0 equiv) and water (14.0 μL, 770 μmol, 10.0 equiv) at 0 °C. The mixture was stirred at room temperature for 4 hours then concentrated *in vacuo*. Purification by column chromatography (silica gel, 4 → 8% MeOH in DCM) afforded **42** as a colourless oil (5.00 mg, 23%).

^{1}H NMR (400 MHz, CD$_3$OD) δ 8.37 (d, *J* = 6.9 Hz, 1H, 6-CH), 7.60 (t, *J* = 57.1 Hz, 1H, 2-OCF$_2$H), 6.25 (app td, *J* = 6.5, 1.7 Hz, 1H, 1′-CH), 4.40 (app dt, *J* = 6.2, 3.6 Hz, 1H, 3′-CH), 3.94 (app q, *J* = 3.2 Hz, 1H, 4′-CH), 3.82 (dd, J = 12.0, 2.9 Hz, 1H, 5′-C*H$_a$*H$_b$), 3.74 (dd, J = 12.0, 3.2 Hz, 1H, 5′-CH$_a$*H$_b$*), 2.33 (ddd, *J* = 13.7, 6.2, 3.8 Hz, 1H, 2′-C*H$_a$*H$_b$), 2.24 (app dt, *J* = 13.4, 6.4 Hz, 1H, 2′-CH$_a$*H$_b$*).

^{13}C{^{1}H} NMR (101 MHz, CD$_3$OD) δ 156.2 (4-C=NH), 148.4 (2-*C*-OCF$_2$H), 126.1 (6-CH), 110.3 (OCF$_2$H), 89.3 (4′-CH), 87.5 (1′-CH), 71.9 (3′-CH), 62.4 (5′-CH$_2$), 30.7 (2′-CH$_2$).

^{19}F NMR (376 MHz, CD$_3$OD) δ -106.18 (dd, *J* = 234.2, 57.0 Hz, C*F$_a$*F$_b$H), -106.56 (dd, *J* = 235.4, 57.1 Hz, CF$_a$*F$_b$*H).

N.B. Fluorine NMR does not pick up the 5-F, but splitting can be seen from the 6-H signal attributed to the fluorine. Two quaternary carbon signals cannot be seen on the carbon NMR (4-C and 2-C), but can be identified from the HMBC correlation with the 6-CH proton.

4-O-difluoromethyl-uridine (43)

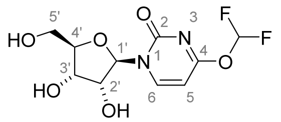

43

To a mixture of **19** (50.0 mg, 79.0 µmol, 1.00 equiv) in anhydrous THF (50.0 uL) was added TEA.3HF (126 µL, 790 µmol, 10.0 equiv) at 0 °C under an argon atmosphere and the mixture was then stirred at rt for 18 h. Once complete, the reaction was neutralized with Na_2CO_3 (0.57 M, aq., 1 mL) at 0 °C then MeCN (2 mL) and brine (2 mL) were added, and the mixture was separated. The aqueous phase was washed with MeCN (3 × 2 mL) and the combined organic phase was washed with brine, dried over anhydrous Mg_2SO_4, filtered, and concentrated *in vacuo.* Purification by column chromatography (silica gel, 4 → 8% MeOH in DCM) afforded **43** as a colourless amorphous solid (11 mg, 48%).

^{1}H NMR (400 MHz, CD_3CN) δ 8.49 (d, *J* = 7.3 Hz, 1H, 6-CH), 7.48 (t, *J* = 71.4 Hz, 1H, 4-OCF_2H), 6.11 (d, *J* = 7.3 Hz, 1H, 5-CH), 5.76 (d, *J* = 2.2 Hz, 1H, 1′-CH) 4.16 – 4.08 (m, 2H, 2′-CH, 3′-CH), 4.03 (app dt, *J* = 5.7, 2.7 Hz, 1H, 4′-CH) 3.88 (ddd, *J* = 12.5, 4.6, 2.5 Hz, 1H, 5′-CH_aH$_b$), 3.72 (ddd, *J* = 12.5, 4.7, 2.8 Hz, 1H, 5′-CH$_a$$H_b$), 3.49 (s, 1H, 2′-OH), 3.38 (d, *J* = 7.5 Hz, 1H, 5′-OH).

^{13}C{^{1}H} NMR (101 MHz, CD_3CN) δ 168.7 (4-*C*-OCF_2H), 155.5 (2-C=O), 148.2 (6-CH), 114.6 (d, *J* = 256.7 Hz, OCF_2H), 94.6 (5-CH), 92.9 (1′-CH), 85.8 (4′-CH), 76.2 (2′-CH), 70.0 (3′-CH), 61.1 (2′-CH).

^{19}F NMR (376 MHz, CD_3CN) δ −91.36 (dd, *J* = 176.4, 71.5 Hz, CF_aF$_b$H), −91.72 (dd, *J* = 176.4, 71.5 Hz, CF$_a$$F_b$H).

HRMS-ESI Exact mass calculated for $[M + Na]^+$ ($C_{10}H_{12}F_2N_2O_6Na$) requires *m/z* 317.0561, found $[M + Na]^+$ *m/z* 317.0547.

4-O-difluoromethyl-thymidine (44)

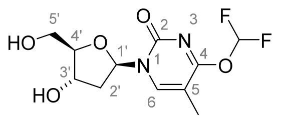

44

To a mixture of **20** (36.0 mg, 79.0 µmol, 1.00 equiv) in anhydrous THF (50.0 µL) was added TEA.3HF (126 µL, 790 µmol, 10.0 equiv) at 0 °C under an argon atmosphere and the mixture was stirred at rt for 18 h. The reaction was neutralized with Na_2CO_3 (0.57 M, 1 mL) at 0 °C then MeCN (2 mL) and brine (sat. aq., 2 mL) were added, and the mixture was separated. The aqueous phase was washed with MeCN (3 × 2 mL) and the combined organic phase was washed with brine, dried over anhydrous Mg_2SO_4, filtered, and concentrated *in vacuo.* Purification by column chromatography (silica gel, 4 → 8% MeOH in DCM) afforded **44** as a colourless amorphous solid (14 mg, 69%).

^{1}H NMR (400 MHz, CD_3CN) δ 8.18 (d, *J* = 1.1 Hz, 1H, 6-CH), 7.47 (t, *J* = 71.6 Hz, 1H, 4-OCF_2H), 6.11 (app t, *J* = 6.1 Hz, 1H, 1′-CH), 4.33 (app dt, *J* = 6.3, 4.4 Hz, 1H, 3′-CH), 3.91 (app q, *J* = 3.6 Hz, 1H, 4′-CH), 3.78 (dd, *J* = 12.1, 3.3 Hz, 1H, 5′-CH_aH$_b$), 3.69 (dd, *J* = 12.1, 3.7 Hz, 1H, 5′-CH$_a$$H_b$), 3.36 (s, 1H, OH), 3.24 (s, 1H, OH), 2.37 (ddd, *J* = 13.6, 6.3, 4.6 Hz, 1H, 2′-CH_aH$_b$), 2.23 – 2.06 (m, 1H, 2′-CH$_a$$H_b$), 1.97 (d, *J* = 1.0 Hz, 3H, 5-Me).

^{13}C NMR (101 MHz, CD_3CN) δ 167.5 (4-*C*-OCF_2H), 154.9 (2-C=O), 145.3 (6-CH), 114.8 (t, J = 256.2 Hz, CF_2H), 103.5 (5-C), 88.8 (4′-CH), 88.0 (1′-CH), 71.0 (3′-CH), 62.1 (5′-CH_2), 41.8 (2′-CH), 11.9 (5-Me).

^{19}F NMR (376 MHz, CD_3CN) δ −91.28 (dd, J = 176.6, 71.4 Hz, CF_aF_bH), −91.77 (dd, J = 176.6, 71.5 Hz, CF_aF_bH).

HRMS-ESI Exact mass calculated for $[2M + Na]^+$ ($C_{22}H_{28}F_4N_4O_{10}Na$) requires m/z 607.1639, found $[2M + Na]^+$ m/z 607.1630.

1.2.1 Crude NMR spectra following difluoromethylation of N-acetylated cytidines

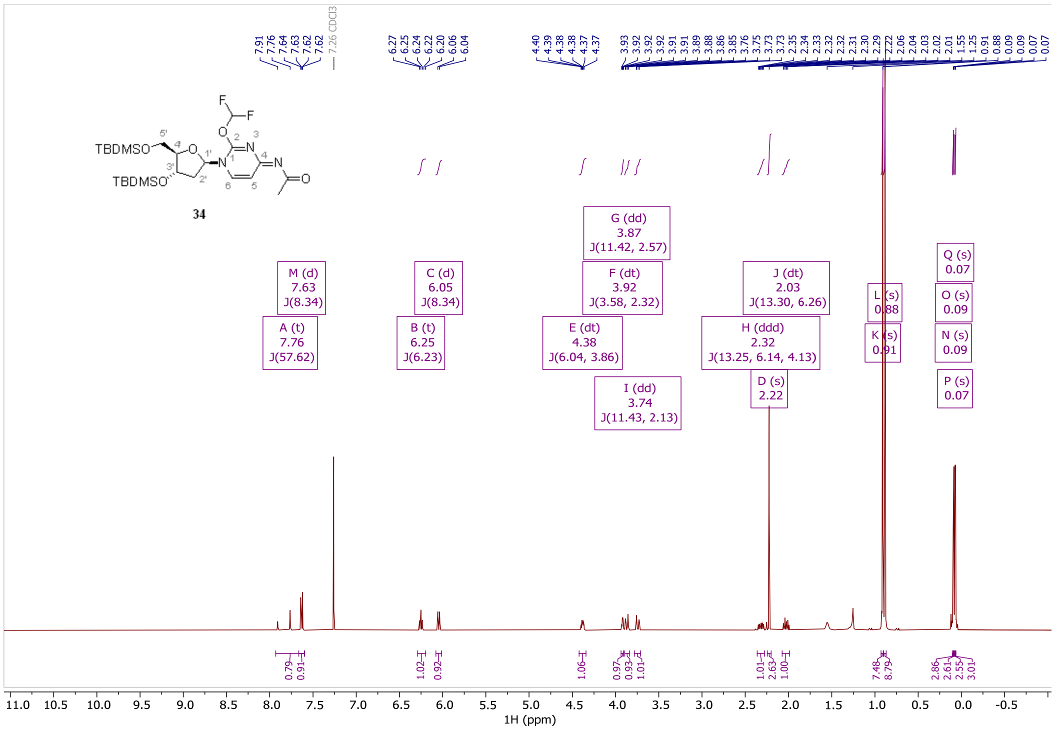

Figure S1 - ^{1}H NMR (400 MHz) spectrum of the crude reaction product of **34** following aqueous workup in $CDCl_3$

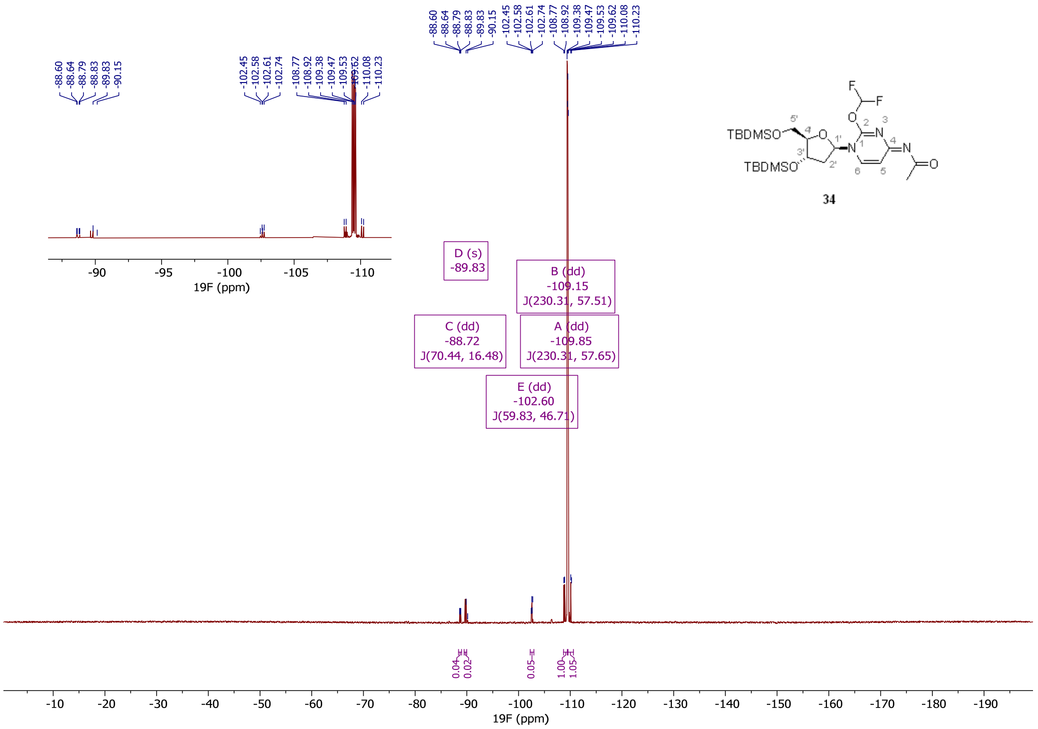

Figure S2 - ^{19}F NMR (400 MHz) spectrum of the crude reaction product of **34** following aqueous workup (376 MHz, $CDCl_3$)

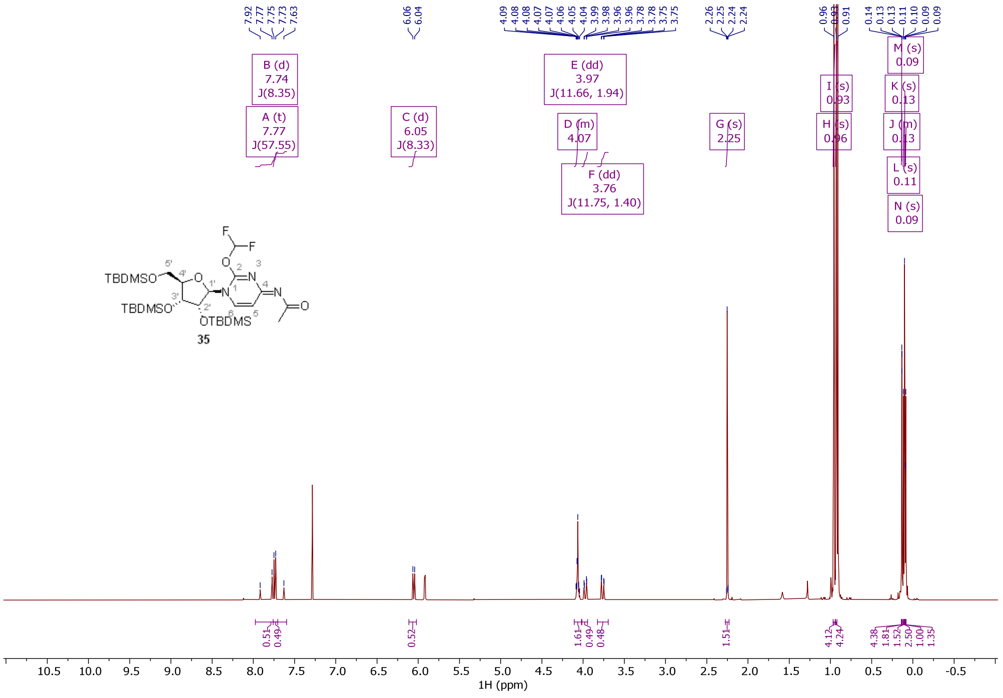

Figure S3 - ^{1}H NMR (400 MHz) spectrum of the crude reaction product of **35** following aqueous workup in $CDCl_3$

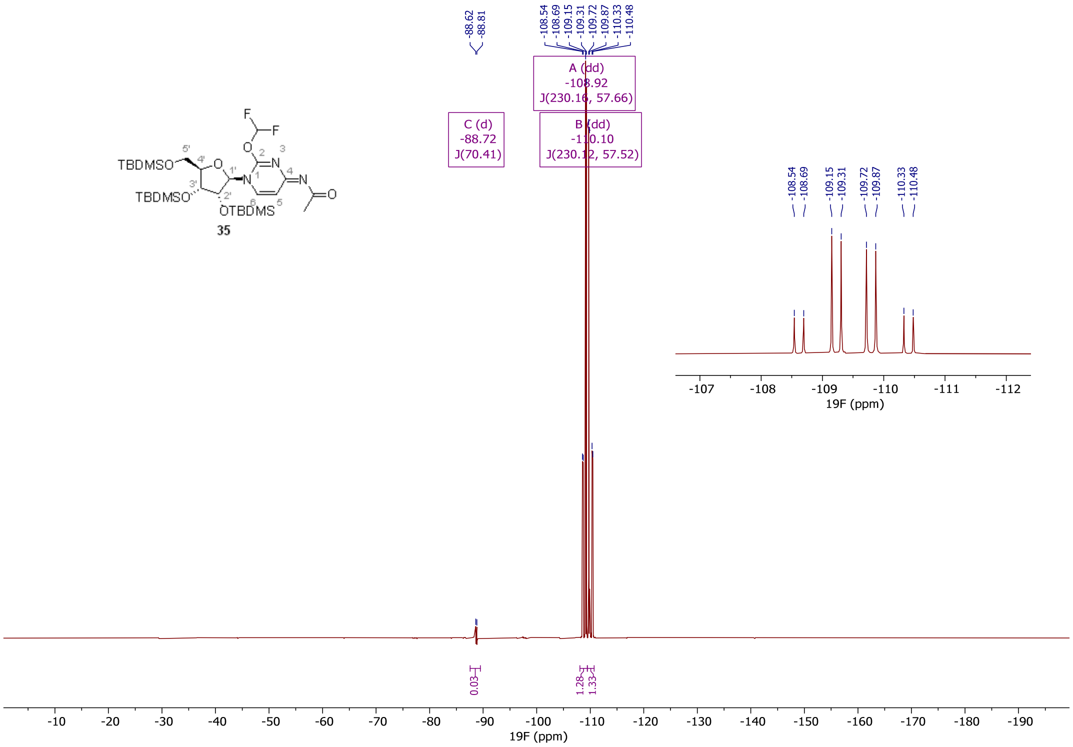

Figure S4 - ^{19}F NMR (376 MHz) spectrum of the crude reaction product of **35** following aqueous workup in $CDCl_3$

1.3 References

(1) Zhang, Y.; Knapp, S. Glycosylation of Nucleosides. *J Org Chem* **2016**, *81* (6), 2228–2242. https://doi.org/10.1021/acs.joc.5b02852.

(2) Grøtli, M.; Undheim, K.; Vares, L.; Kühn, I.; Claesson, A.; Arnarp, J.; Björk, L.; Gawinecki, R. Synthesis of 2'-Allyl-2'-Deoxyribonucleosides by Radical Reactions. *Acta Chem Scand* **1995**, *49* (49), 217–224. https://doi.org/10.3891/acta.chem.scand.49-0217.

(3) Drenichev, M.; Kulikova, I.; Bobkov, G.; Tararov, V.; Mikhailov, S. A New Protocol for Selective Cleavage of Acyl Protecting Groups in 2′-O-Modified 3′,5′-O-(Tetraisopropyldisiloxane-1,3-Diyl)Ribonucleosides. *Synthesis (Stuttg)* **2010**, *2010* (22), 3827–3834. https://doi.org/10.1055/s-0030-1258270.

(4) Ghiazza, C.; Wagner, L.; Fernández, S.; Leutzsch, M.; Cornella, J. Bio-Inspired Deaminative Hydroxylation of Aminoheterocycles and Electron-Deficient Anilines. *Angewandte Chemie International Edition* **2023**, *62* (2), e202212219. https://doi.org/10.1002/anie.202212219.

(5) Hegelein, A.; Müller, D.; Größl, S.; Göbel, M.; Hengesbach, M.; Schwalbe, H. Genetic Code Expansion Facilitates Position-Selective Labeling of RNA for Biophysical Studies. *Chemistry - A European Journal* **2020**, *26* (8), 1800–1810. https://doi.org/10.1002/chem.201904623.

(6) Quinn, J. R.; Zimmerman, S. C. Structure-Function Studies on a Synthetic Guanosine Receptor That Simultaneously Binds Watson-Crick and Hoogsteen Sites. *Journal of Organic Chemistry* **2005**, *70* (19), 7459–7467. https://doi.org/10.1021/jo0501689.

(7) Zheng, X.-A.; Huang, H.-S.; Kong, R.; Chen, W.-J.; Gong, S.-S.; Sun, Q. An Efficient PyAOP-Based C4-Amination Method for Direct Access of Oxidized 5MedC Derivatives. *Tetrahedron* **2018**, *74* (49), 7095–7101. https://doi.org/10.1016/j.tet.2018.10.046.

1.4 Characterisation data

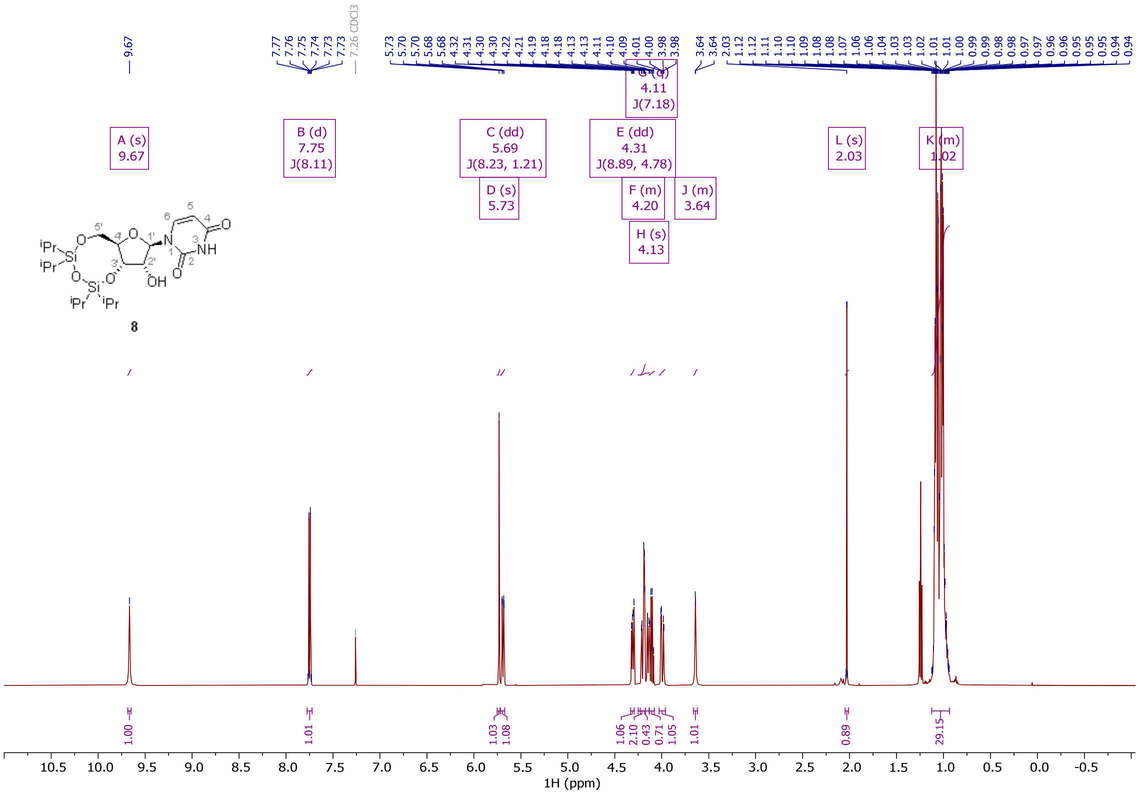

Figure S5 - ^{1}H NMR (500 MHz) spectrum of **8** in $CDCl_3$

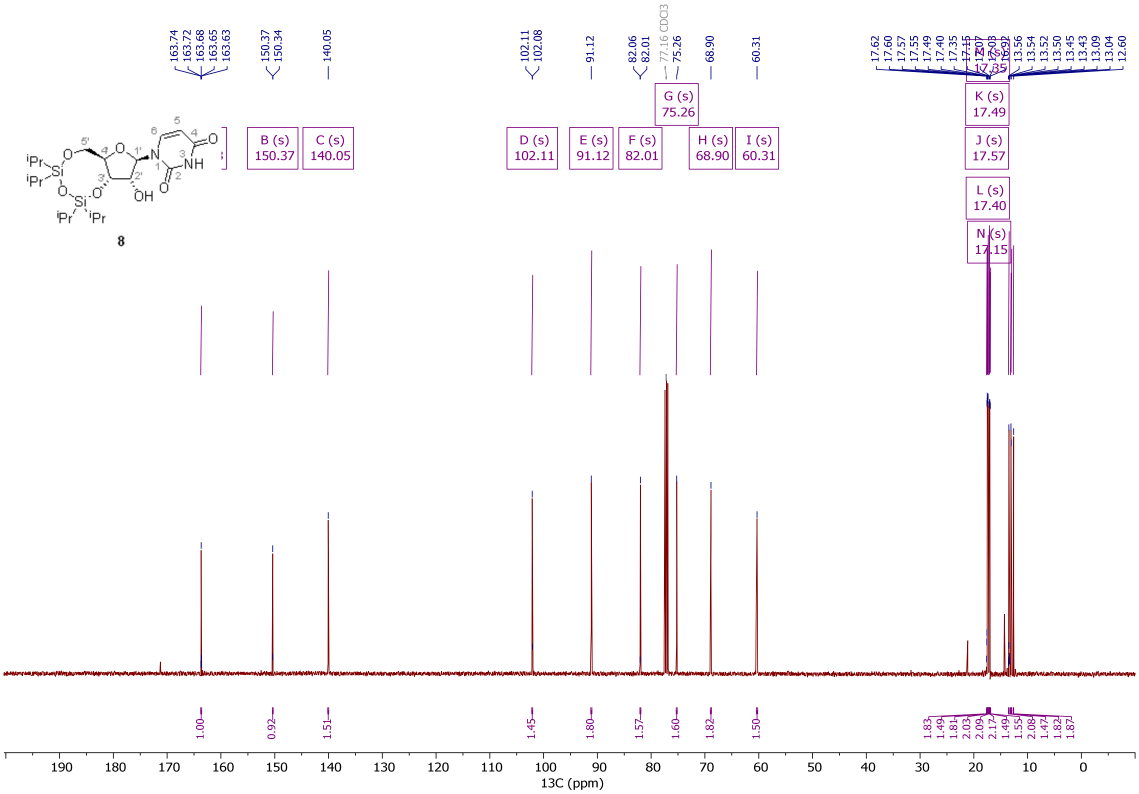

Figure S6 - ^{13}C NMR (126 MHz) spectrum of **8** in $CDCl_3$

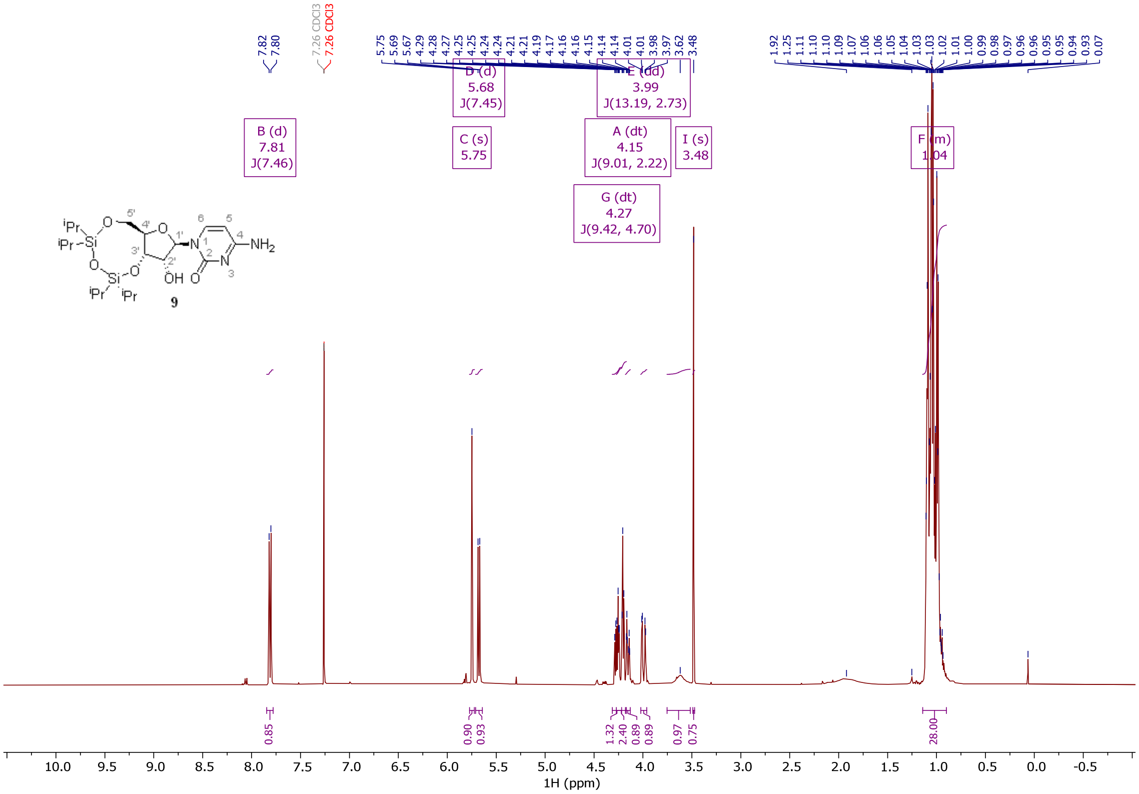

Figure S7 – ^{1}H NMR (400 MHz) spectrum of **9** in $CDCl_3$

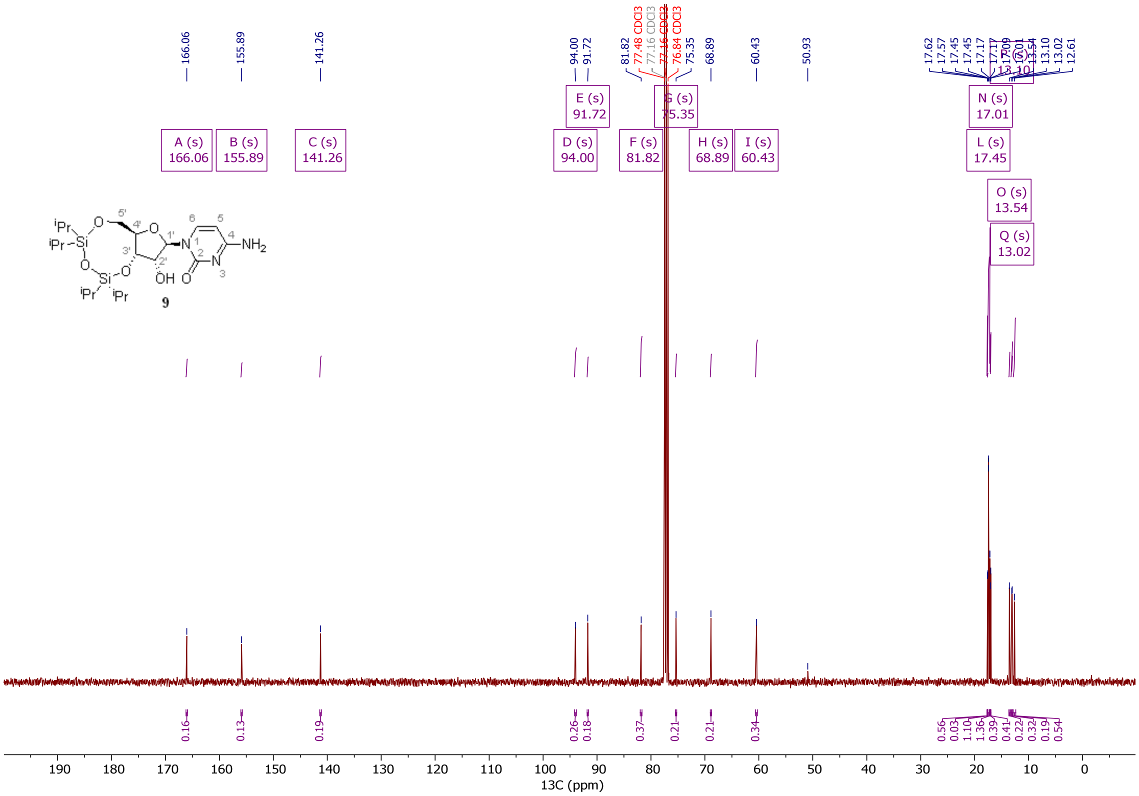

Figure S8 - ^{13}C NMR (101 MHz) spectrum of **9** in $CDCl_3$

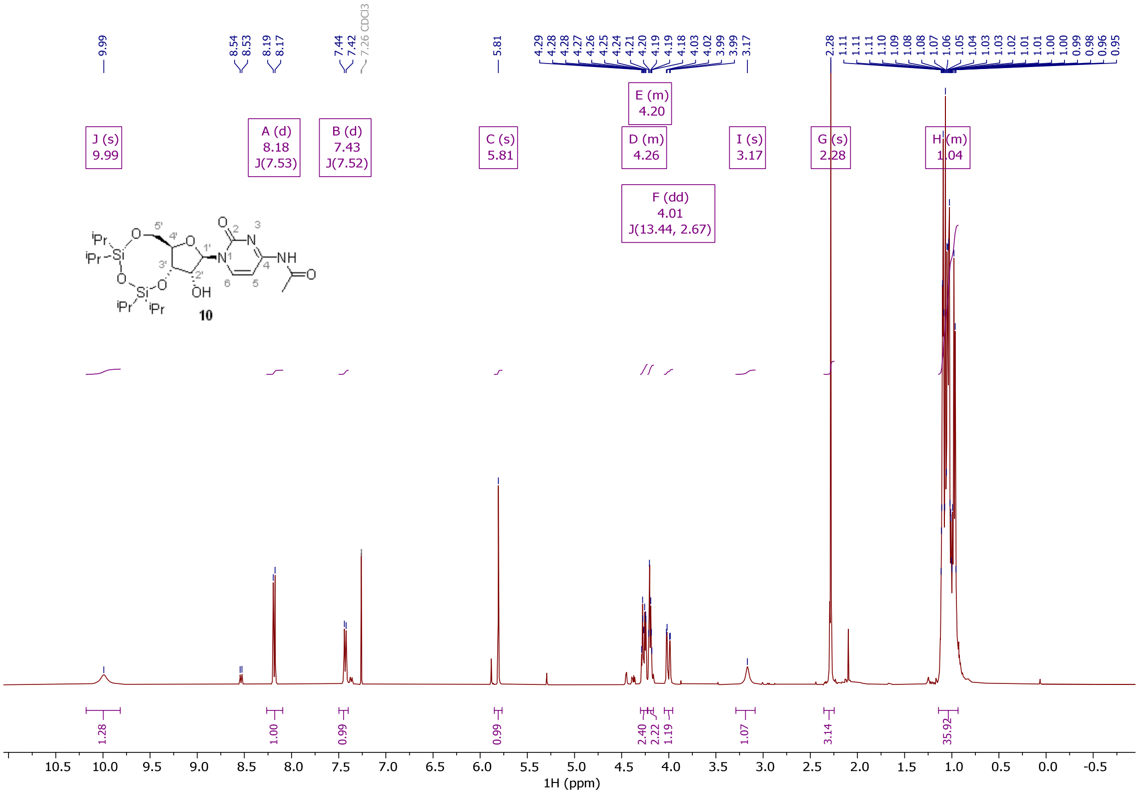

Figure S9 – ^{1}H NMR (400 MHz) spectrum of **10** in $CDCl_3$

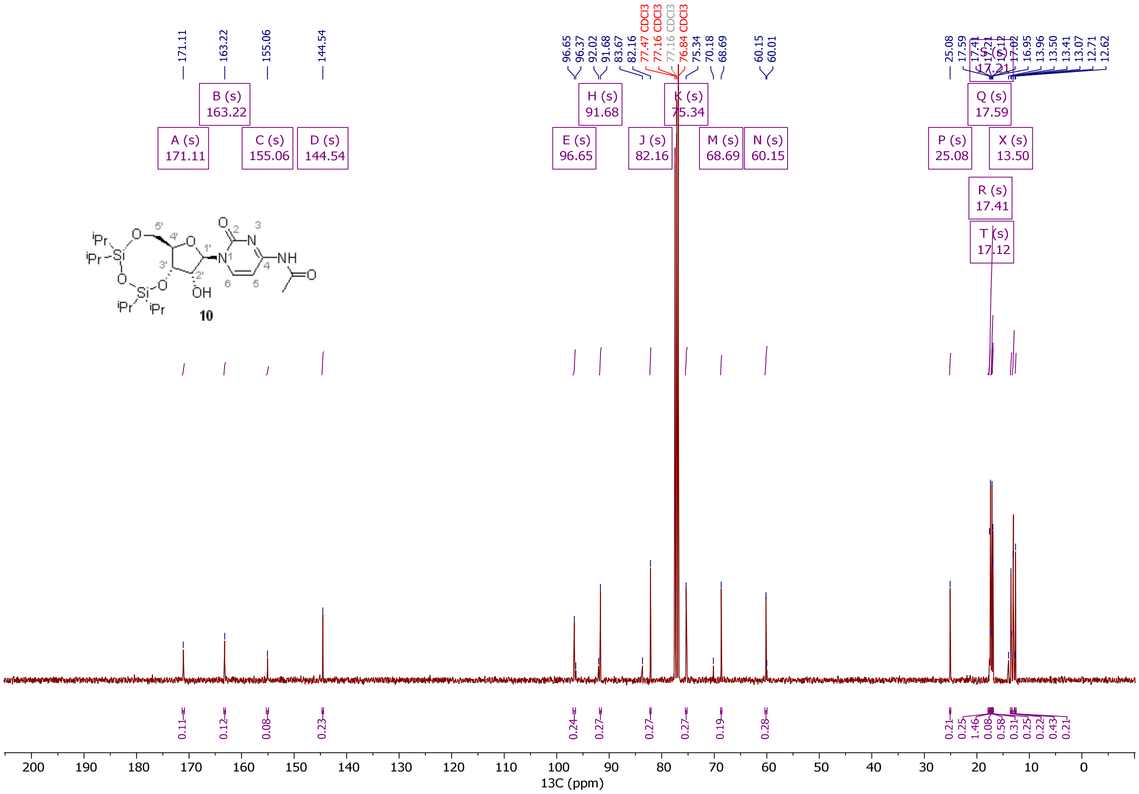

Figure S10 - ^{13}C NMR (101 MHz) spectrum of **10** in $CDCl_3$

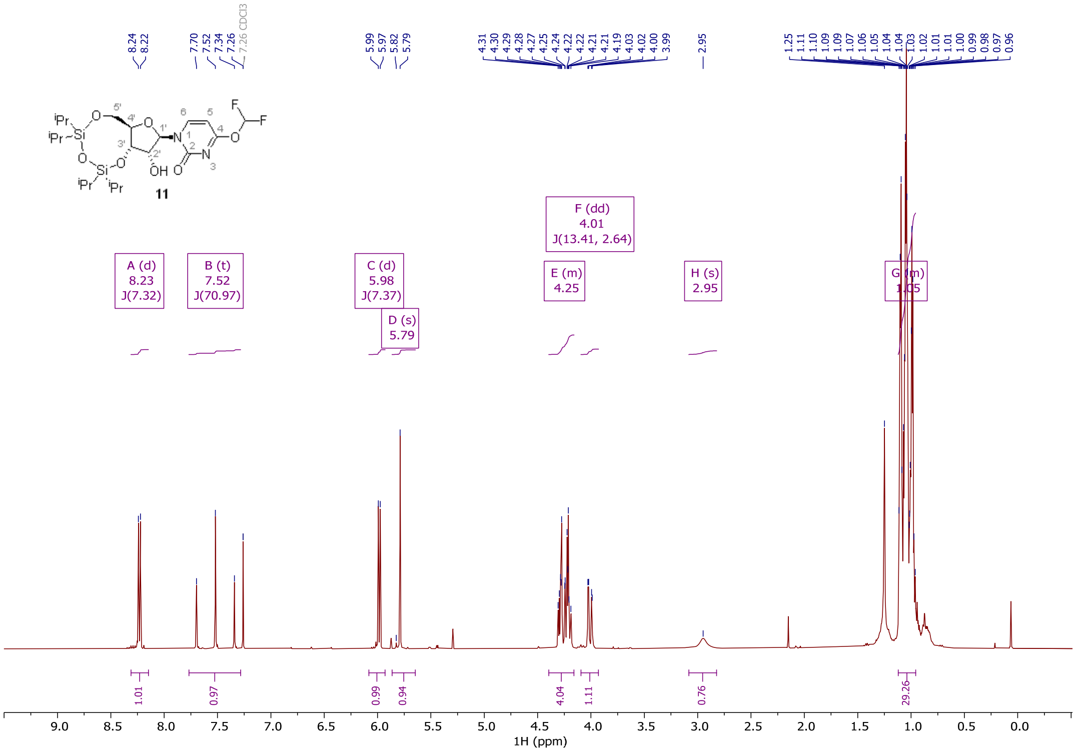

Figure S11 - ^{1}H NMR (400 MHz) spectrum of **11** in $CDCl_3$

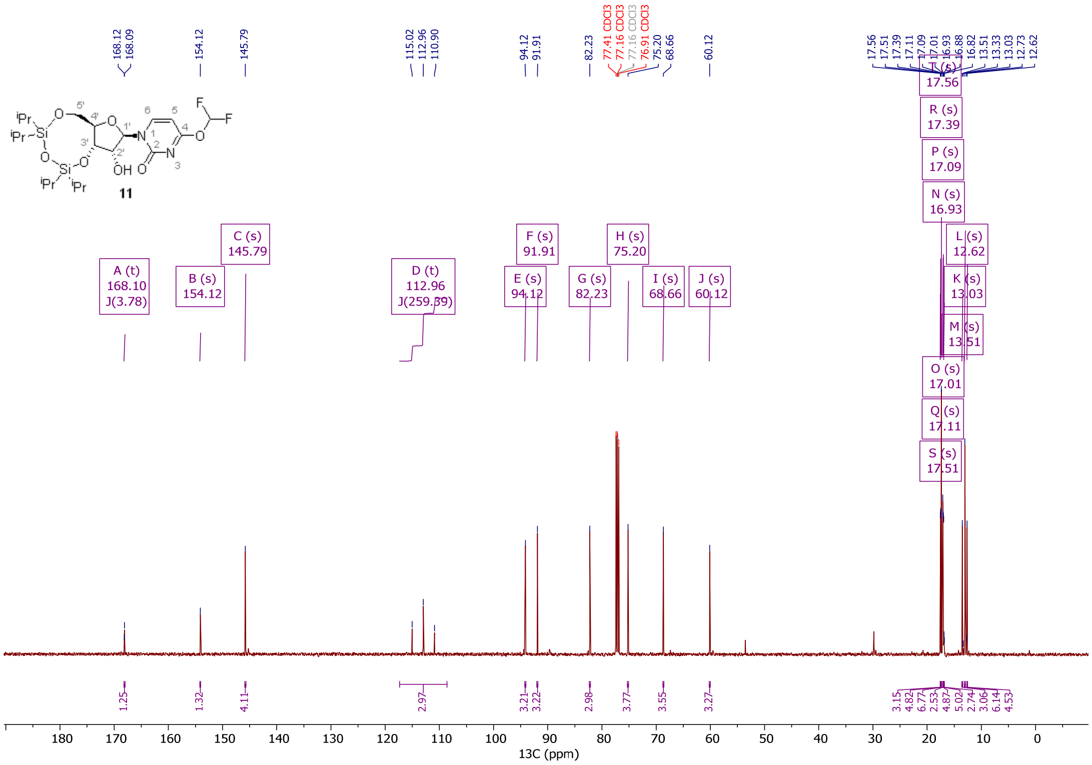

Figure S12 - ^{13}C NMR spectrum of **11** in $CDCl_3$ (101 MHz)

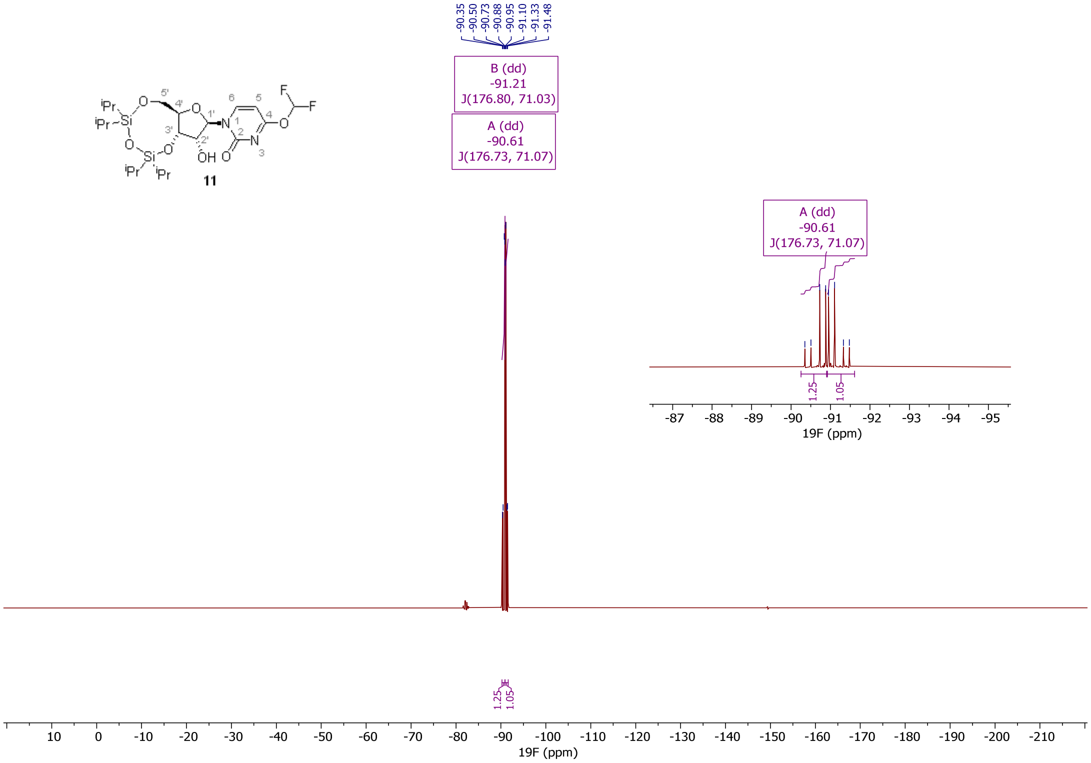

Figure S13 - ^{19}F NMR (376 MHz) spectrum of **11** in $CDCl_3$

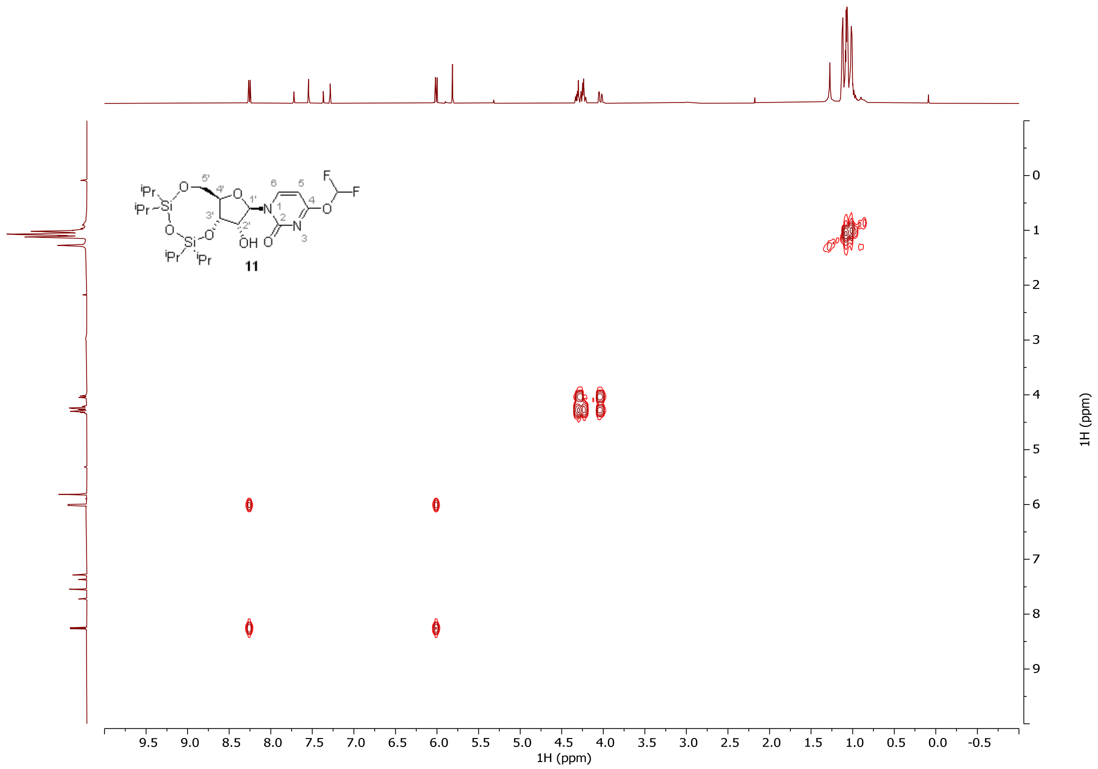

Figure S14 - ^{1}H-^{1}H COSY NMR spectrum of **11** in $CDCl_3$

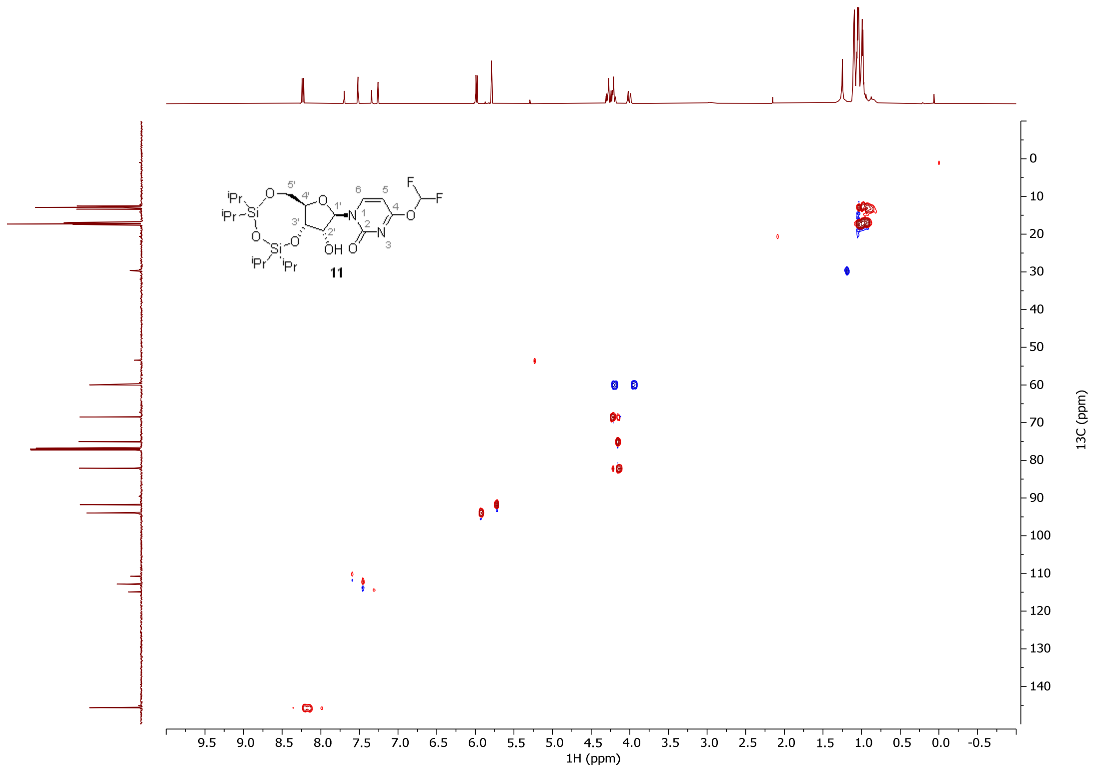

Figure S15 - ^{1}H-^{13}C- NMR spectrum of **11** in $CDCl_3$

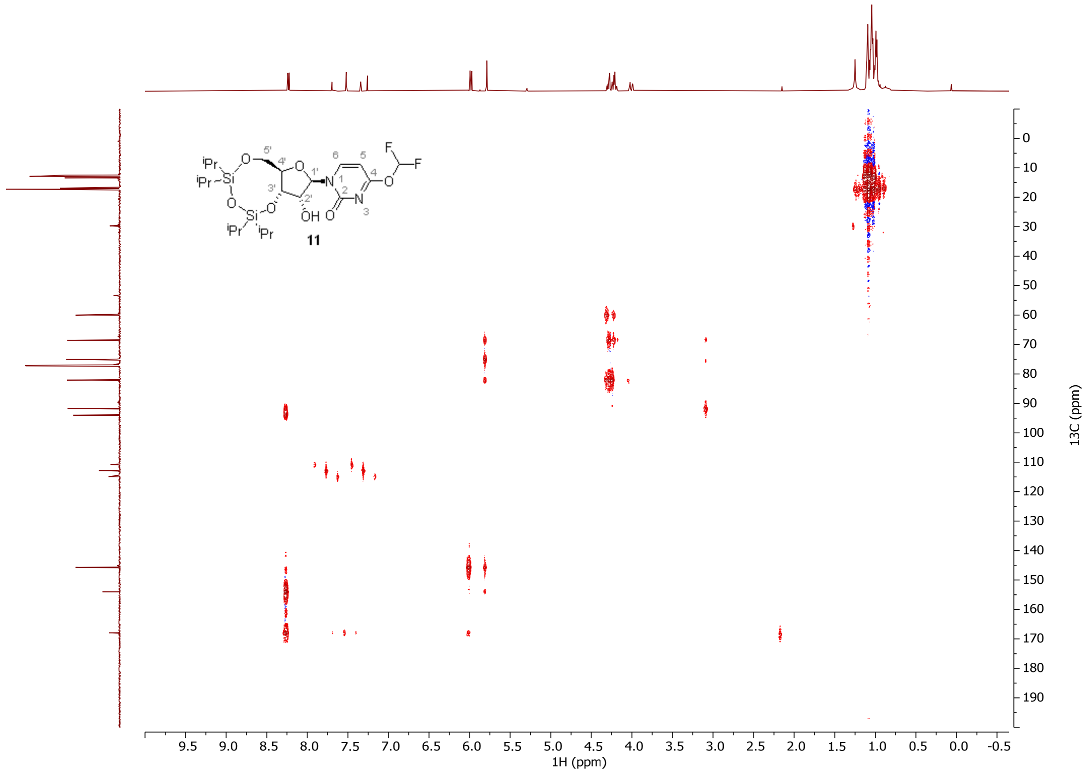

Figure S16 - ^{1}H-^{13}C HMBC NMR spectrum of **11** in $CDCl_3$

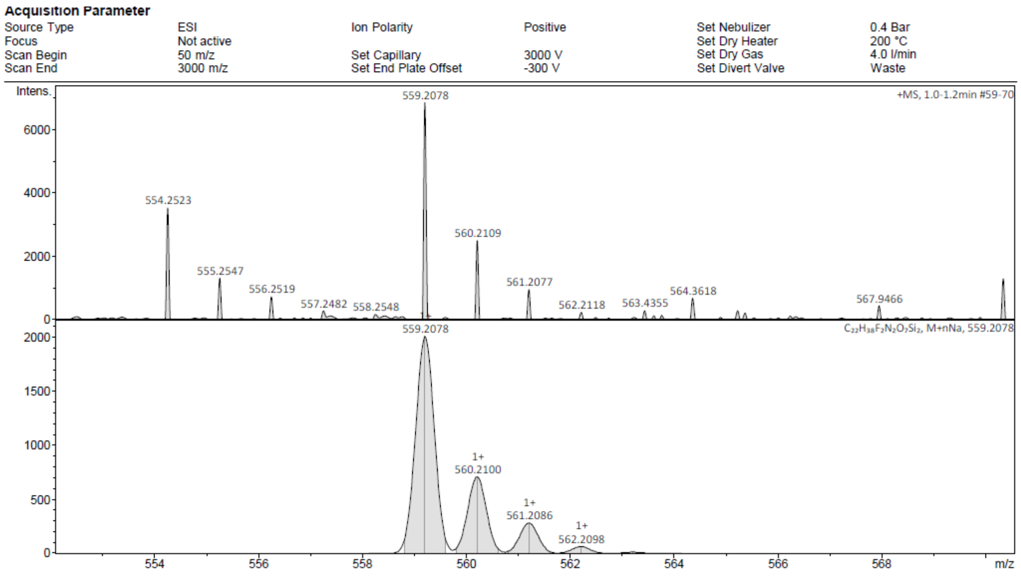

Figure S17 - HRMS spectrum of **11**

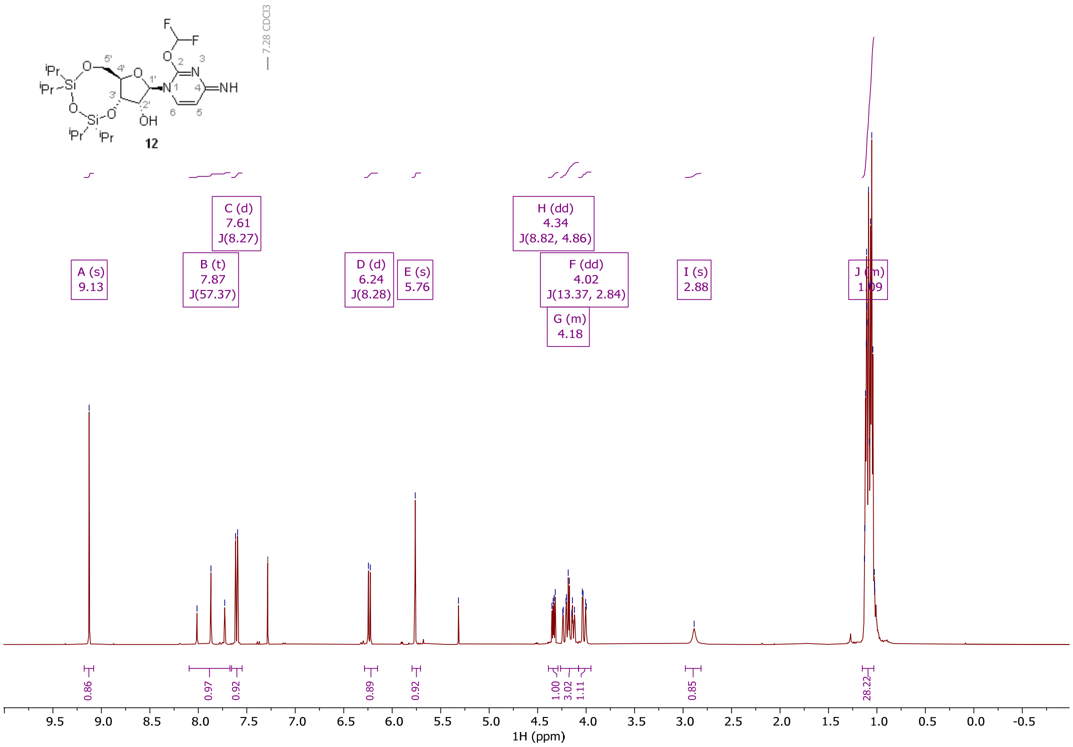

Figure S18 - ^{1}H NMR (400 MHz) spectrum of **12** in $CDCl_3$

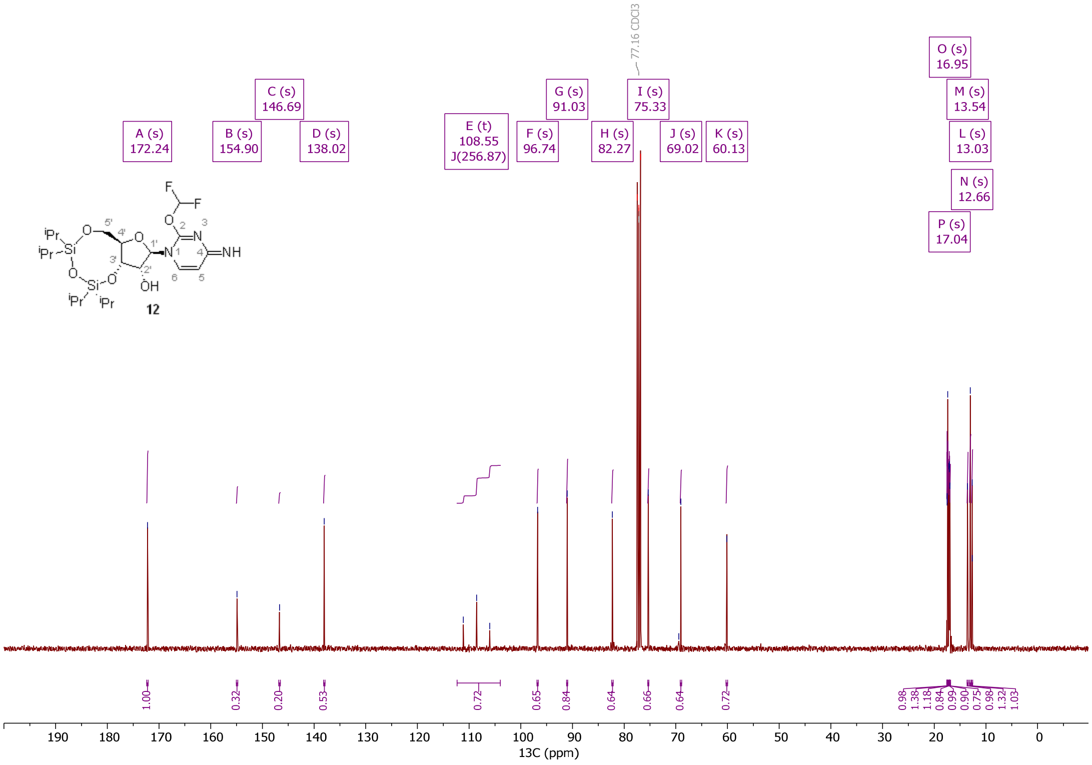

Figure S19 - ^{13}C NMR (101 MHz) spectrum of **12** in $CDCl_3$

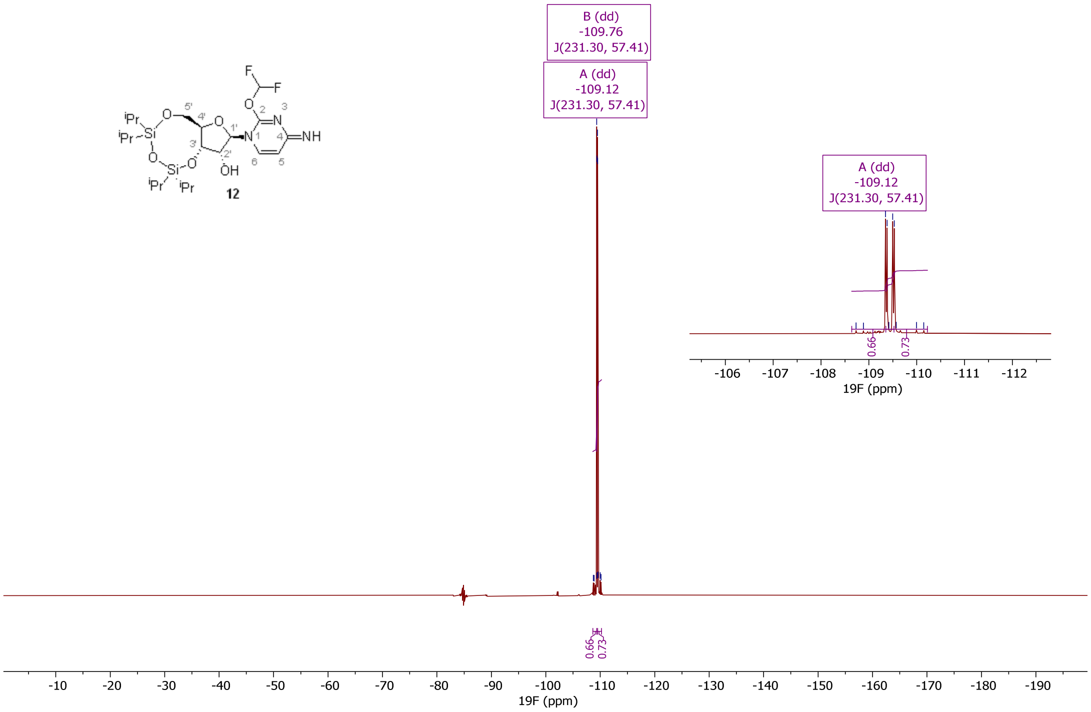

Figure S20 – ^{19}F NMR (376 MHz) spectrum of **12** in $CDCl_3$

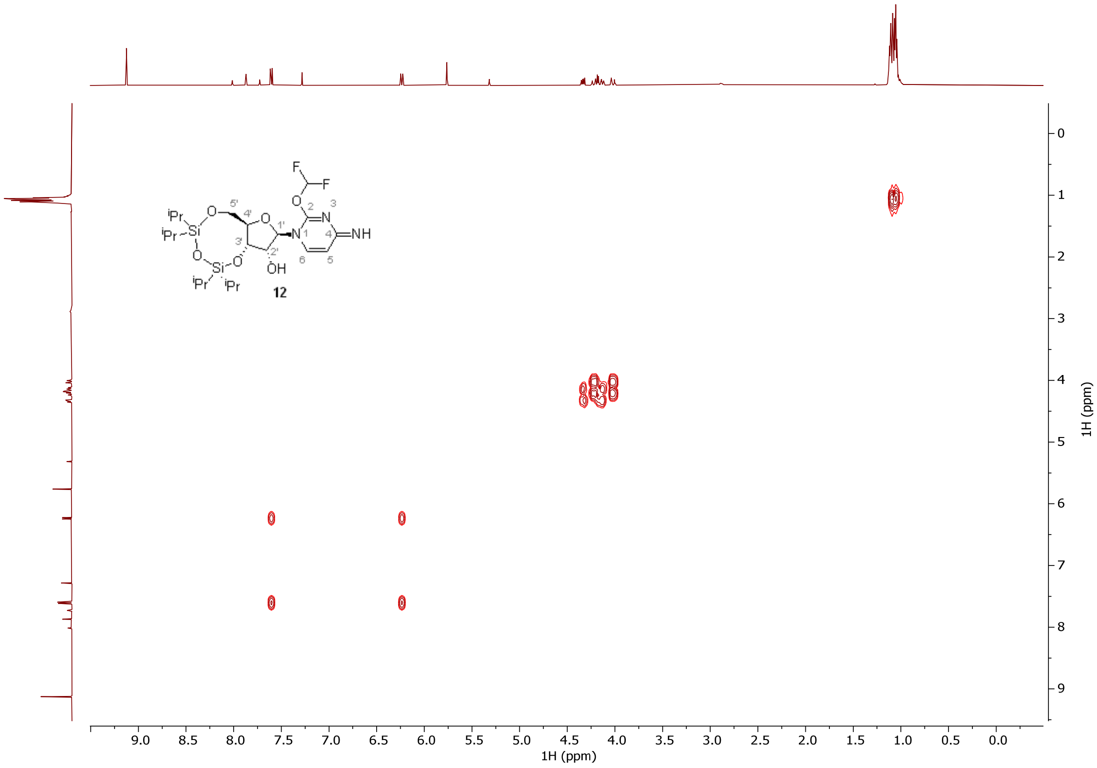

Figure S21 - ^{1}H-^{1}H COSY NMR spectrum of **12** in $CDCl_3$

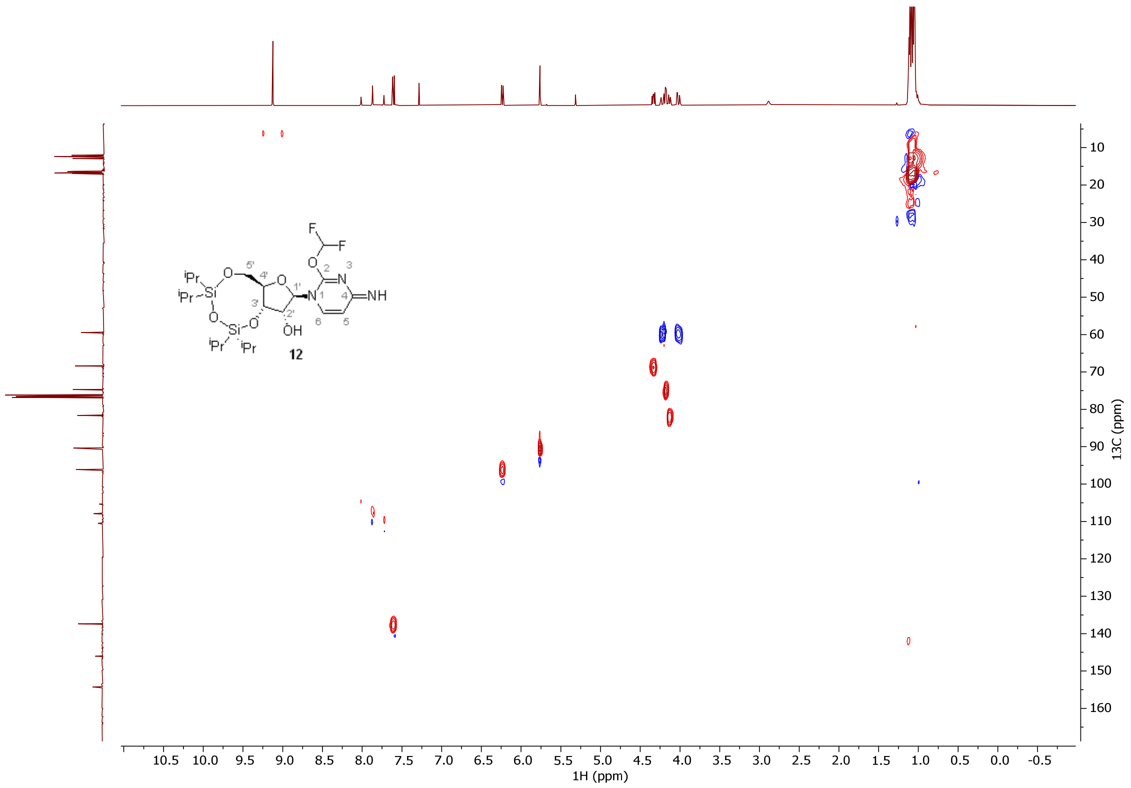

Figure S22 – ^{1}H-^{13}C HSQC NMR spectrum of **12** in $CDCl_3$

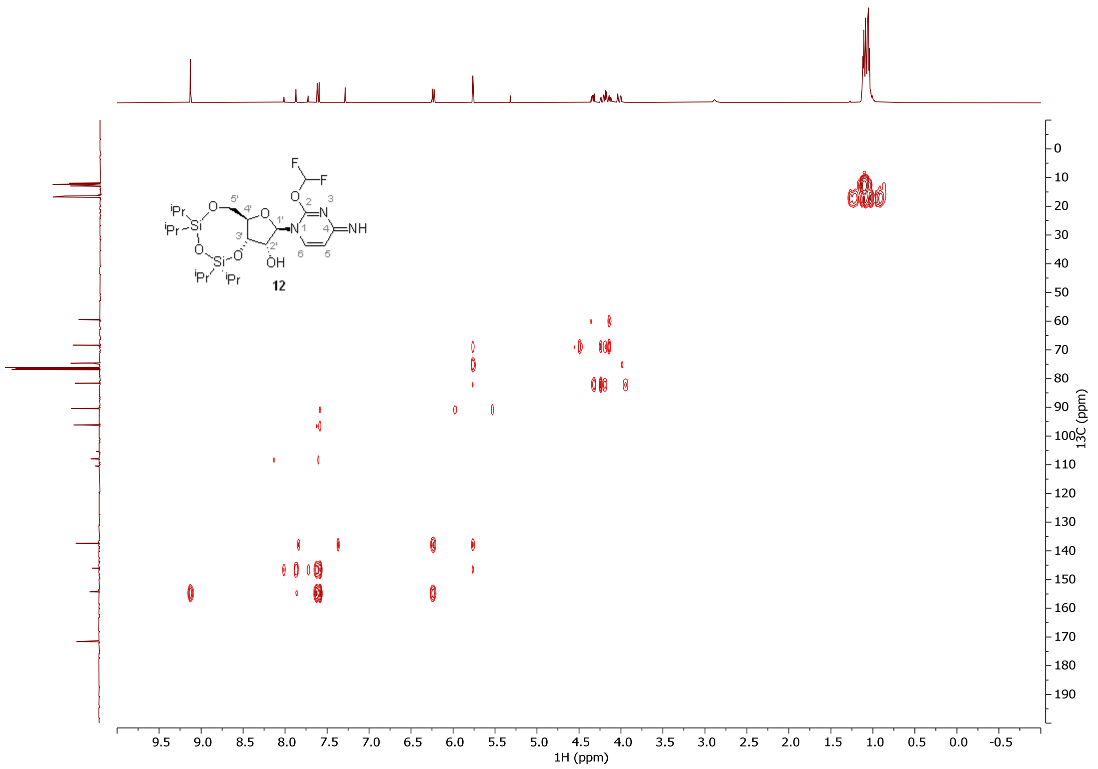

Figure S23 - ^{1}H-^{13}C HMBC NMR spectrum of **12** in $CDCl_3$

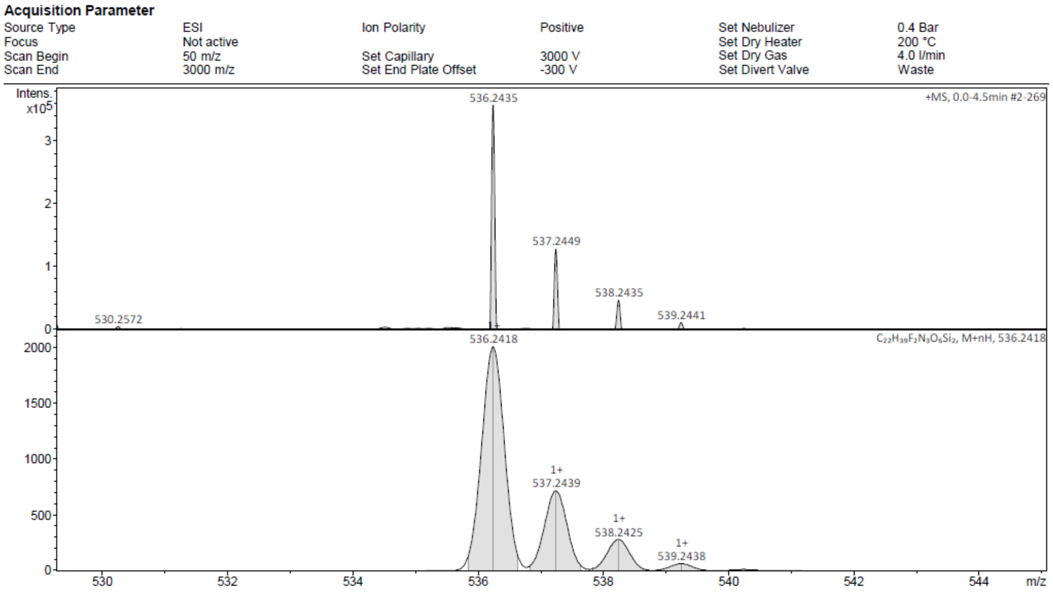

Figure S24 - HRMS spectrum of **12**

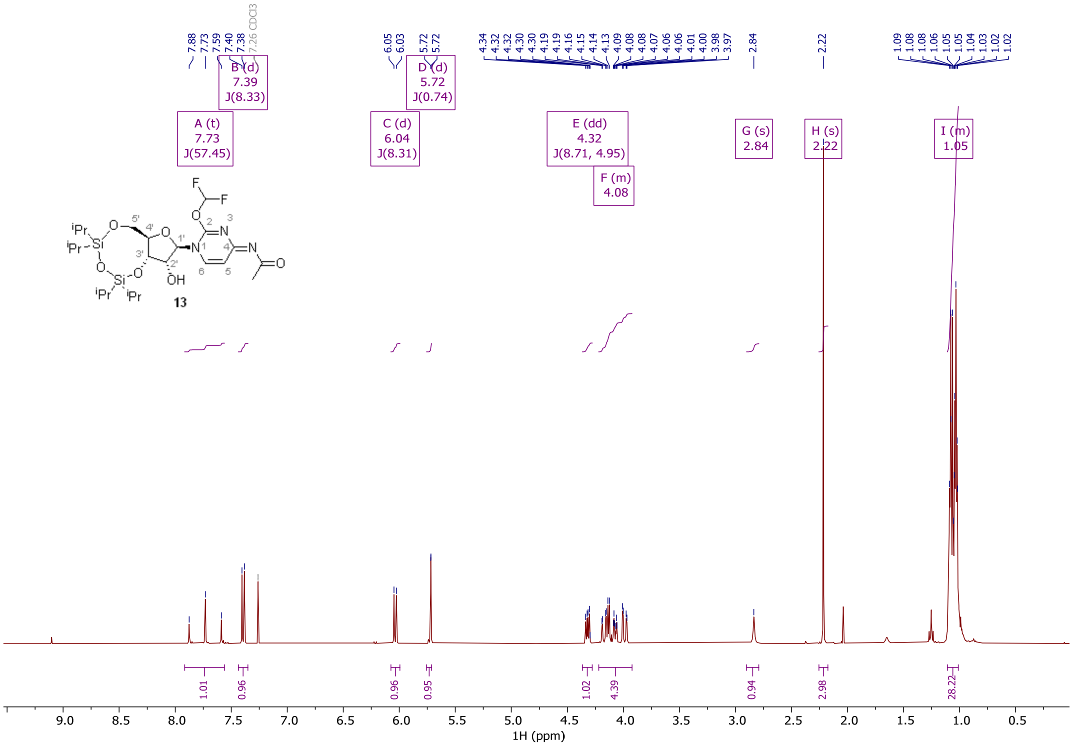

Figure S25 - ^{1}H NMR (400 MHz) spectrum of **13** in $CDCl_3$

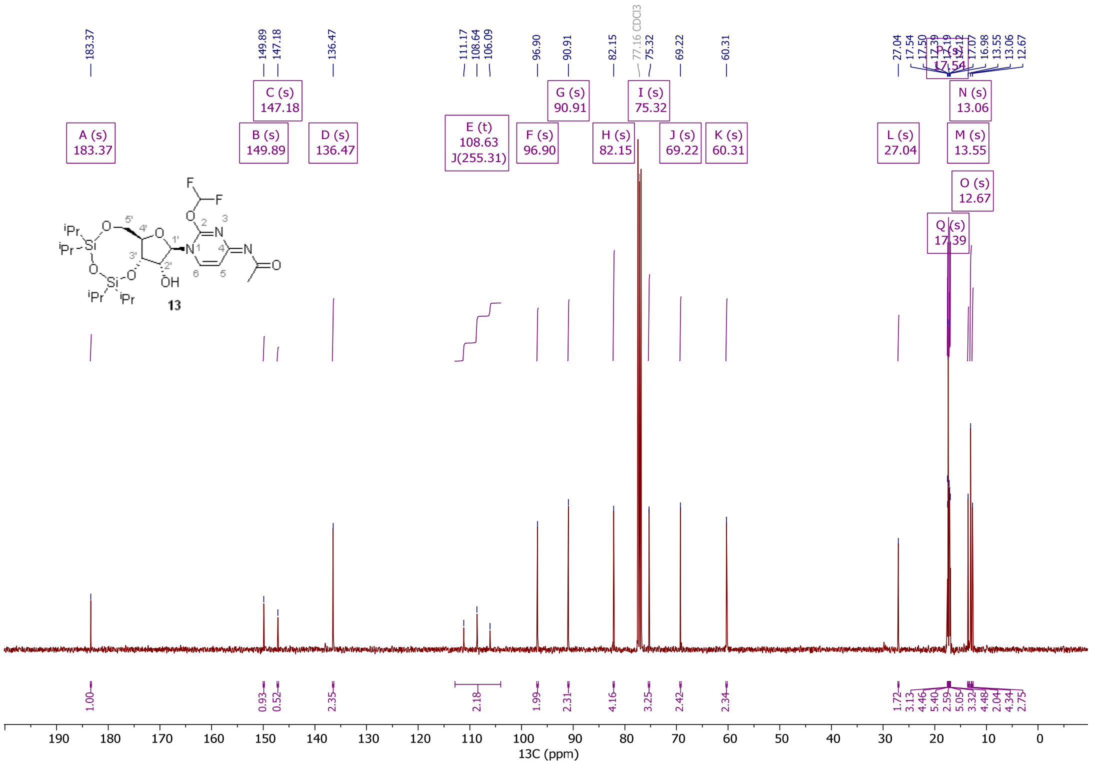

Figure S26 - ^{13}C NMR (101 MHz) spectrum of **13** in $CDCl_3$

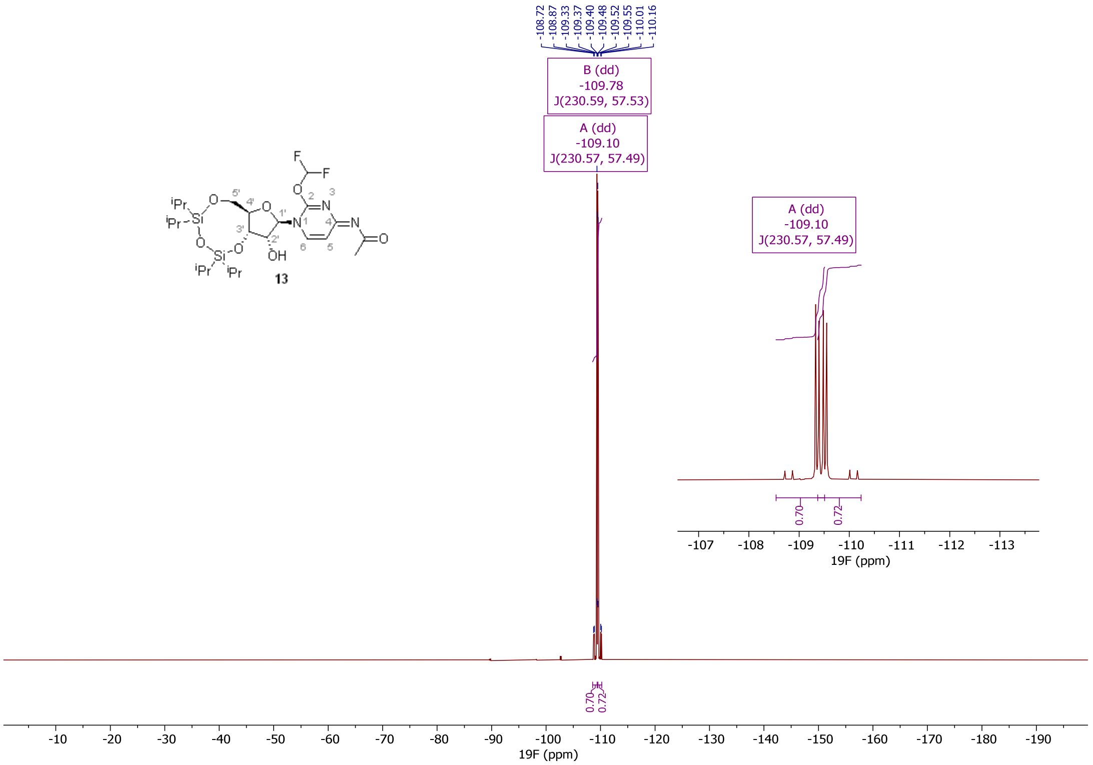

Figure S27 - ^{19}F NMR (376 MHz) spectrum of **13** in $CDCl_3$

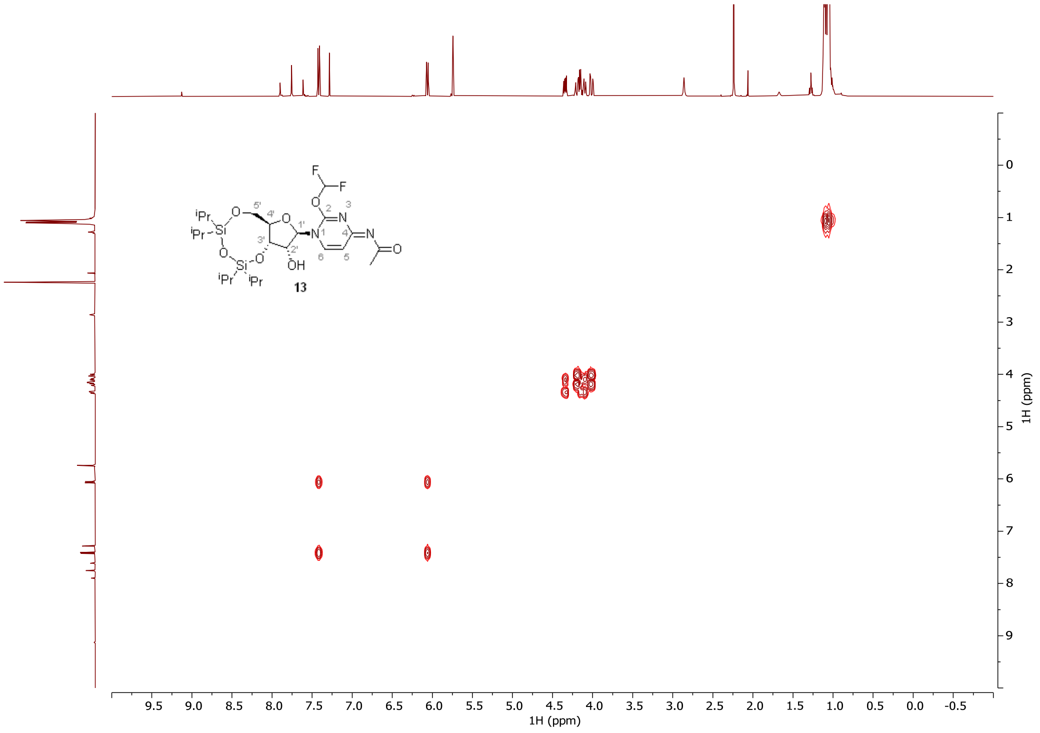

Figure S28 - ^{1}H-^{1}H COSY spectrum of **13** in $CDCl_3$

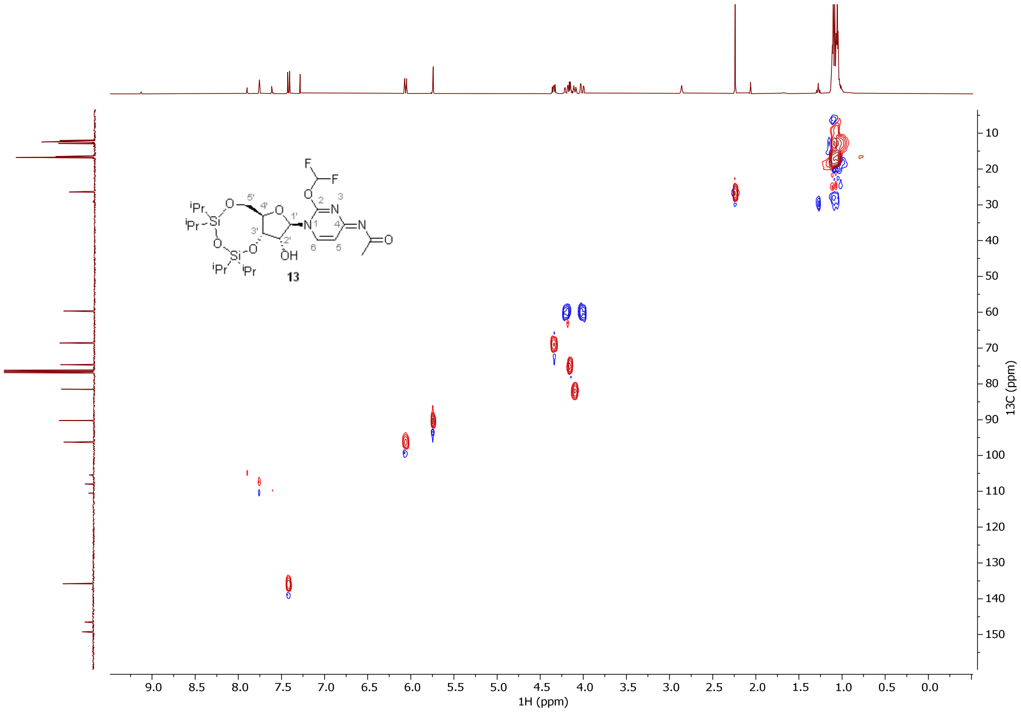

Figure S29 - ^{1}H-^{13}C HSQC NMR spectrum of **13** in $CDCl_3$

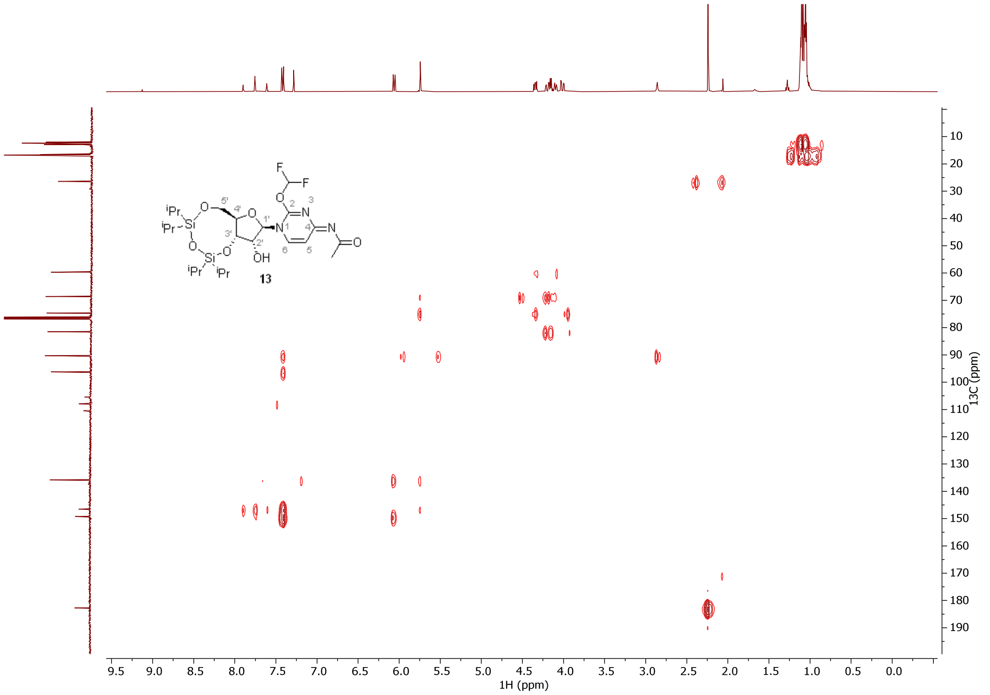

Figure S30 - ^{1}H-^{13}C HMBC NMR spectrum of **13** in $CDCl_3$

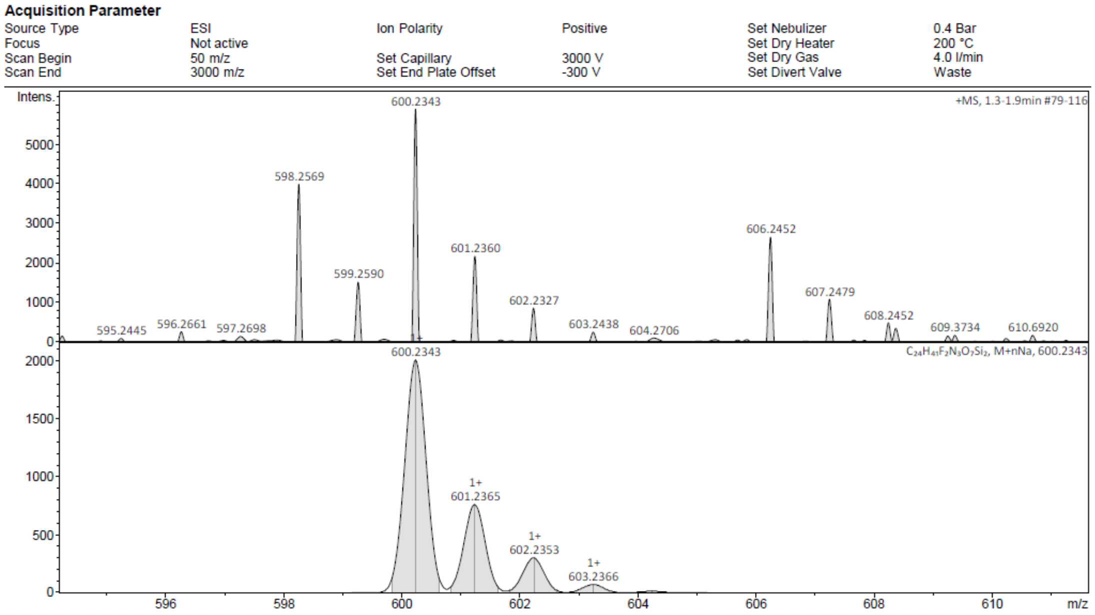

Figure S31 – HRMS spectrum of **13**

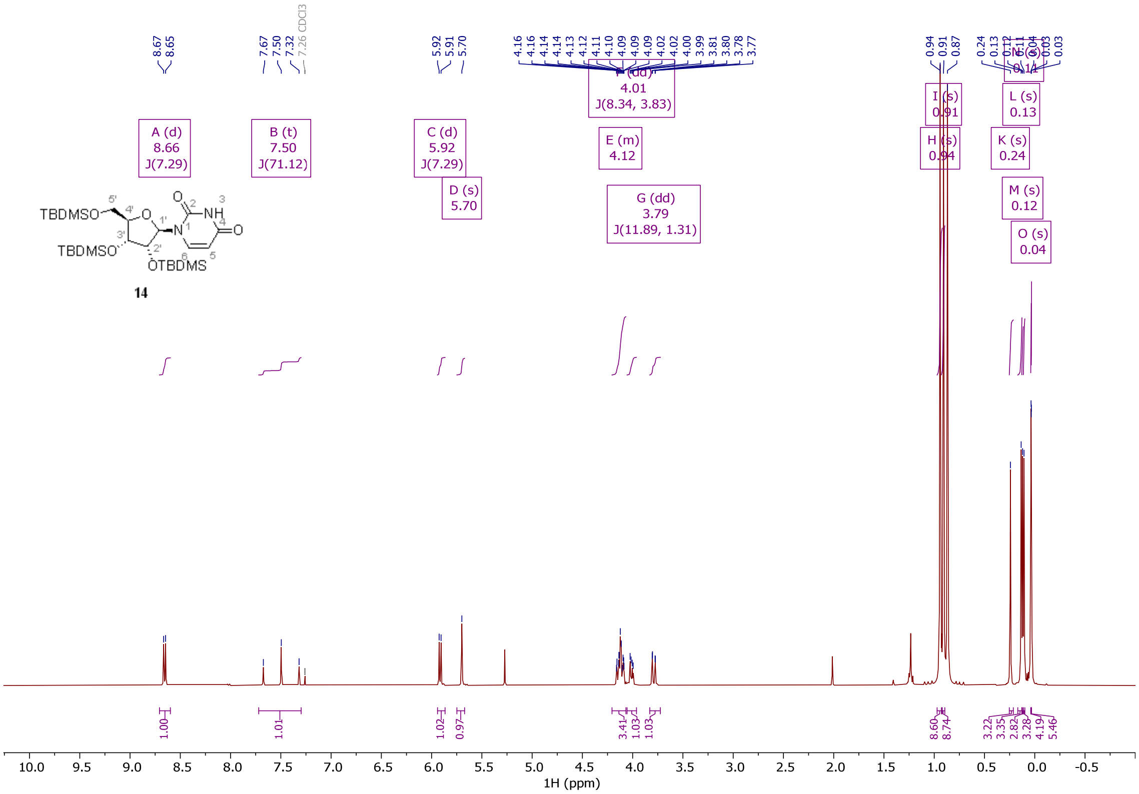

Figure S32 - ^{1}H NMR (400 MHz) spectrum of **14** in $CDCl_3$

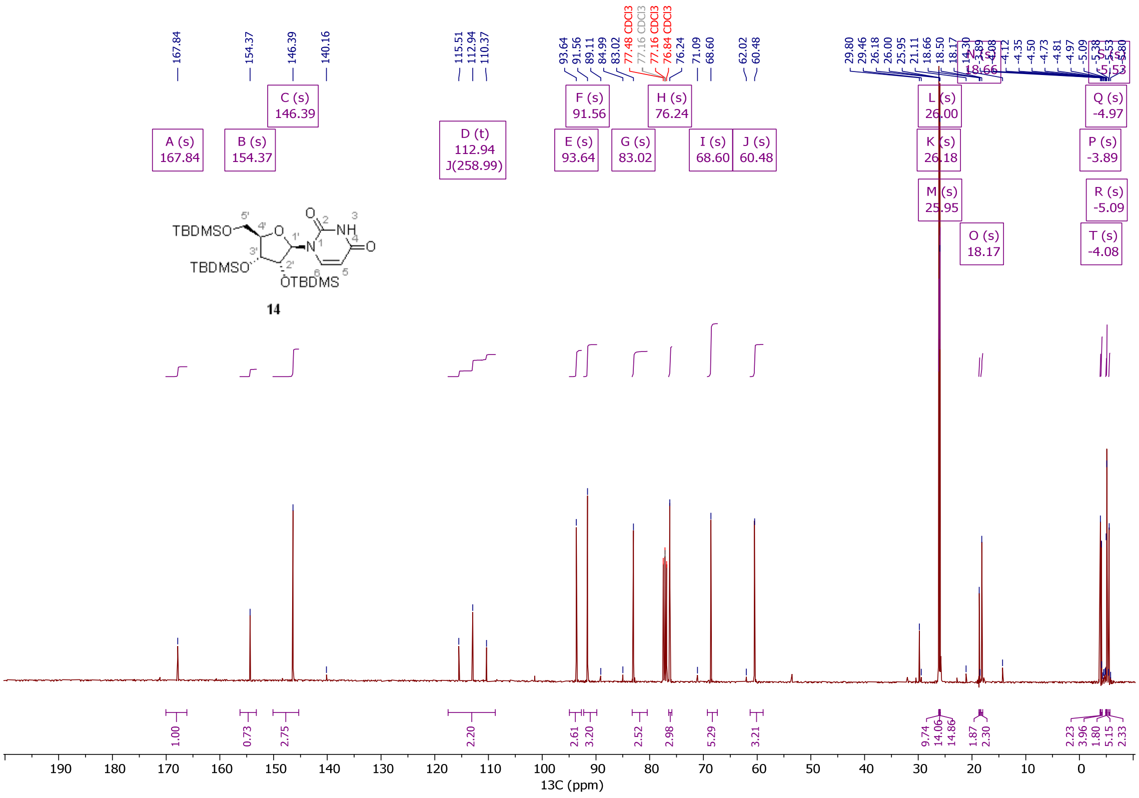

Figure S33 - ^{13}C NMR (101 MHz) spectrum of **14** in $CDCl_3$

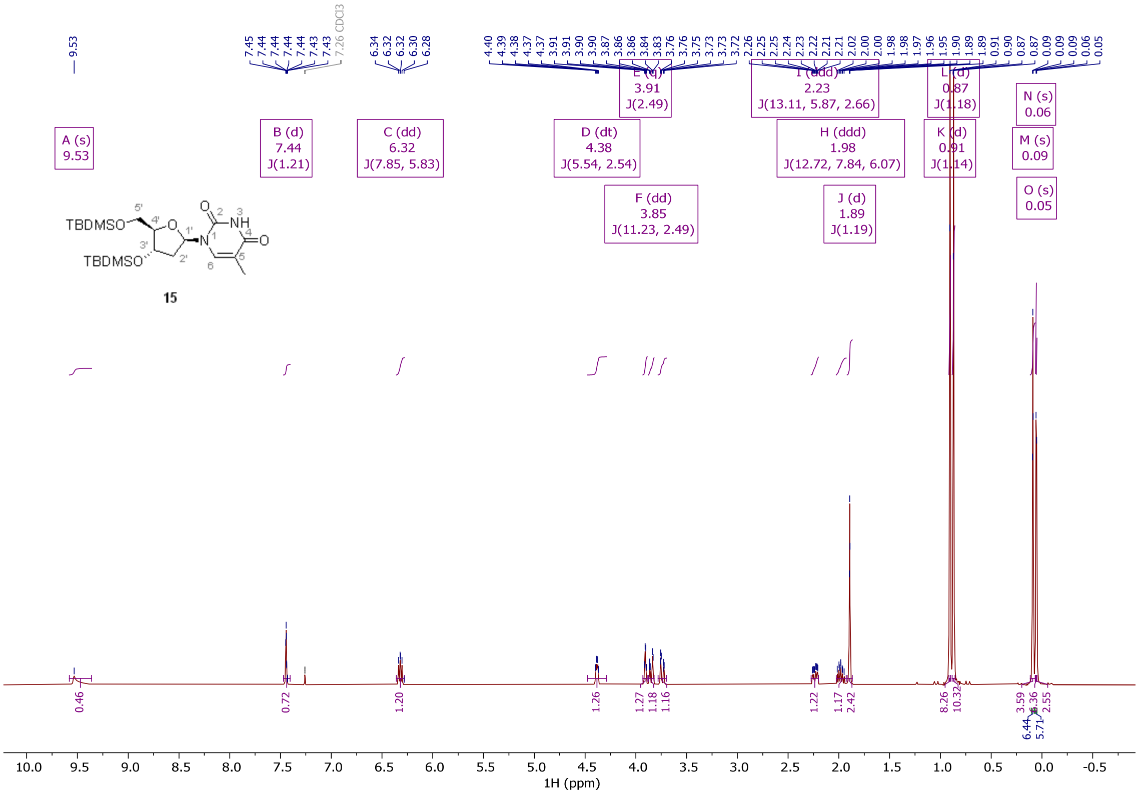

Figure S34 - ^{1}H NMR (400 MHz) spectrum of **15** in $CDCl_3$

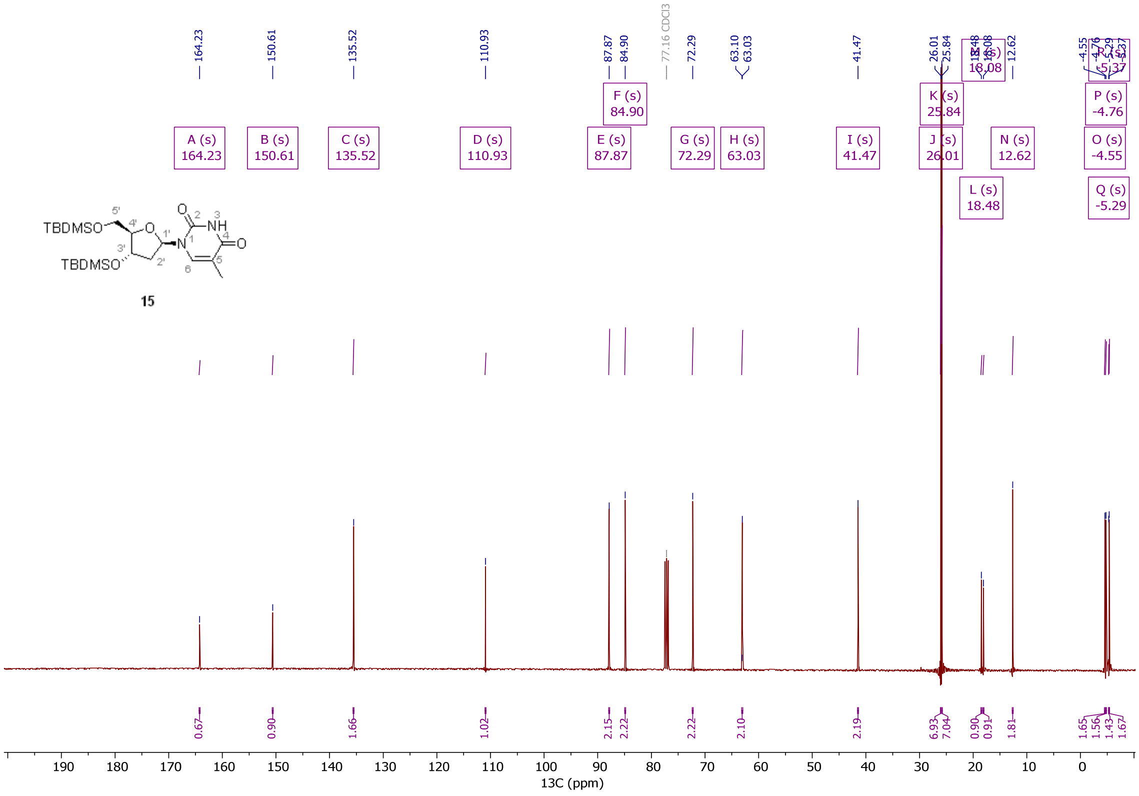

Figure S35 - ^{13}C NMR (101 MHz) spectrum of **15** in $CDCl_3$

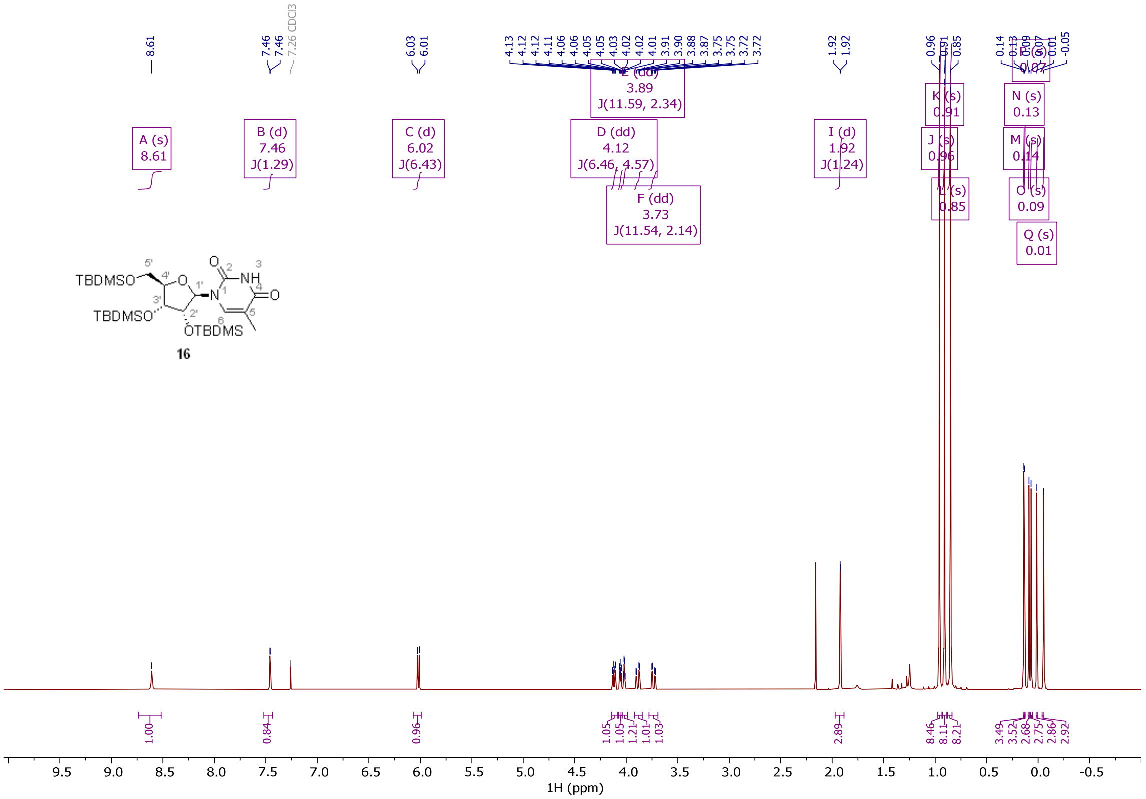

Figure S36 - ^{1}H NMR (400 MHz) spectrum of **16** in $CDCl_3$

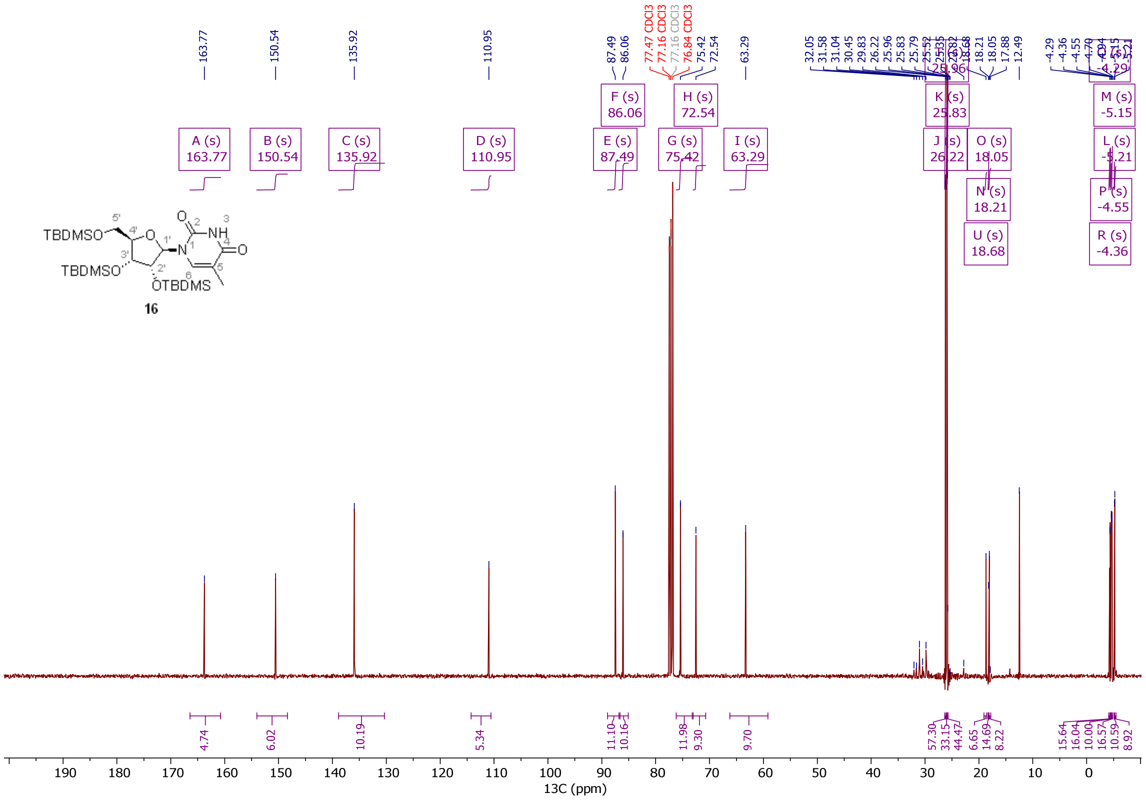

Figure S37 - ^{13}C NMR (101 MHz) spectrum of **16** in $CDCl_3$

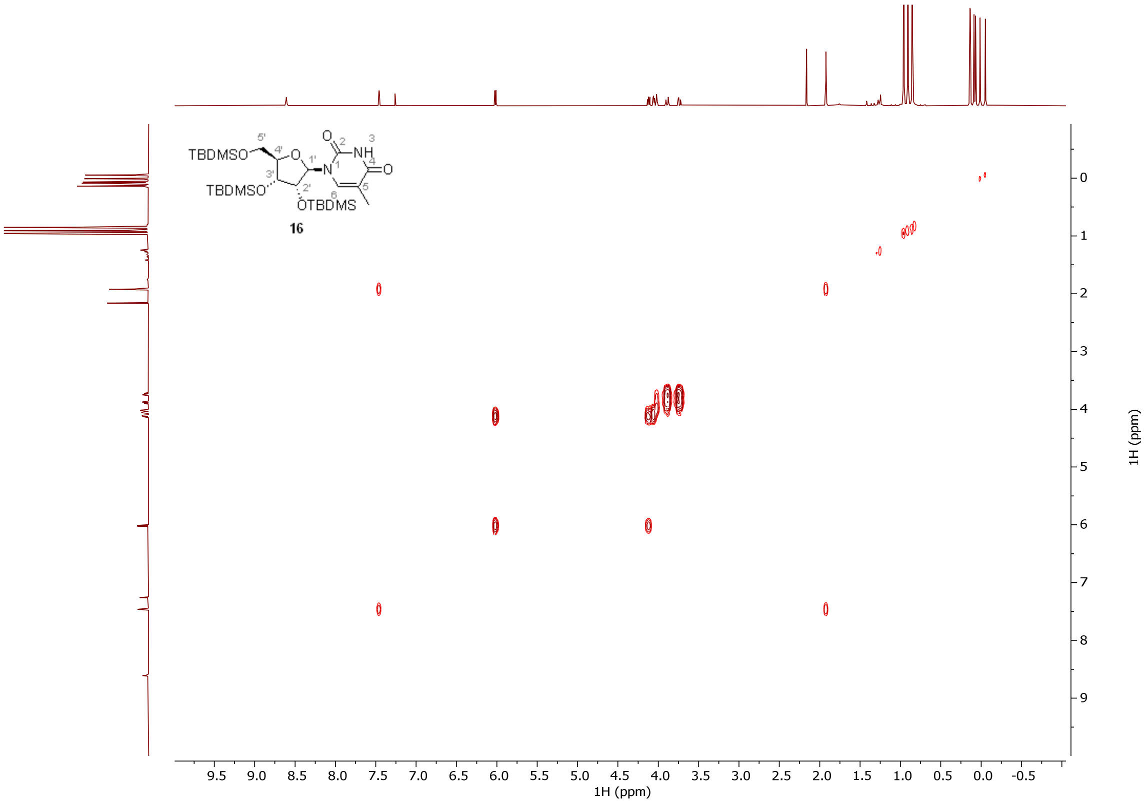

Figure S38 - ^{1}H-^{1}H COSY NMR spectrum of **16** in $CDCl_3$

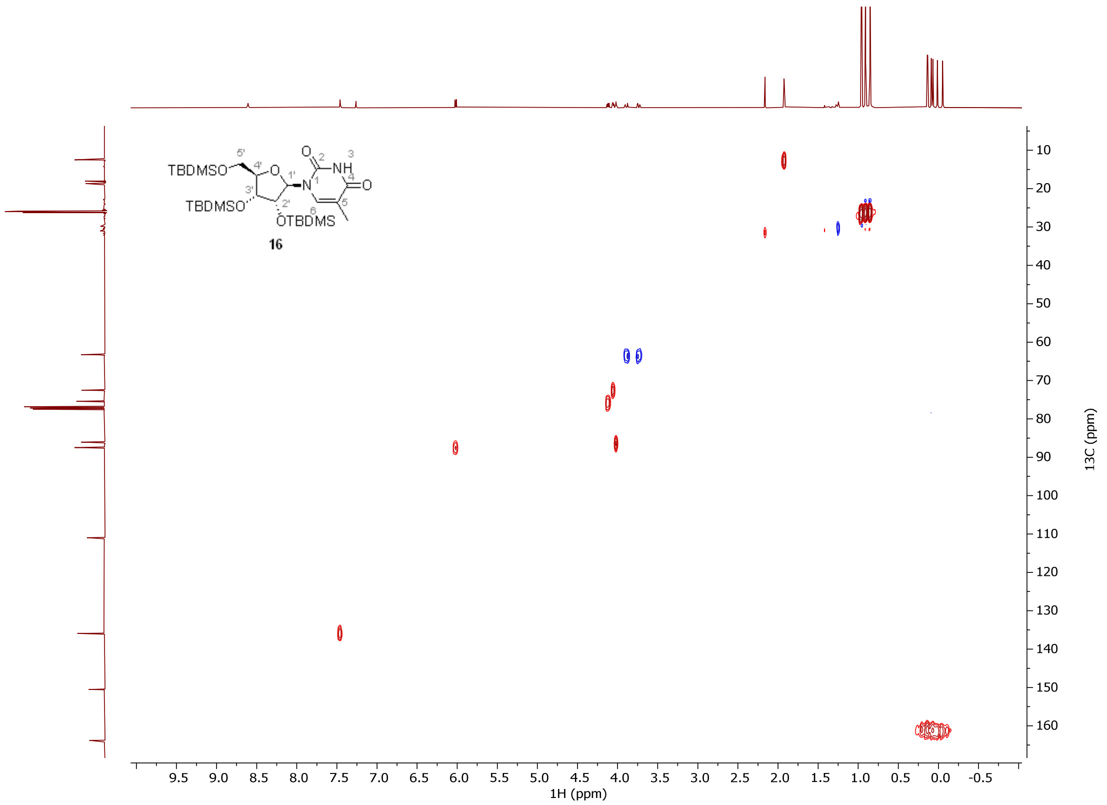

Figure S39 - ^{1}H-^{13}C HSQC NMR spectrum of **16** in $CDCl_3$

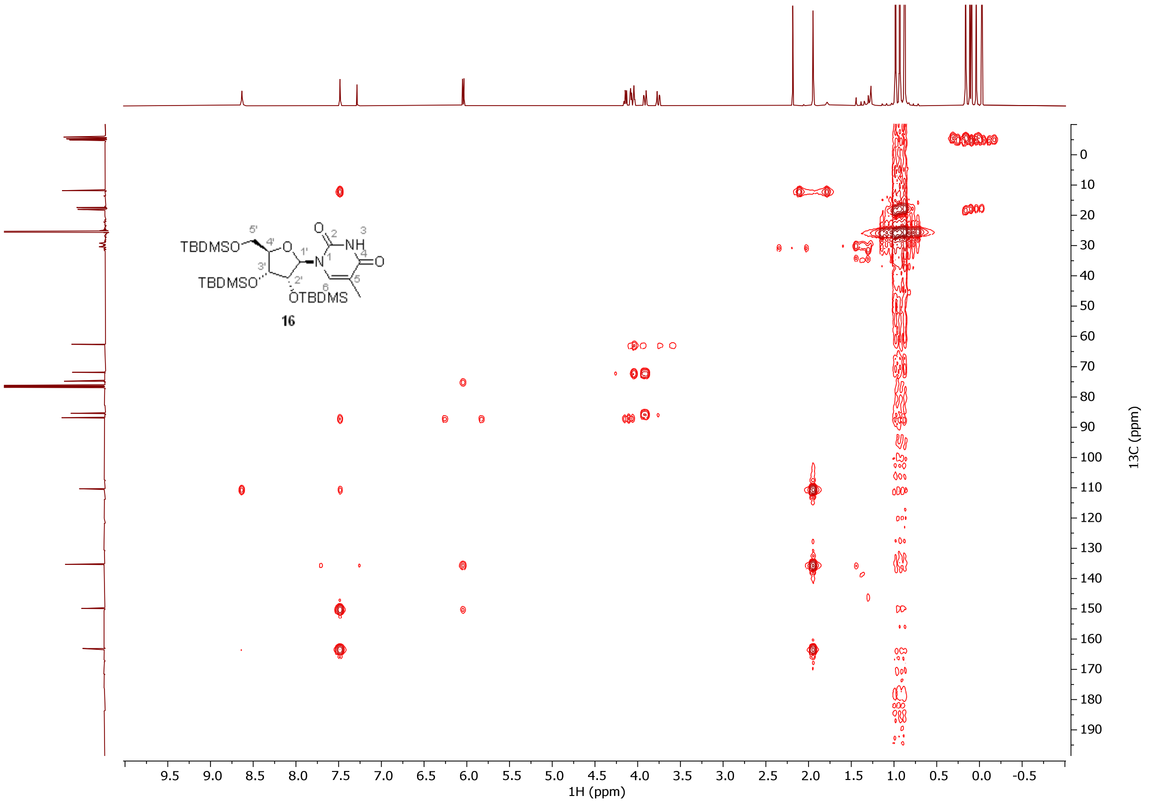

Figure S40 - ^{1}H-^{13}C HMBC NMR spectrum of **16** in $CDCl_3$

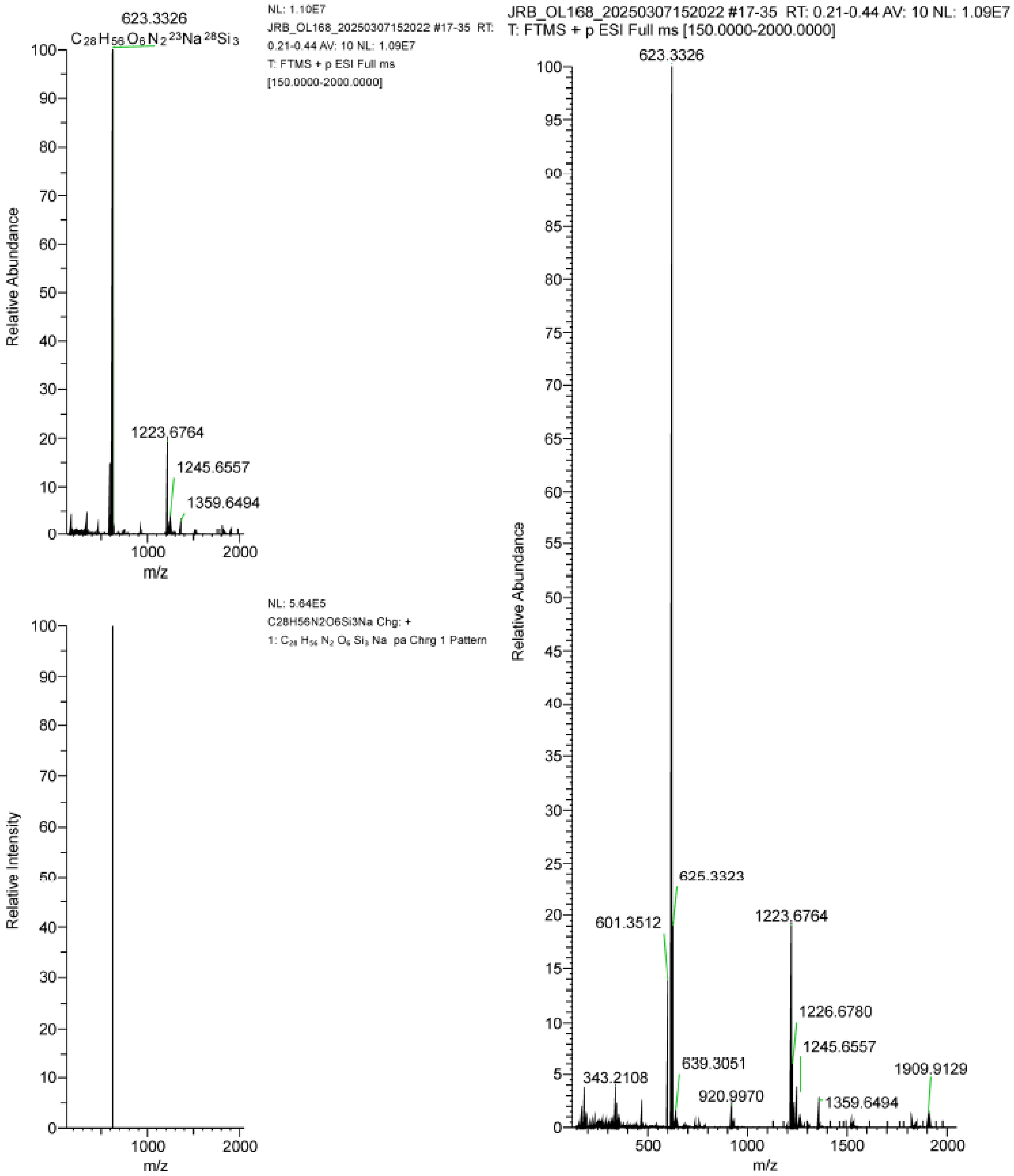

Peak Mass	Display...	S Fit	RDB	Delta [p...	Theo. m...	Rank	Combin...	# Match...	# Misse...	MS Cov....	Pattern...	MSMS...
623.3326	$C_{28}H_{56}O_6N_2{}^{23}Na^{28}Si_3$	54.742900237150 2	4.50	-1.94	623.33384	1	97.46	6	0	99.83	100	(Collection)

Figure S41 - HRMS spectrum of **16**

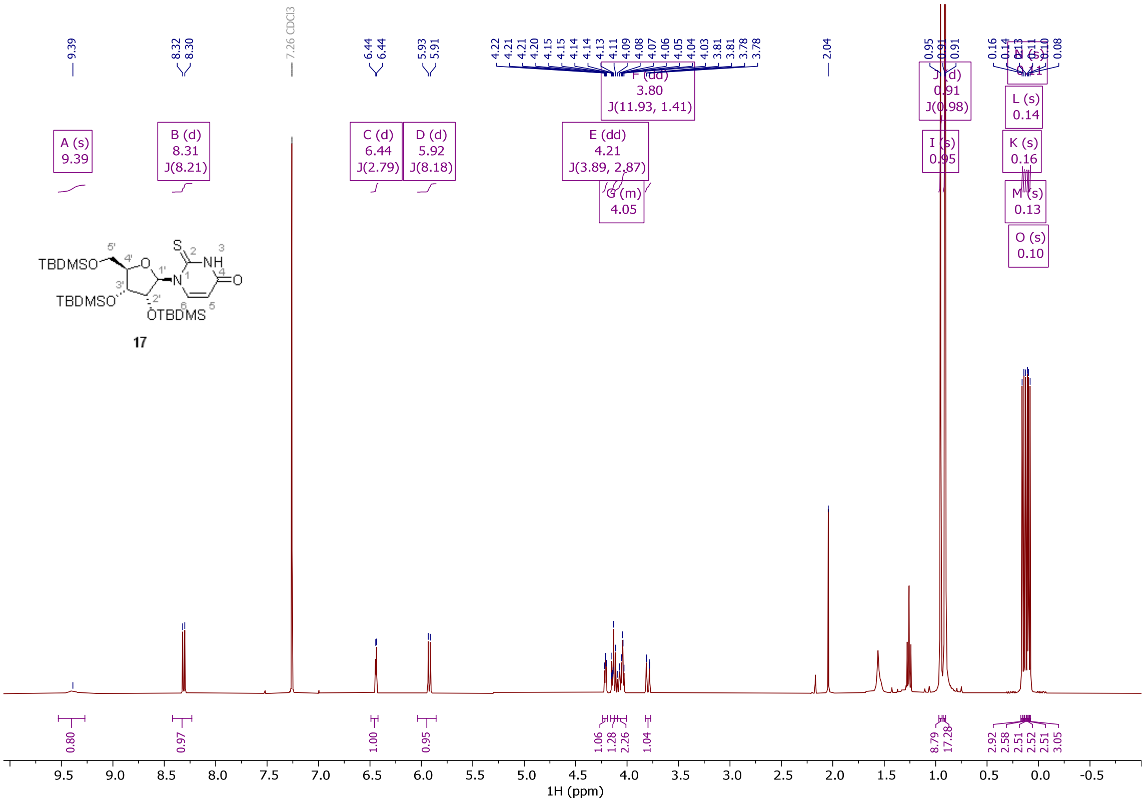

Figure S42 - ^{1}H NMR (400 MHz) spectrum of **17** in $CDCl_3$

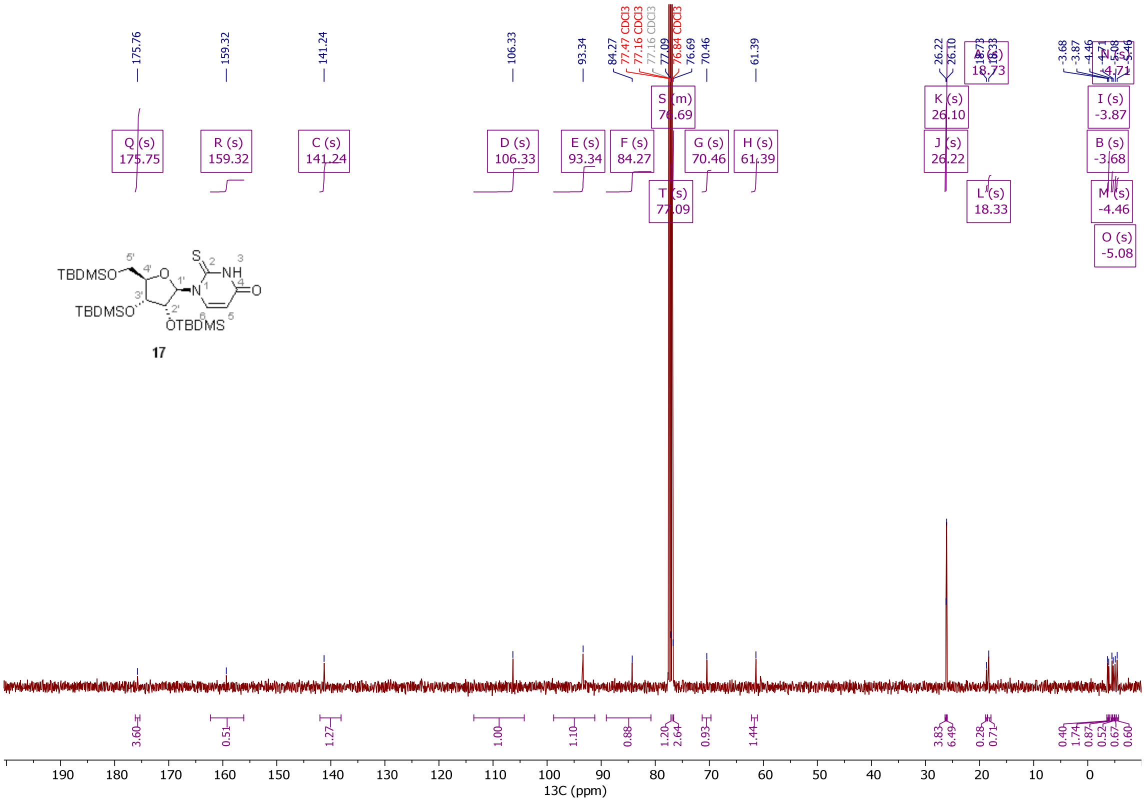

Figure S43 - ^{13}C NMR (101 MHz) spectrum of **17** in $CDCl_3$

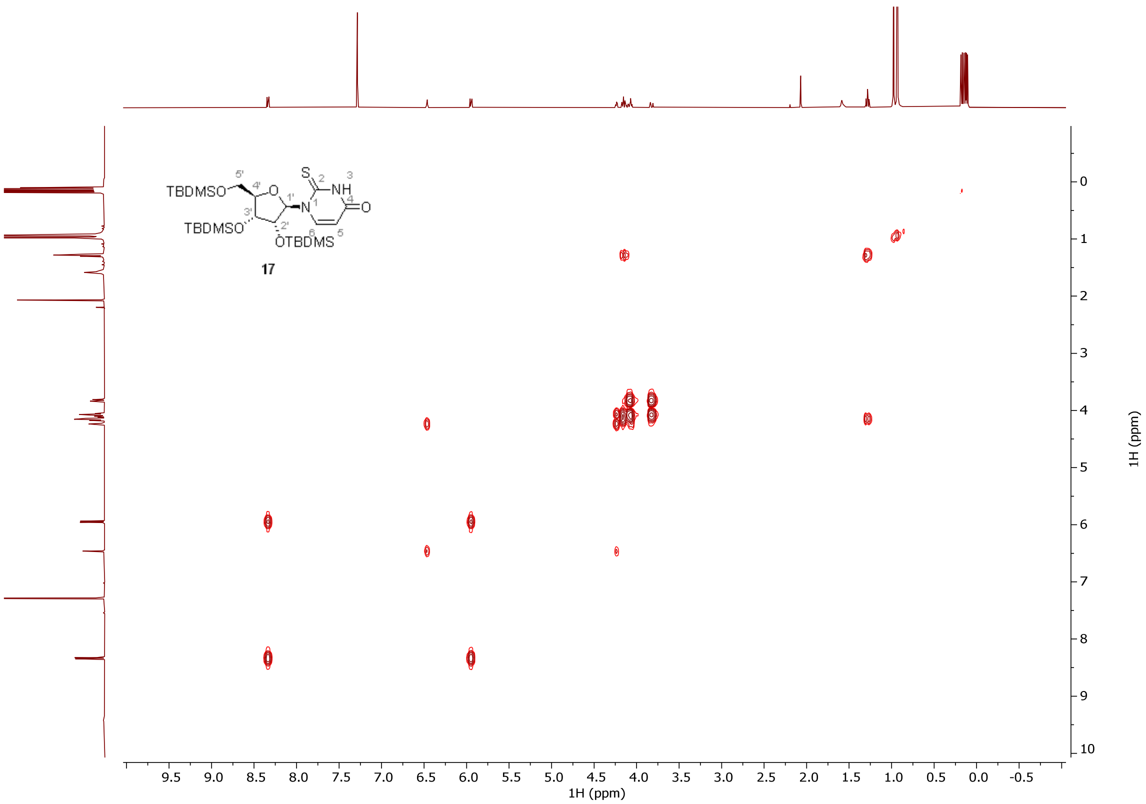

Figure S44 - ¹H-¹H COSY NMR spectrum of **17** in $CDCl_3$

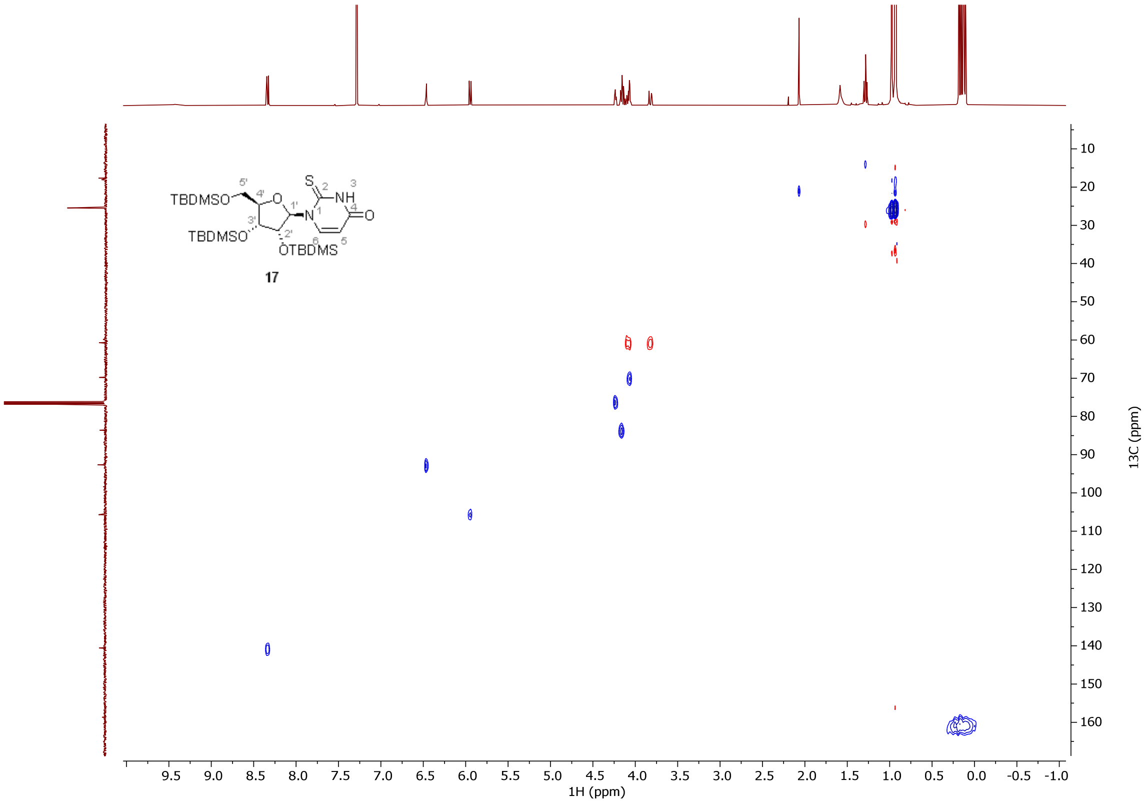

Figure S45 - ¹H-¹³C HSQC NMR spectrum of **17** in $CDCl_3$

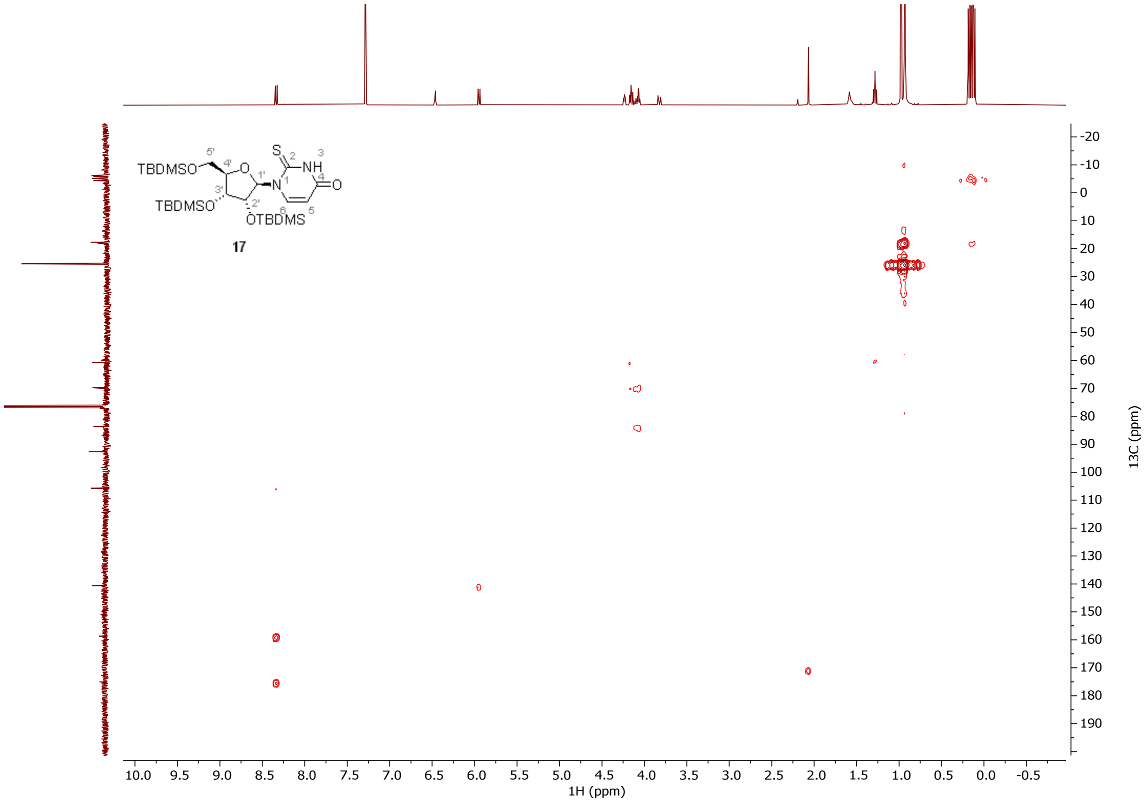

Figure S46 - ^{1}H-^{13}C HMBC NMR spectrum of **17** in $CDCl_3$

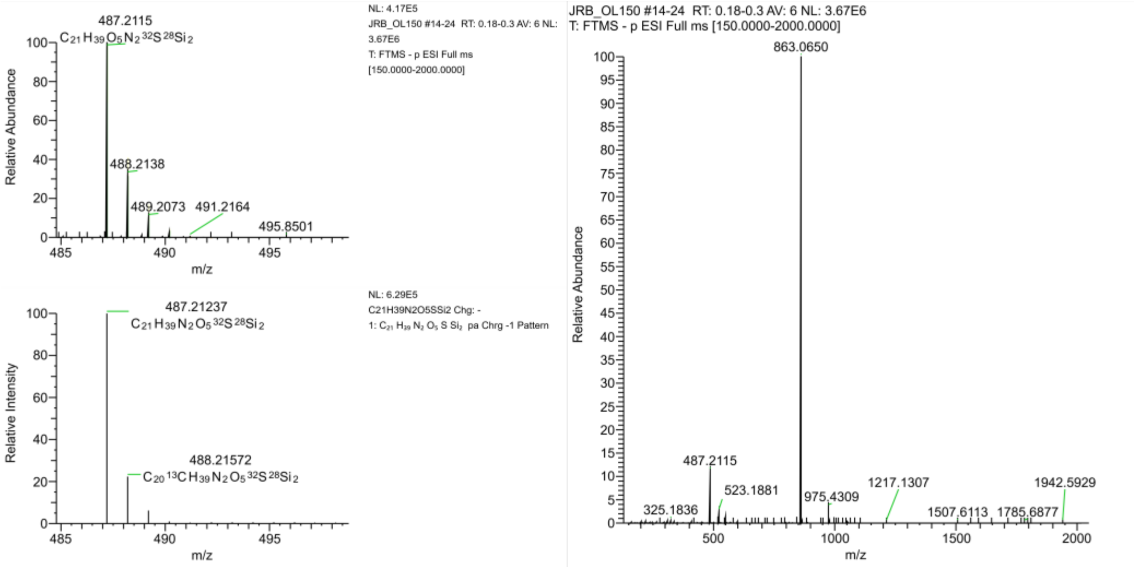

Peak Mass	Display For...	S Fit	RDB	Delta [ppm]	Theo. mass	Rank	Combined S...	# Matched I...	# Missed Iso.	MS Cov. [%]	Pattern Cov....	MSMS Matc...
487.2115	$C_{21}H_{39}O_5N_2{}^{32}S^{28}Si_2$	32.5124105595572	5.50	0.51	487.21127	1	94.14	6	0	97.56	99.47	(Collection)
487.2115	$C_{20}H_{39}O_6N_2{}^{28}Si_3$	24.4917767600345	5.50	0.98	487.21104	2	87.22	6	0	90.7	90.79	(Collection)
487.2115	$C_{24}H_{33}O_6N_3{}^{28}Si$	13.4935601928189	11.00	-3.68	487.21331	3	84.85	2	3	88.81	94.4	(Collection)
487.2115	$C_{23}H_{37}O_3N_3{}^{28}Si_3$	10.439947503237	10.00	-4.52	487.21372	4	84.69	6	0	88.81	88.34	(Collection)
487.2115	$C_{25}H_{33}O_5N_3{}^{32}S$	5.61546213321796	11.00	-4.15	487.21354	5	63.85	5	1	67.09	71.23	(Collection)
487.2115	$C_{24}H_{37}O_2N_3{}^{32}S^{28}Si_2$	4.26803952484449	10.00	-4.99	487.21395	6	63.78	6	0	67.09	65.39	(Collection)

Figure S47 - HRMS spectrum of **17**

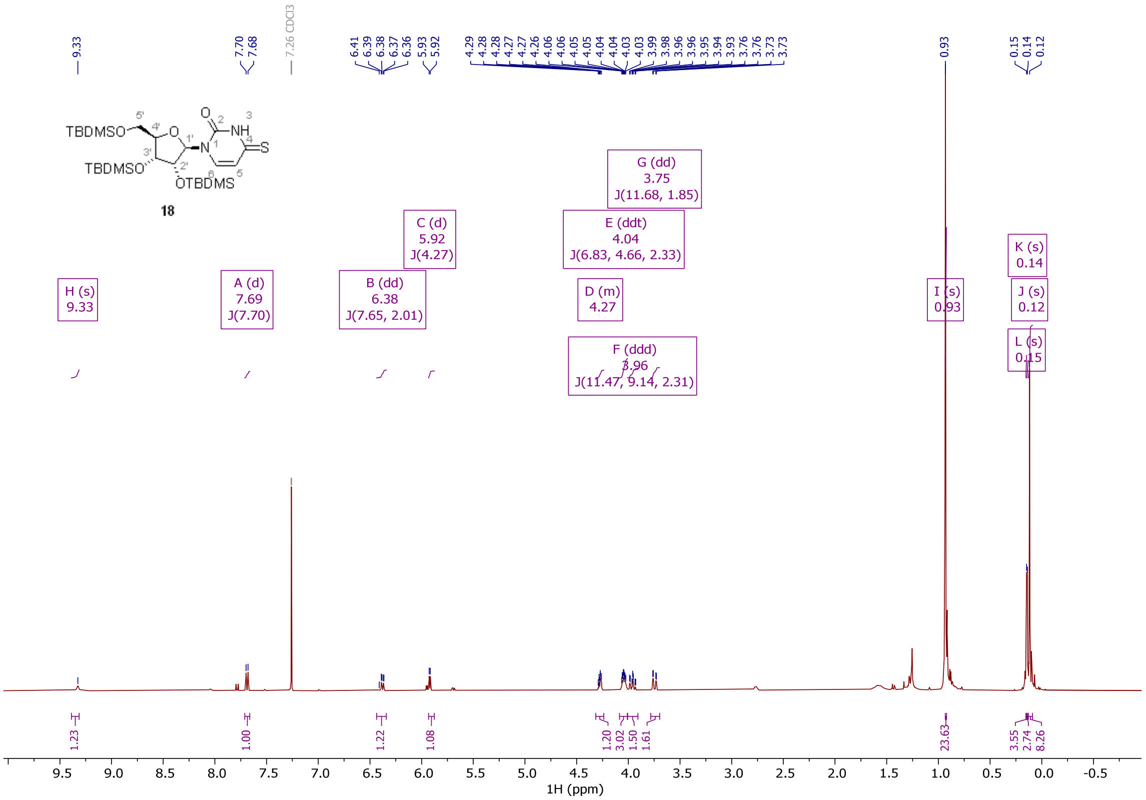

Figure S48 - ^{1}H NMR (400 MHz) spectrum of **18** in $CDCl_3$

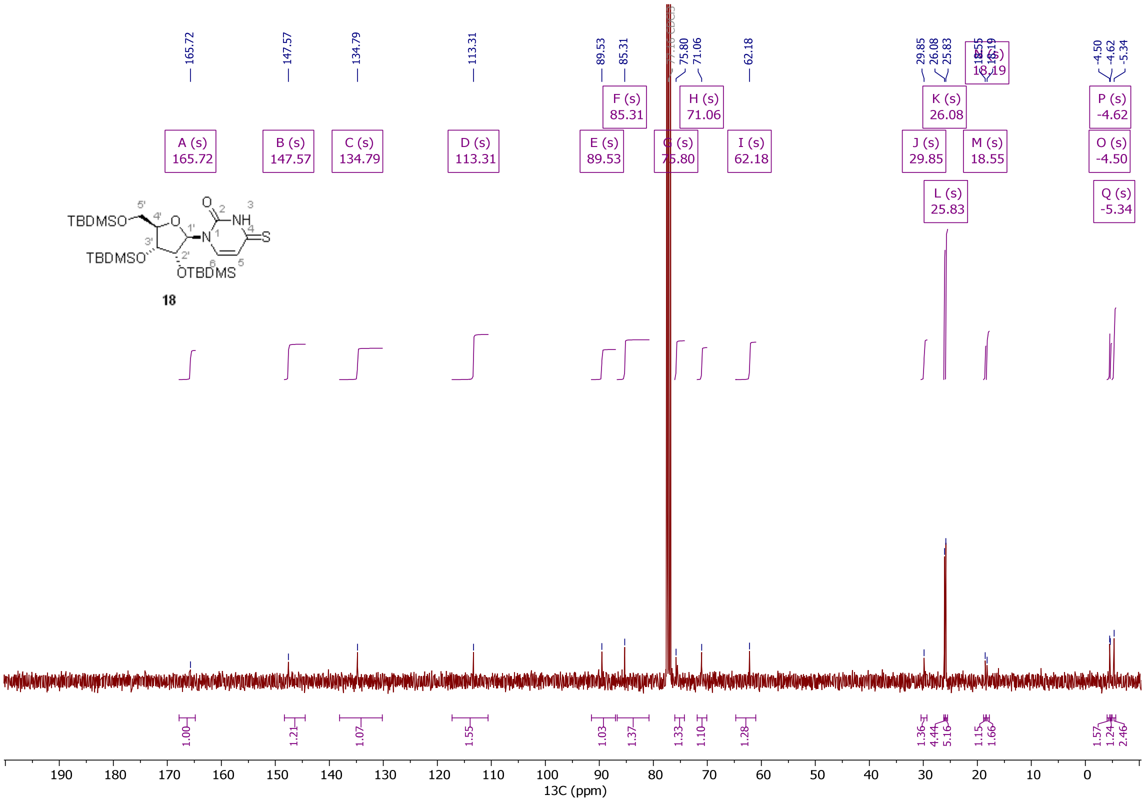

Figure S49 - ^{13}C NMR (101 MHz) spectrum of **18** in $CDCl_3$

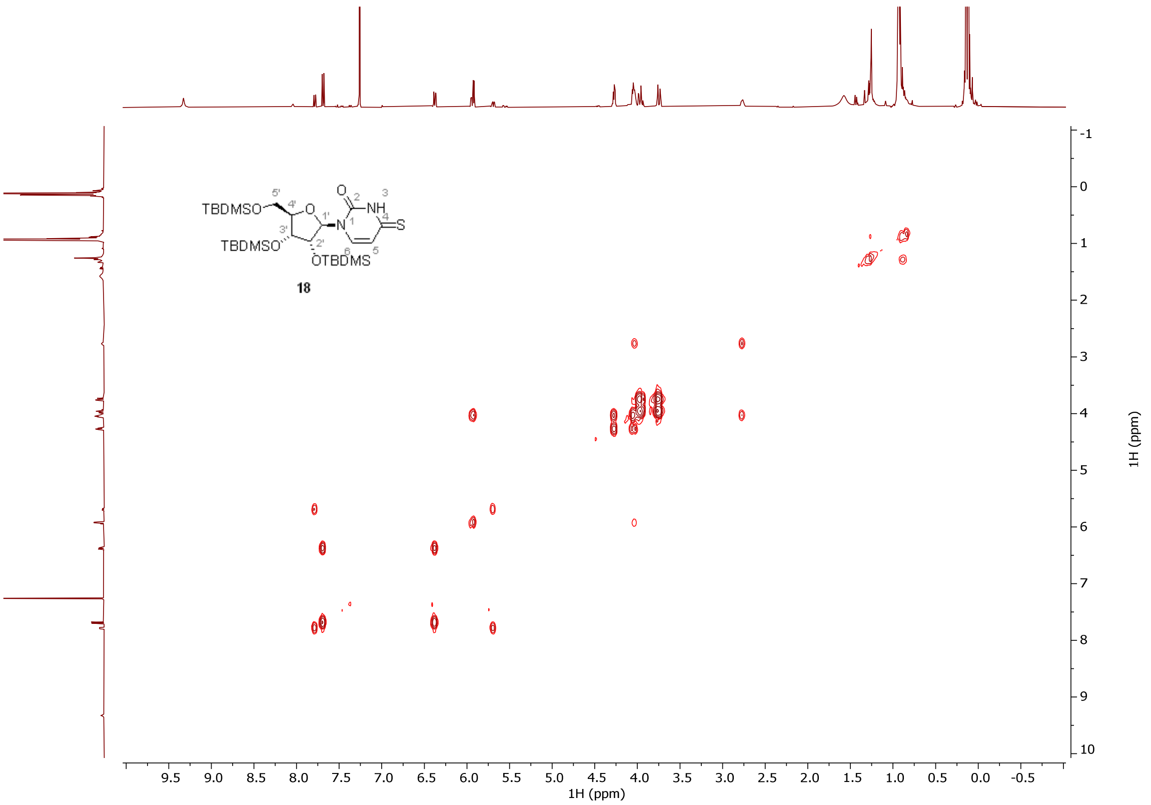

Figure S50 - ^{1}H-^{1}H COSY NMR spectrum of **18** in $CDCl_3$

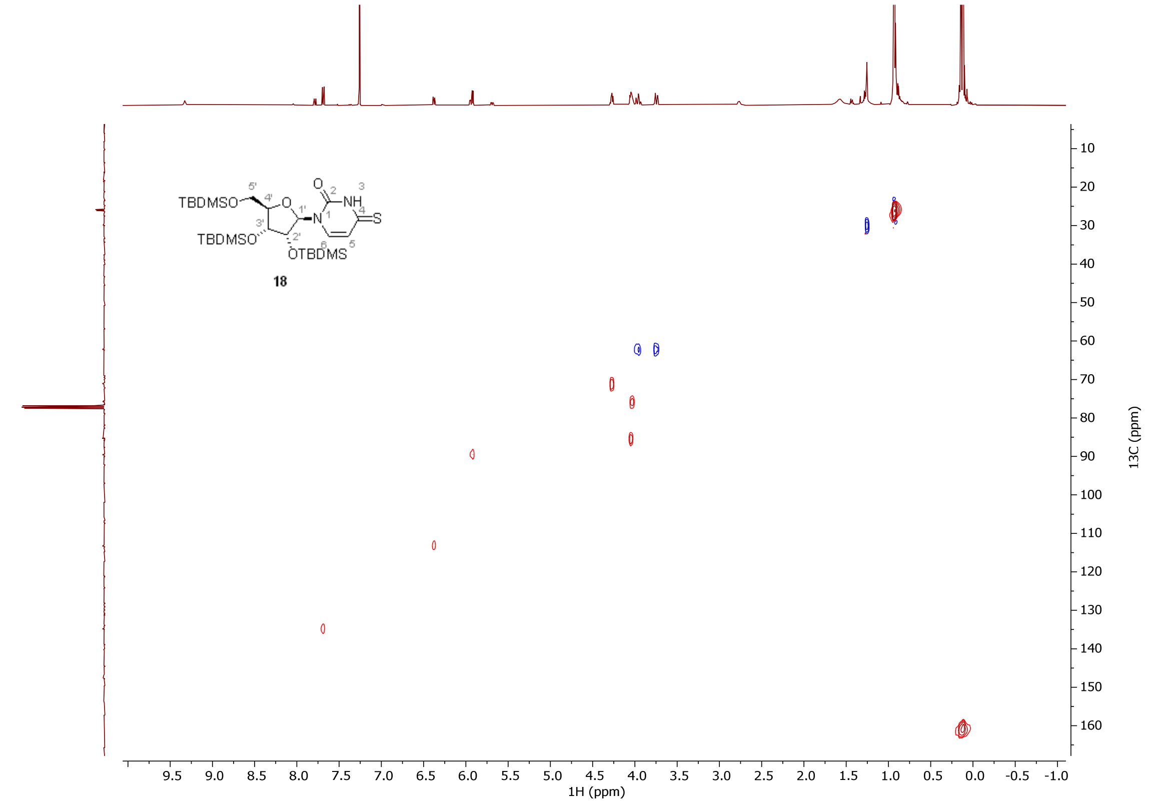

Figure S51 - ^{1}H-^{13}C HSQC NMR spectrum of **18** in $CDCl_3$

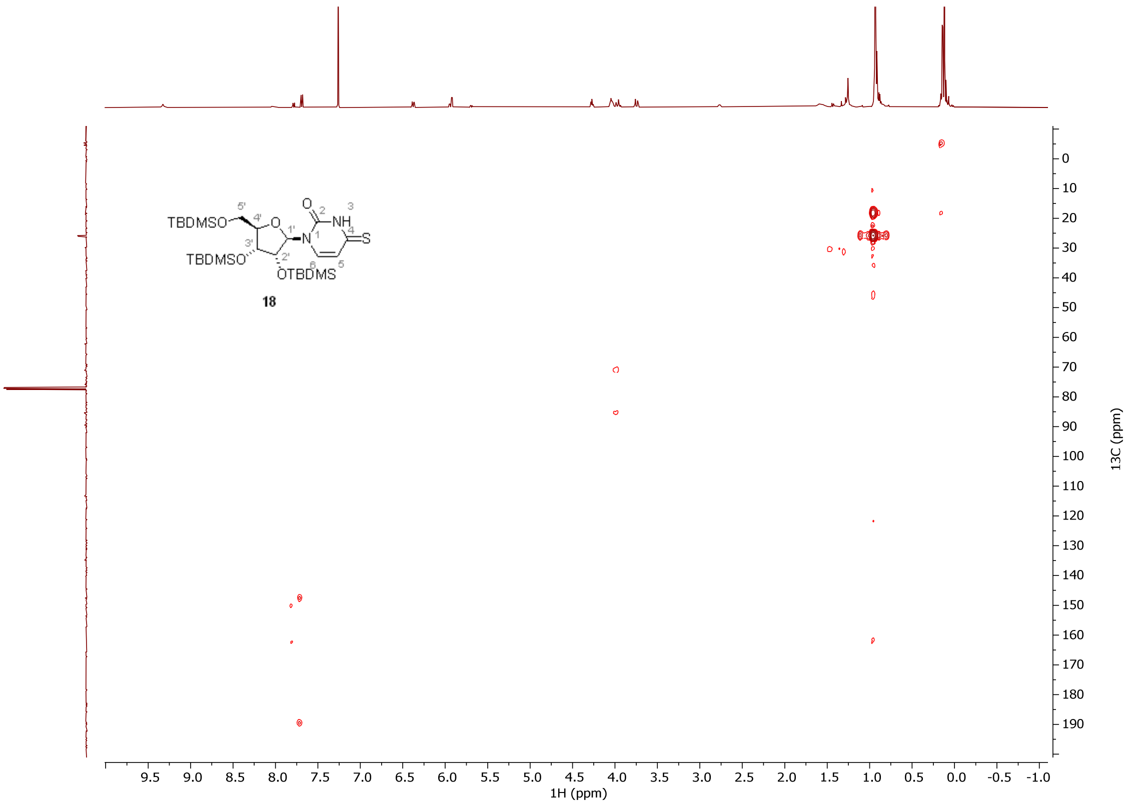

Figure S52 - ^{1}H-^{13}C HMBC NMR spectrum of **18** in $CDCl_3$

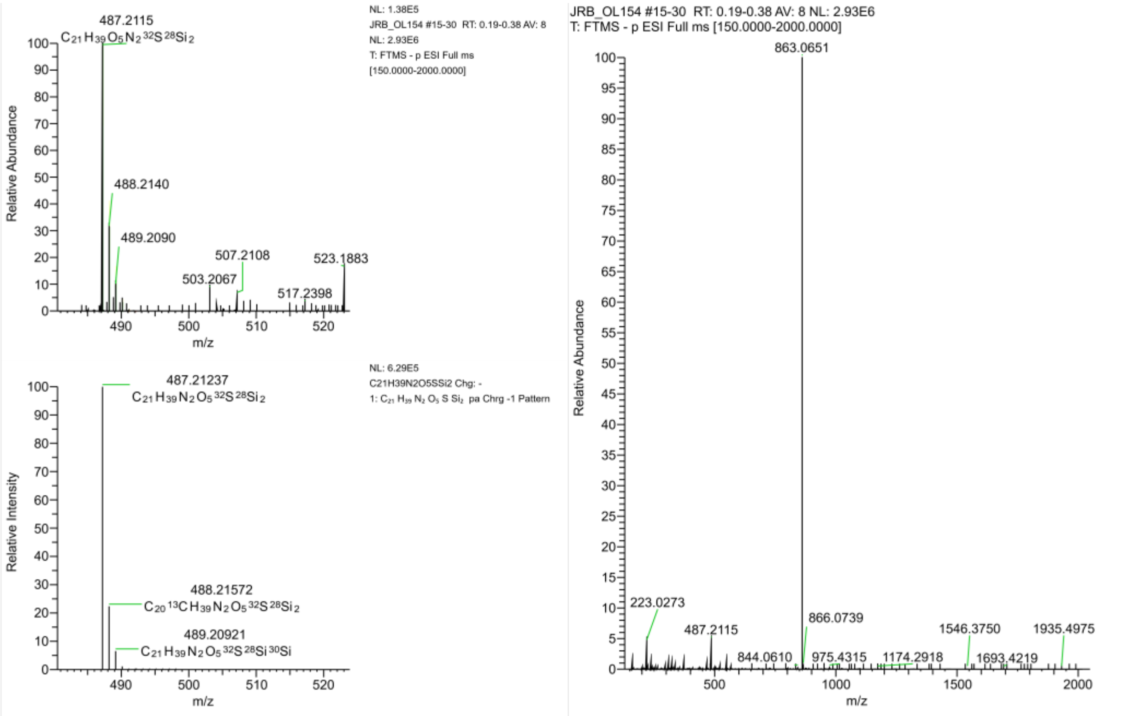

Peak Mass	Display For...	S Fit	RDB	Delta [ppm]	Theo. mass	Rank	Combined S...	# Matched I...	# Missed Iso.	MS Cov. [%]	Pattern Cov....	MSMS Matc...
487.2115	$C_{21}H_{39}O_5N_2{}^{32}S^{28}Si_2$	34.5214108473624	5.50	0.52	487.21127	1	90.28	5	1	93.38	99.48	(Collection)

Figure S53 - HRMS spectrum of **18**

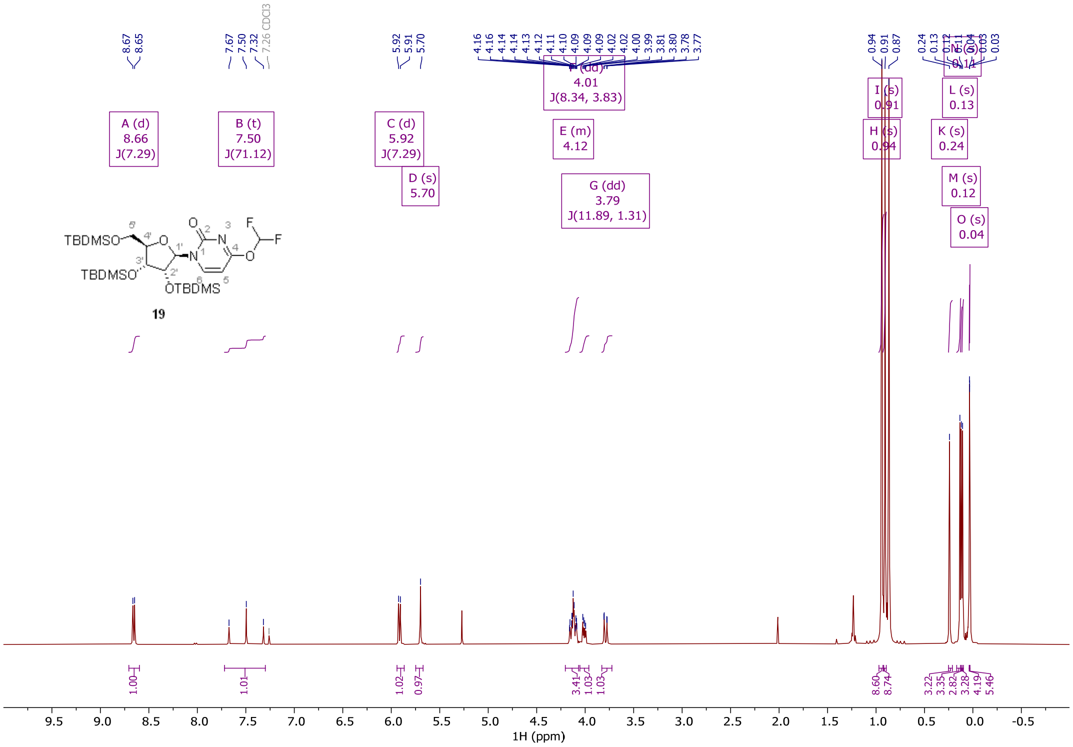

Figure S54 - ^{1}H NMR (400 MHz) spectrum of **19** in $CDCl_3$

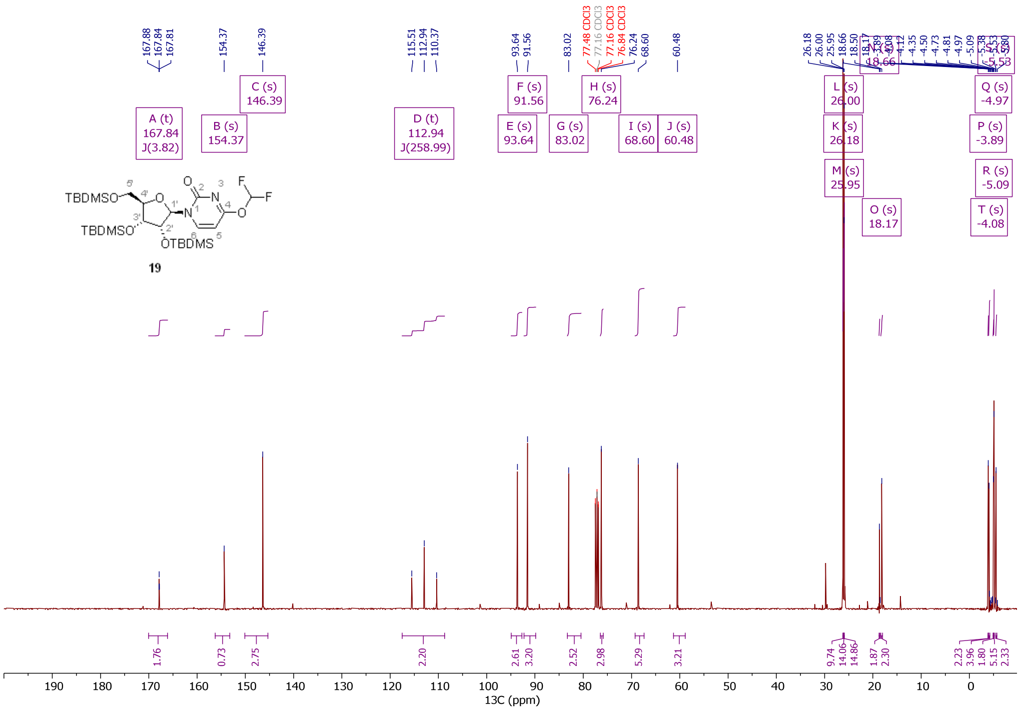

Figure S55 - ^{13}C NMR (101 MHz) spectrum of **19** in $CDCl_3$

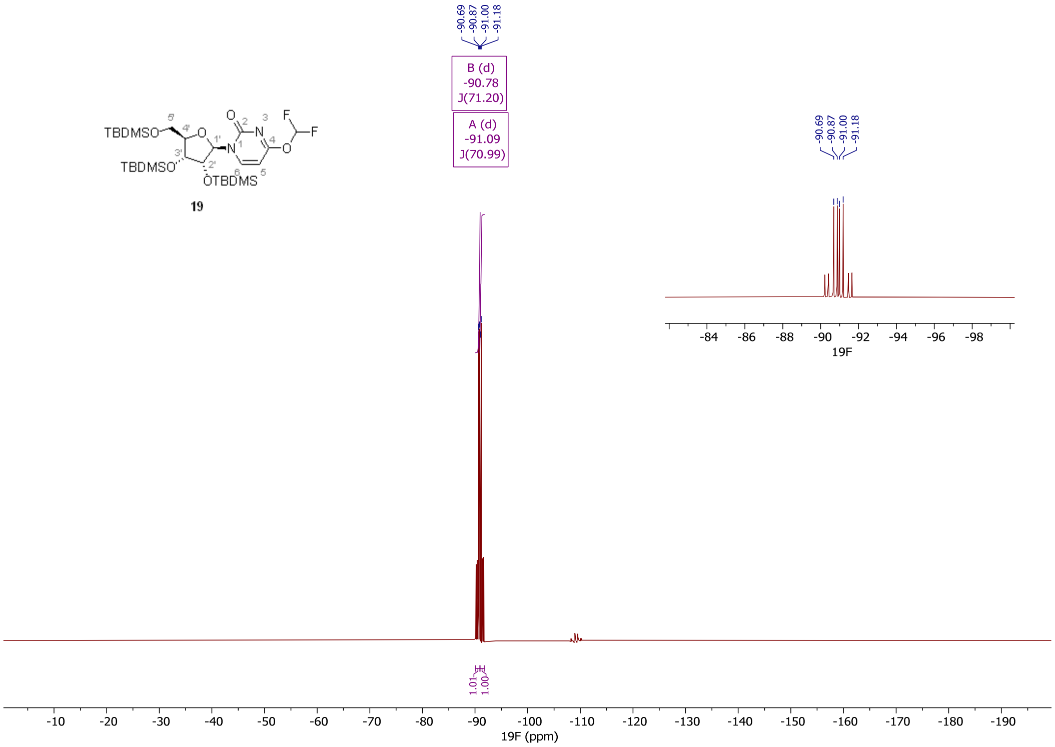

Figure S56 - ^{19}F NMR (376 MHz) spectrum of **19** in $CDCl_3$

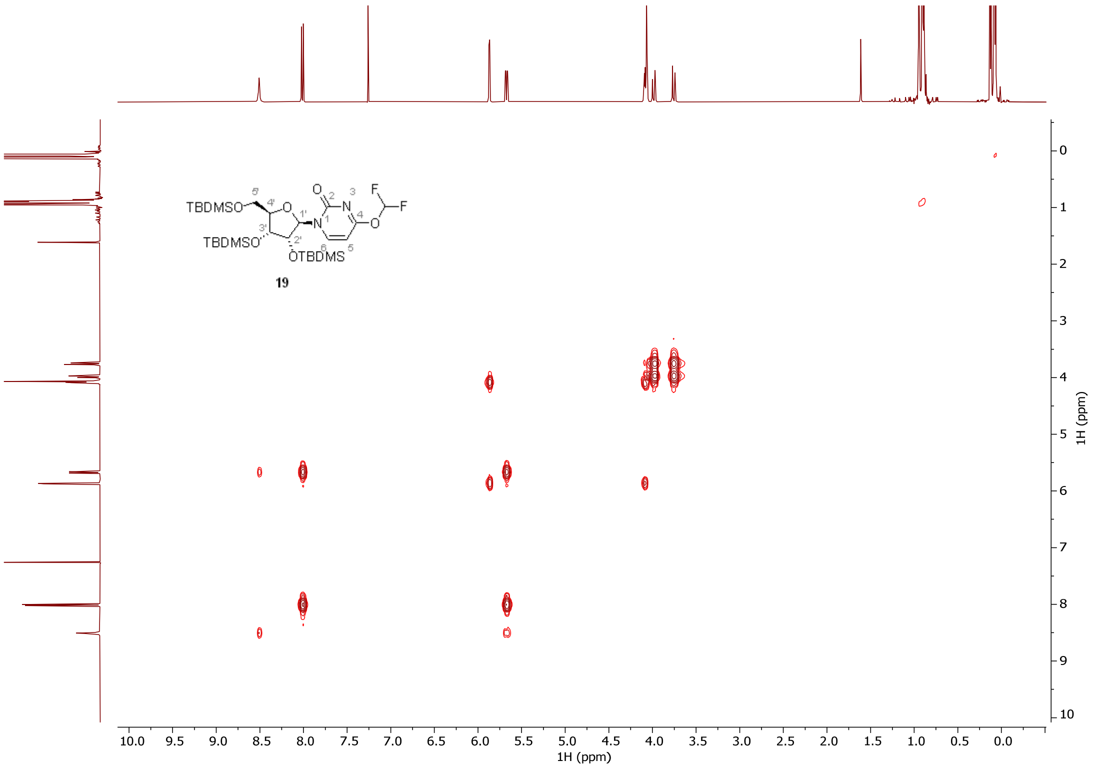

Figure S57 - ^{1}H-^{1}H COSY NMR spectrum of **19** in $CDCl_3$

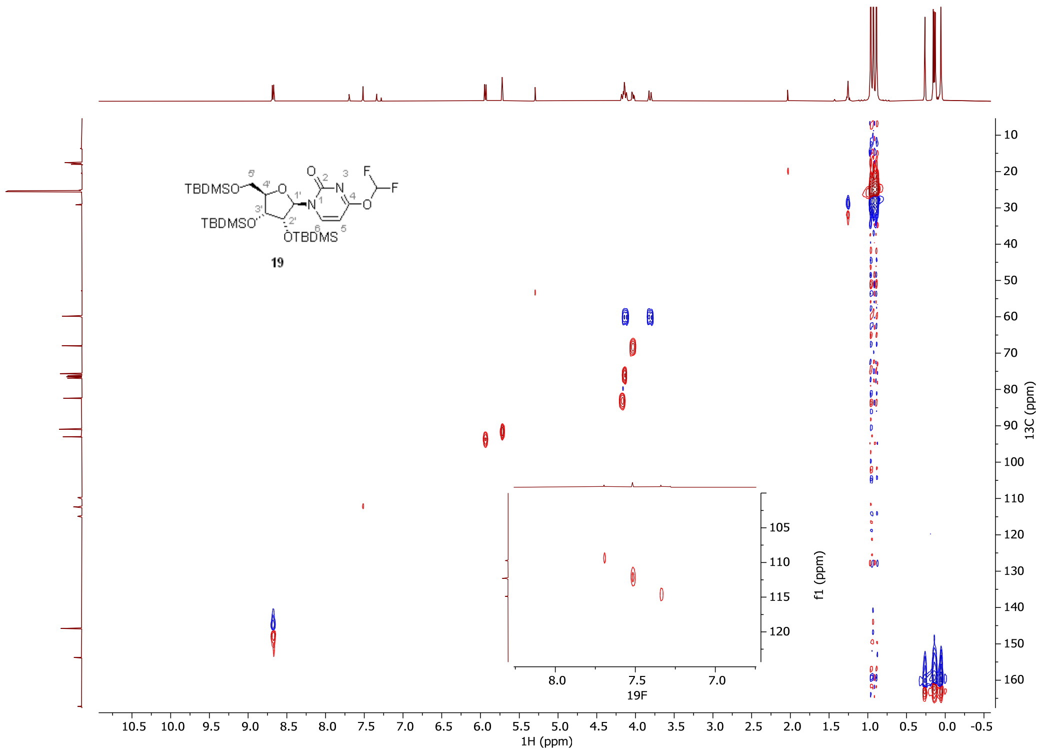

Figure S58 - ^{1}H-^{13}C HSQC NMR spectrum of **19** in $CDCl_3$

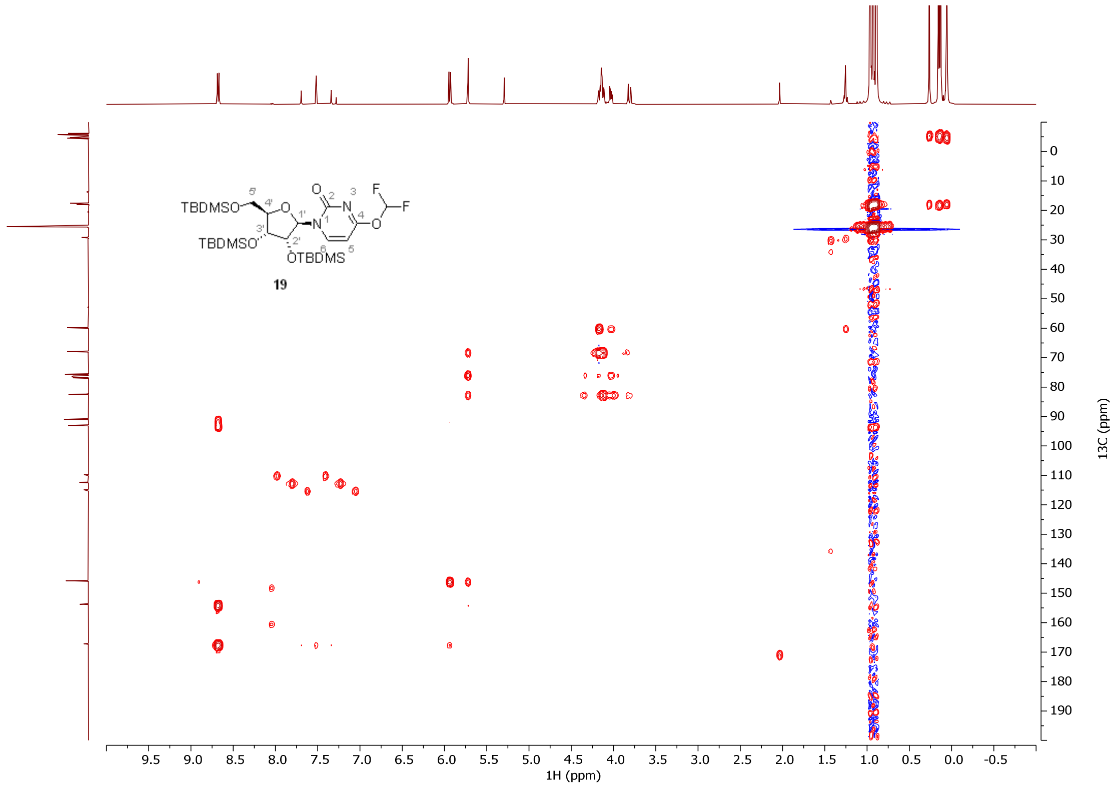

Figure S59 - ^{1}H-^{13}C HMBC NMR spectrum of **19** in $CDCl_3$

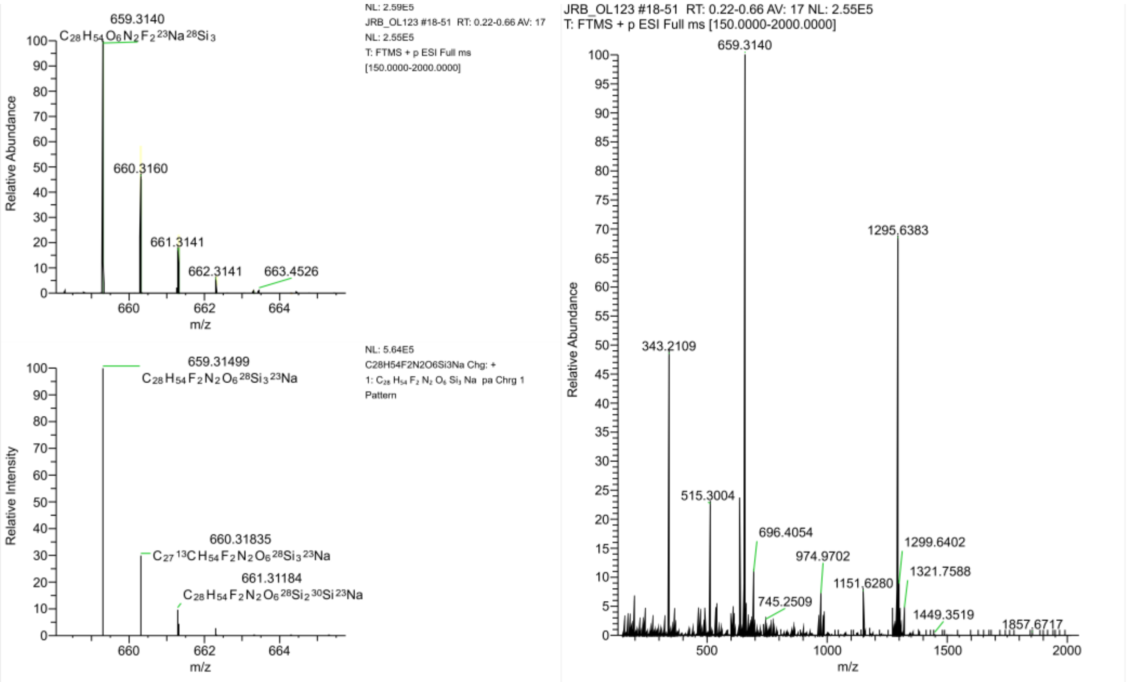

Peak Mass	Display For...	S Fit	RDB	Delta [ppm]	Theo. mass	Rank	Combined S...	# Matched I...	# Missed Iso.	MS Cov. [%]	Pattern Cov....	MSMS Matc...
659.3140	$C_{28}H_{54}O_6N_2F_2{}^{23}Na^{28}Si_3$	45.125693890522	4.50	-1.46	659.31499	1	96.43	6	0	99.28	99.88	(Collection)
659.3140	$C_{28}H_{44}O_4N_9F_2{}^{23}Na^{28}Si$	18.7190246210878	11.00	-0.83	659.31458	2	82.61	3	2	86.16	92.17	(Collection)

Figure S60 - HRMS spectrum of **19**

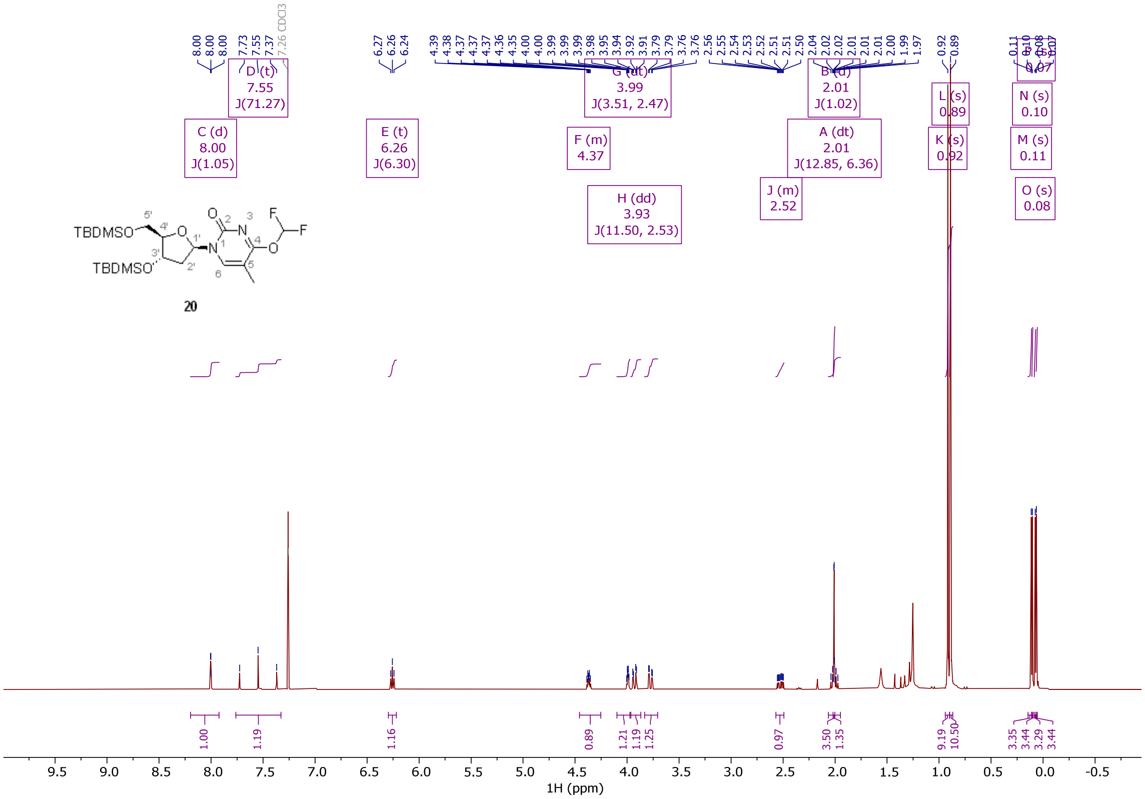

Figure S61 - ^1H NMR (400 MHz) spectrum of **20** in $CDCl_3$

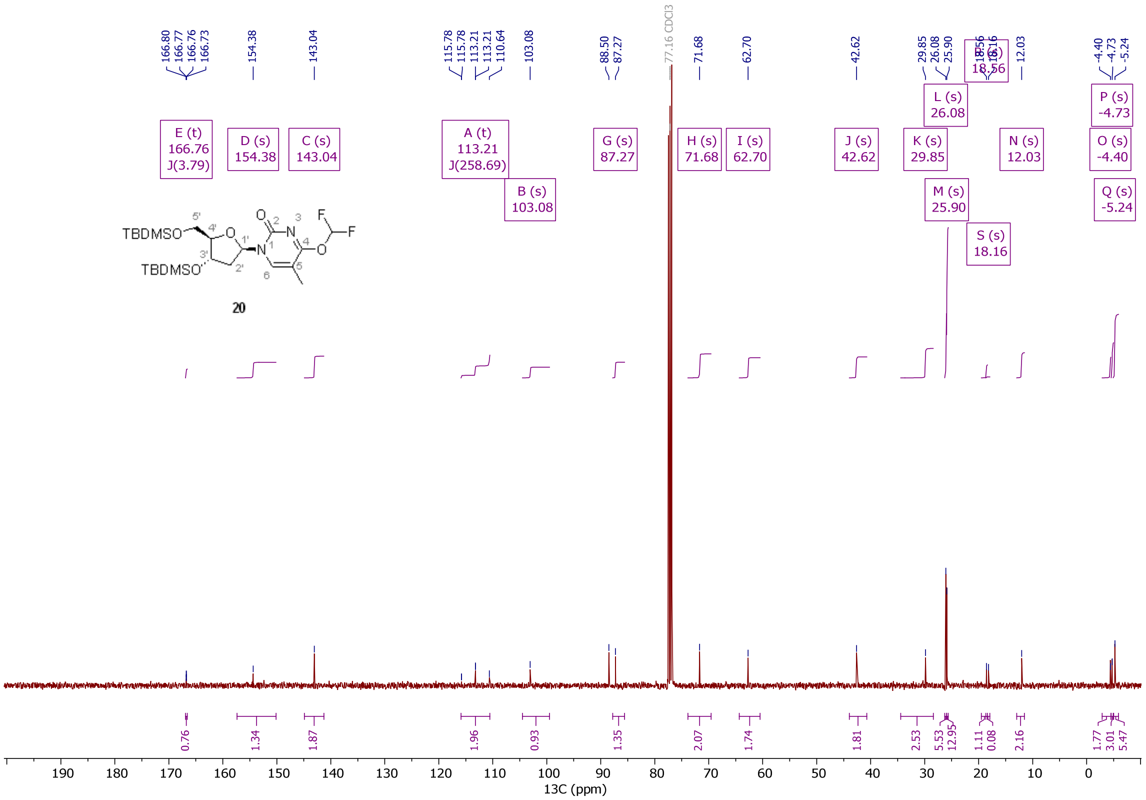

Figure S62 - ^{13}C NMR (101 MHz) spectrum of **20** in $CDCl_3$

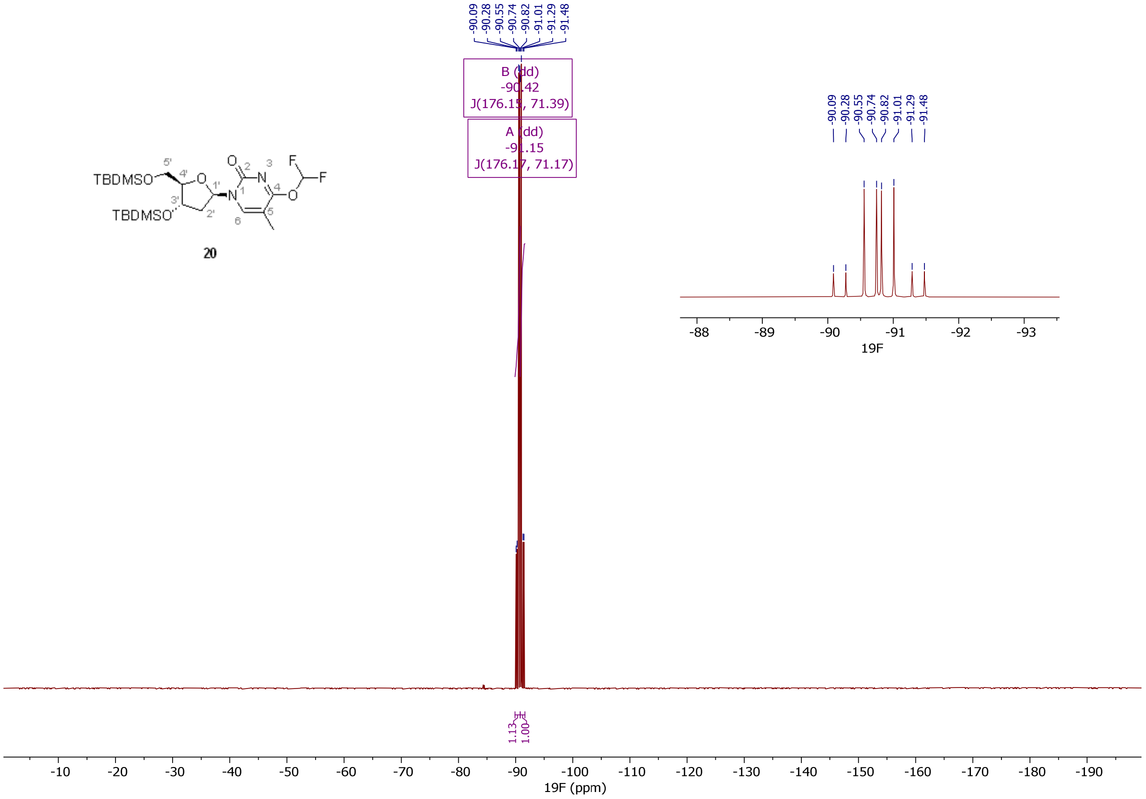

Figure S63 - ^{19}F NMR (376 MHz) spectrum of **20** in $CDCl_3$

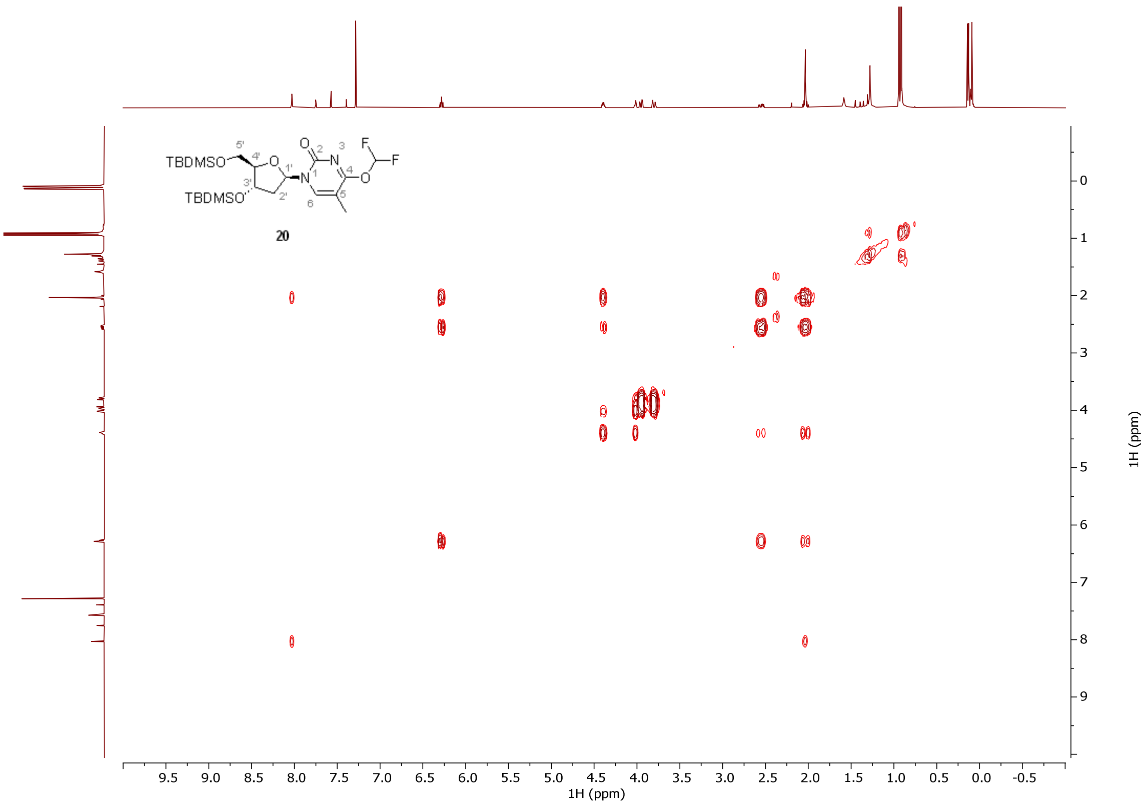

Figure S64 - ^{1}H-^{1}H COSY NMR spectrum of **20** in $CDCl_3$

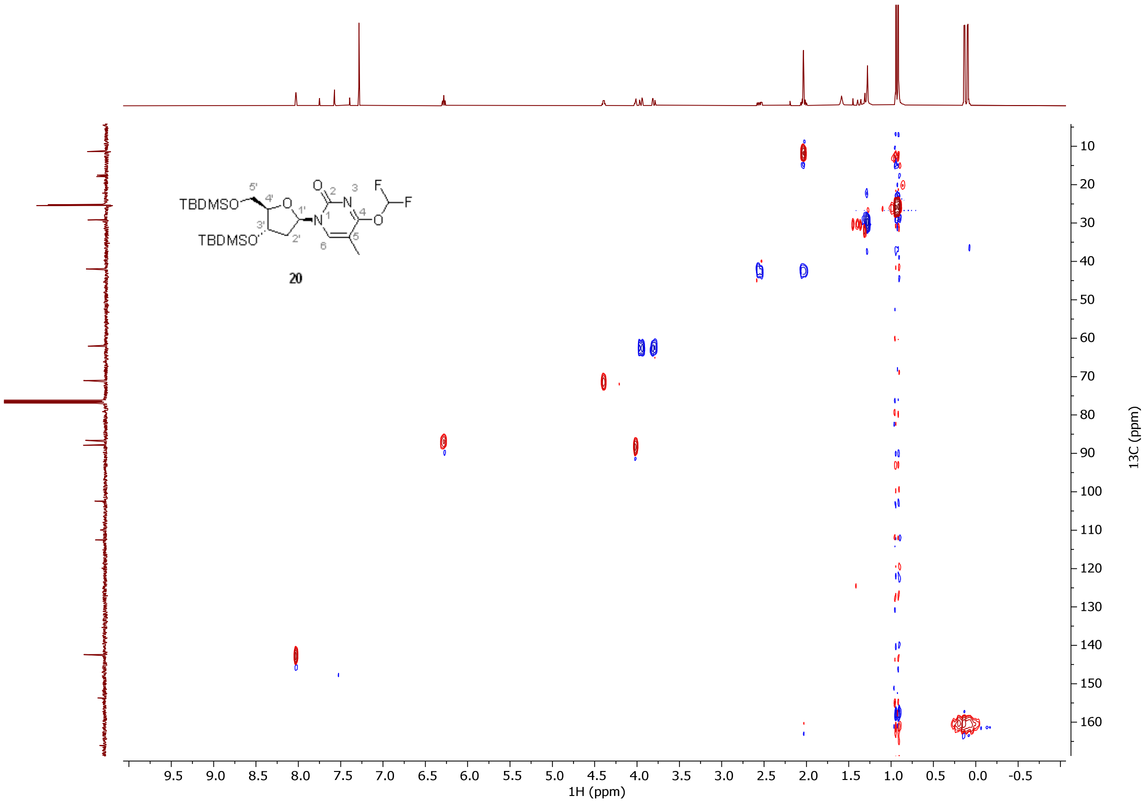

Figure S65 - ^{1}H-^{13}C HSQC NMR spectrum of **20** in $CDCl_3$

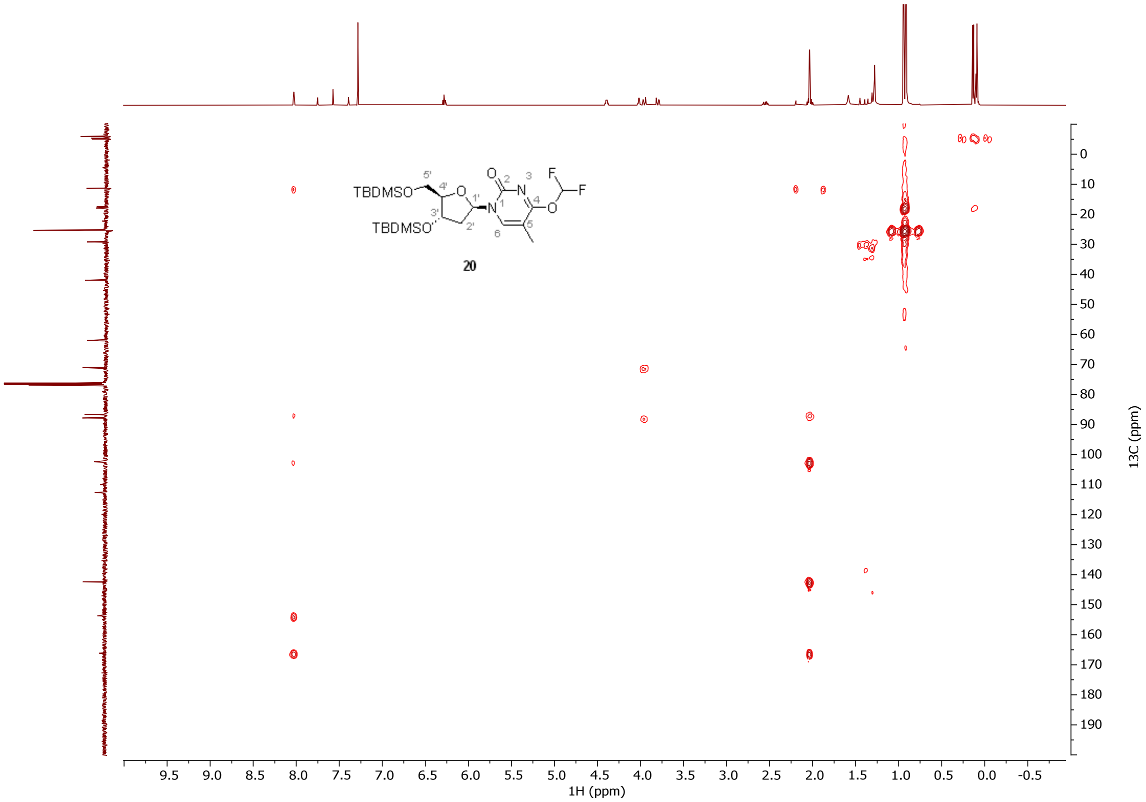

Figure S66 - ^{1}H-^{13}C HMBC NMR spectrum of **20** in CDCl$_3$

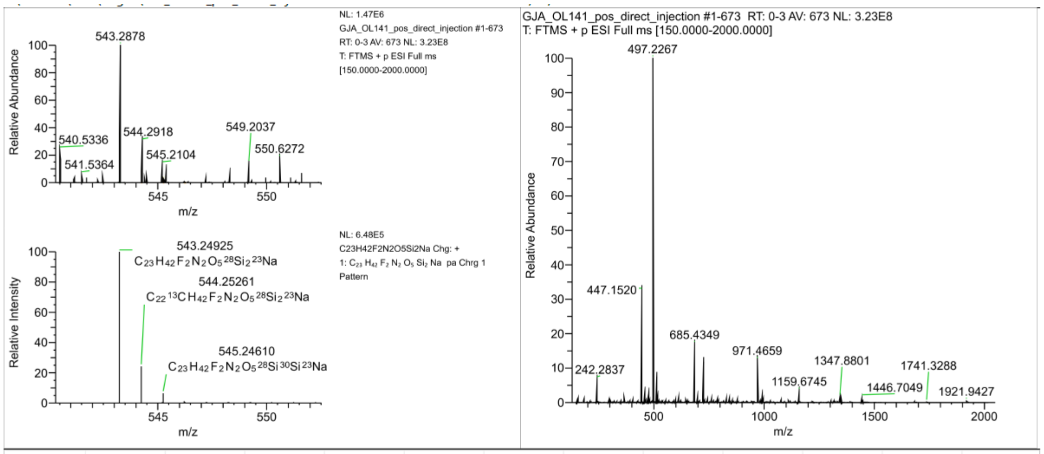

Peak Mass	Display For...	S Fit	RDB	Delta [ppm]	Theo. mass	Rank	Combined S...	# Matched I...	# Missed Iso.	MS Cov. [%]	Pattern Cov....	MSMS Matc...
543.2512	$C_{23}H_{40}O_{11}N_2{}^{23}Na$	37.6038079782069	4.50	-2.18	543.25243	1	21.73	2	1	20.85	95.93	(Collection)
543.2512	$C_{23}H_{37}O_{10}N_5$	28.0353840294775	8.00	-4.14	543.25349	2	21.23	2	1	20.85	95.98	(Collection)
543.2512	$C_{23}H_{38}O_7N_5F{}^{28}Si$	23.268472752629	8.00	-1.21	543.25190	3	20.98	3	1	20.85	94.17	(Collection)
543.2512	$C_{23}H_{41}O_8N_2F{}^{23}Na{}^{28}Si$	21.719135915522	4.50	0.74	543.25084	4	20.89	3	1	20.85	94.18	(Collection)
543.2512	$C_{23}H_{39}O_4N_5F_2{}^{28}Si_2$	12.5541279053664	8.00	1.71	543.25032	5	16.67	3	1	16.9	68.04	(Collection)
543.2512	$C_{23}H_{32}O_3N_9F_2{}^{23}Na$	10.689251842408	11.00	4.43	543.24884	6	16.57	3	0	16.9	76.43	(Collection)
543.2512	$C_{23}H_{42}O_5N_2F_2{}^{23}Na{}^{28}Si_2$	9.59412606694054	4.50	3.67	543.24925	7	16.51	4	0	16.9	68.41	(Collection)
543.2512	$C_{23}H_{39}O_{11}NF_2$	8.97126707456043	4.00	4.93	543.24857	8	16.48	3	0	16.9	76.26	(Collection)

Figure S67 -HMRS spectrum of **20**

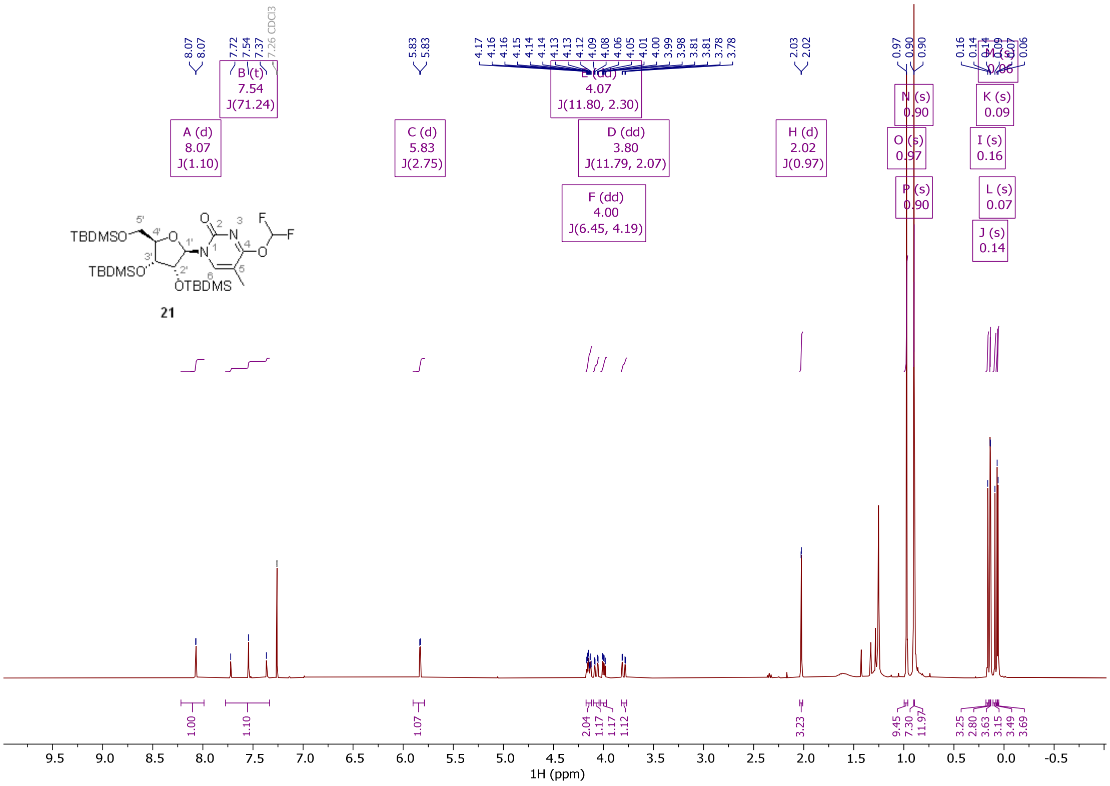

Figure S68 - ^{1}H NMR (400 MHz) spectrum of **21** in $CDCl_3$

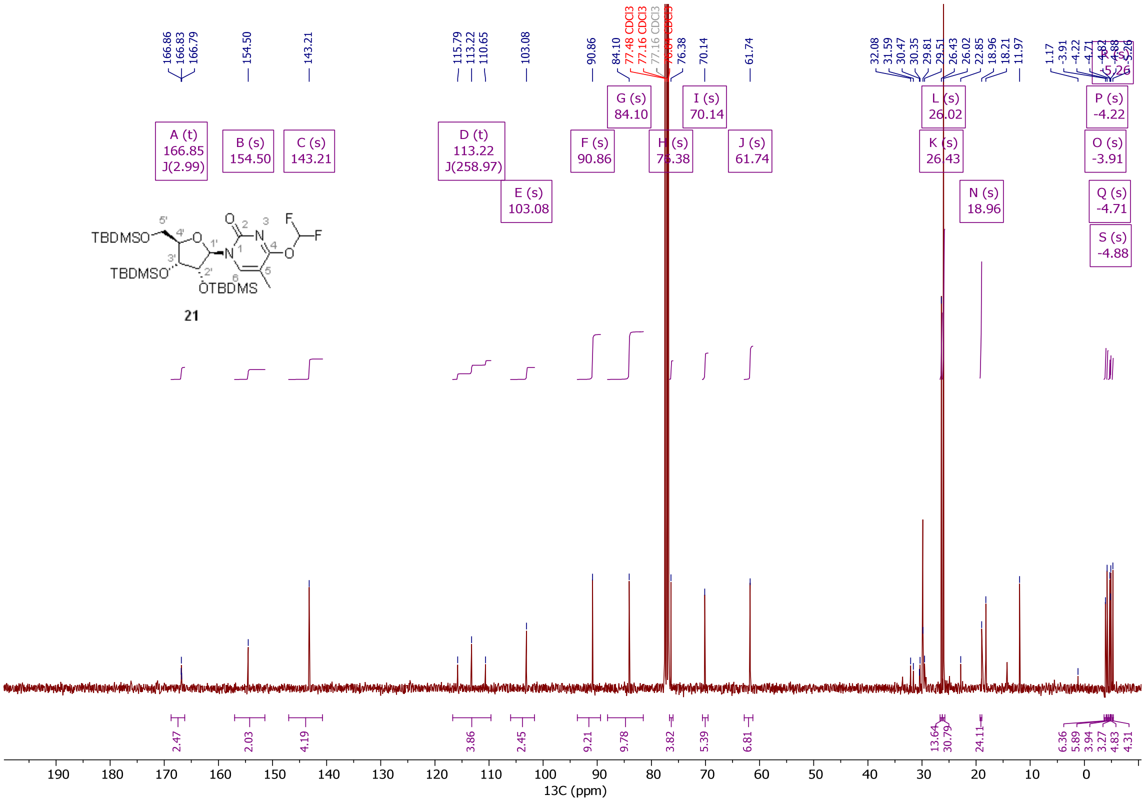

Figure S69 - ^{13}C NMR (101 MHz) spectrum of **21** in $CDCl_3$

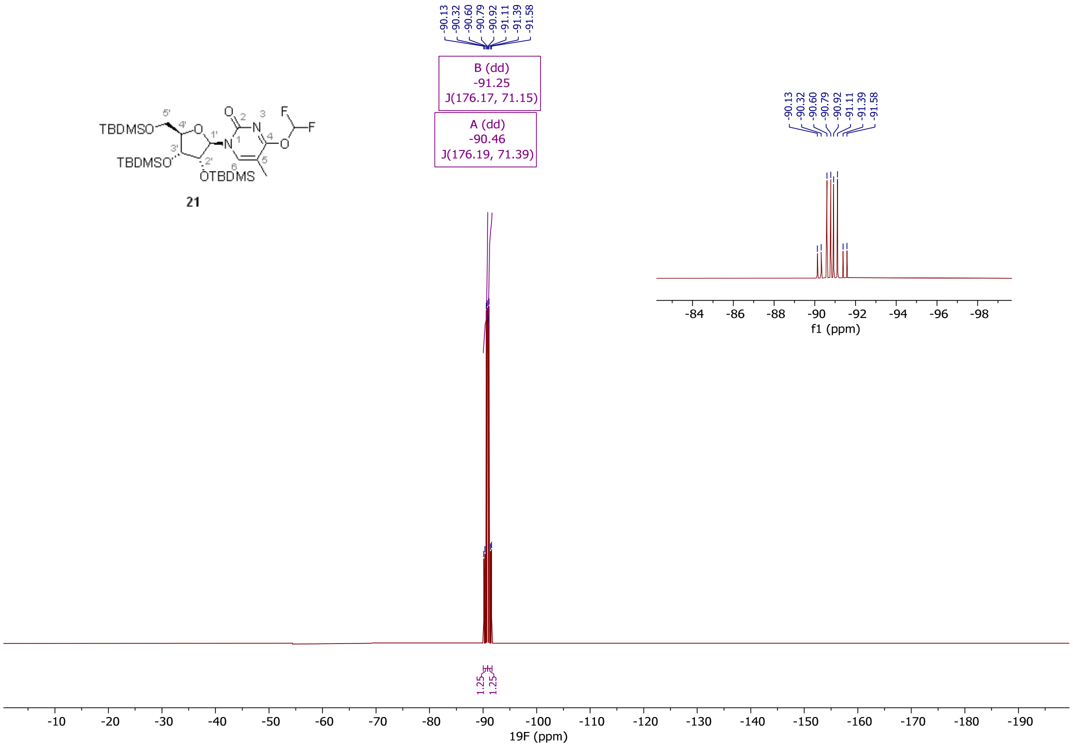

Figure S70 - ^{19}F NMR (376 MHz) spectrum of **21** in $CDCl_3$

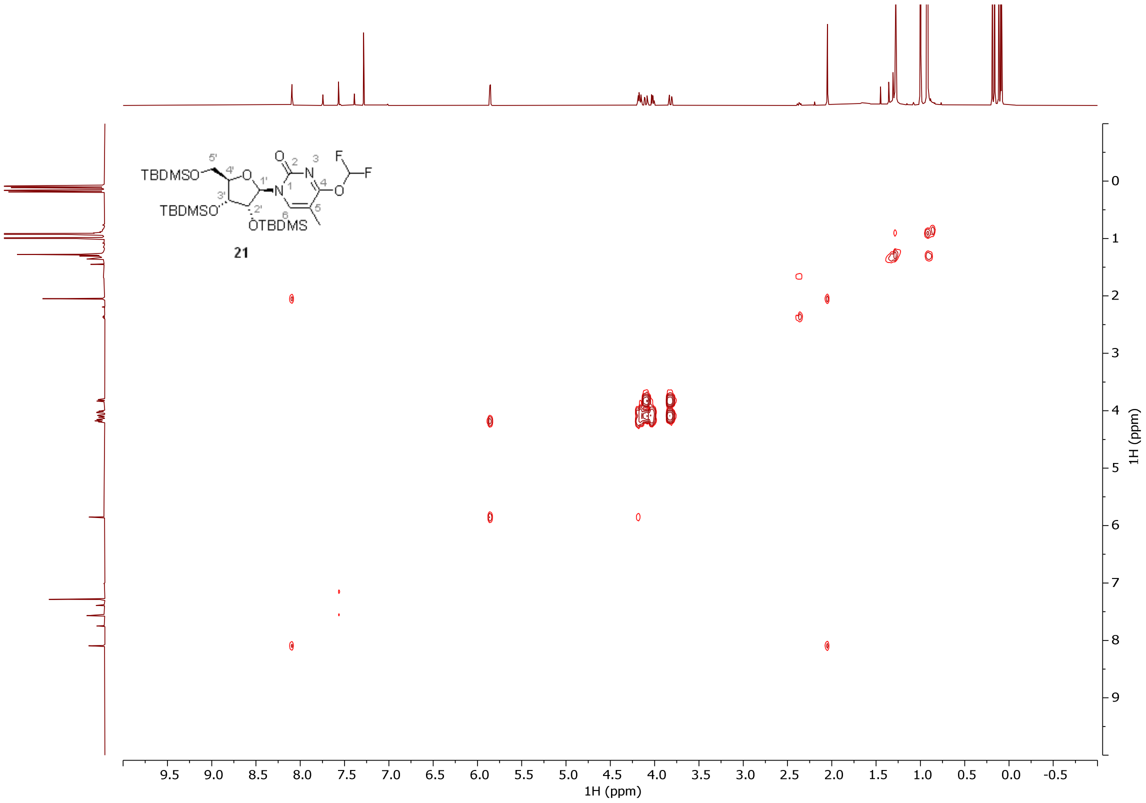

Figure S71 - ^{1}H-^{1}H COSY NMR spectrum of **21** in $CDCl_3$

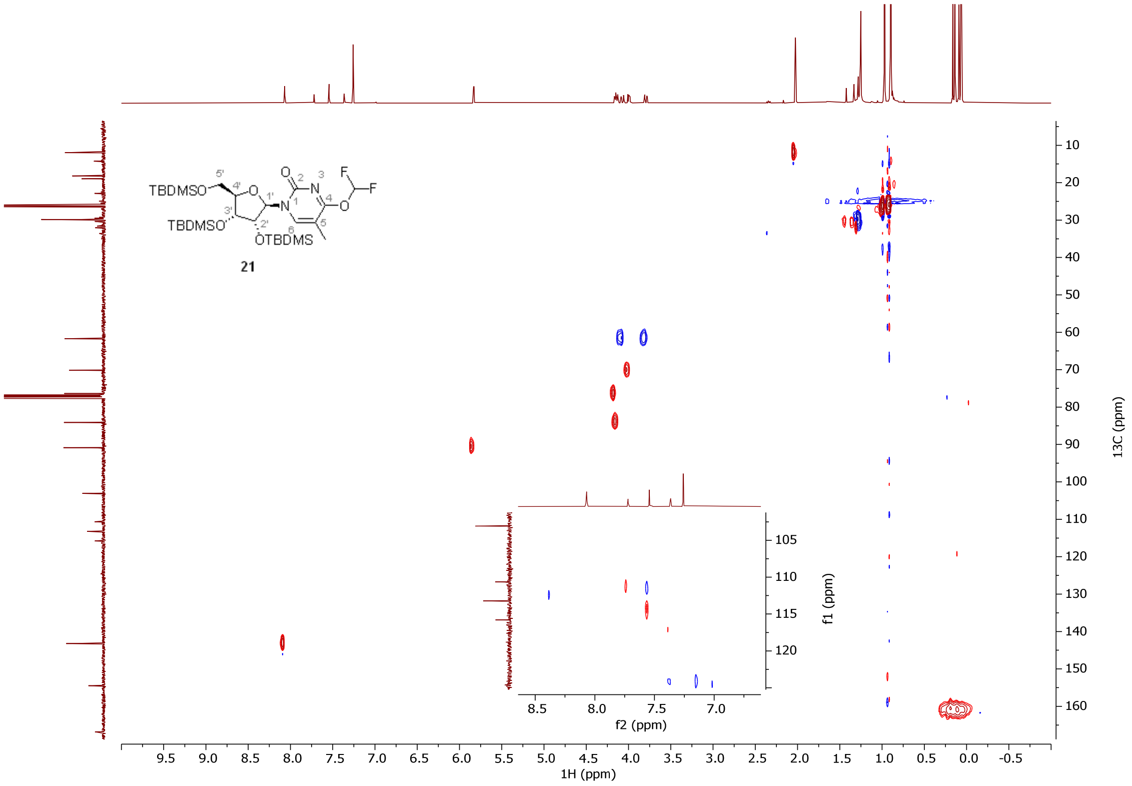

Figure S72 - ^{1}H-^{13}C HSQC NMR spectrum of **21** in $CDCl_3$

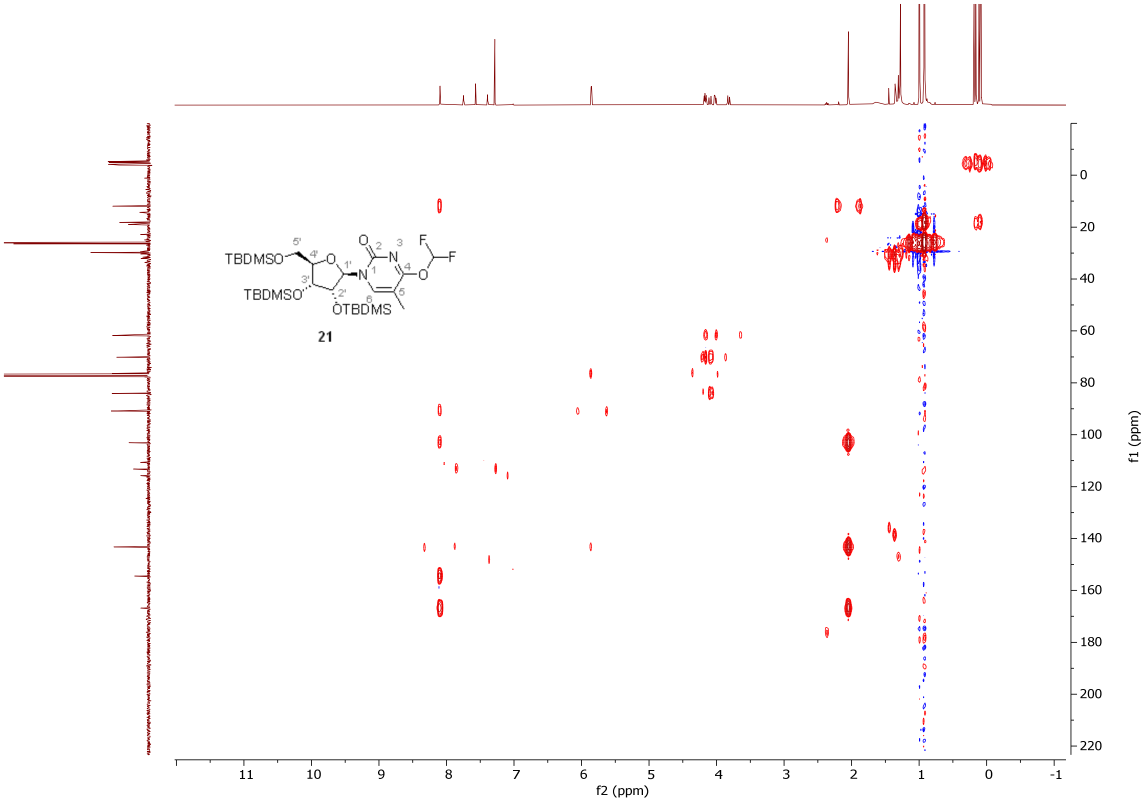

Figure S73 - ^{1}H-^{13}C HMBC NMR spectrum of **21** in $CDCl_3$

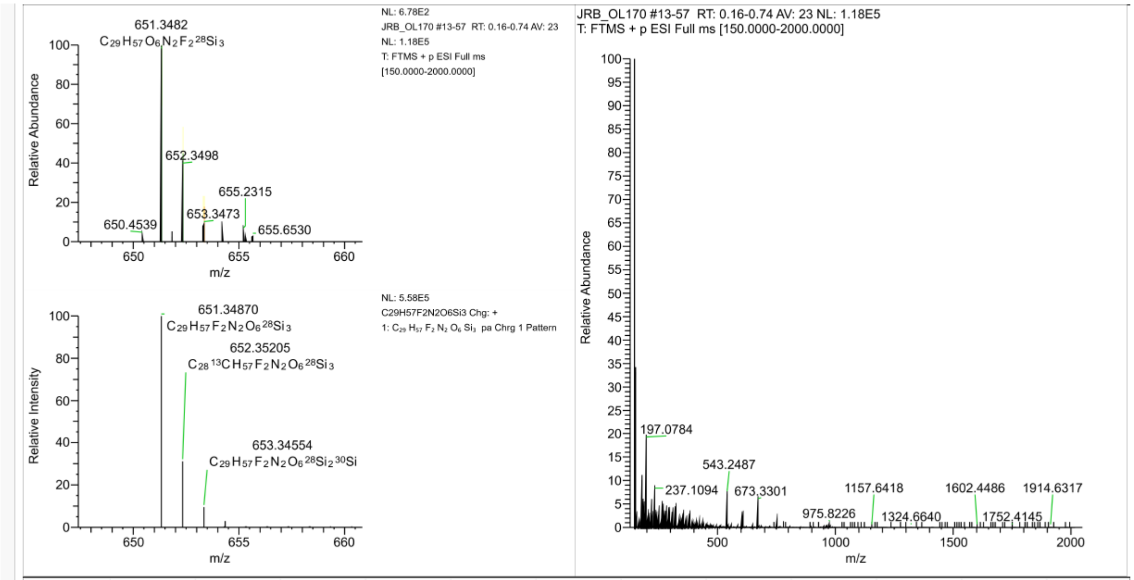

Peak Mass	Display For...	S Fit	RDB	Delta [ppm]	Theo. mass	Rank	Combined S...	# Matched I...	# Missed Iso.	MS Cov. [%]	Pattern Cov....	MSMS Matc...
651.3482	$C_{27}H_{55}O_5N_5F_2\,^{28}Si_3$	38.6232111485843	5.00	1.34	651.34736	1	85.16	3	0	87.74	89.38	(Collection)
651.3482	$C_{29}H_{57}O_6N_2F_2\,^{28}Si_3$	37.5100447325234	4.50	-0.72	651.34870	2	85.1	3	0	87.74	88.93	(Collection)
651.3482	$C_{28}H_{51}ON_9F_2\,^{28}Si_3$	37.0997985125136	10.00	-0.71	651.34869	3	85.08	3	0	87.74	89.25	(Collection)
651.3482	$C_{26}H_{59}O_9NF_2\,^{28}Si_3$	31.9244277300208	0.00	3.40	651.34602	4	84.81	3	0	87.74	89.02	(Collection)
651.3482	$C_{25}H_{53}O_4N_8F_2\,^{28}Si_3$	29.9373031439935	5.50	3.41	651.34601	5	84.7	3	0	87.74	89.32	(Collection)
651.3482	$C_{30}H_{53}O_2N_6F_2\,^{28}Si_3$	27.20749494137	9.50	-2.77	651.35004	6	84.56	3	0	87.74	89.48	(Collection)

Figure S74 – HRMS spectrum of **21**

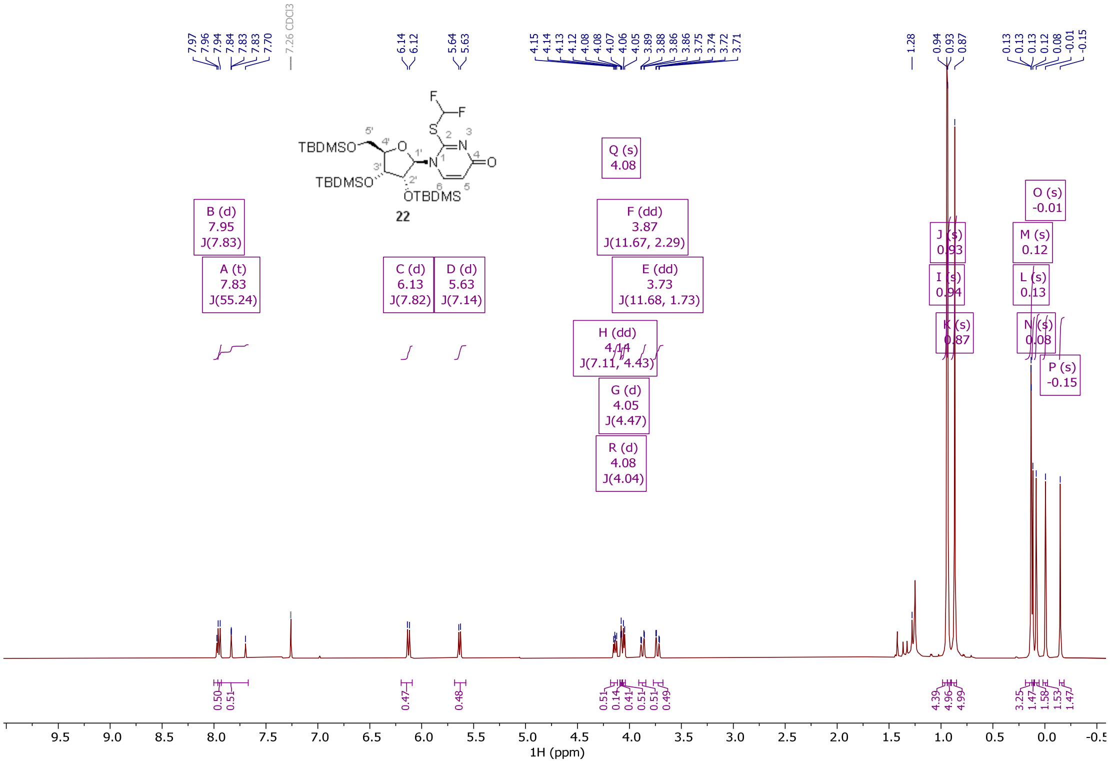

Figure S75 - ^{1}H NMR (400 MHz) spectrum of **22** in $CDCl_3$

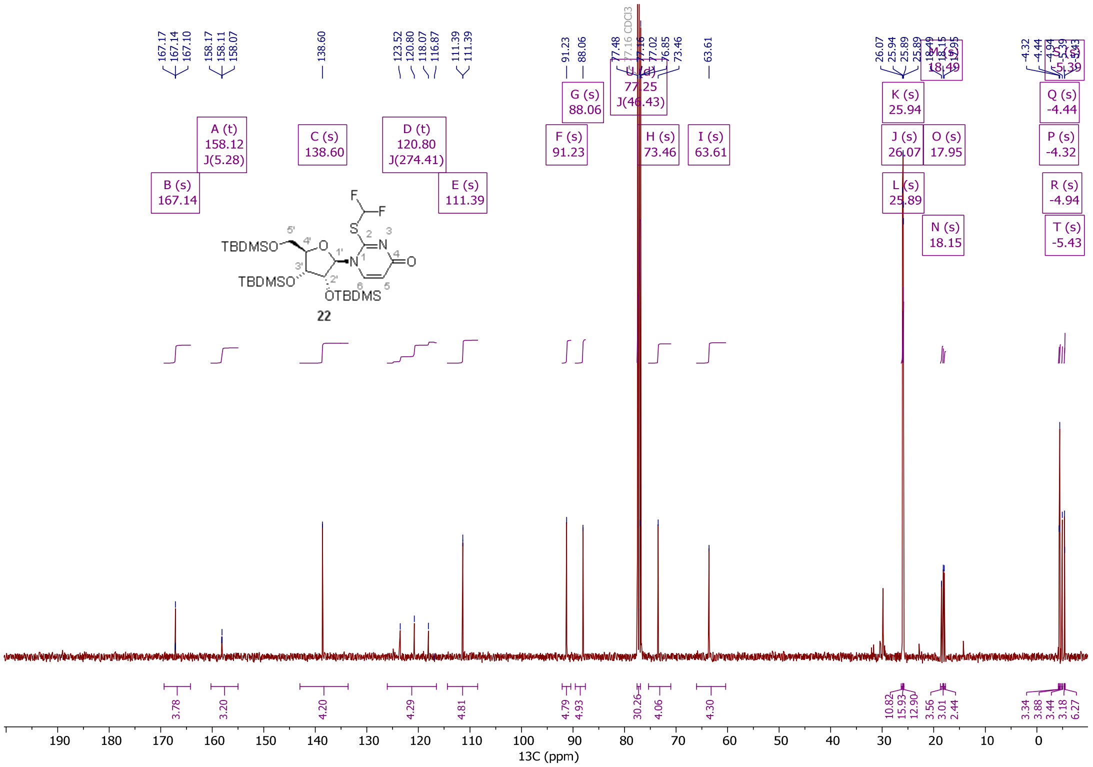

Figure S76 - ^{13}C NMR (101 MHz) spectrum of **22** in $CDCl_3$

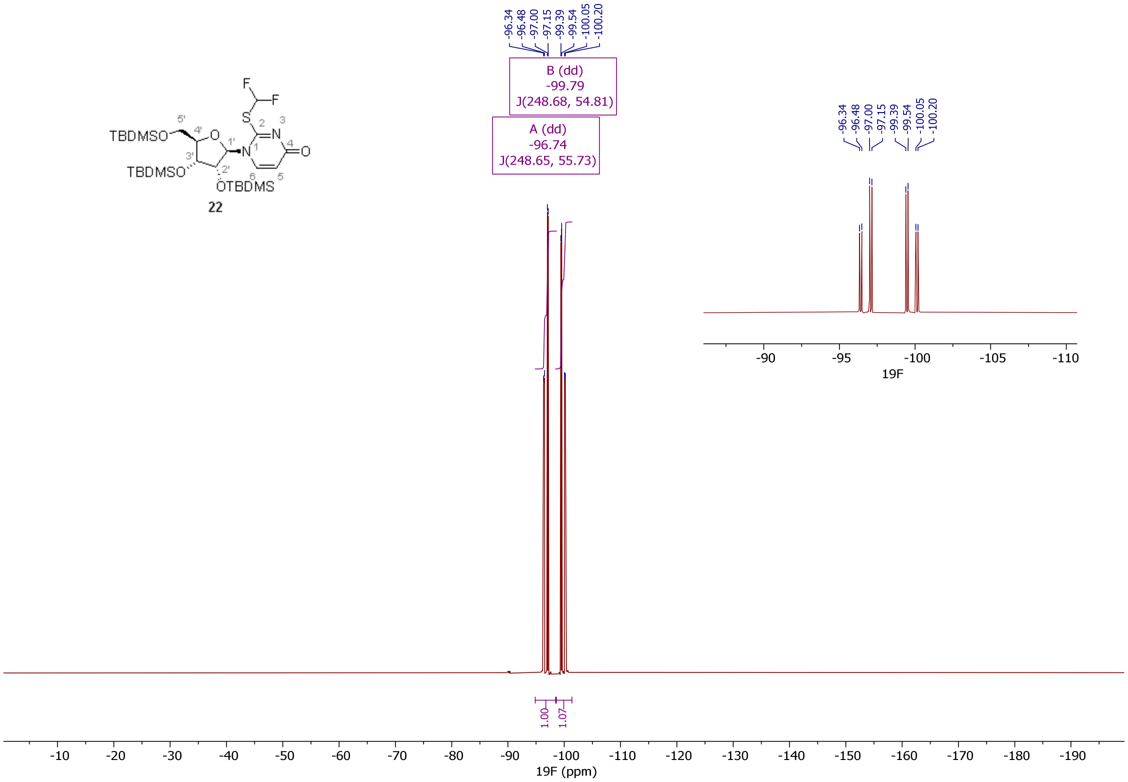

Figure S77 - ^{19}F NMR (376 MHz) spectrum of **22** in $CDCl_3$

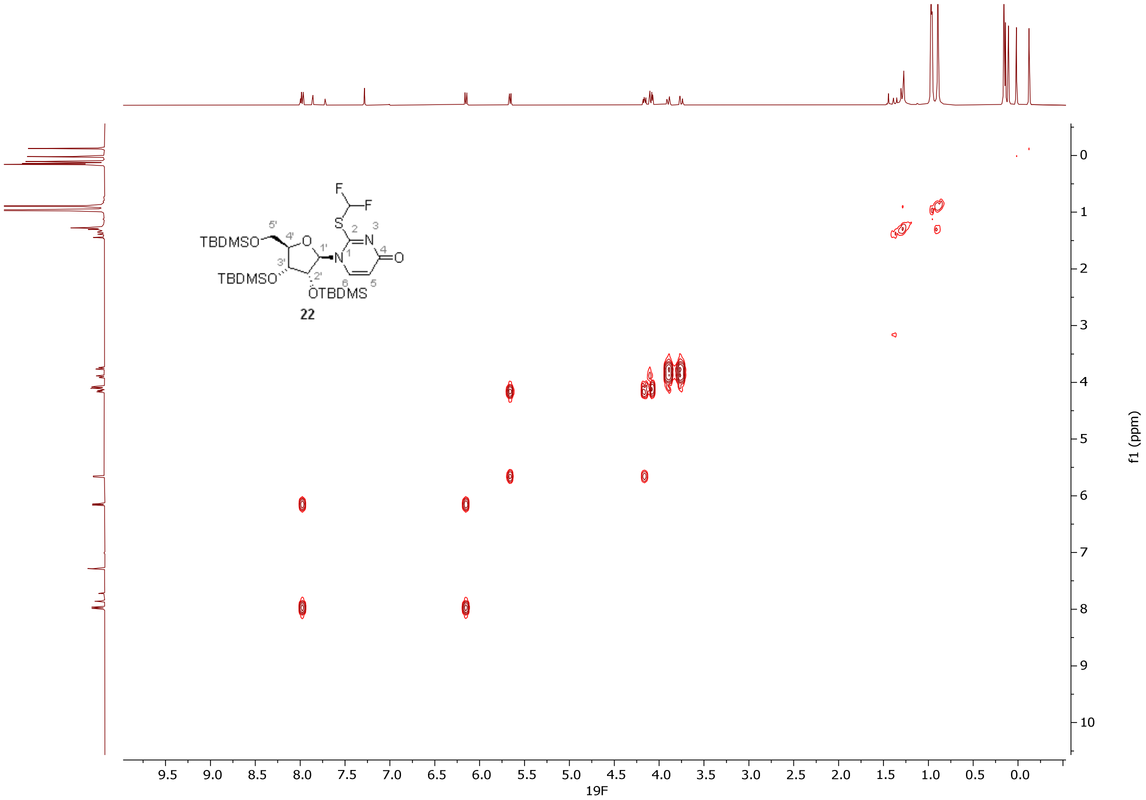

Figure S78 - ^{1}H-^{1}H COSY NMR spectrum of **22** in $CDCl_3$

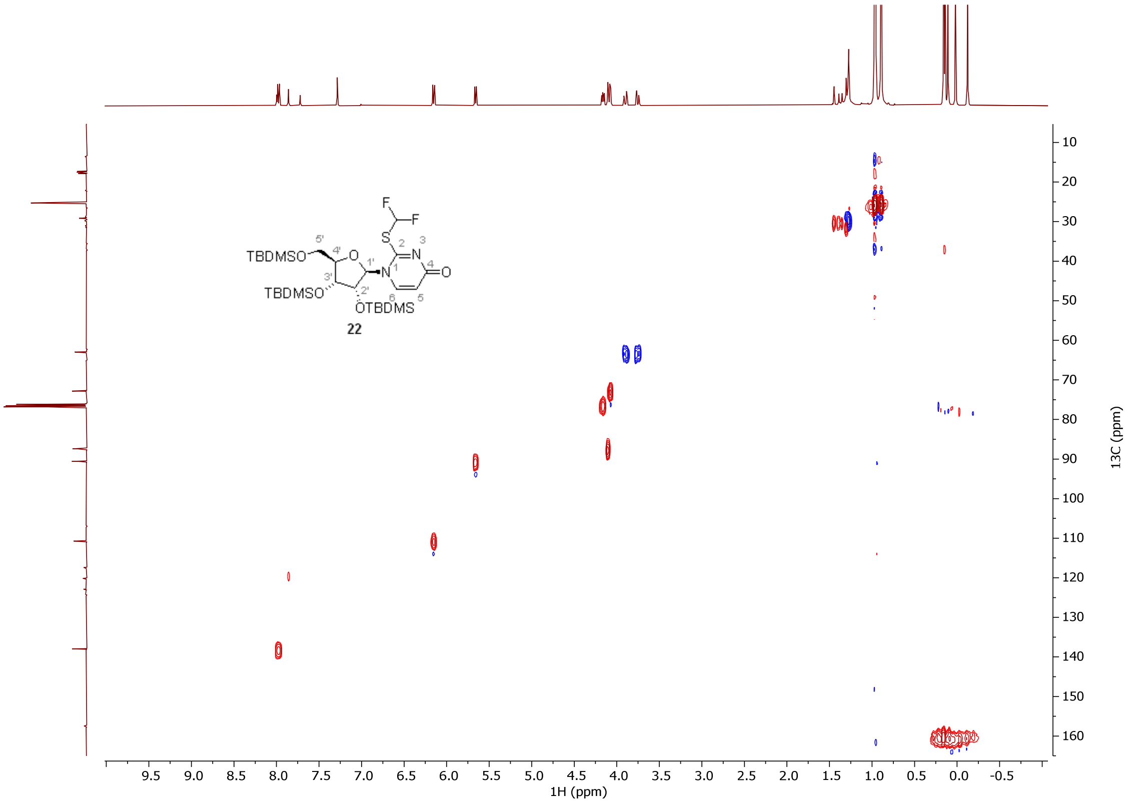

Figure S79 - ^{1}H-^{13}C HSQC NMR spectrum of **22** in $CDCl_3$

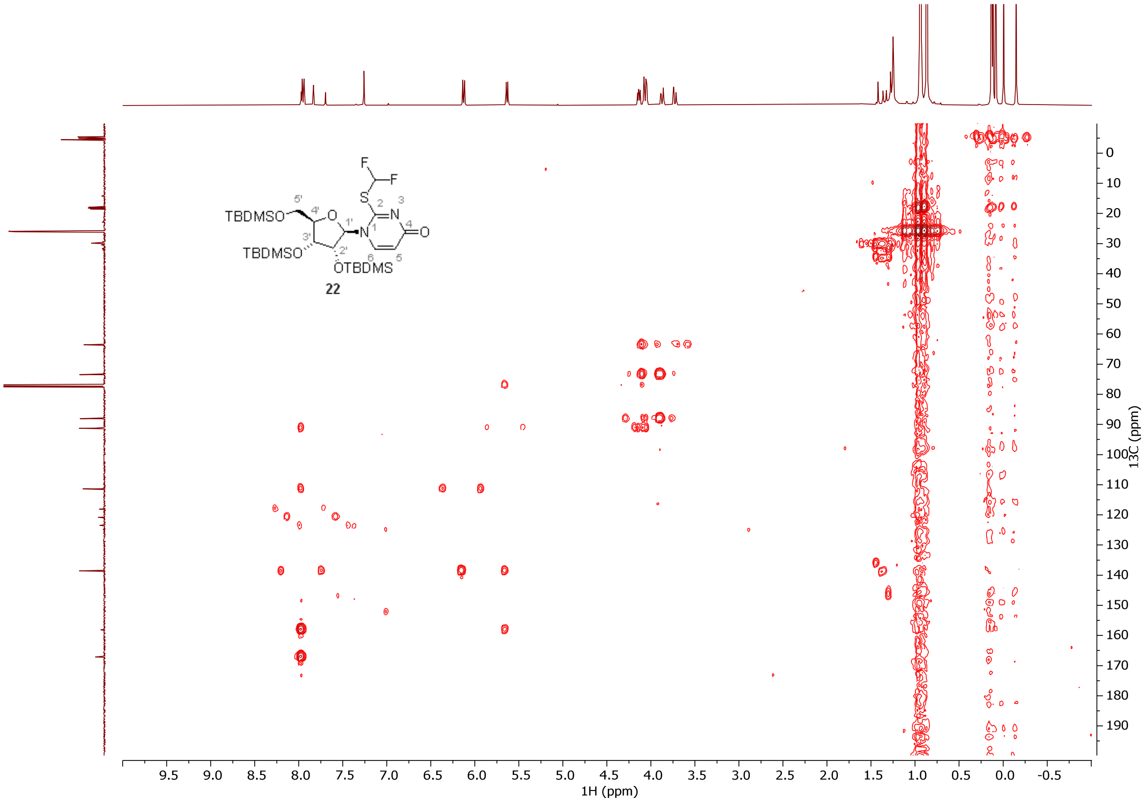

Figure S80 - ^{1}H-^{13}C HMBC NMR spectrum of **22** in $CDCl_3$

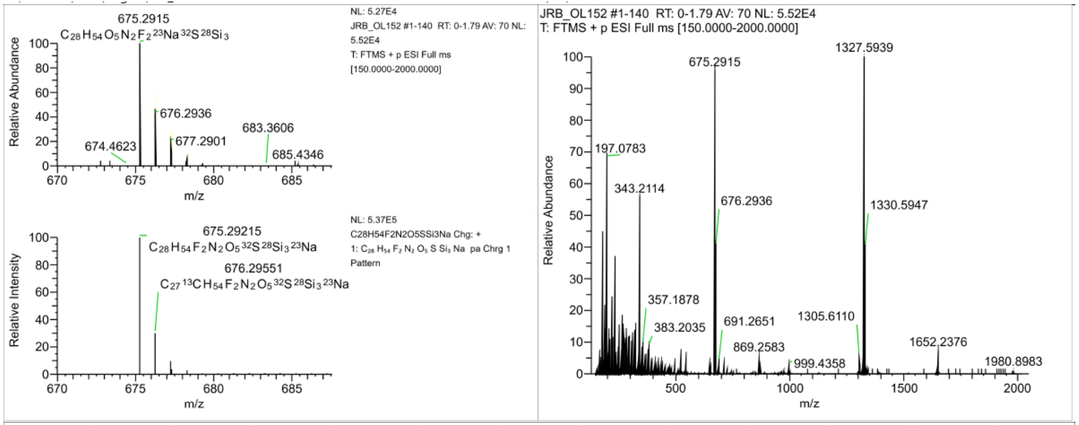

Peak Mass	Display For...	S Fit	RDB	Delta [ppm]	Theo. mass	Rank	Combined S...	# Matched I...	# Missed Iso.	MS Cov. [%]	Pattern Cov....	MSMS Matc...
675.2915	$C_{28}H_{51}O_9N_4{}^{32}S^{28}Si_2$	64.1475627390725	7.50	0.75	675.29098	1	98.07	6	0	99.95	100	(Collection)
675.2915	$C_{28}H_{50}O_7N_5{}^{23}Na^{28}Si_3$	63.813454682856	9.00	0.69	675.29102	2	98.05	6	0	99.95	100	(Collection)
675.2915	$C_{28}H_{54}O_{10}N^{23}Na^{32}S^{28}Si_2$	57.8720857102009	4.00	2.33	675.28992	3	97.73	6	0	99.95	100	(Collection)
675.2915	$C_{28}H_{47}O_6N_8{}^{28}Si_3$	53.8675731743695	12.50	-0.89	675.29209	4	97.52	6	0	99.95	100	(Collection)
675.2915	$C_{28}H_{54}O_5N_2F_2{}^{23}Na^{32}S^{28}Si_3$	46.9966940397905	4.50	-0.98	675.29215	5	97.07	6	0	99.85	99.77	(Collection)
675.2915	$C_{28}H_{51}O_4N_5F_2{}^{32}S^{28}Si_3$	37.3102114129809	8.00	-2.56	675.29321	6	96.56	6	0	99.85	99.78	(Collection)
675.2915	$C_{28}H_{52}O_6N_4F^{32}S^{28}Si_3$	31.9159587737755	7.50	3.11	675.28939	7	95.46	6	0	98.99	98.63	(Collection)
675.2915	$C_{28}H_{55}O_7NF^{23}Na^{32}S^{28}Si_3$	23.6356905024469	4.00	4.68	675.28833	8	95.03	6	0	98.99	98.63	(Collection)
675.2915	$C_{28}H_{50}O_9N_2F_2{}^{23}Na^{28}Si_2$	21.9337635655357	5.50	-0.04	675.29151	9	80.43	5	0	83.67	88.43	(Collection)
675.2915	$C_{28}H_{44}O_3N_9F_2{}^{23}Na^{32}S^{28}Si$	20.6781902497205	11.00	-0.37	675.29174	10	80.36	4	1	83.67	89.05	(Collection)

Figure S81 - HMRS spectrum of **22**

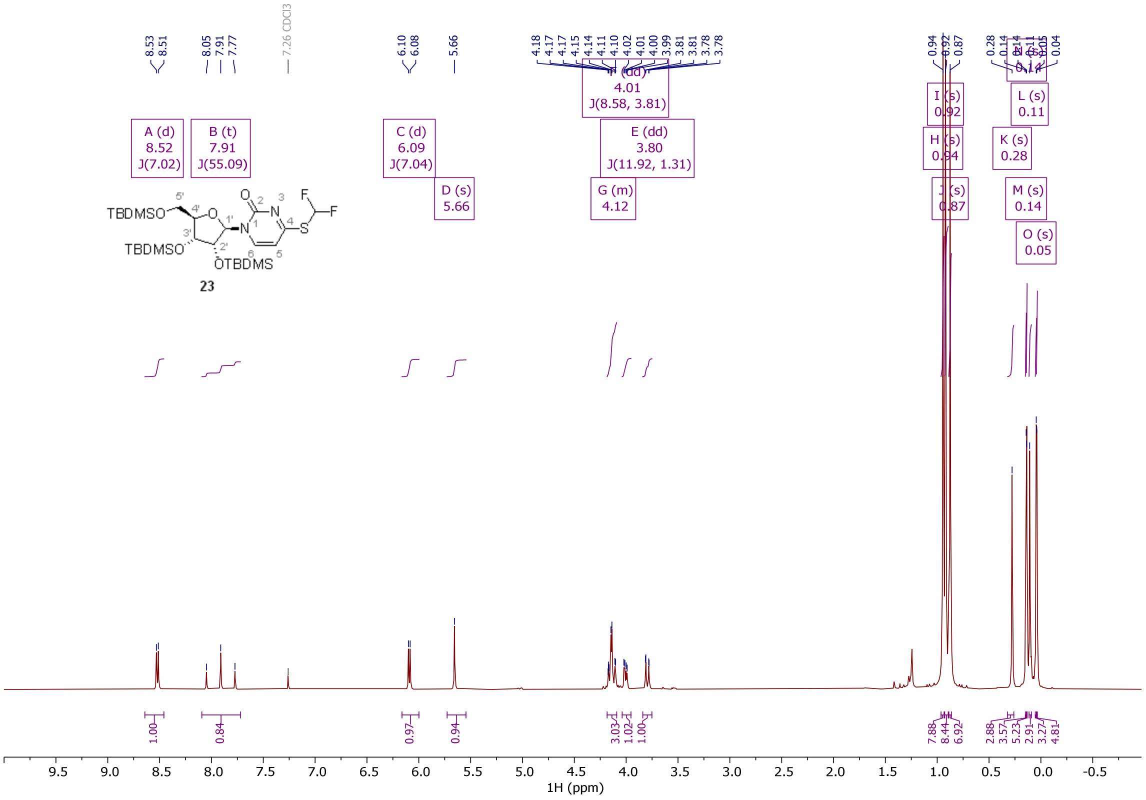

Figure S82 - ^{1}H NMR (400 MHz) spectrum of **23** in $CDCl_3$

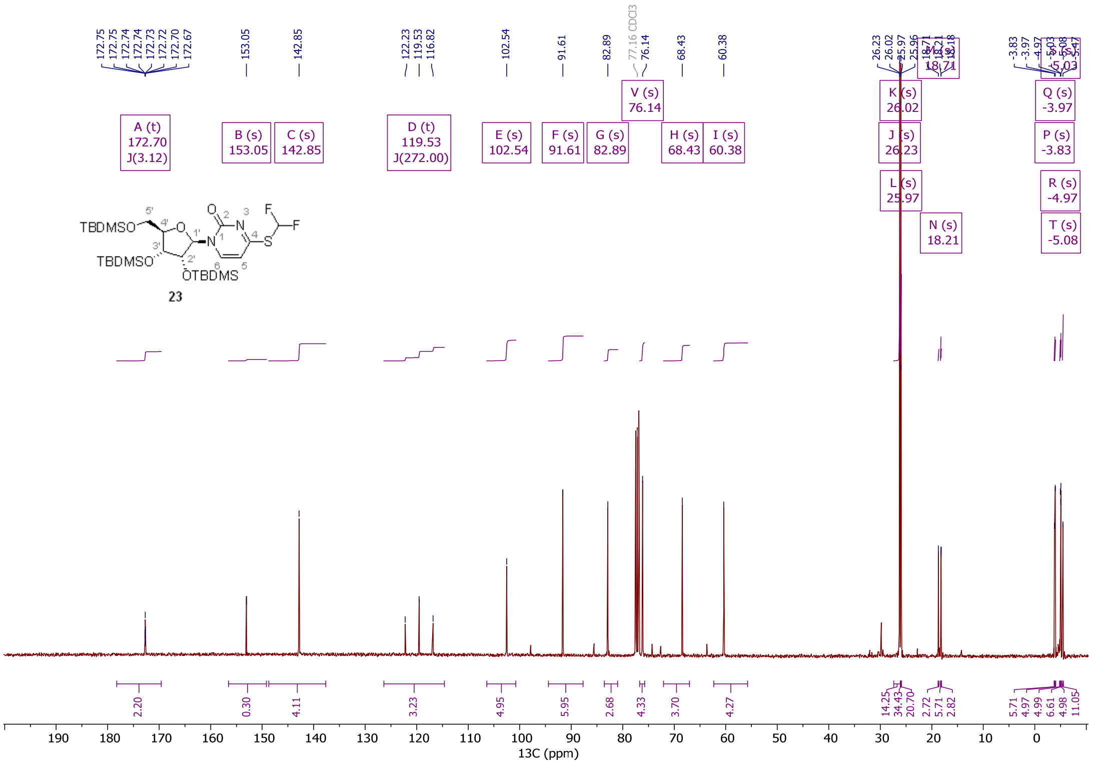

Figure S83 - ^{13}C NMR (101 MHz) spectrum of **23** in $CDCl_3$

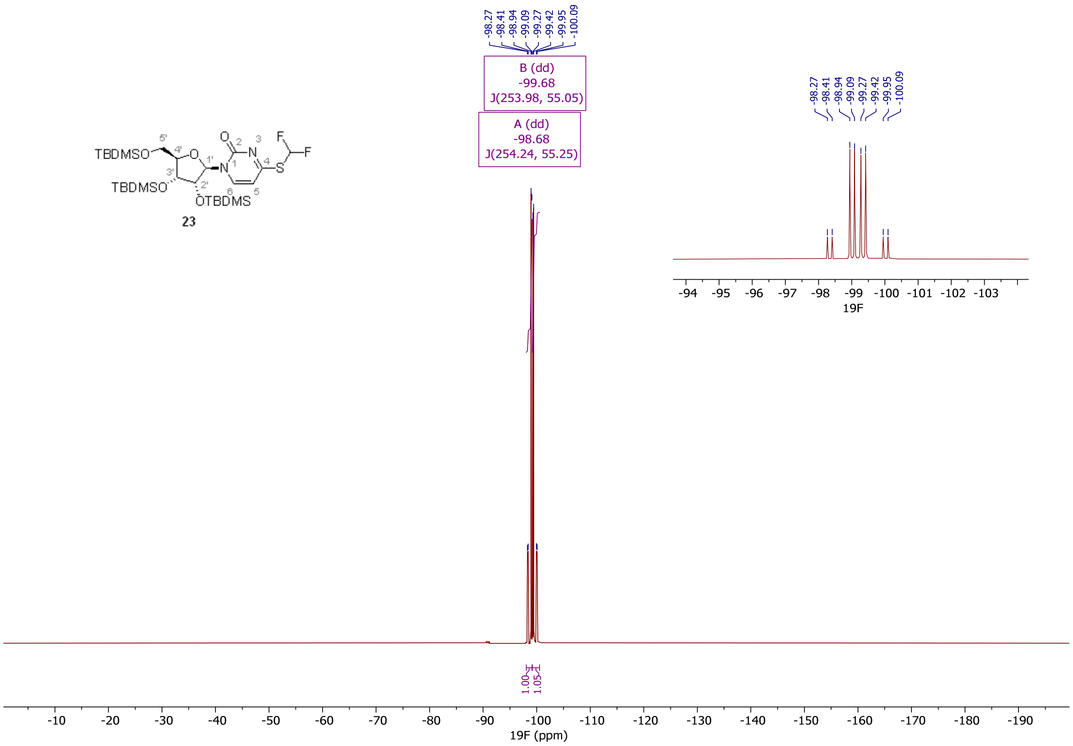

Figure S84 - ^{19}F NMR (376 MHz) spectrum of **23** in $CDCl_3$

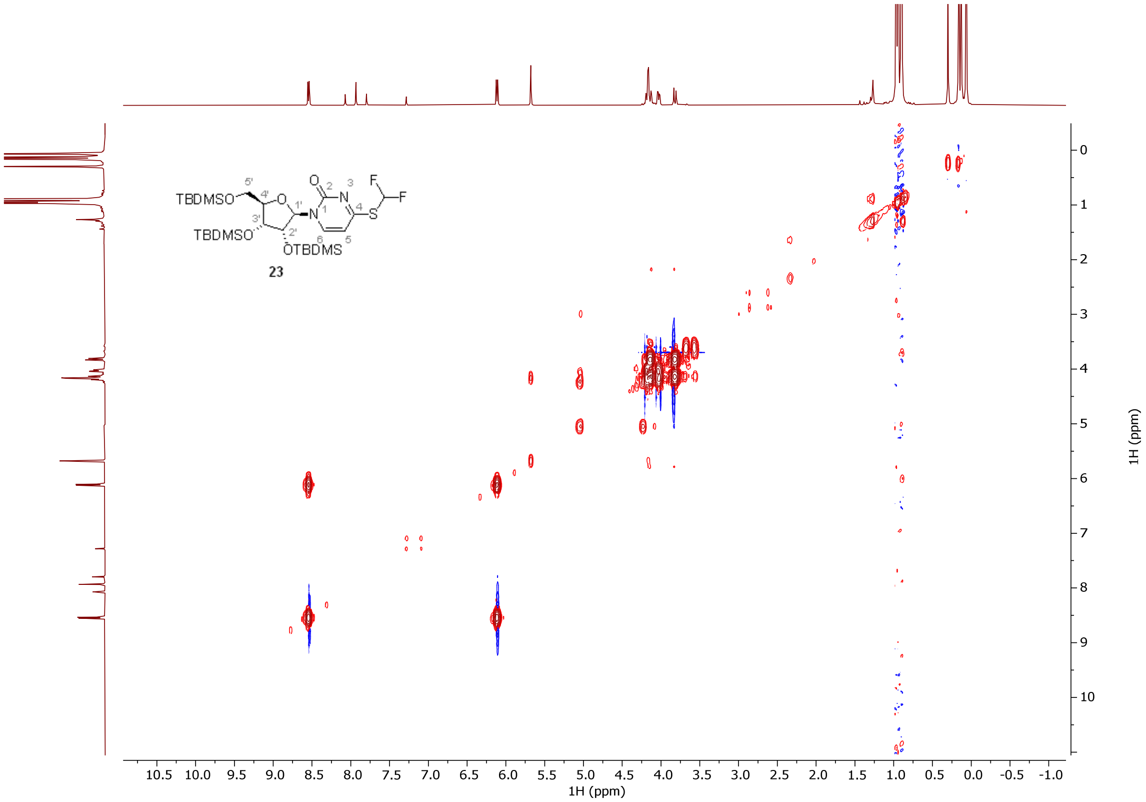

Figure S85 - ^{1}H-^{1}H COSY NMR spectrum of **23** in $CDCl_3$

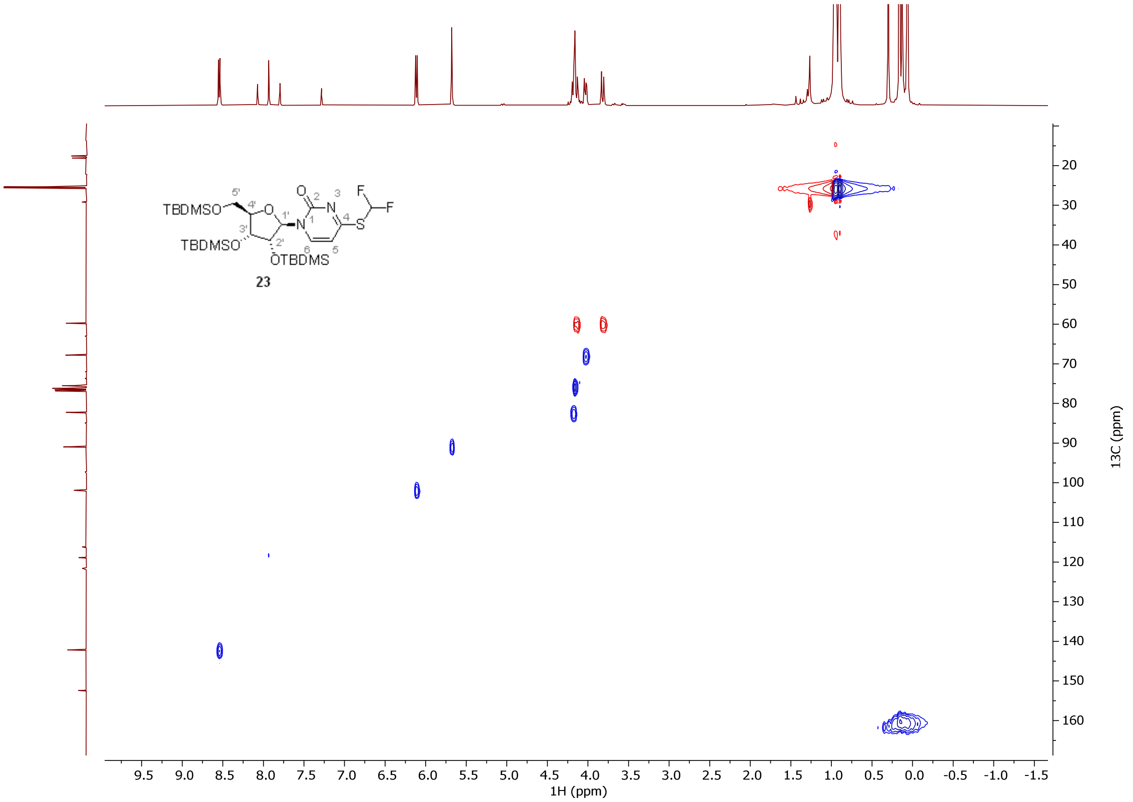

Figure S86 - ^{1}H-^{13}C HSQC NMR spectrum of **23** in $CDCl_3$

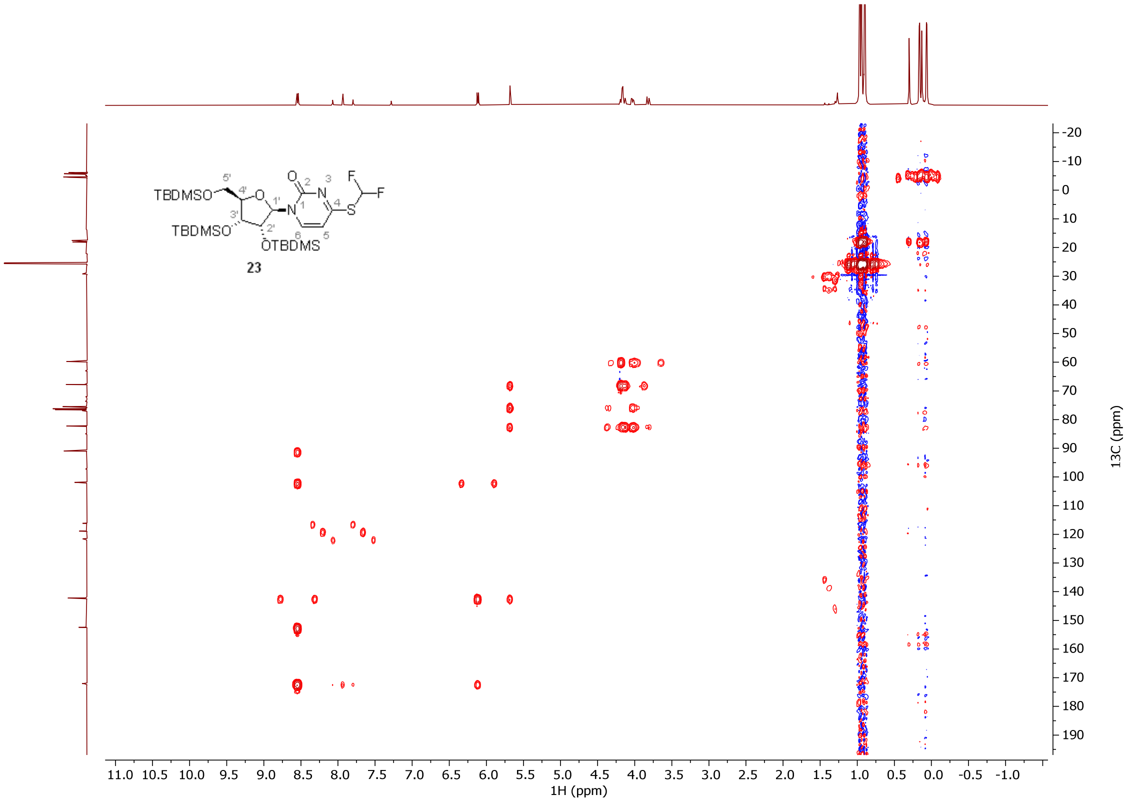

Figure S87 - ^{1}H-^{13}C HMBC NMR spectrum of **23** in $CDCl_3$

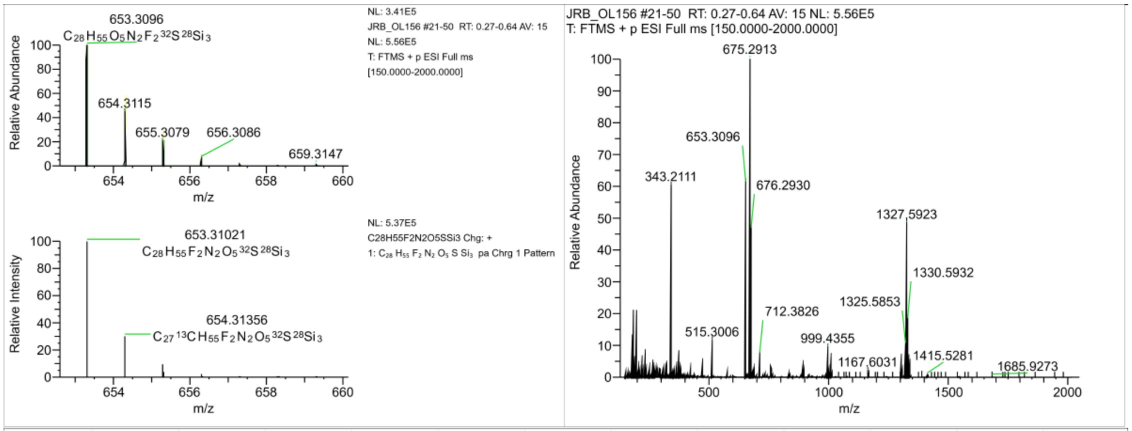

Peak Mass	Display For...	S Fit	RDB	Delta [ppm]	Theo. mass	Rank	Combined S...	# Matched I...	# Missed Iso.	MS Cov. [%]	Pattern Cov....	MSMS Matc...
653.3096	$C_{26}H_{53}O_4N_5F_2{}^{32}S^{28}Si_3$	74.4225425889549	5.00	1.11	653.30886	1	98.63	6	0	99.98	100	(Collection)
653.3096	$C_{27}H_{49}N_9F_2{}^{32}S^{28}Si_3$	71.8613942814085	10.00	-0.94	653.31020	2	98.5	6	0	99.98	100	(Collection)
653.3096	$C_{23}H_{54}O_5N_5F_3{}^{32}S^{28}Si_3$	70.5317797338945	1.00	-0.64	653.31001	3	98.43	6	0	99.98	100	(Collection)
653.3096	$C_{28}H_{55}O_5N_2F_2{}^{32}S^{28}Si_3$	61.8185141008261	4.50	-0.95	653.31021	4	97.97	6	0	99.98	100	(Collection)
653.3096	$C_{25}H_{57}O_8NF_2{}^{32}S^{28}Si_3$	61.1173248322141	0.00	3.15	653.30753	5	97.93	6	0	99.98	100	(Collection)
653.3096	$C_{24}H_{50}ON_9F_3{}^{32}S^{28}Si_3$	58.7366704991996	6.00	-2.69	653.31134	6	97.81	6	0	99.98	100	(Collection)
653.3096	$C_{21}H_{52}O_4N_8F_3{}^{32}S^{28}Si_3$	47.5437801804662	1.50	1.41	653.30866	7	97.01	6	0	99.75	99.83	(Collection)
653.3096	$C_{25}H_{56}O_6N_2F_3{}^{32}S^{28}Si_3$	41.2082535202712	0.50	-2.70	653.31135	8	96.67	6	0	99.75	99.79	(Collection)
653.3096	$C_{24}H_{51}O_3N_8F_2{}^{32}S^{28}Si_3$	40.2280965053854	5.50	3.16	653.30752	9	95.92	6	0	99.01	98.99	(Collection)
653.3096	$C_{29}H_{51}ON_6F_2{}^{32}S^{28}Si_3$	35.7717820942516	9.50	-3.00	653.31154	10	86.11	6	0	88.91	87.56	(Collection)

Figure S88 – HRMS spectrum of **23**

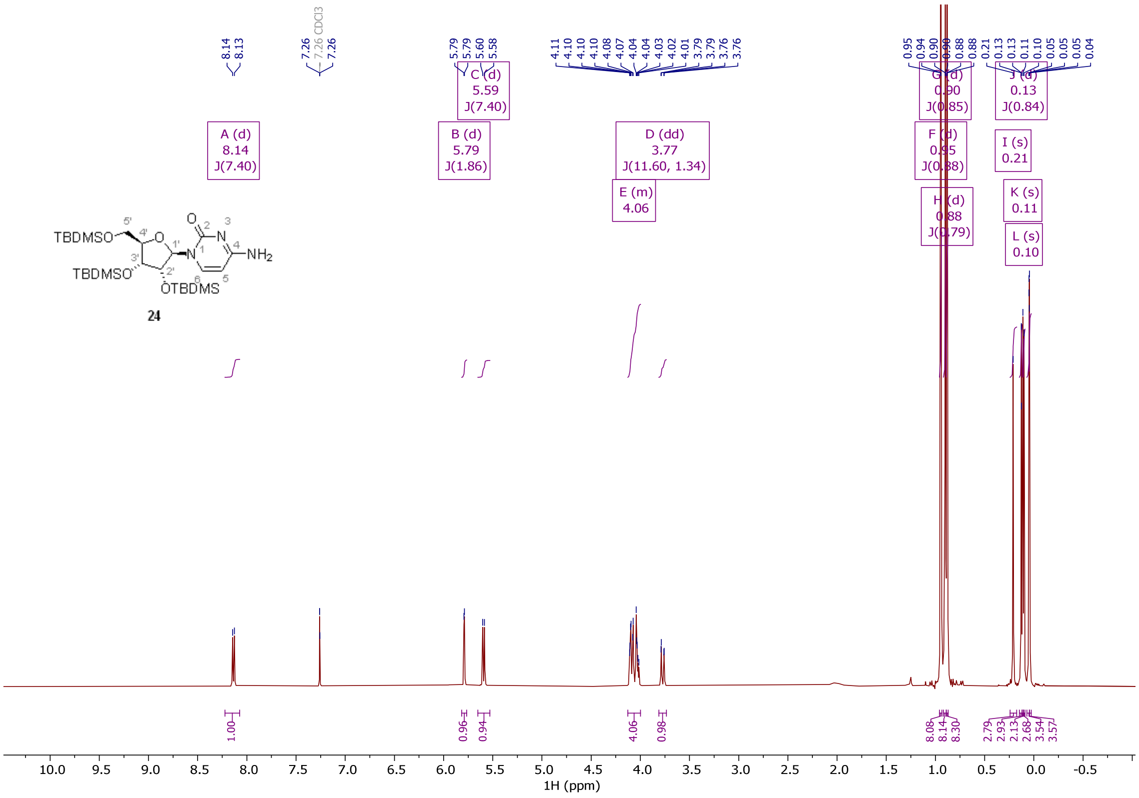

Figure S89 - ^{1}H NMR (400 MHz) spectrum of **24** in $CDCl_3$

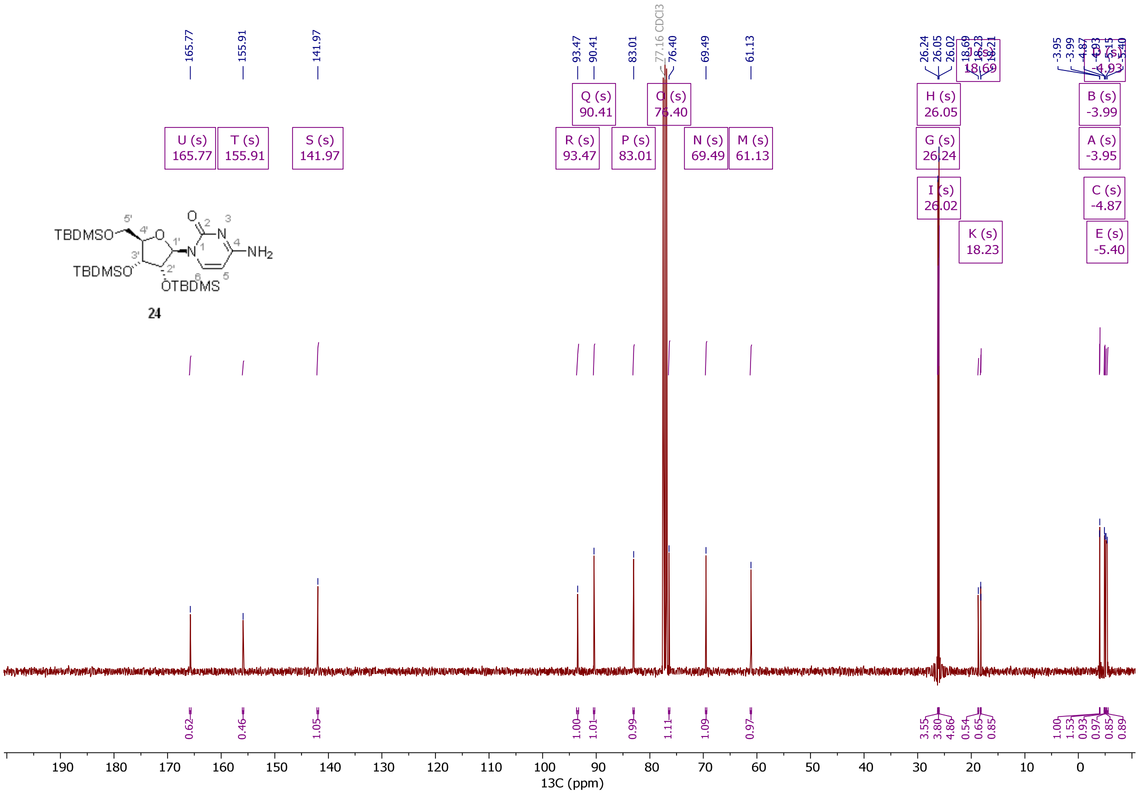

Figure S90 – ^{13}C NMR (101 MHz) spectrum of **24** in $CDCl_3$

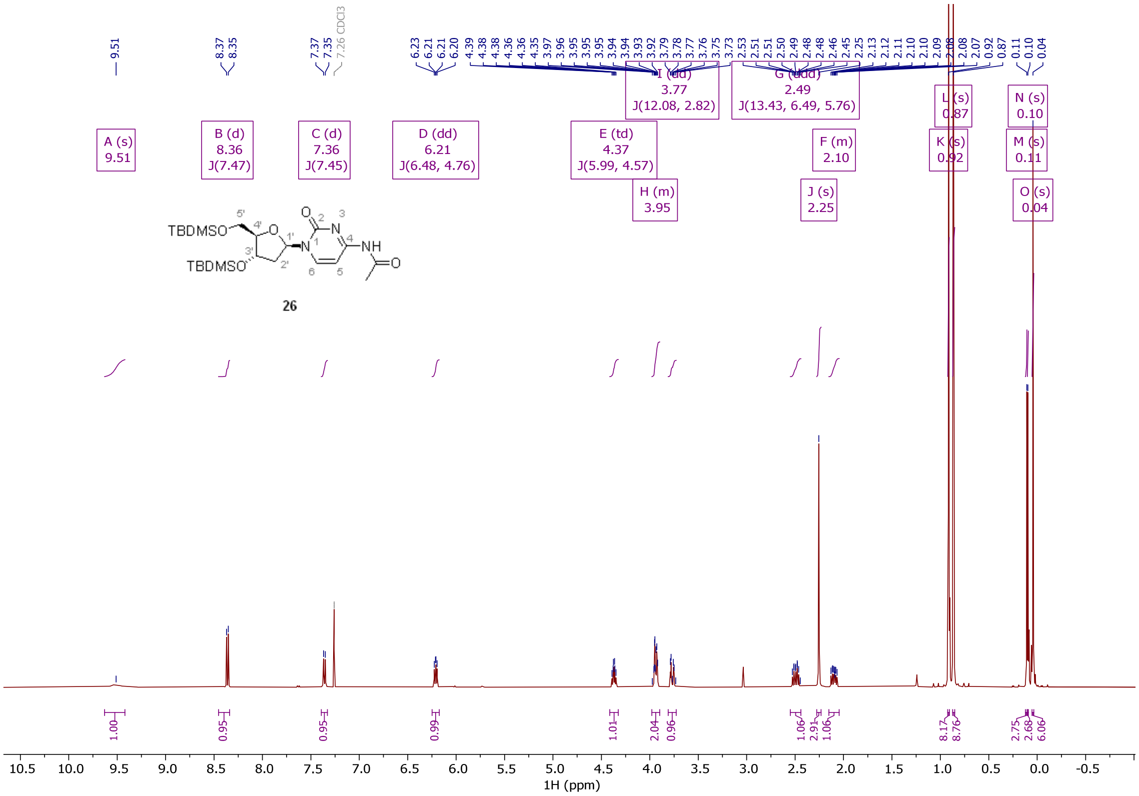

Figure S91 - ^{1}H NMR (400 MHz) spectrum of **25** in $CDCl_3$

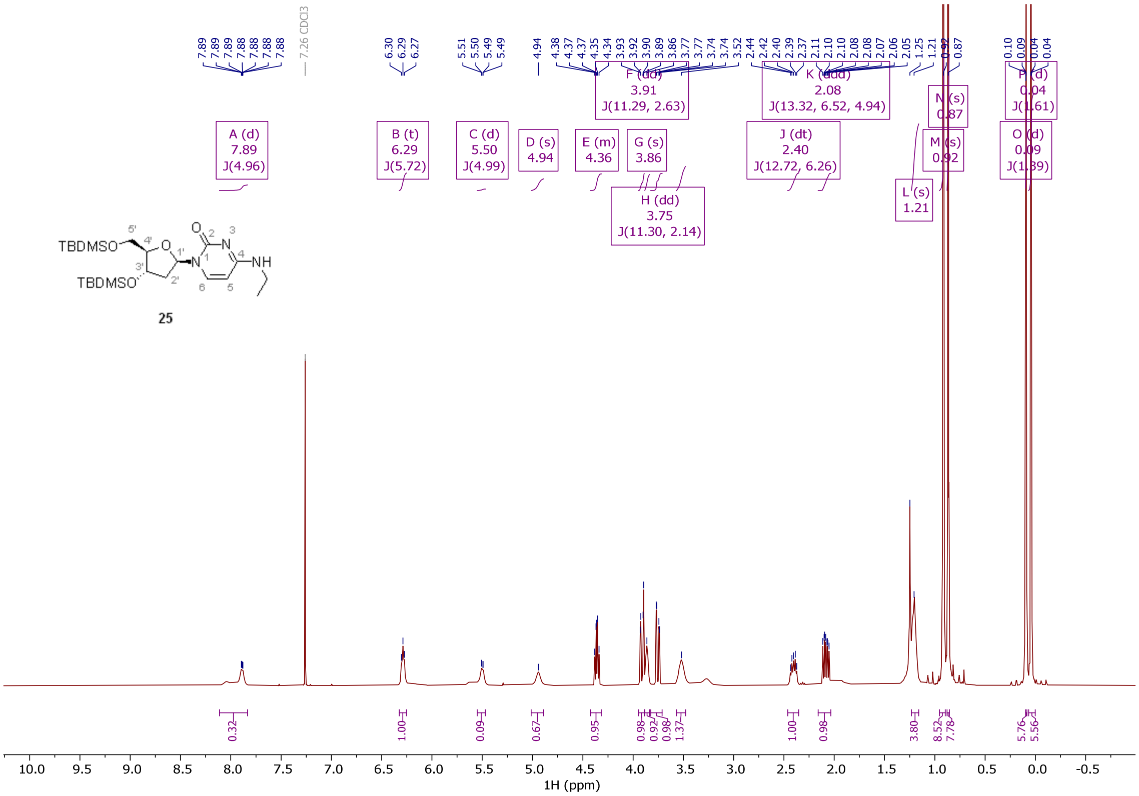

Figure S92 - ^{1}H NMR (400 MHz) spectrum of **25** in $CDCl_3$

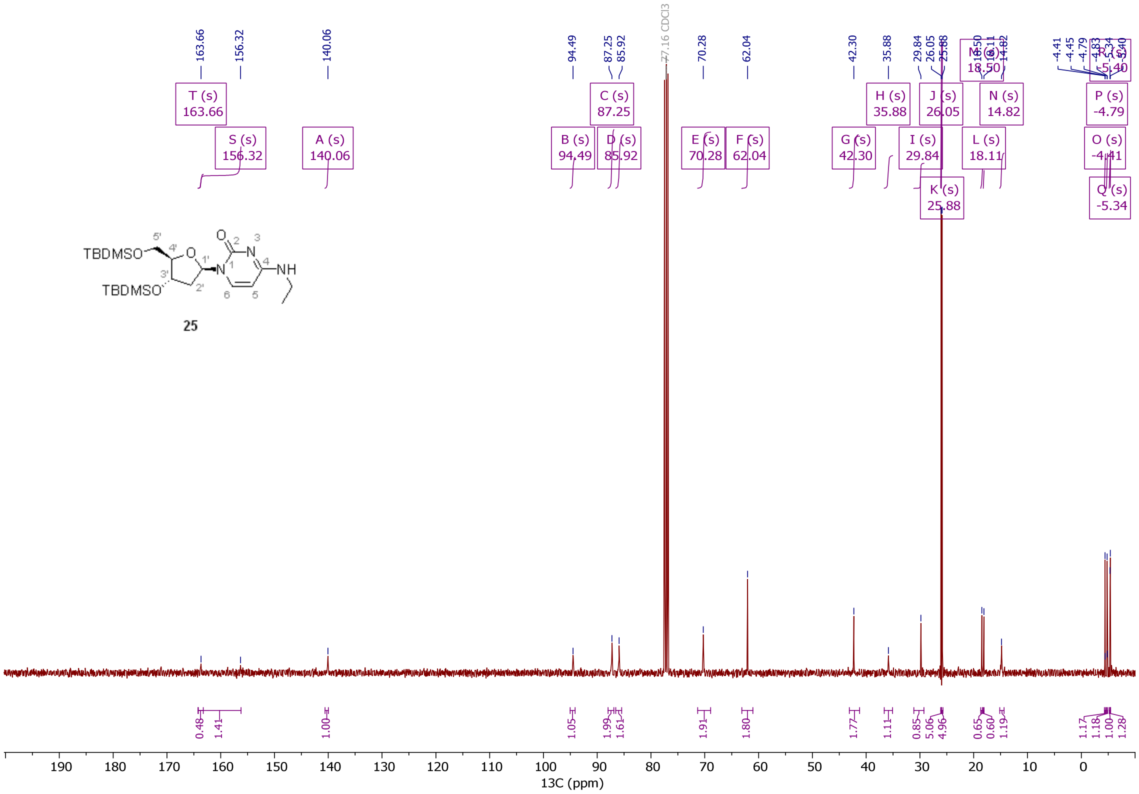

Figure S93 - ^{13}C NMR (101 MHz) spectrum of **25** in $CDCl_3$

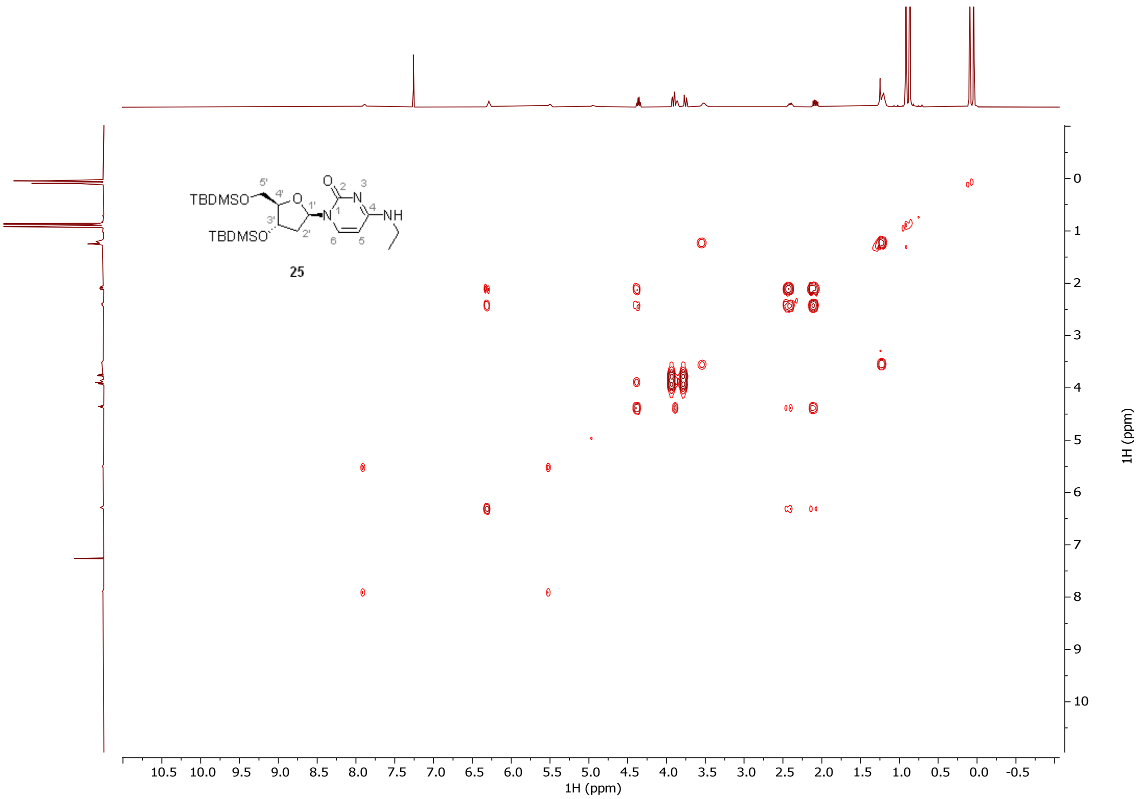

Figure S94 - ^{1}H-^{1}H COSY NMR spectrum of **25** in $CDCl_3$

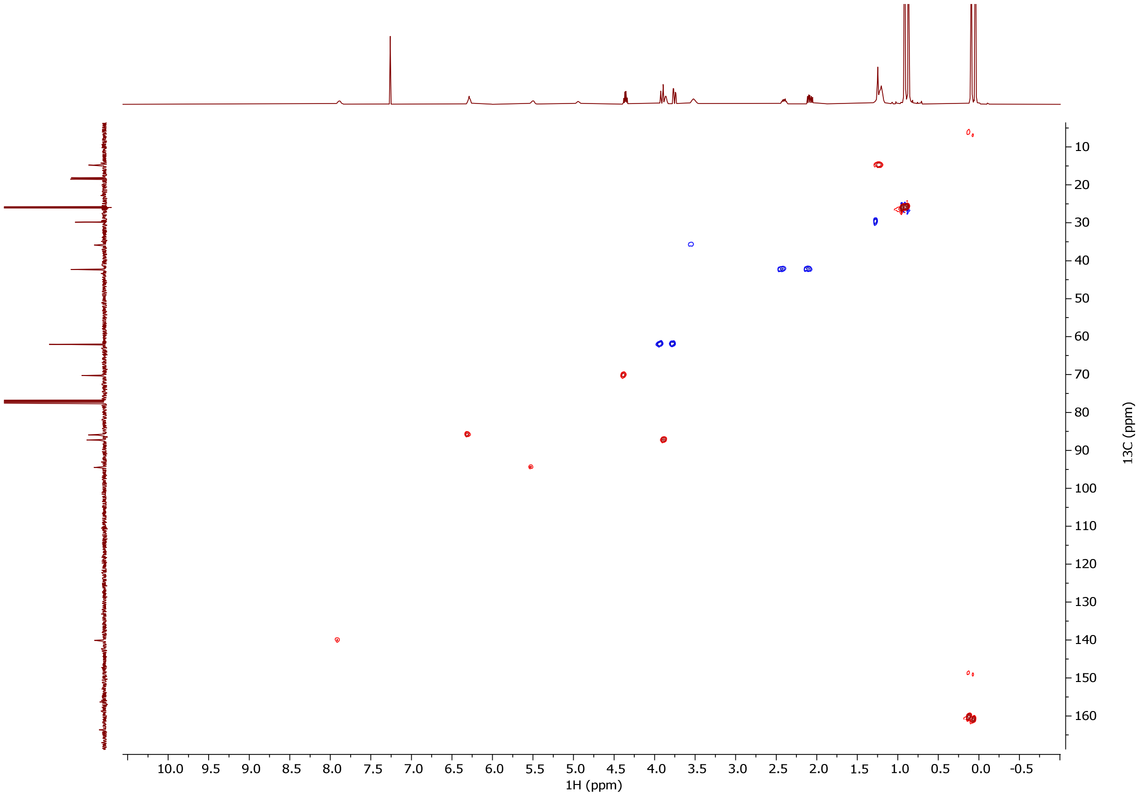

Figure S95 - ^{1}H-^{13}C HSQC NMR spectrum of **25** in $CDCl_3$

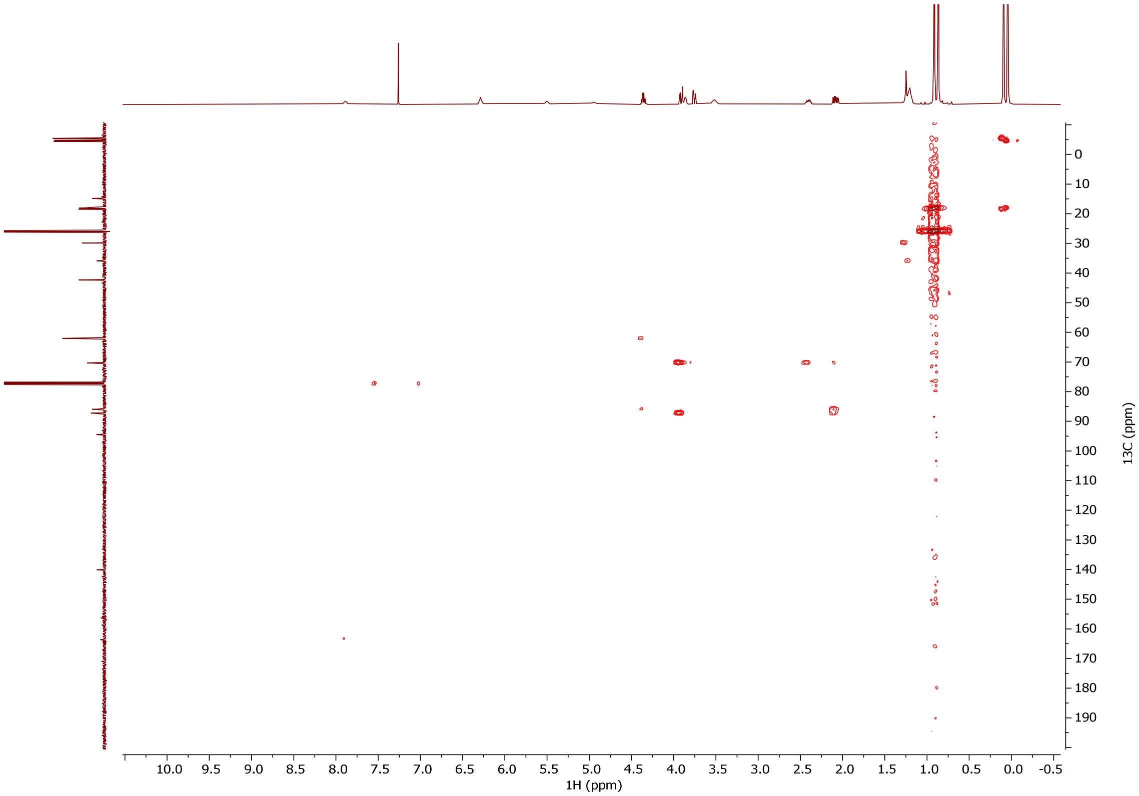

Figure S96 - ^{1}H-^{13}C HMBC NMR spectrum of **25** in $CDCl_3$

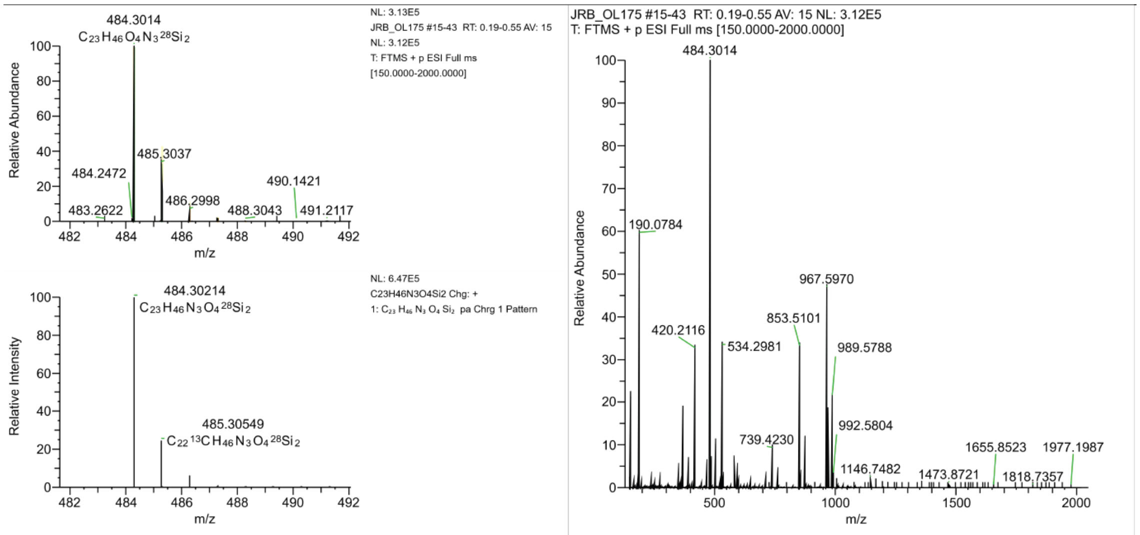

Peak Mass	Display For...	S Fit	RDB	Delta [ppm]	Theo. mass	Rank	Combined S...	# Matched I...	# Missed Iso.	MS Cov. [%]	Pattern Cov....	MSMS Matc...
484.3014	$C_{21}H_{44}O_3N_6{}^{28}Si_2$	32.583516310764	5.00	1.24	484.30079	1	91.9	5	0	95.2	93.47	(Collection)
484.3014	$C_{20}H_{48}O_7N_2{}^{28}Si_2$	25.096157063748	0.00	4.00	484.29946	2	91.51	5	0	95.2	93.51	(Collection)
484.3014	$C_{19}H_{42}O_2N_9{}^{28}Si_2$	23.0214557971907	5.50	4.01	484.29945	3	91.4	5	0	95.2	93.41	(Collection)
484.3014	$C_{23}H_{46}O_4N_3{}^{28}Si_2$	21.2328491527782	4.50	-1.54	484.30214	4	90.1	5	0	93.93	91.95	(Collection)
484.3014	$C_{24}H_{42}N_7{}^{28}Si_2$	13.5206455764894	9.50	-4.30	484.30347	5	89.7	5	0	93.93	91.84	(Collection)
484.3014	$C_{25}H_{48}O_5{}^{28}Si_2$	12.3228039354135	4.00	-4.31	484.30348	6	89.64	5	0	93.93	91.9	(Collection)

Figure S97 – HRMS spectrum of **25**

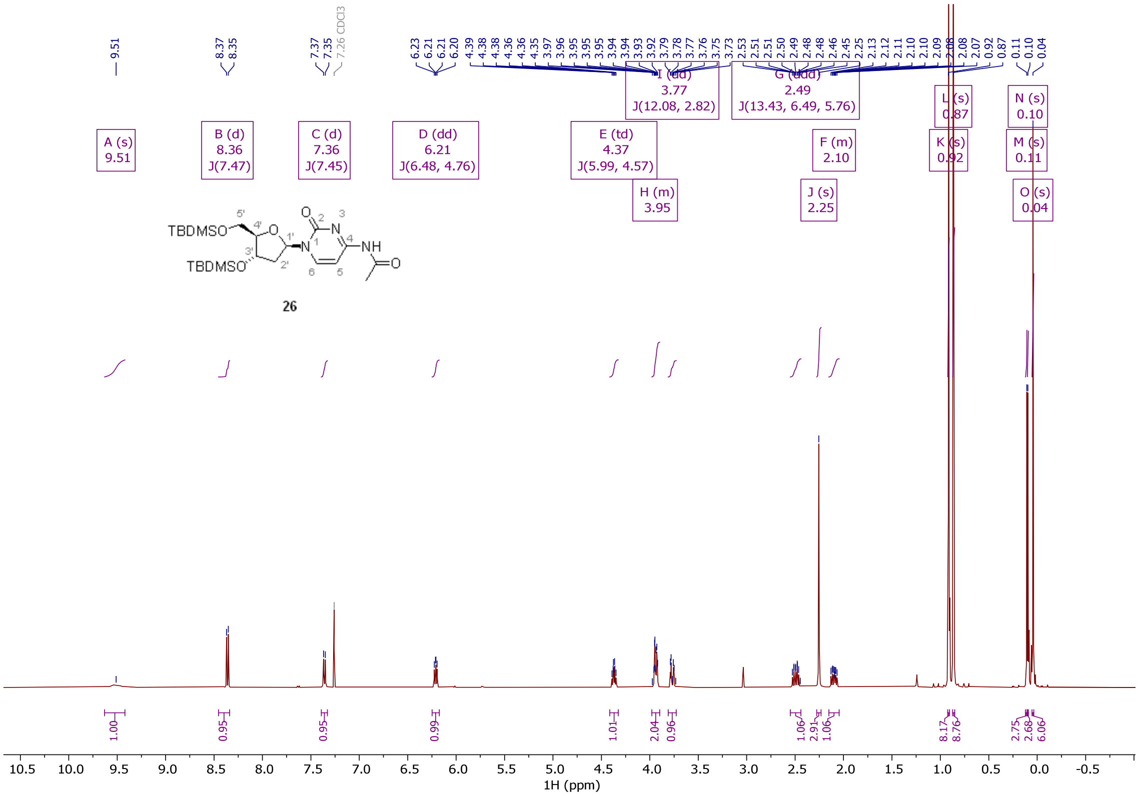

Figure S98 - ^{1}H NMR (400 MHz) spectrum of **26** in $CDCl_3$

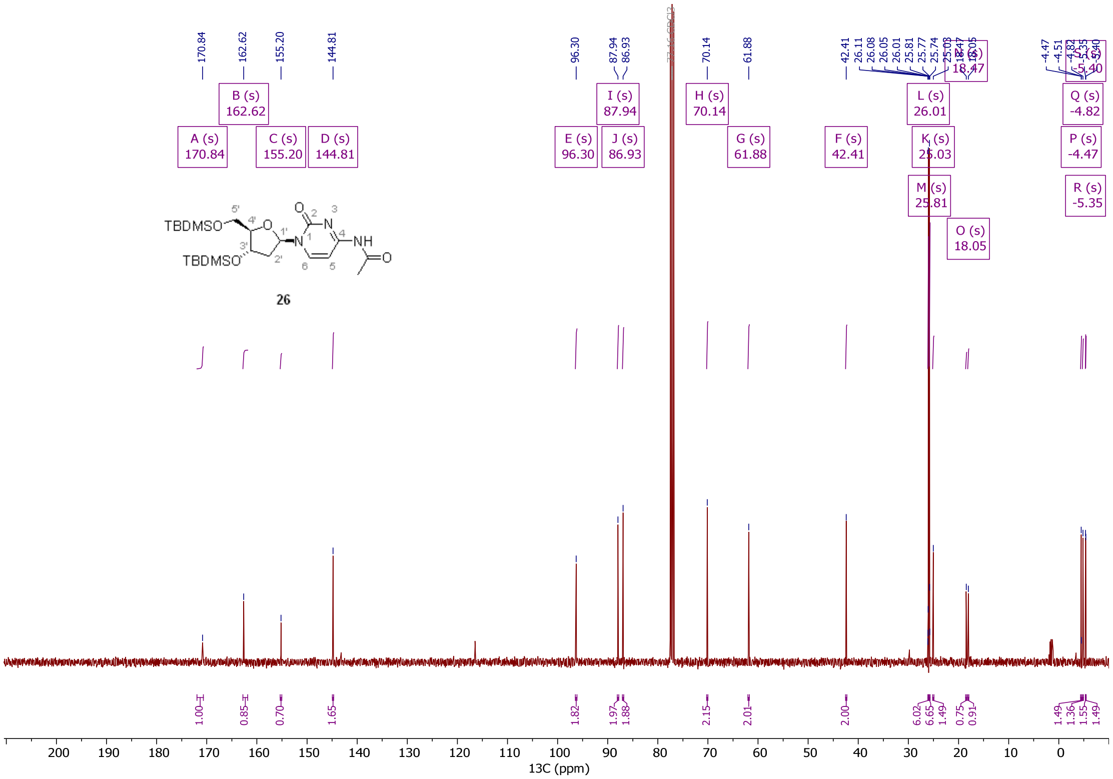

Figure S99 – ^{13}C NMR (101 MHz) spectrum of **26** in $CDCl_3$

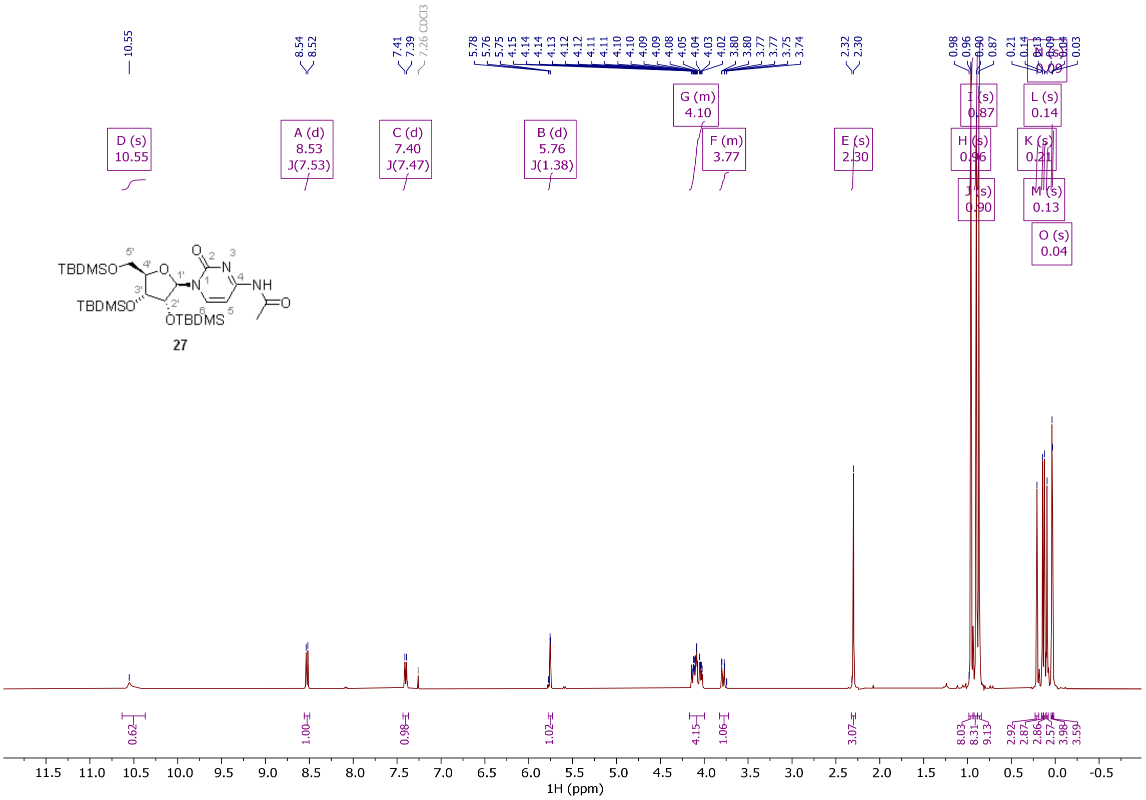

Figure S100 - ^{1}H NMR (400 MHz) spectrum of **27** in $CDCl_3$

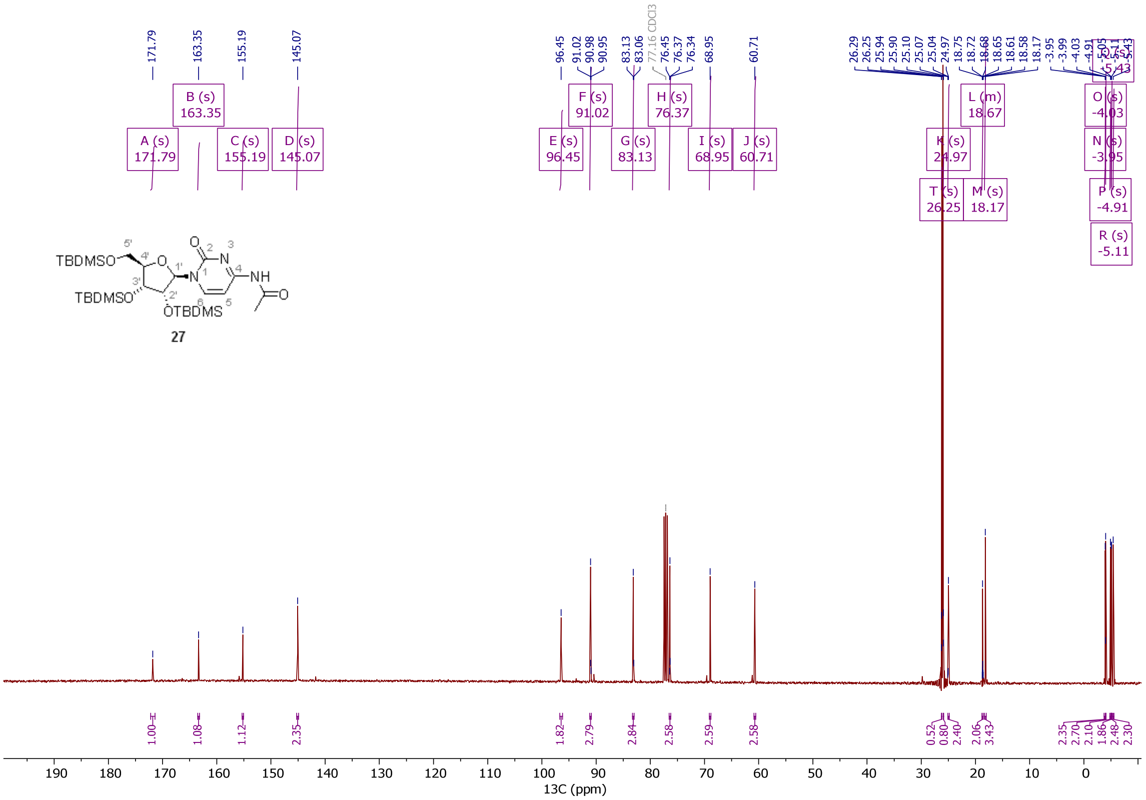

Figure S101 - ^{13}C NMR (101 MHz) spectrum of **27** in $CDCl_3$

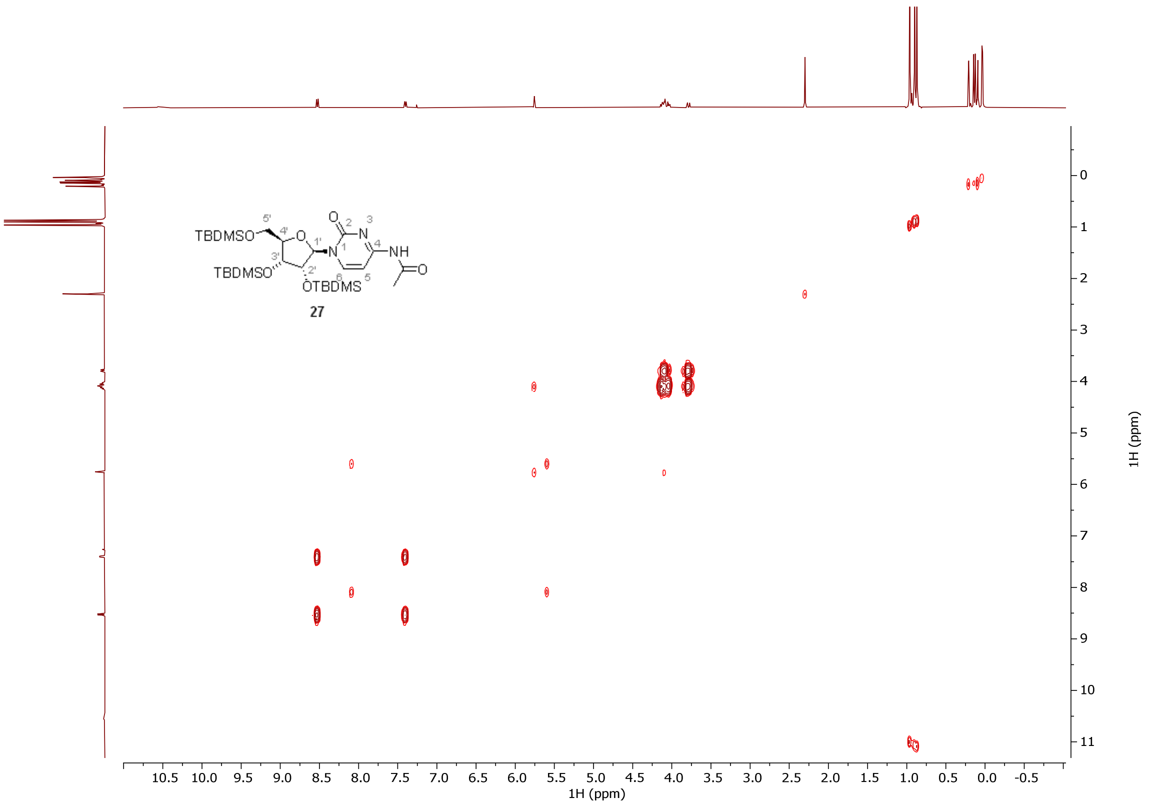

Figure S102 - ^{1}H-^{1}H COSY NMR spectrum of **27** in $CDCl_3$

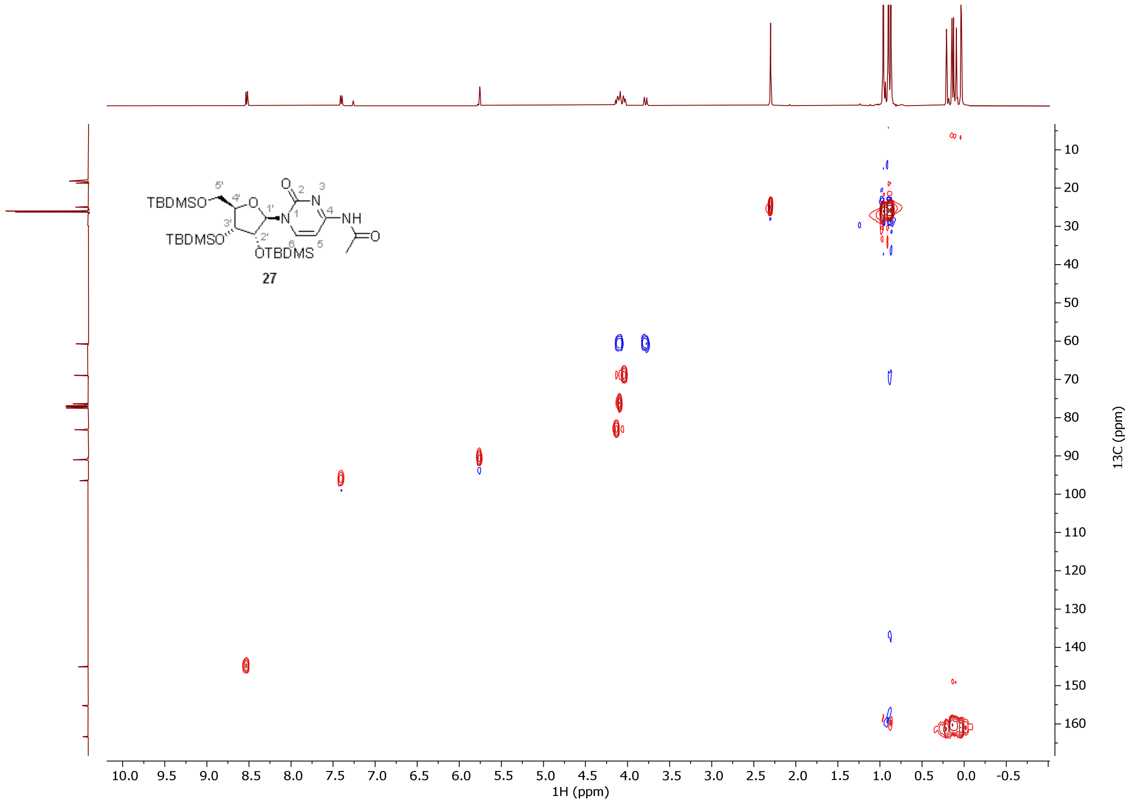

Figure S103 - ^{1}H-^{13}C HSQC NMR spectrum of **27** in $CDCl_3$

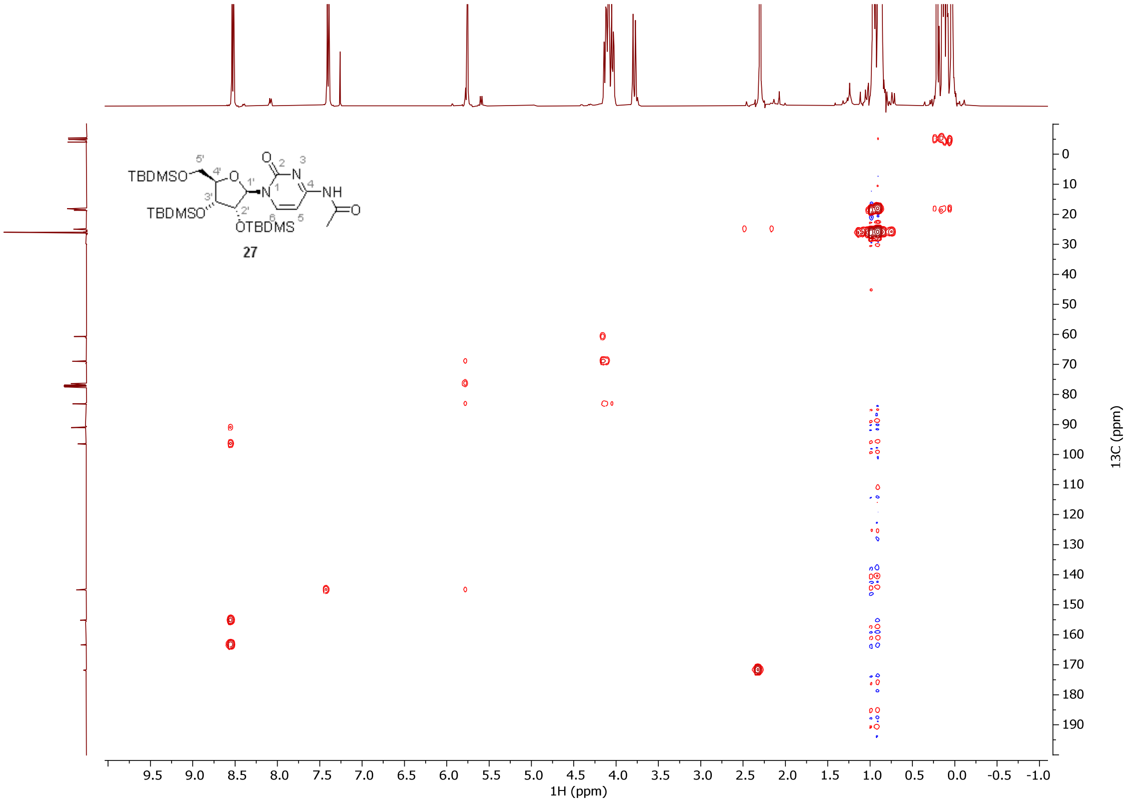

Figure S104 - ^{1}H-^{13}C HMBC NMR spectrum of **27** in $CDCl_3$

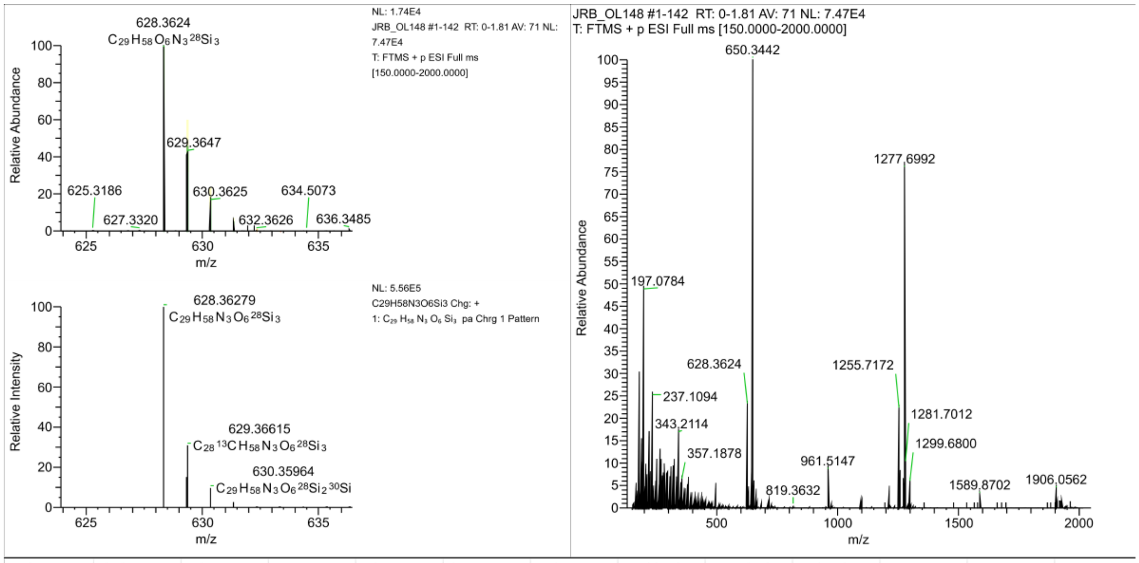

Peak Mass	Display For...	S Fit	RDB	Delta [ppm]	Theo. mass	Rank	Combined S...	# Matched I...	# Missed Iso.	MS Cov. [%]	Pattern Cov....	MSMS Matc...
628.3624	$C_{28}H_{52}ON_{10}{}^{2}$ $^{8}Si_3$	48.41563777 40183	11.00	-0.69	628.36279	1	96.88	5	0	99.57	99.34	(Collection)
628.3624	$C_{29}H_{58}O_6N_3{}^{2}$ $^{8}Si_3$	36.26602765 74621	5.50	-0.70	628.36279	2	96.24	5	1	99.57	99.17	(Collection)
628.3624	$C_{30}H_{54}O_2N_7{}^{2}$ $^{8}Si_3$	35.47928838 08077	10.50	-2.83	628.36413	3	96.2	5	0	99.57	99.29	(Collection)
628.3624	$C_{29}H_{48}O_4N_{10}$ ^{28}Si	21.50255027 97701	12.00	-0.04	628.36238	4	83.92	4	1	87.38	91.97	(Collection)
628.3624	$C_{30}H_{54}O_9N_3{}^{2}$ ^{8}Si	21.11850826 73455	6.50	-0.05	628.36238	5	83.9	5	0	87.38	91.38	(Collection)
628.3624	$C_{28}H_{52}O_8N_6{}^{2}$ ^{8}Si	18.71753509 6734	7.00	2.09	628.36104	6	83.77	5	0	87.38	91.8	(Collection)

Figure S105 - HRMS spectrum of **27**

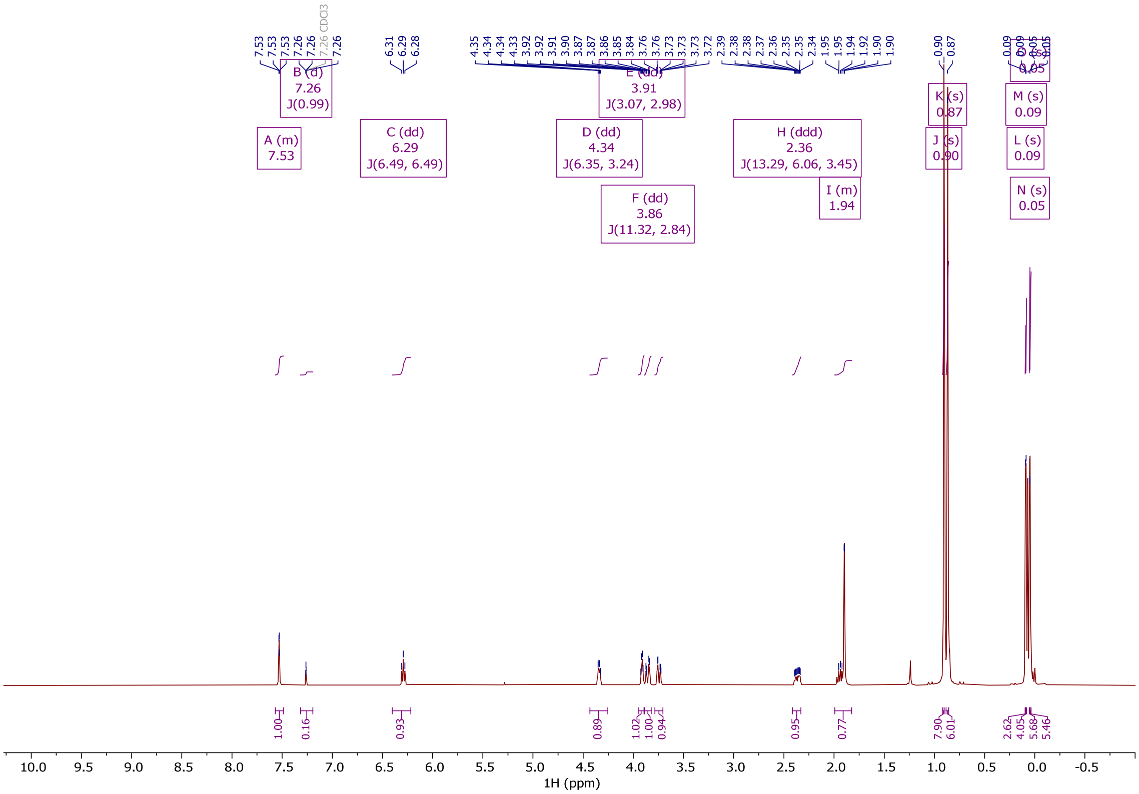

Figure S106 - ^{1}H NMR (400 MHz) spectrum of **28** in $CDCl_3$

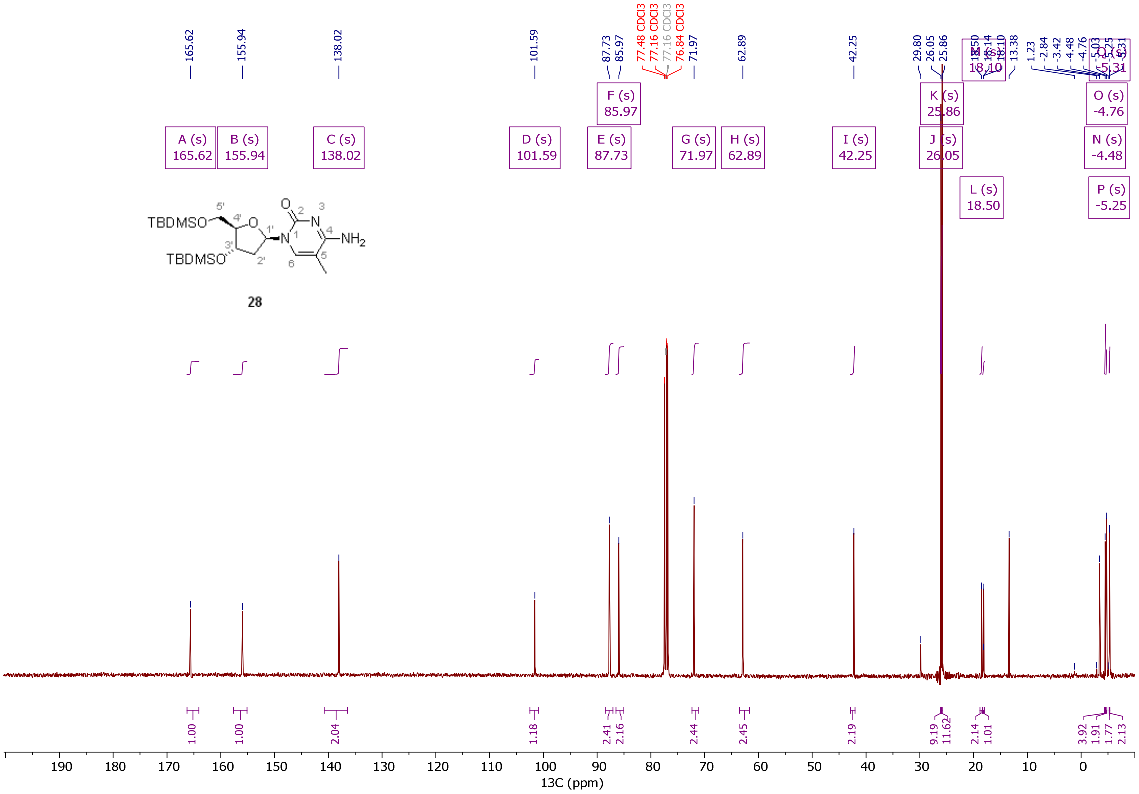

Figure S107 – ^{13}C NMR (101 MHz) spectrum of **28** in $CDCl_3$

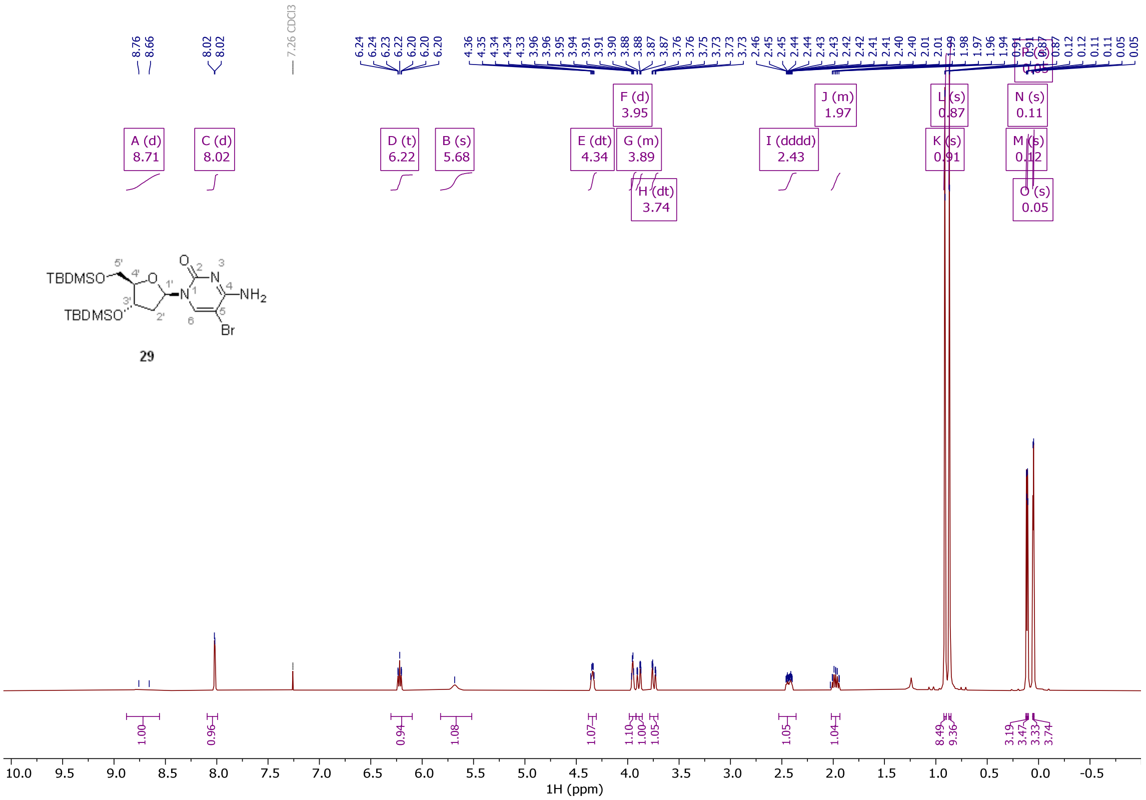

Figure S108 - ^{1}H NMR (400 MHz) spectrum of **29** in $CDCl_3$

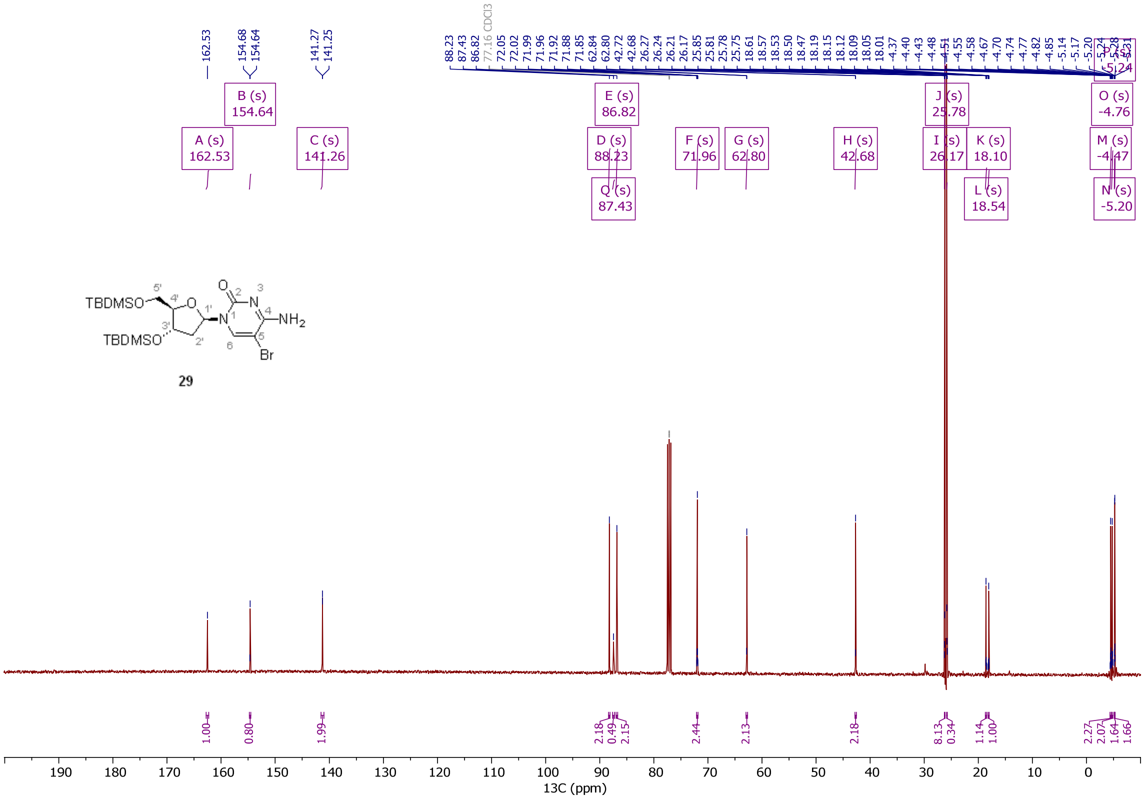

Figure S109 - ^{13}C NMR (101 MHz) spectrum of **29** in $CDCl_3$

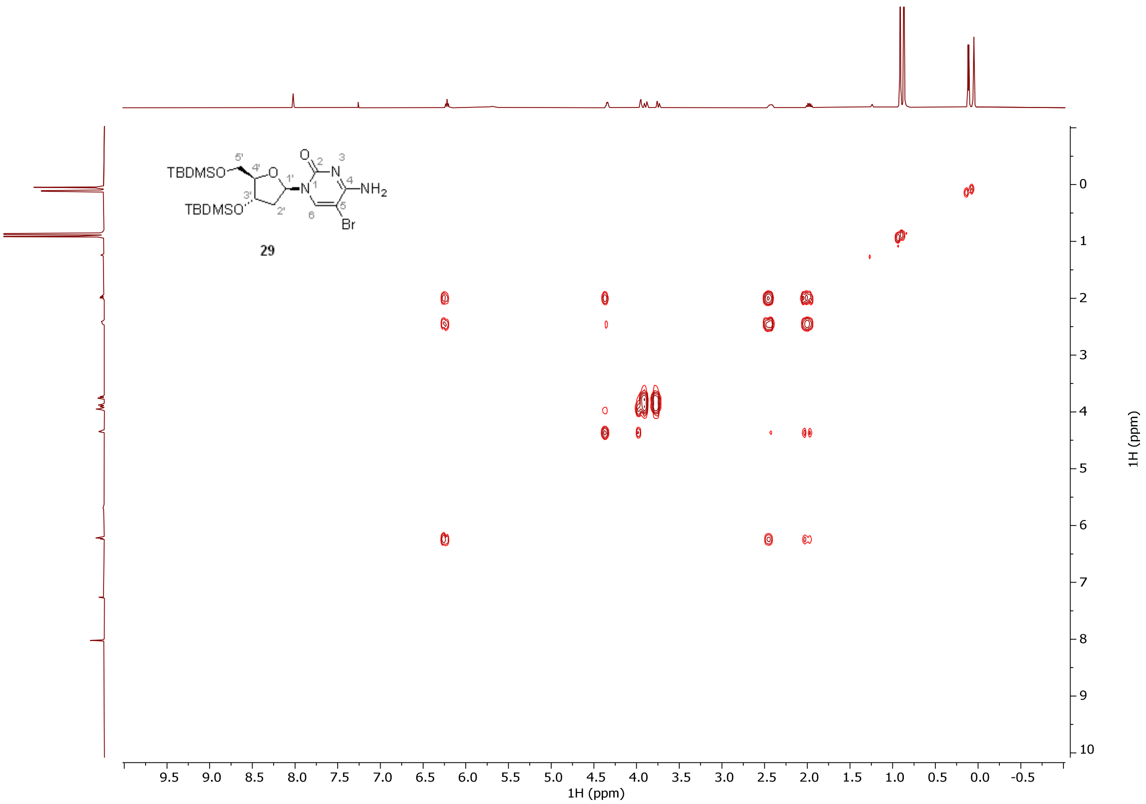

Figure S110 - ^{1}H-^{1}H COSY NMR spectrum of **29** in $CDCl_3$

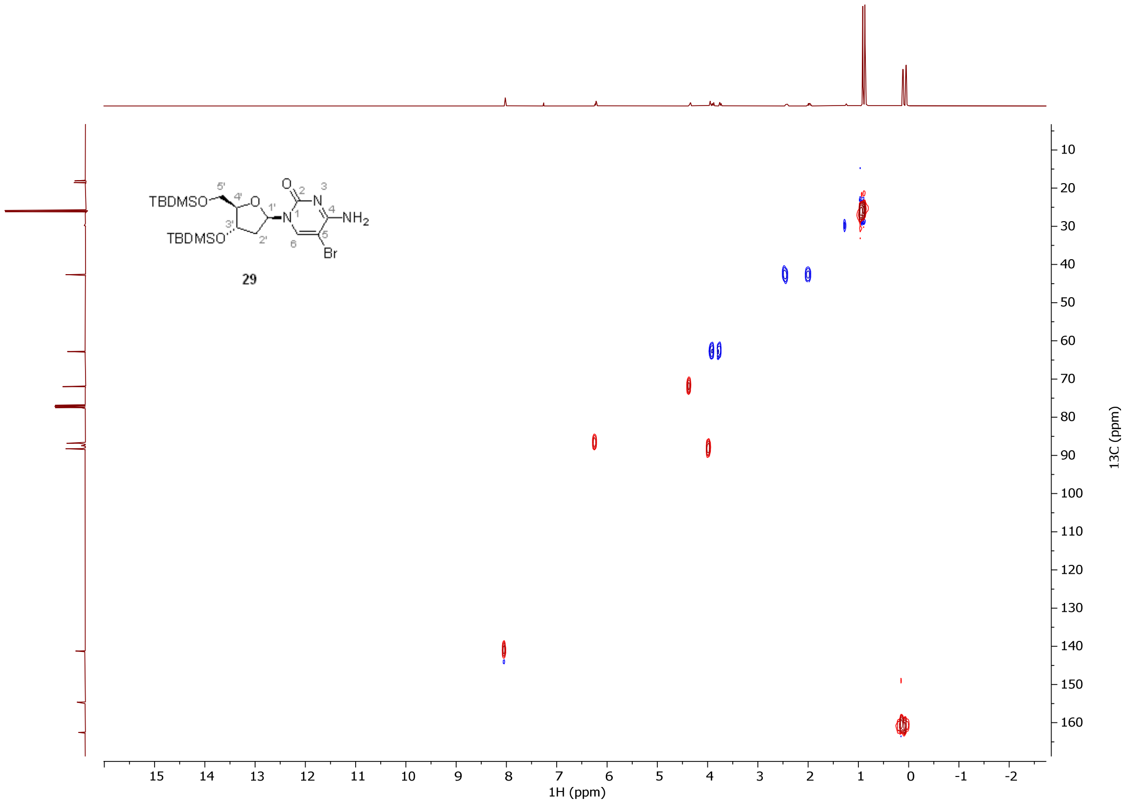

Figure S111 - ^{1}H-^{13}C HSQC NMR spectrum of **29** in $CDCl_3$

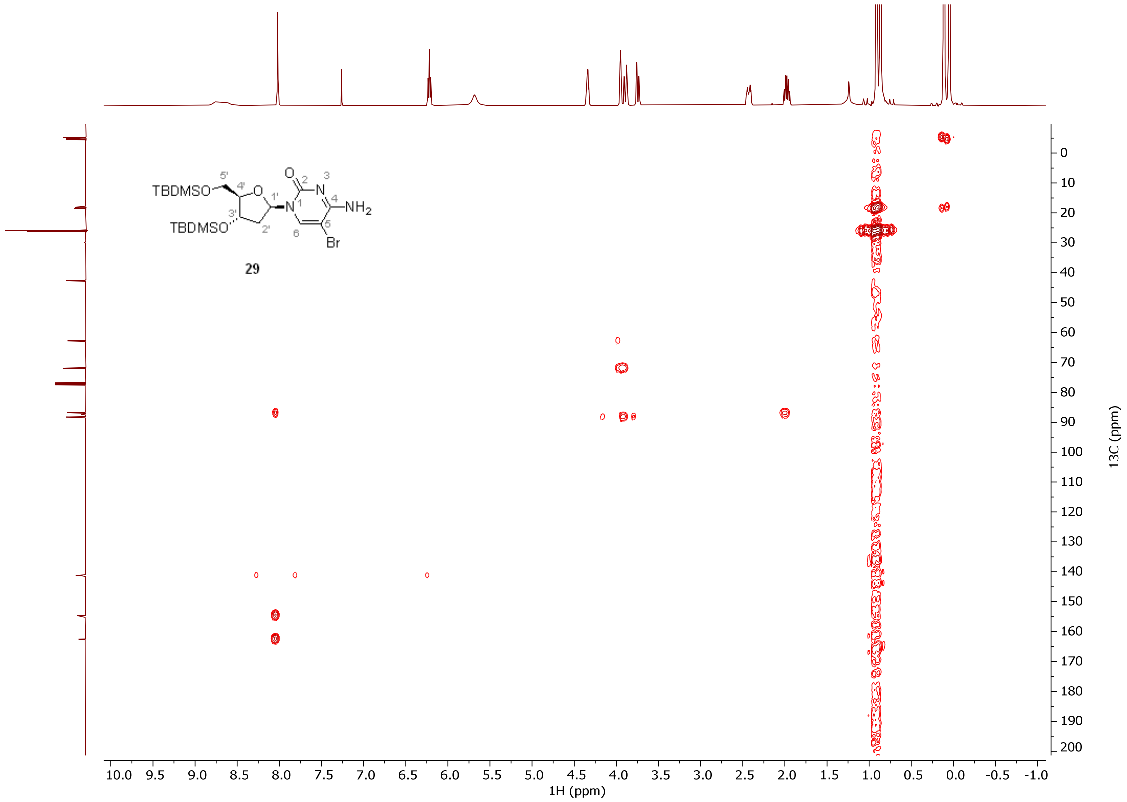

Figure S112 - ^{1}H-^{13}C HMBC NMR spectrum of **29** in $CDCl_3$

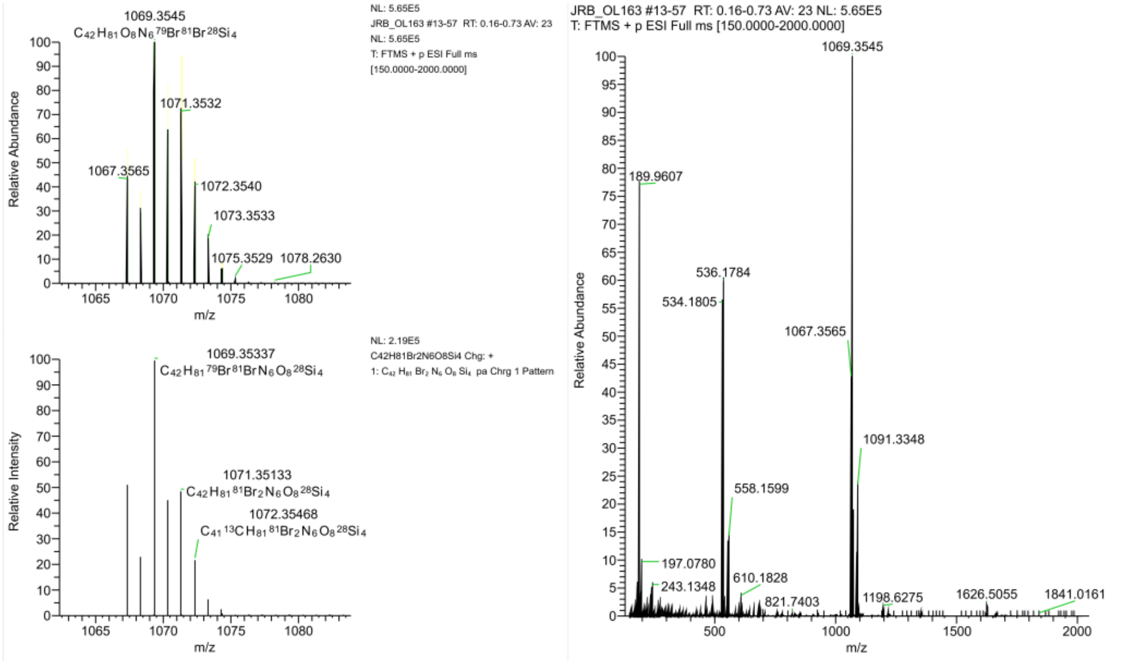

Peak Mass	Display For...	S Fit	RDB	Delta [ppm]	Theo. mass	Rank	Combined S...	# Matched I...	# Missed Iso.	MS Cov. [%]	Pattern Cov....	MSMS Matc...
1069.3545	$C_{42}H_{81}O_8N_6{}^{79}Br^{81}Br^{28}Si_4$	89.43955166 37394	8.50	1.04	1069.35337	1	99.43	10	0	99.99	100	(Collection)
1069.3545	$C_{42}H_{81}O_7N_7{}^{81}Br_2{}^{28}Si_4$	0	9.00	-4.68	1069.35949	2	0	4	6	0	0	(Collection)

Figure S113 - HRMS spectrum of **29**

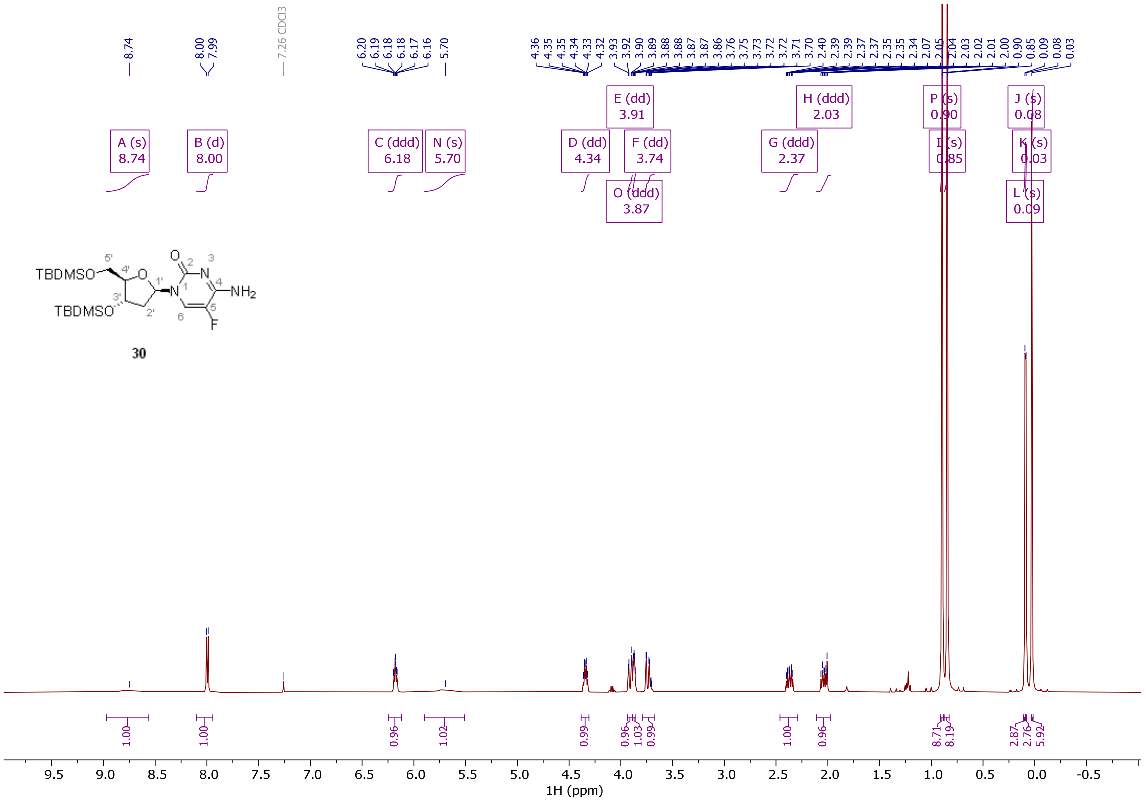

Figure S114 - ^{1}H NMR (400 MHz) spectrum of **30** in $CDCl_3$

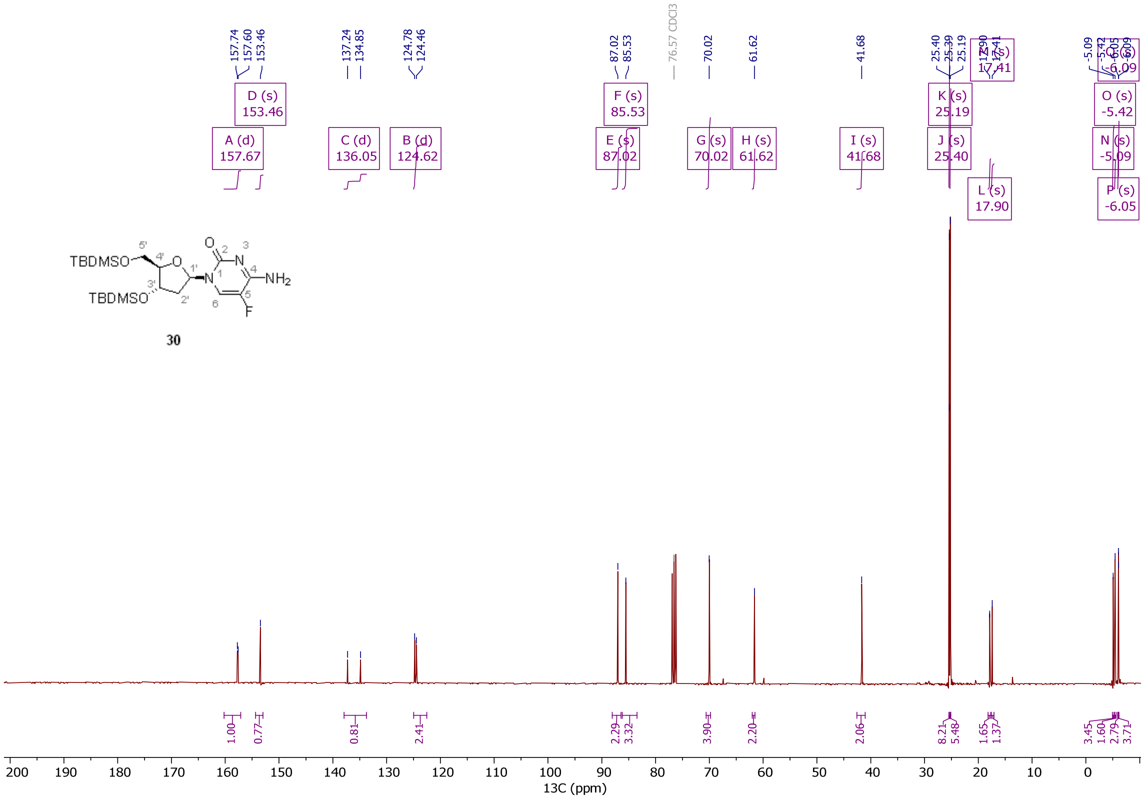

Figure S115 - ^{13}C NMR (101 MHz) spectrum of **30** in $CDCl_3$

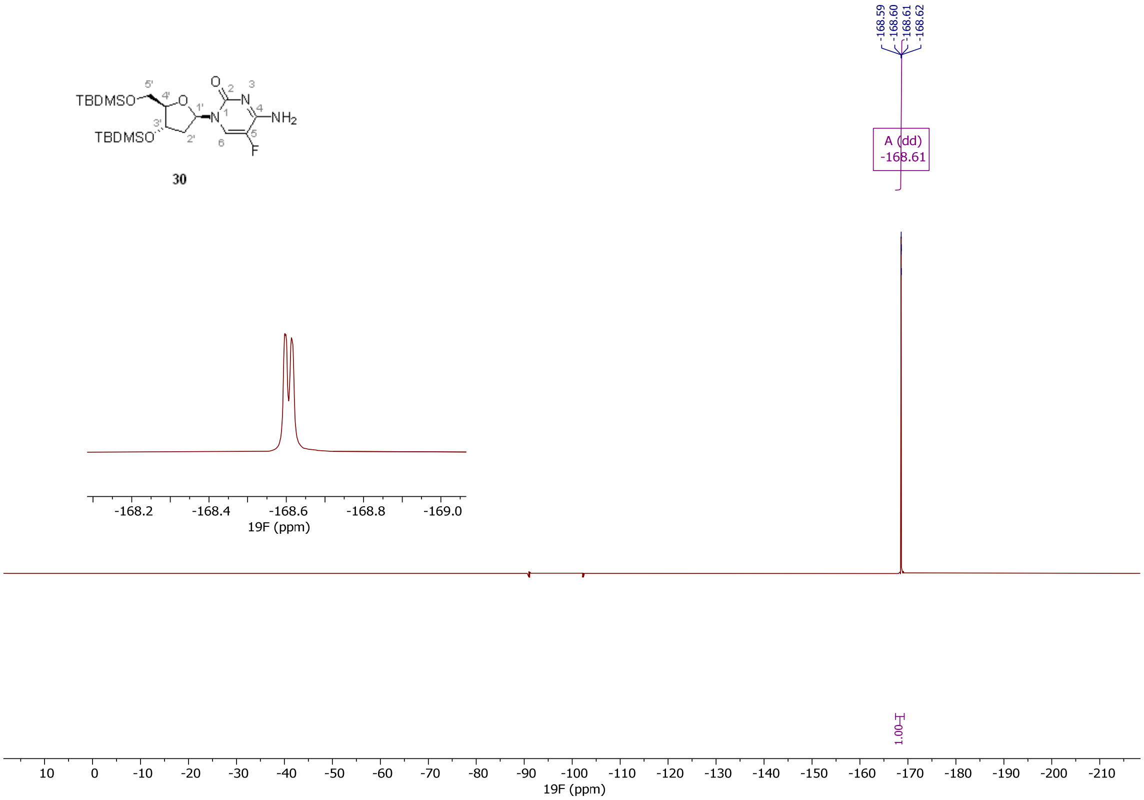

Figure S116 - ^{19}F NMR (376 MHz) spectrum of **30** in $CDCl_3$

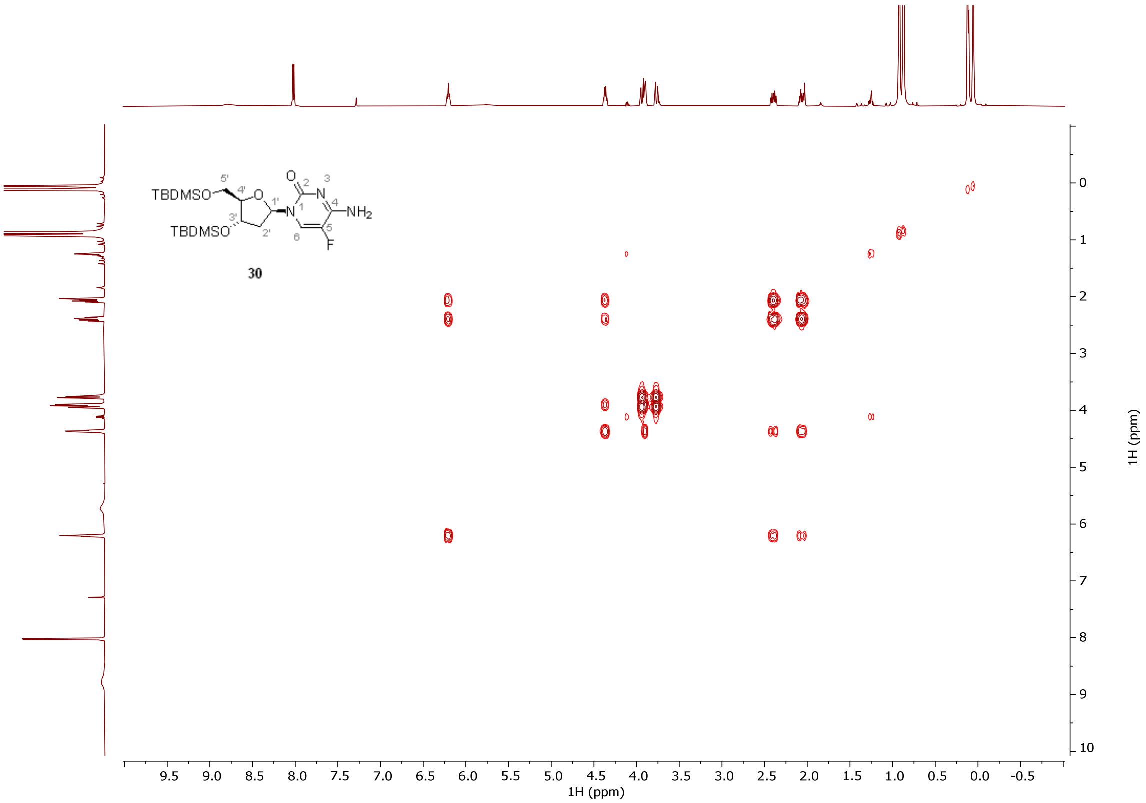

Figure S117 - ^{1}H-^{1}H COSY NMR spectrum of **30** in $CDCl_3$

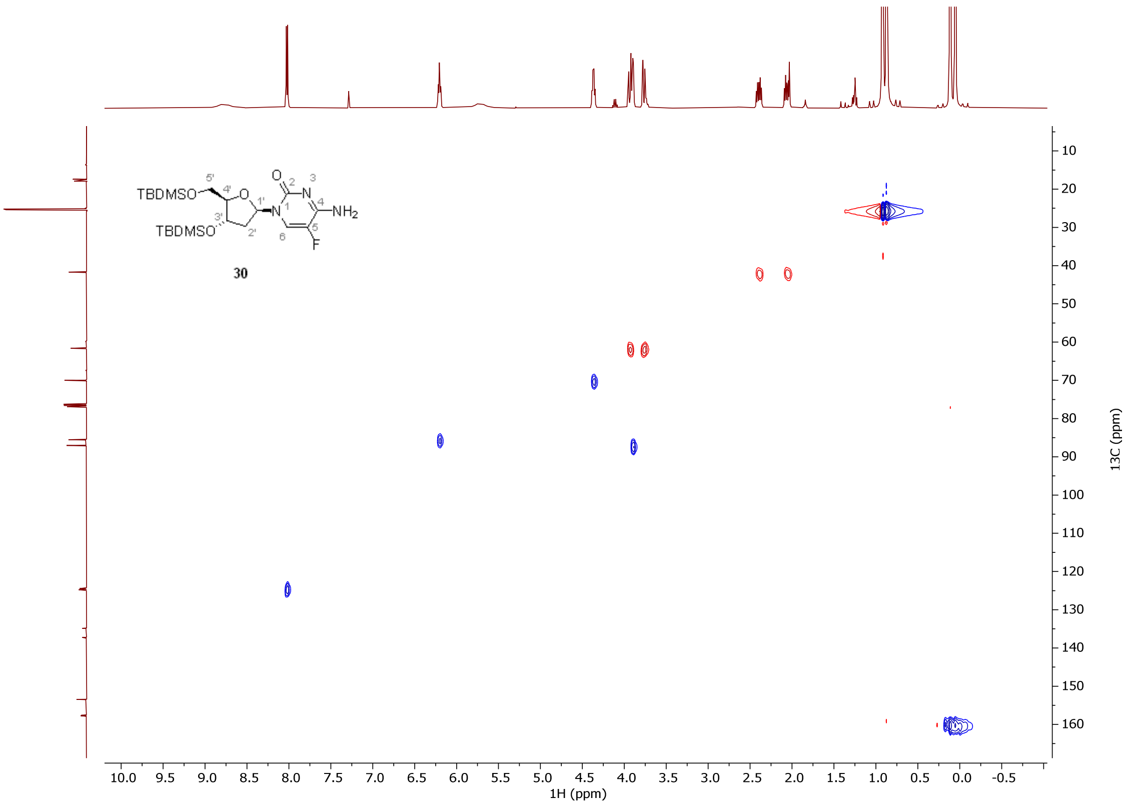

Figure S118 - ^{1}H-^{13}C HSQC NMR spectrum of **30** in $CDCl_3$

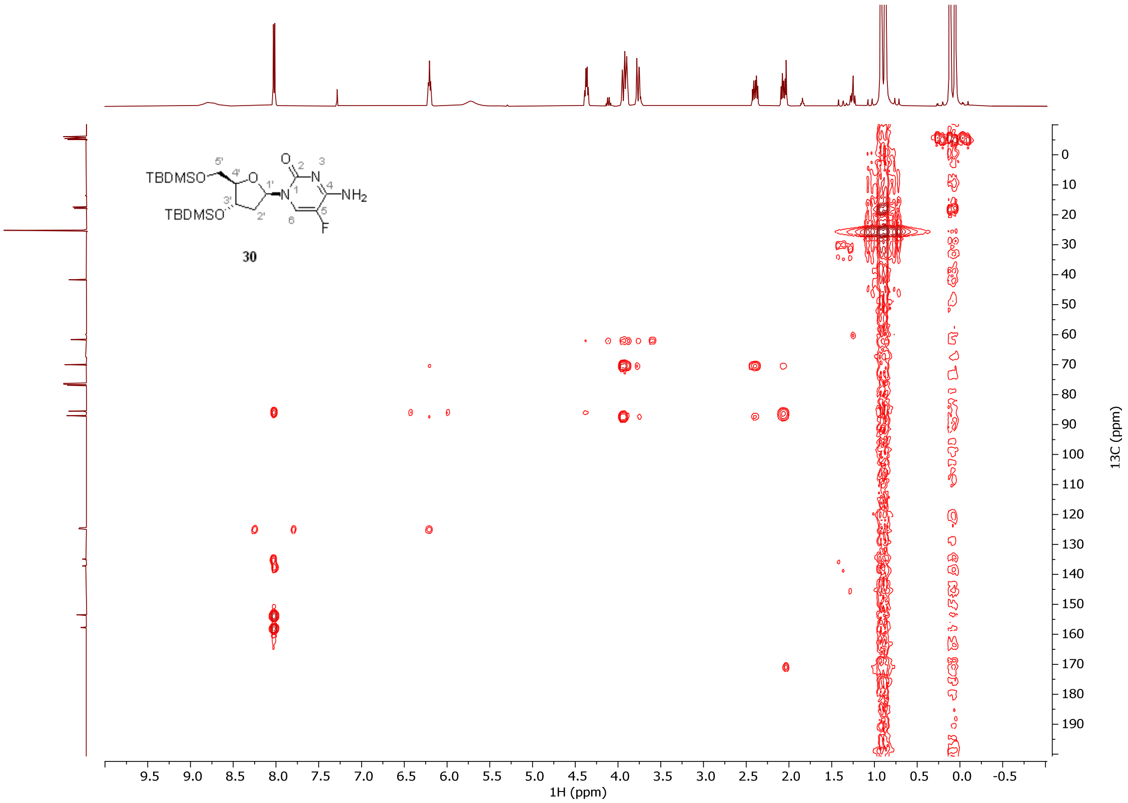

Figure S119 - ^{1}H-^{13}C HMBC NMR spectrum of **30** in $CDCl_3$

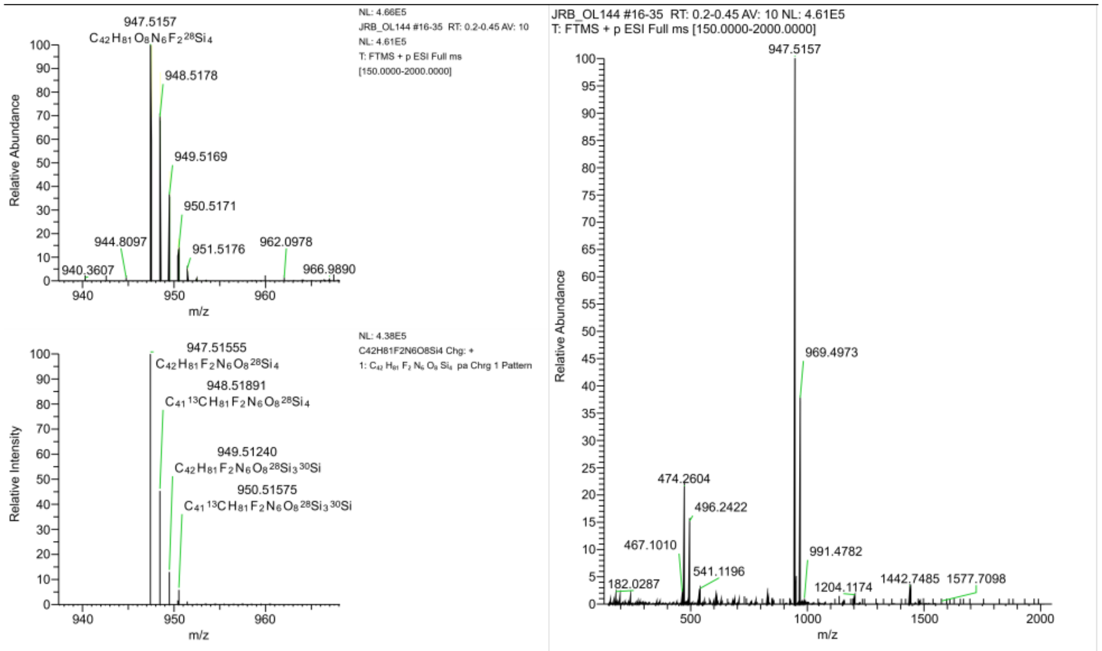

Peak Mass	Display For...	S Fit	RDB	Delta [ppm]	Theo. mass	Rank	Combined S...	# Matched I...	# Missed Iso.	MS Cov. [%]	Pattern Cov....	MSMS Matc...
947.5157	$C_{42}H_{81}O_8N_6F_2{}^{28}Si_4$	86.18229748 77496	8.50	0.10	947.51555	1	99.25	7	0	99.97	100	(Collection)
947.5157	$C_{45}H_{79}O_5N_7F_2{}^{28}Si_4$	55.35119473 76281	13.00	-2.73	947.51823	2	97.63	7	0	99.97	100	(Collection)
947.5157	$C_{40}H_{79}O_7N_9F_2{}^{28}Si_4$	56.41175717 56881	9.00	1.52	947.51421	3	97.59	7	0	99.87	99.93	(Collection)

Figure S120 - HRMS spectrum of **30**

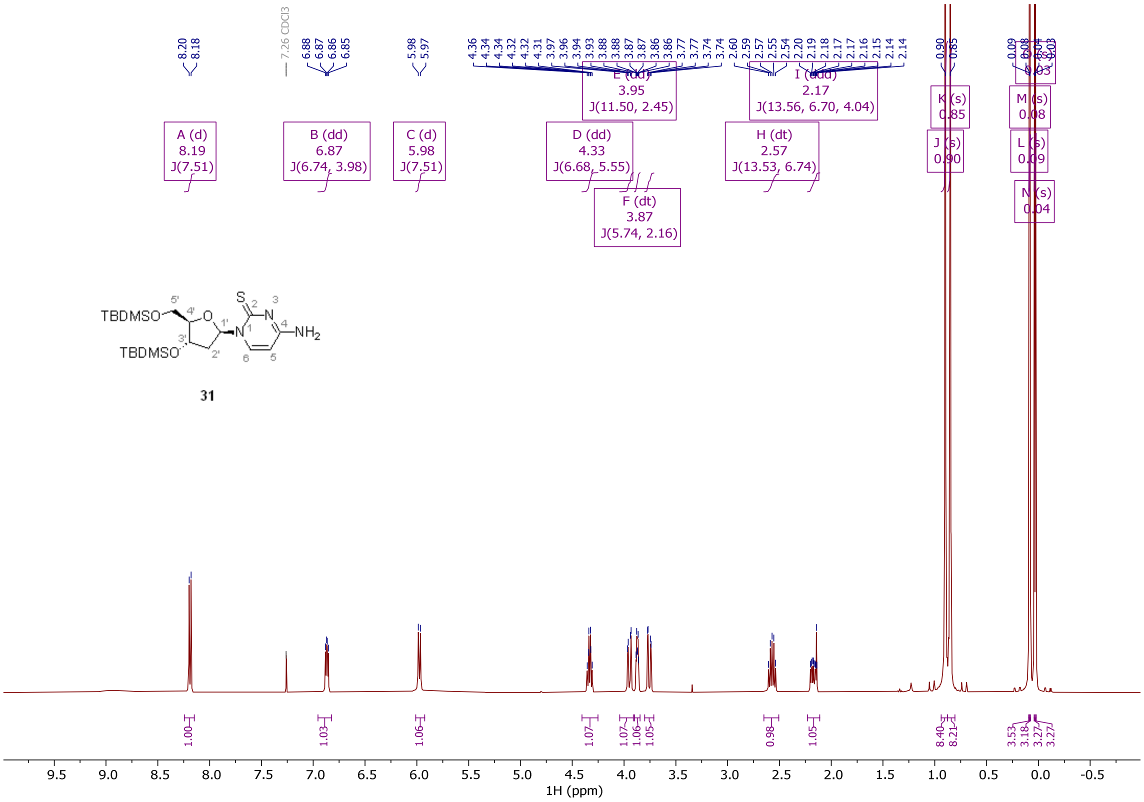

Figure S121 - ^{1}H NMR (400 MHz) spectrum of **31** in $CDCl_3$

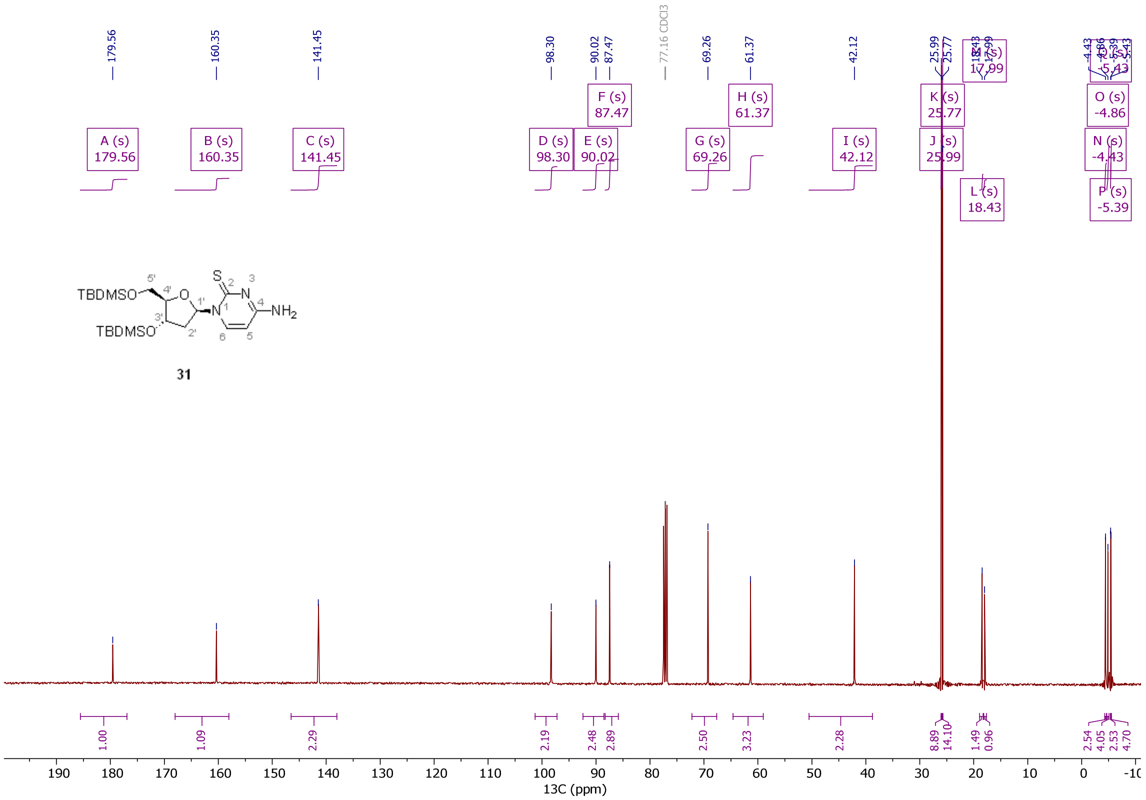

Figure S122 - ^{13}C NMR (101 MHz) spectrum of **31** in $CDCl_3$

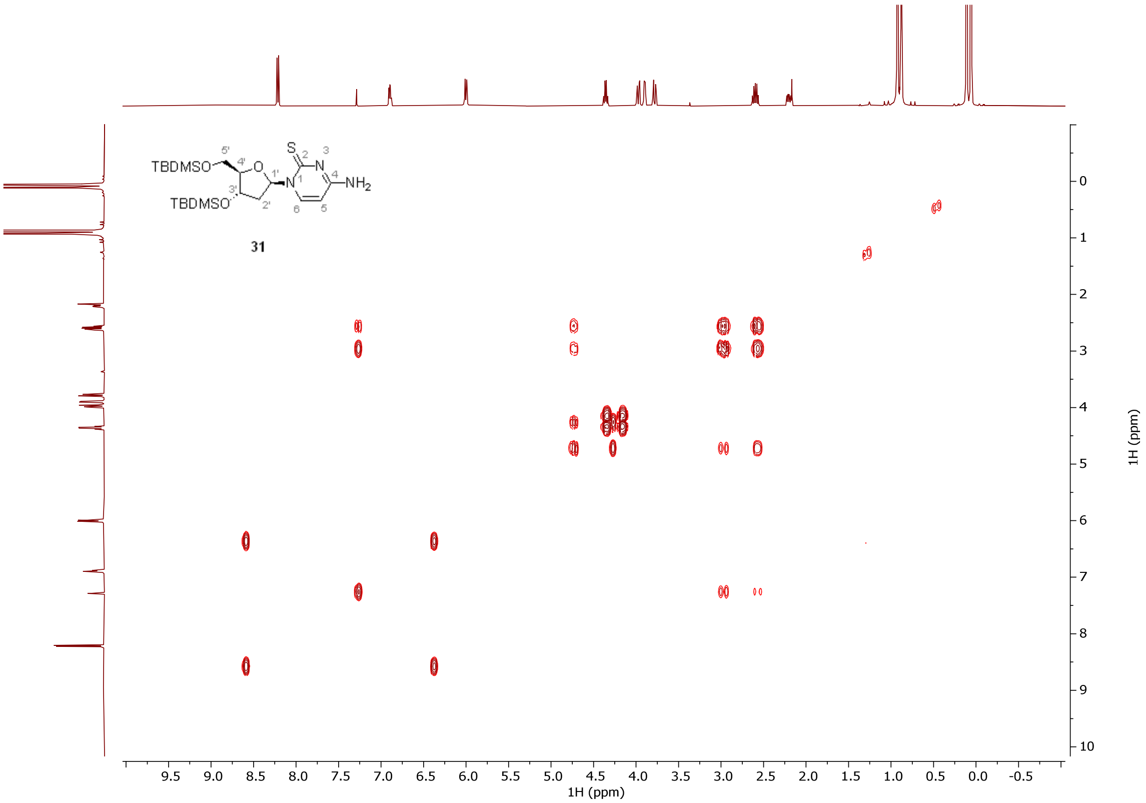

Figure S123 - ^{1}H-^{1}H COSY NMR spectrum of **31** in $CDCl_3$

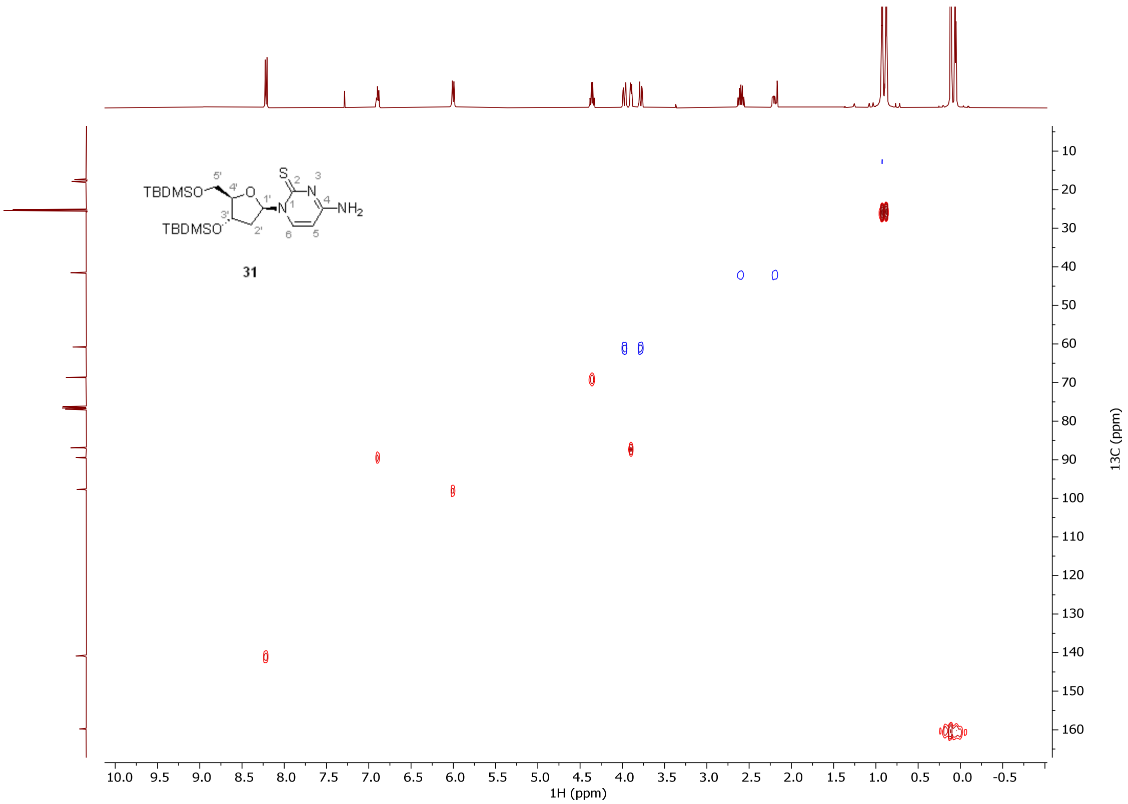

Figure S124 - ^{1}H-^{13}C HSQC NMR spectrum of **31** in $CDCl_3$

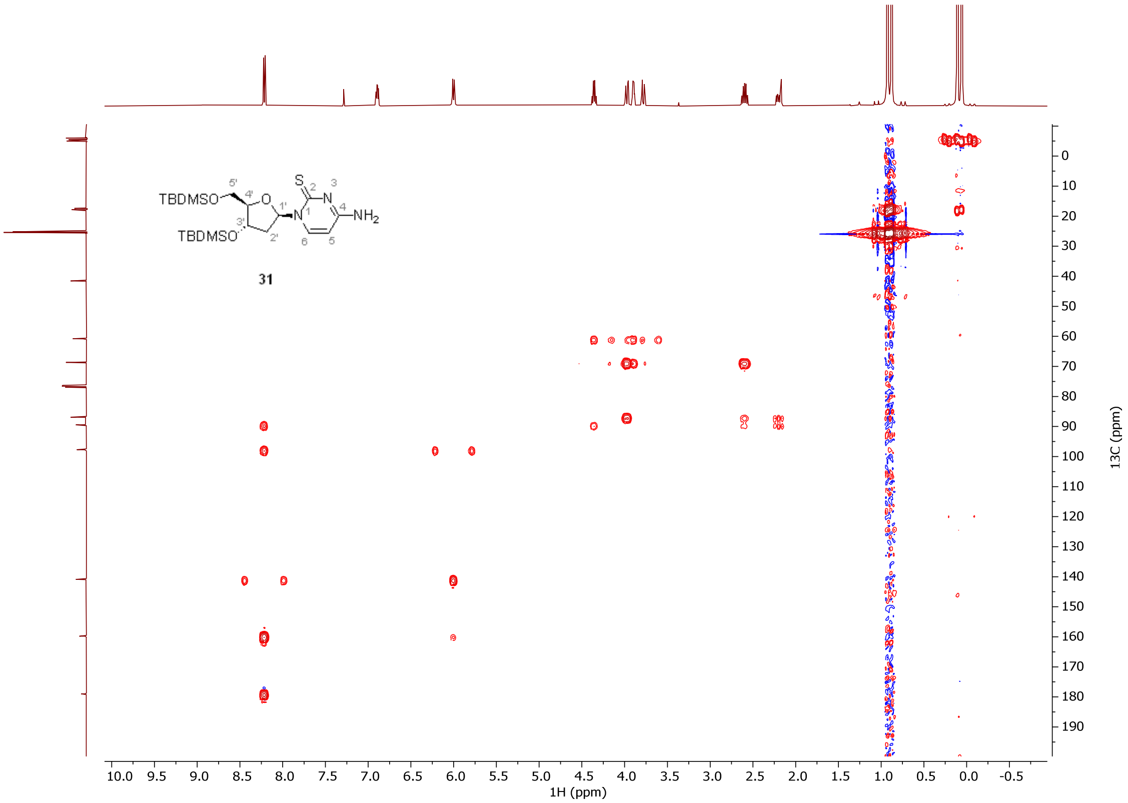

Figure S125 - ^{1}H-^{13}C HMBC NMR spectrum of **31** in $CDCl_3$

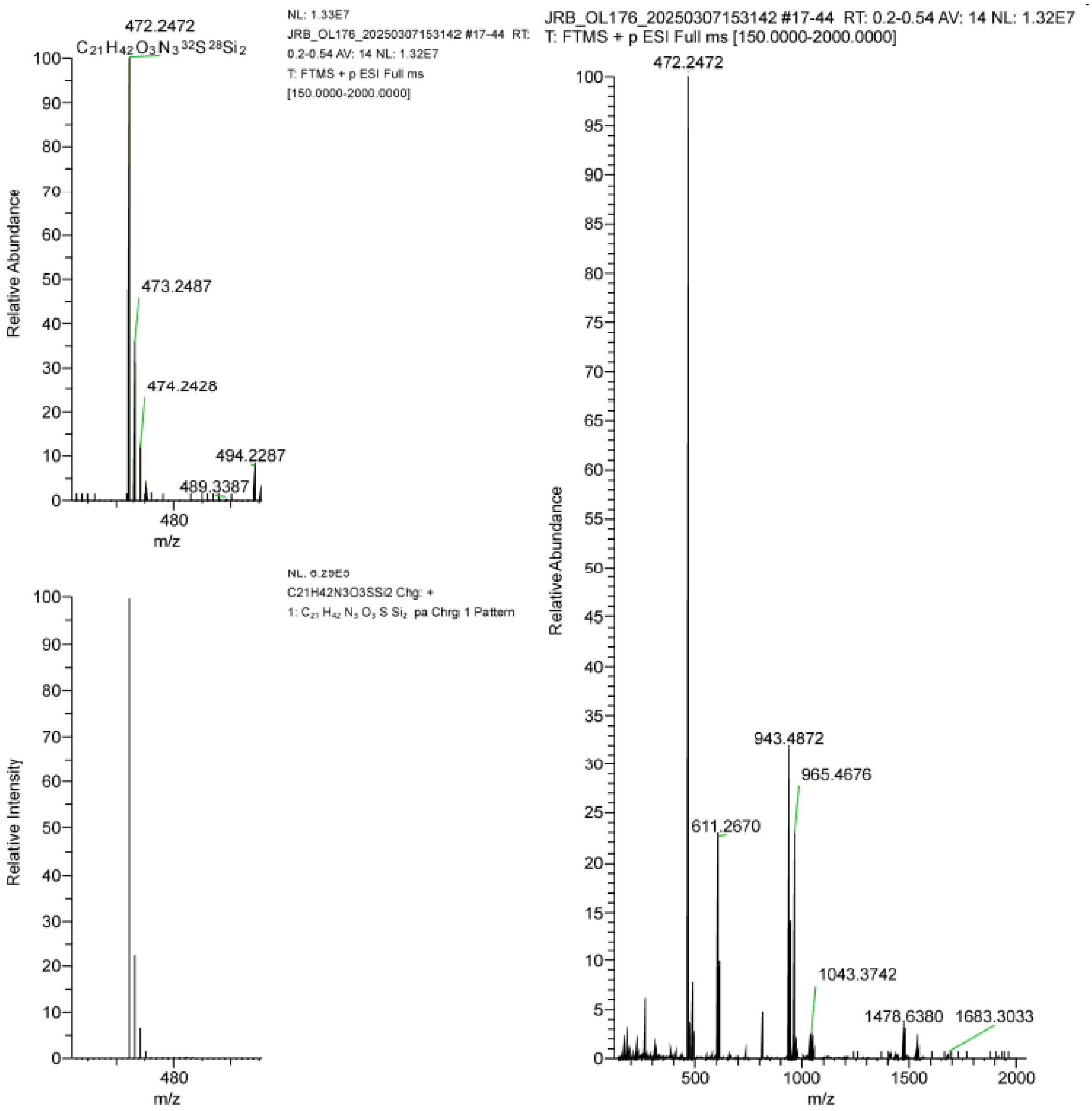

Peak Mass	Display...	S Fit	RDB	Delta [p...	Theo. m...	Rank	Combin...	# Match...	# Misse...	MS Cov....	Pattern...	MSMS...
472.2472	$C_{20}H_{42}O_4N_3{}^{28}Si_3$	21.1227280219929	4.50	-1.25	472.24776	1	86.26	5	0	89.88	88.93	(Collection)
472.2472	$C_{21}H_{42}O_3N_3{}^{32}S^{28}Si_2$	14.8679169294263	4.50	-1.73	472.24799	2	85.93	6	0	89.88	88.69	(Collection)
472.2472	C[illegible]H[illegible]O[illegible]$N_3{}^{32}S_2{}^{28}Si$	13.142385668570 8	4.50	-2.22	472.24822	3	70.9	6	0	74.1	77.23	(Collection)
472.2472	$C_{23}H_{44}O_4{}^{32}S^{28}Si_2$	4.9642481308458 3	4.00	-4.58	472.24934	4	63.27	5	1	66.51	66.68	(Collection)

Figure S126 - HRMS spectrum of **31**

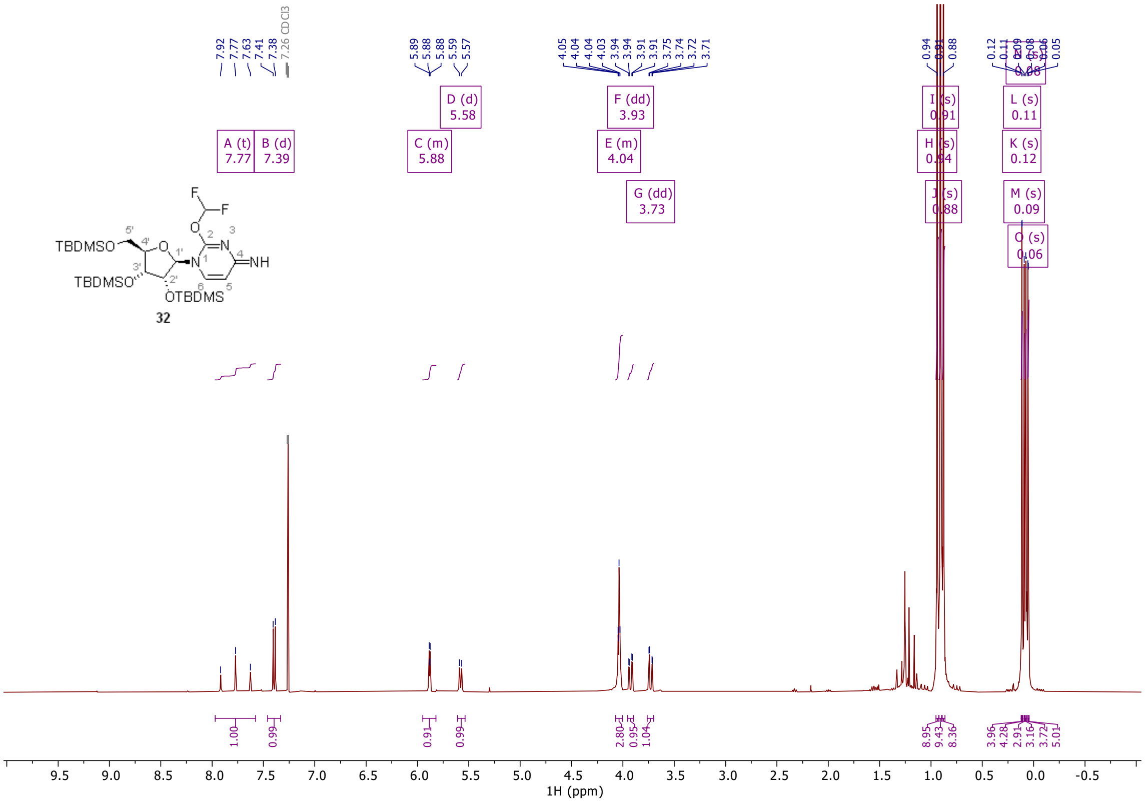

Figure S127 - ^{1}H NMR (400 MHz) spectrum of **32** in $CDCl_3$

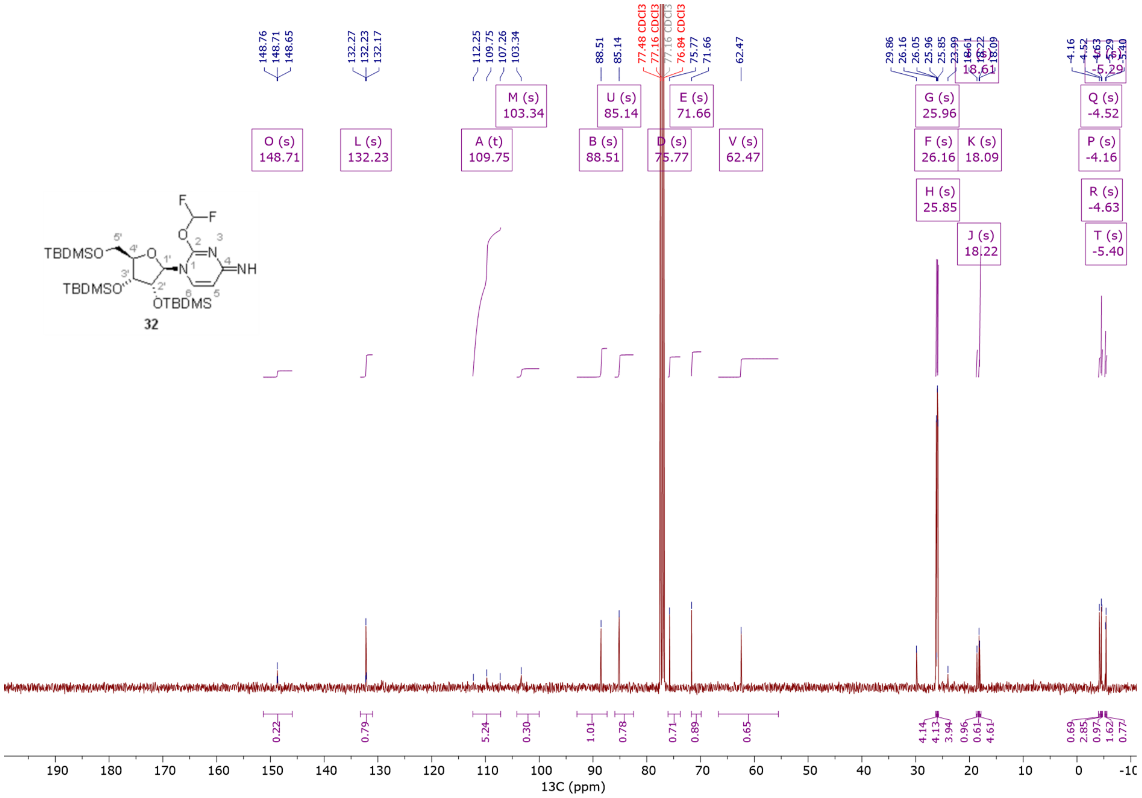

Figure S128 - ^{13}C NMR (101 MHz) spectrum of **32** in $CDCl_3$

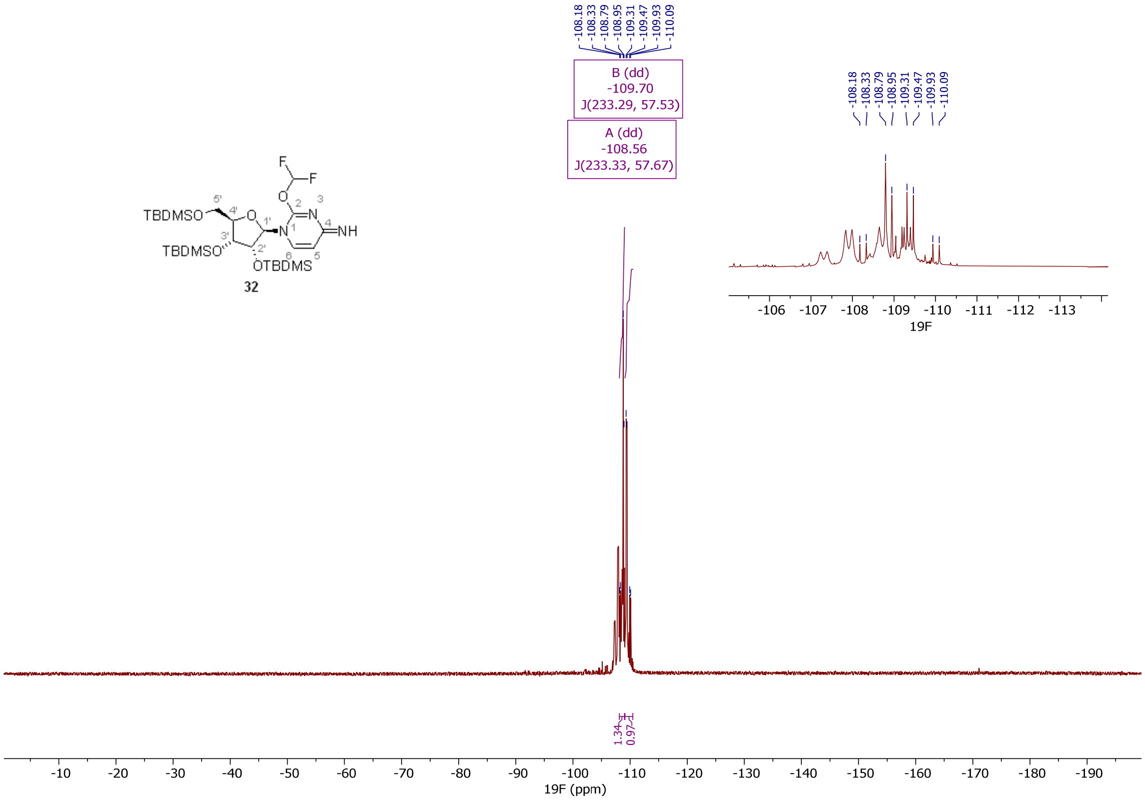

Figure S129 - ^{19}F NMR (376 MHz) spectrum of **32** in $CDCl_3$

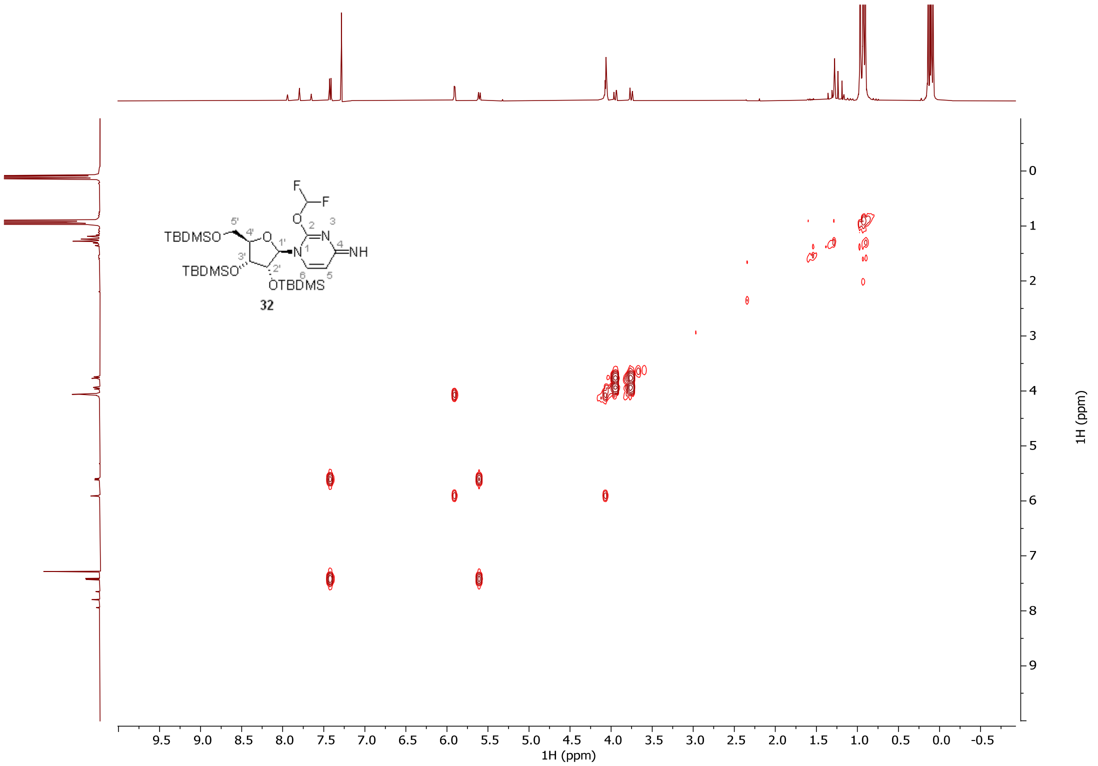

Figure S130 - ^{1}H-^{1}H COSY NMR spectrum of **32** in $CDCl_3$

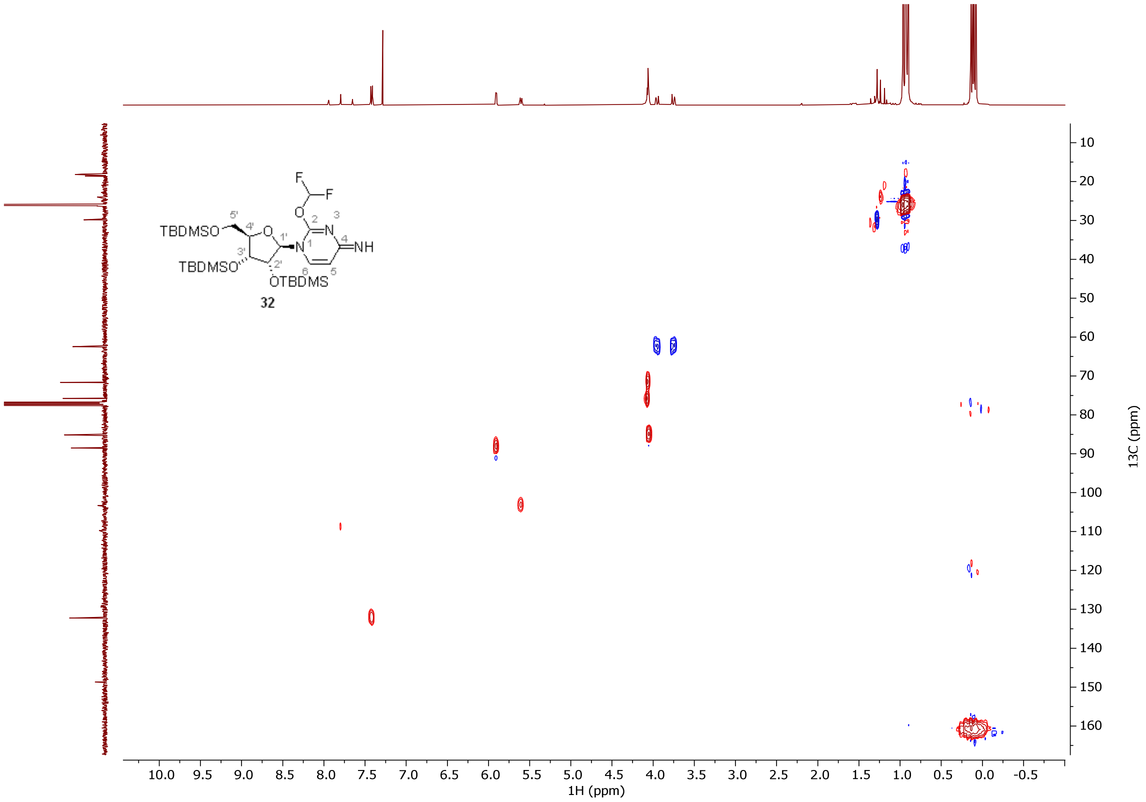

Figure S131 - ^{1}H-^{13}C HSQC NMR spectrum of **32** in $CDCl_3$

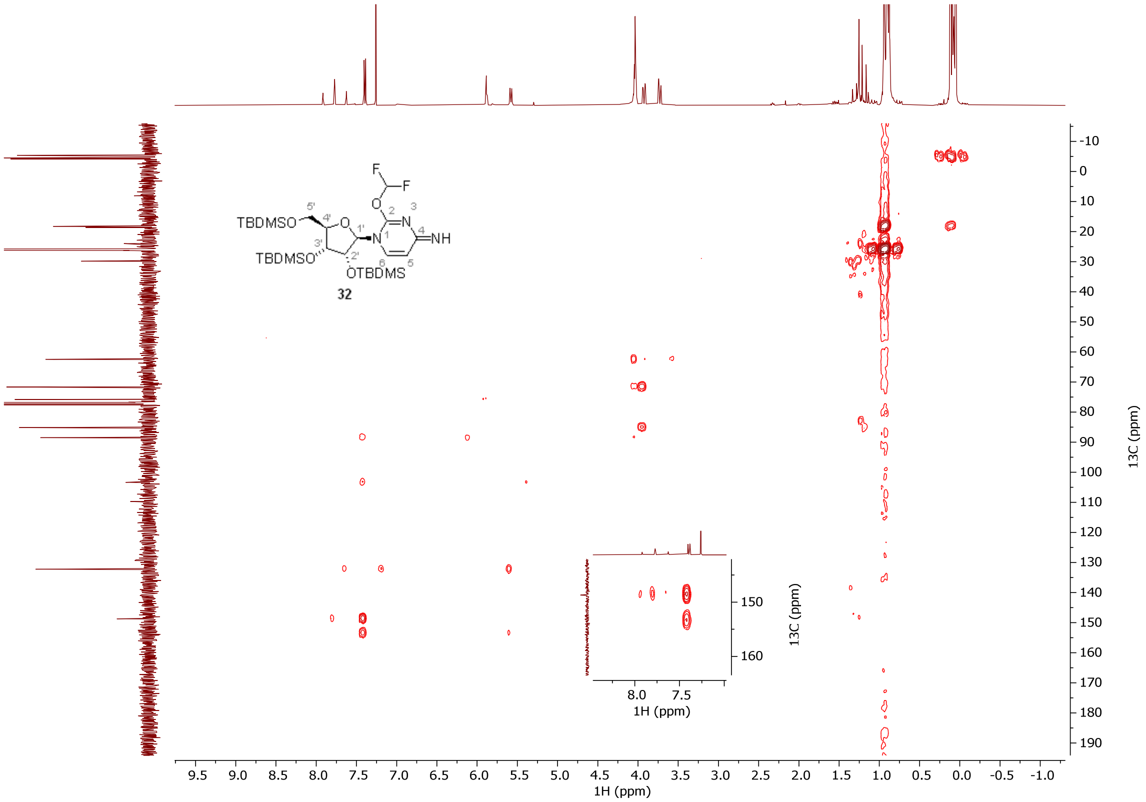

Figure S132 - ^{1}H-^{13}C HMBC NMR spectrum of **32** in $CDCl_3$

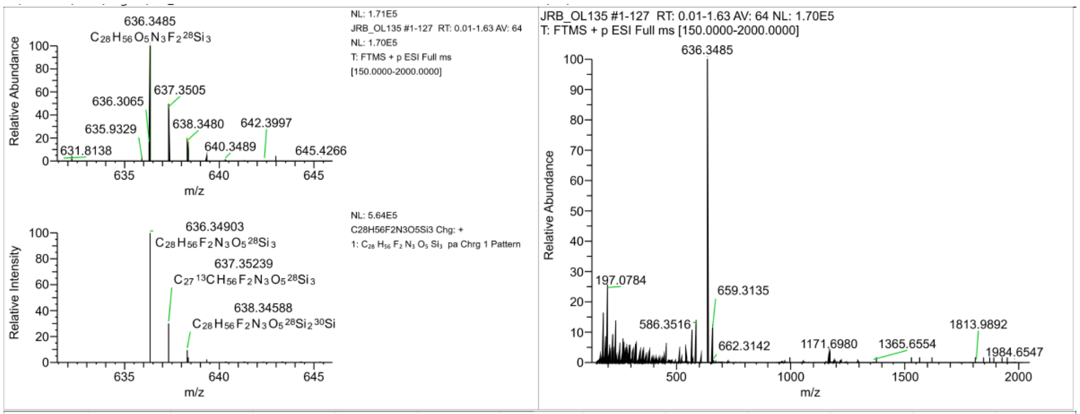

Peak Mass	Display For...	S Fit	RDB	Delta [ppm]	Theo. mass	Rank	Combined S...	# Matched I...	# Missed Iso.	MS Cov. [%]	Pattern Cov....	MSMS Matc...
636.3485	$C_{27}H_{50}N_{10}F_2{}^{28}Si_3$	65.9130766395726	10.00	-0.88	636.34903	1	98.18	6	0	99.97	100	(Collection)
636.3485	$C_{28}H_{56}O_5N_3F_2{}^{28}Si_3$	45.6915069339511	4.50	-0.89	636.34903	2	97.03	6	0	99.89	99.88	(Collection)
636.3485	$C_{28}H_{56}O_{10}N_2{}^{28}Si_2$	39.5390364397484	4.00	2.62	636.34680	3	96.71	6	0	99.89	99.94	(Collection)
636.3485	$C_{29}H_{53}O_3N_6F{}^{28}Si_3$	39.1672433604246	9.00	3.02	636.34655	4	96.69	6	0	99.89	99.88	(Collection)
636.3485	$C_{29}H_{52}ON_7F_2{}^{28}Si_3$	33.2158482585015	9.50	-2.99	636.35037	5	96.38	6	0	99.89	99.89	(Collection)
636.3485	$C_{29}H_{52}O_6N_6{}^{28}Si_2$	40.8590001917092	9.00	0.52	636.34814	6	96.27	5	0	99.35	99.57	(Collection)
636.3485	$C_{27}H_{50}O_5N_9{}^{28}Si_2$	29.1137094749326	9.50	2.63	636.34680	7	93.09	5	0	96.65	97.45	(Collection)
636.3485	$C_{28}H_{46}O_3N_{10}F_2{}^{28}Si$	19.4132583028689	11.00	-0.24	636.34862	8	83.47	3	2	87.03	92.39	(Collection)
636.3485	$C_{29}H_{52}O_8N_3F_2{}^{28}Si$	18.7265519097872	5.50	-0.25	636.34862	9	83.43	3	2	87.03	91.79	(Collection)
636.3485	$C_{27}H_{50}O_7N_6F_2{}^{28}Si$	16.572149782327	6.00	1.86	636.34728	10	83.32	3	2	87.03	92.22	(Collection)

Figure S133 - HRMS spectrum of **32**

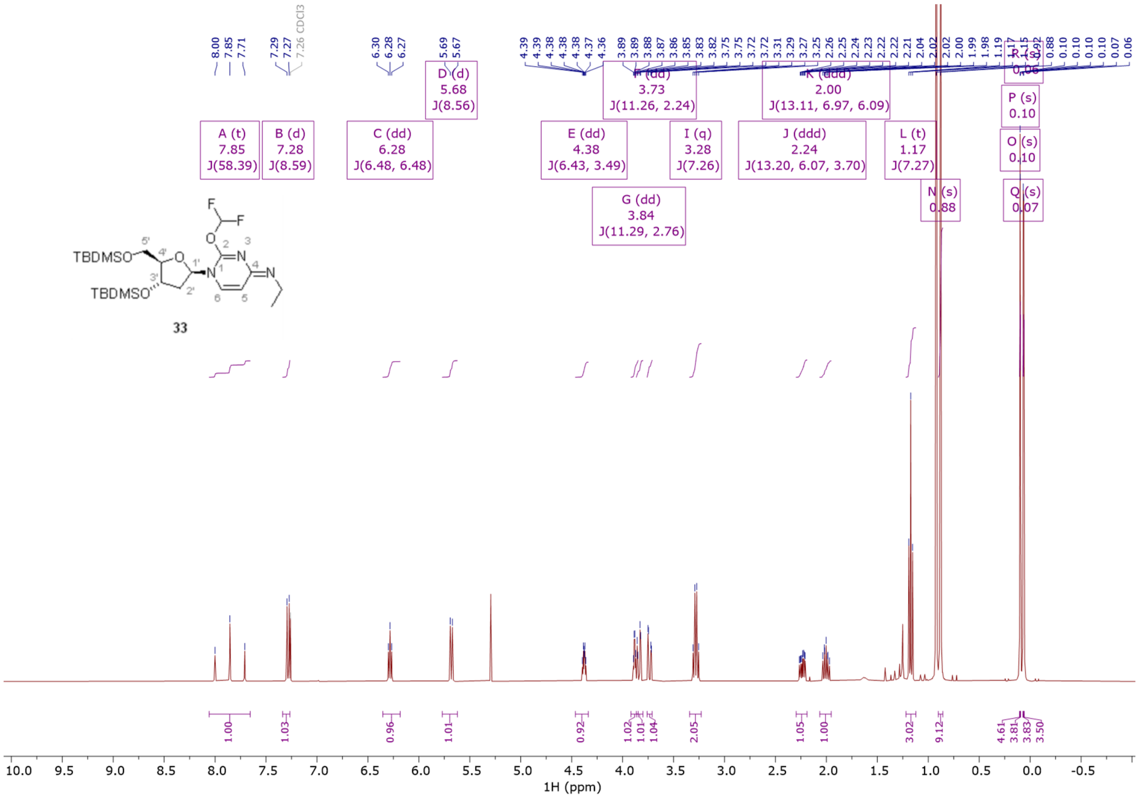

Figure S134 - ^1H NMR (400 MHz) spectrum of **33** in $CDCl_3$

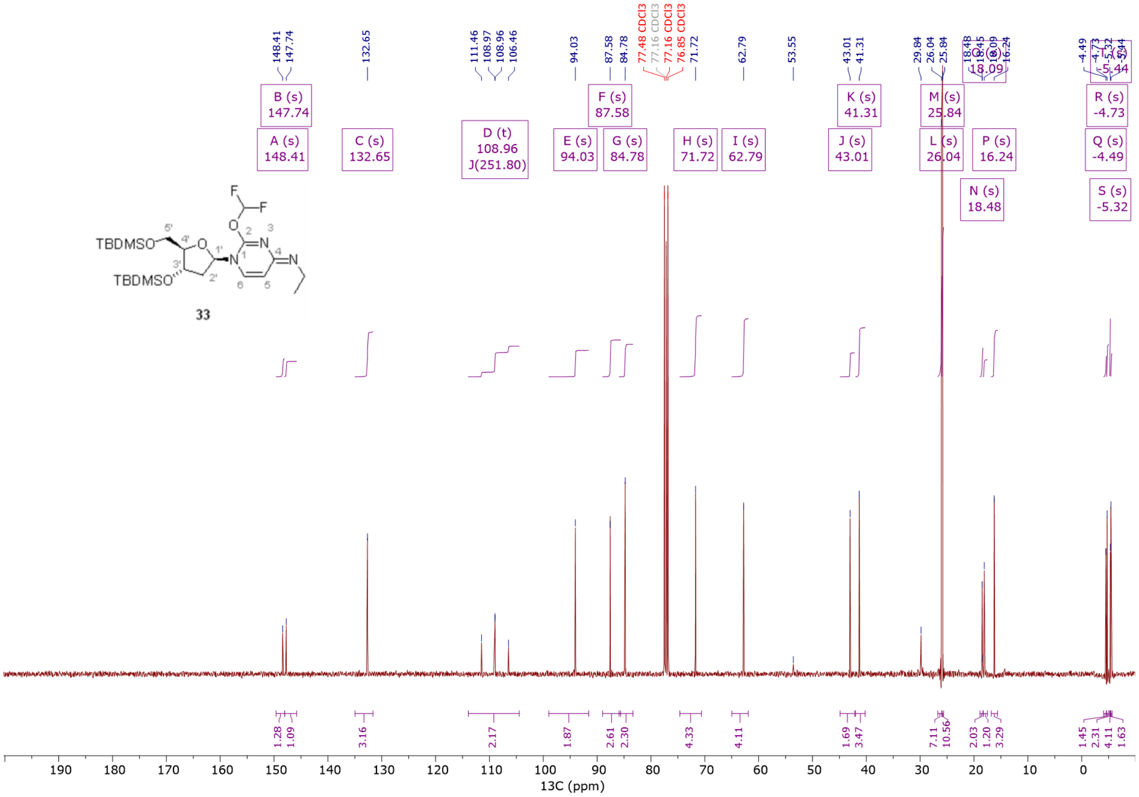

Figure S135 - ^{13}C NMR (101 MHz) spectrum of **33** in $CDCl_3$

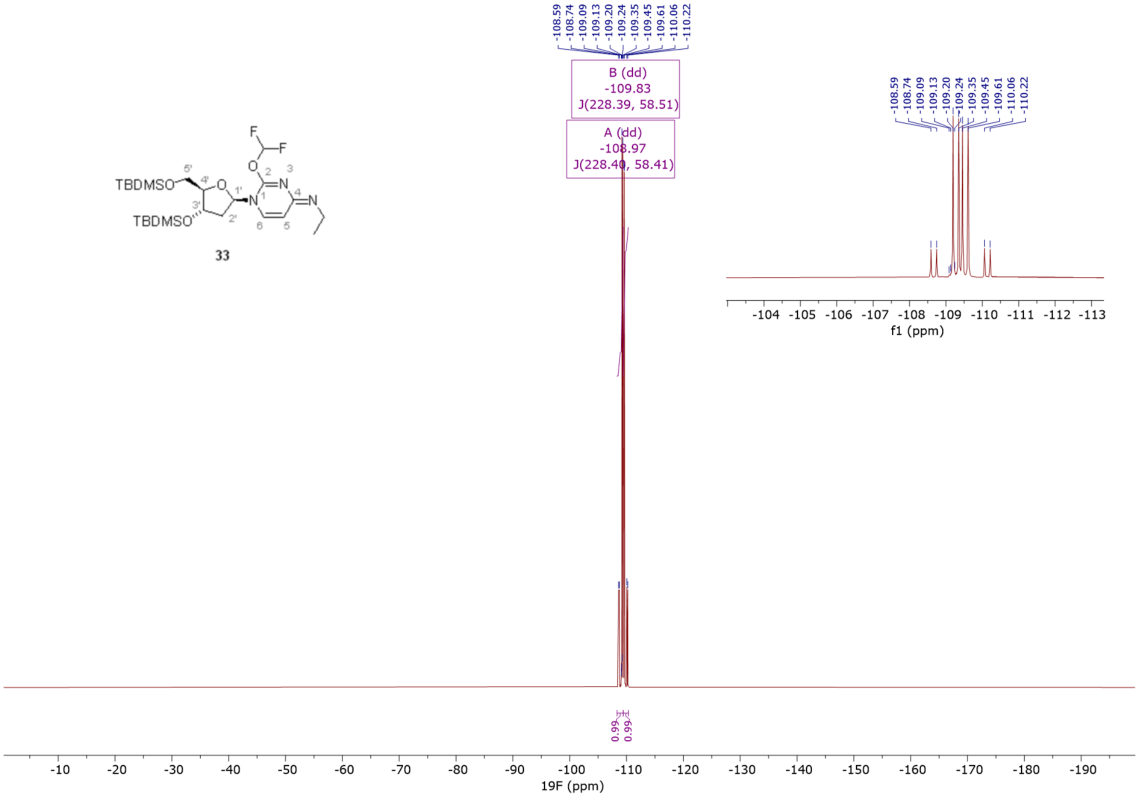

Figure S136 - ^{19}F NMR (376 MHz) spectrum of **33** in $CDCl_3$

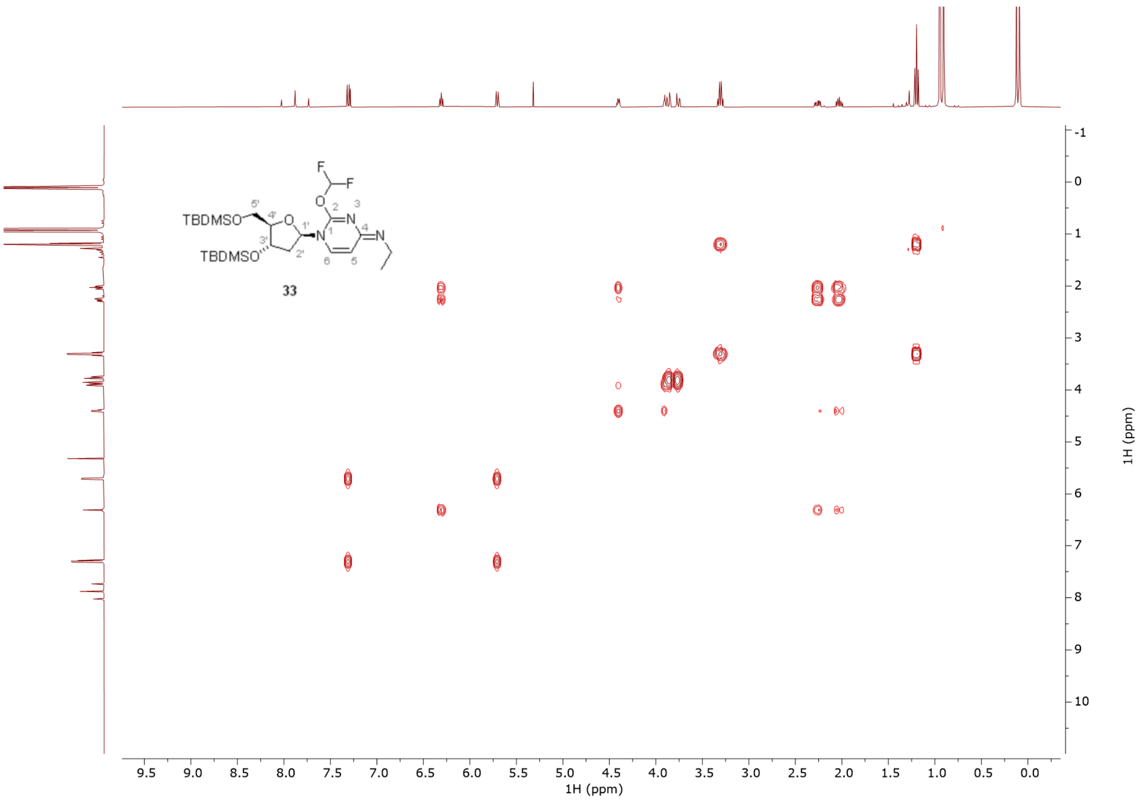

Figure S137 - ^{1}H-^{1}H COSY NMR spectrum of **33** in $CDCl_3$

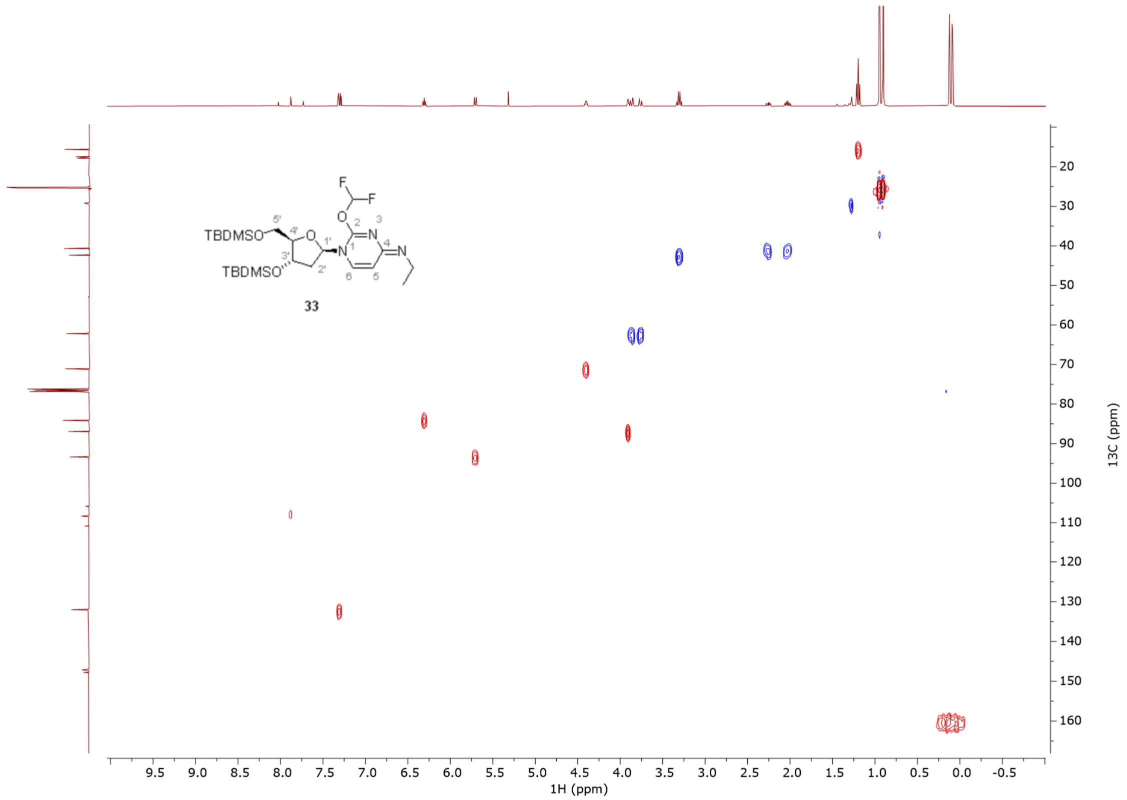

Figure S138 - ^{1}H-^{13}C HSQC NMR spectrum of **33** in $CDCl_3$

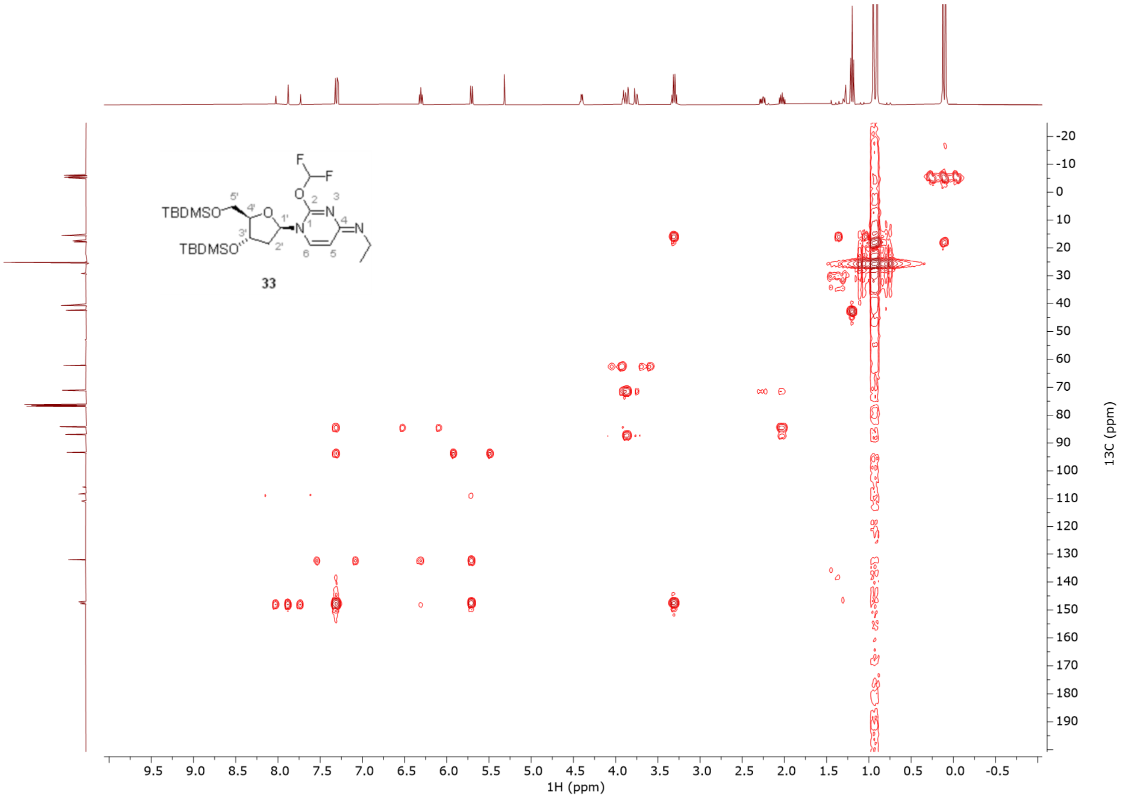

Figure S139 - ^{1}H-^{13}C HMBC NMR spectrum of **33** in $CDCl_3$

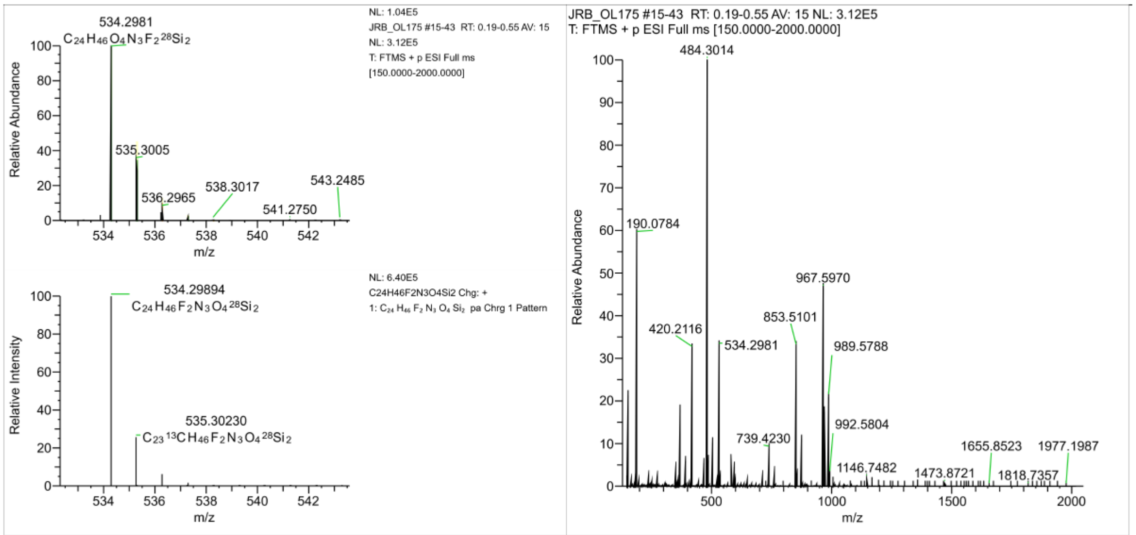

Peak Mass	Display For...	S Fit	RDB	Delta [ppm]	Theo. mass	Rank	Combined S...	# Matched I...	# Missed Iso.	MS Cov. [%]	Pattern Cov....	MSMS Matc...
534.2981	$C_{22}H_{44}O_3N_6F_2{}^{28}Si_2$	45.92566581 45159	5.00	0.87	534.29760	1	95.55	5	0	98.31	99.76	(Collection)
534.2981	$C_{21}H_{48}O_7N_2F_2{}^{28}Si_2$	37.47639356 49995	0.00	3.37	534.29626	2	95.11	5	0	98.31	99.73	(Collection)
534.2981	$C_{20}H_{42}O_2N_9F_2{}^{28}Si_2$	31.50877387 55757	5.50	3.38	534.29626	3	94.79	5	0	98.31	99.78	(Collection)
534.2981	$C_{24}H_{46}O_4N_3F_2{}^{28}Si_2$	32.20372764 91692	4.50	-1.64	534.29894	4	90.07	5	0	93.29	92.88	(Collection)
534.2981	$C_{25}H_{42}N_7F_2{}^{28}Si_2$	21.13615907 23517	9.50	-4.15	534.30028	5	89.49	5	0	93.29	92.87	(Collection)
534.2981	$C_{26}H_{48}O_5F_2{}^{28}Si_2$	13.44440355 17983	4.00	-4.16	534.30029	6	87.59	5	0	91.71	90.99	(Collection)

Figure S140 - HRMS spectrum of **33**

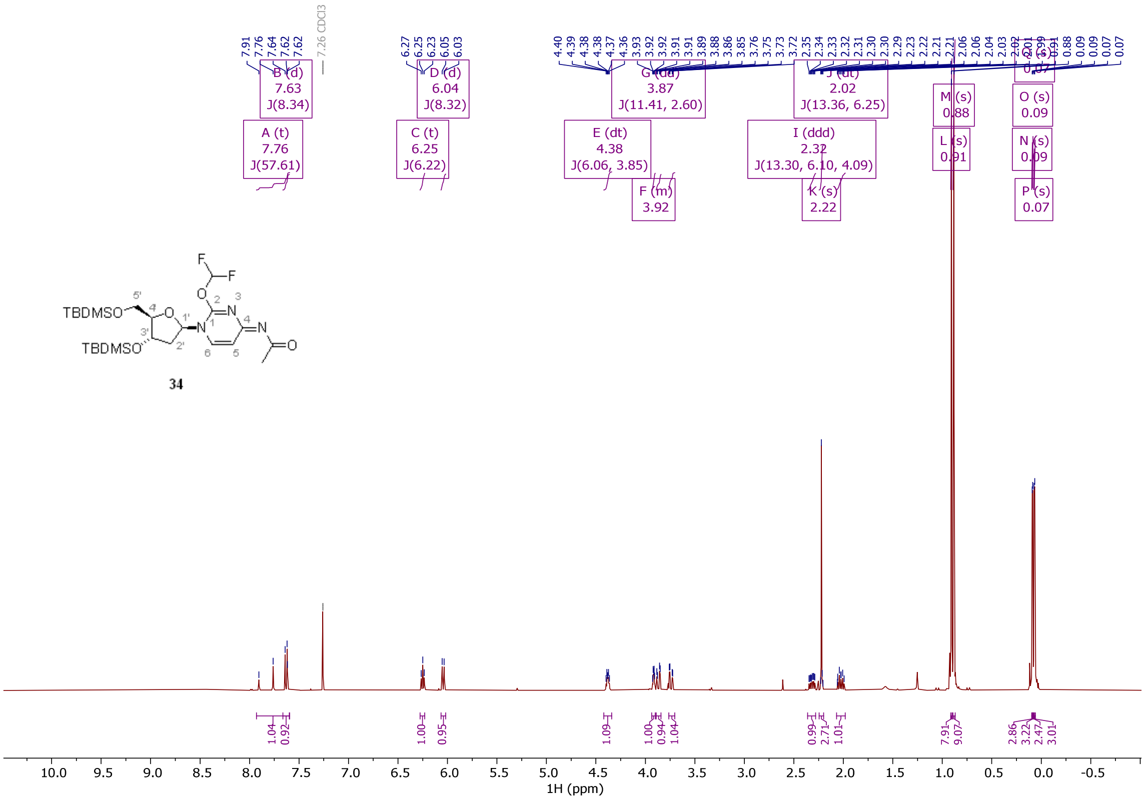

Figure S141 - ^{1}H NMR (400 MHz) spectrum of **34** in $CDCl_3$

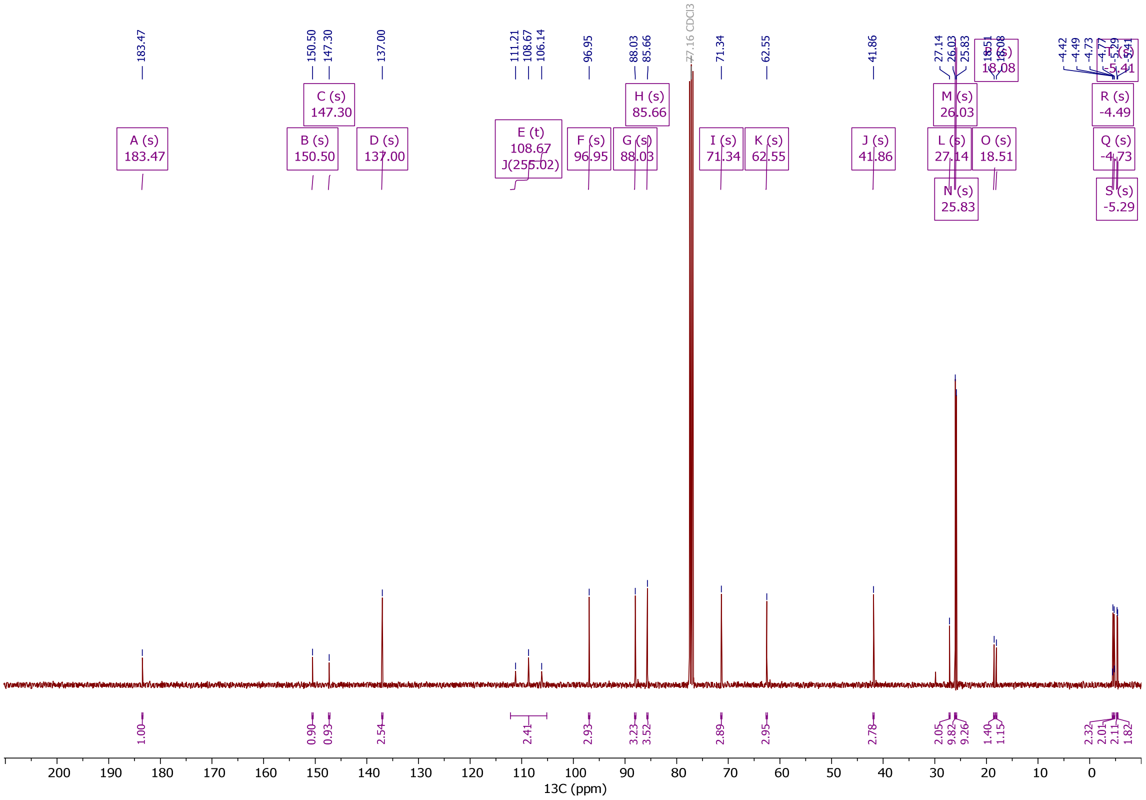

Figure S142 - ^{13}C NMR (101 MHz) spectrum of **34** in $CDCl_3$

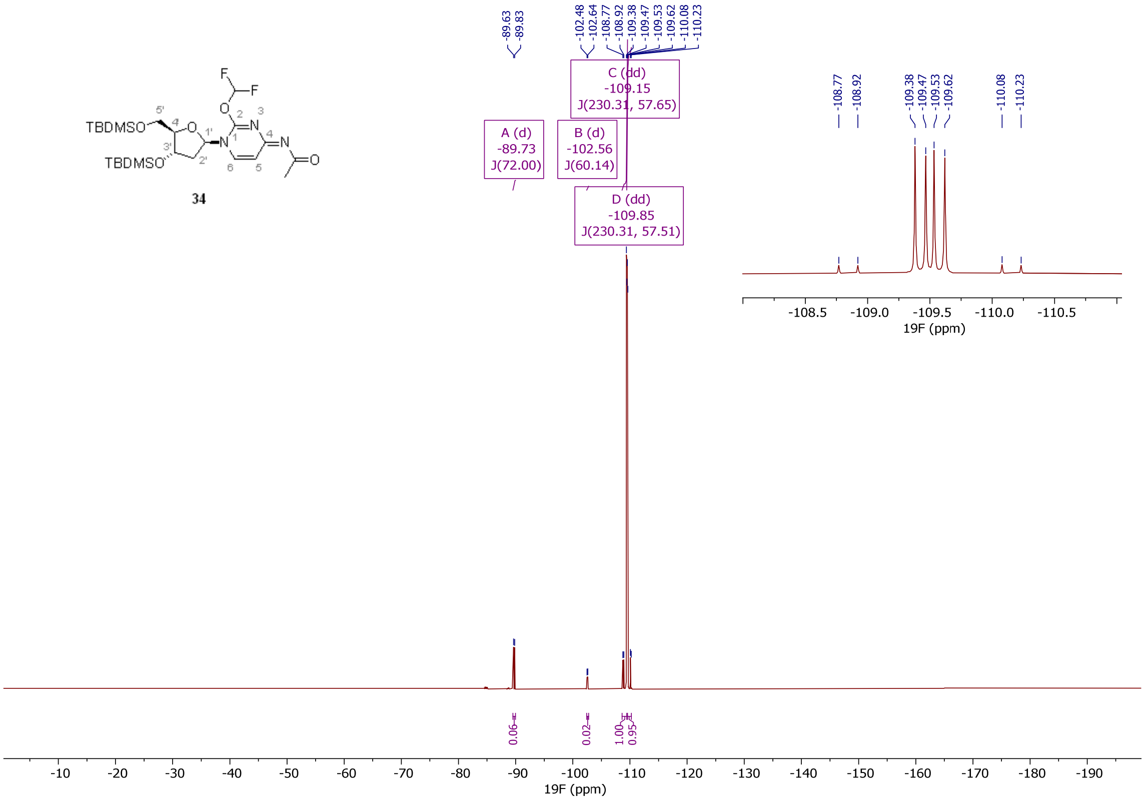

Figure S143 - ^{19}F NMR (376 MHz) spectrum of **34** in $CDCl_3$

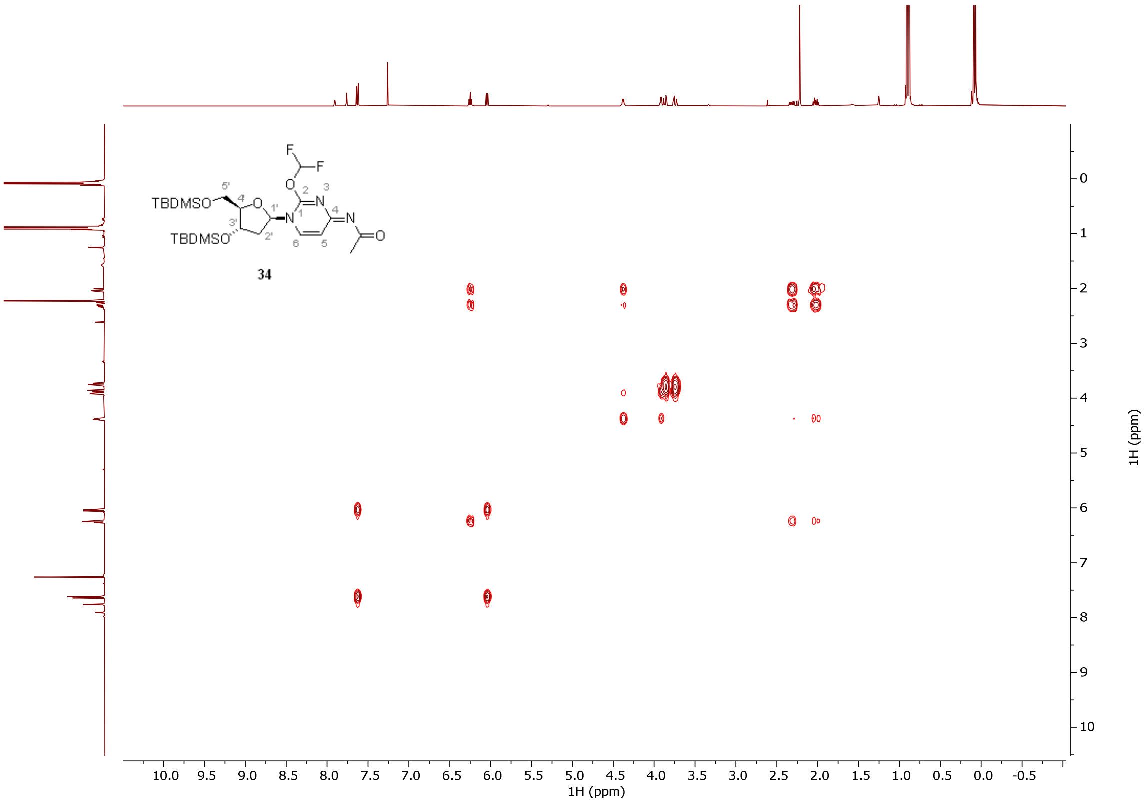

Figure S144 - ^{1}H-^{1}H COSY NMR spectrum of **34** in $CDCl_3$

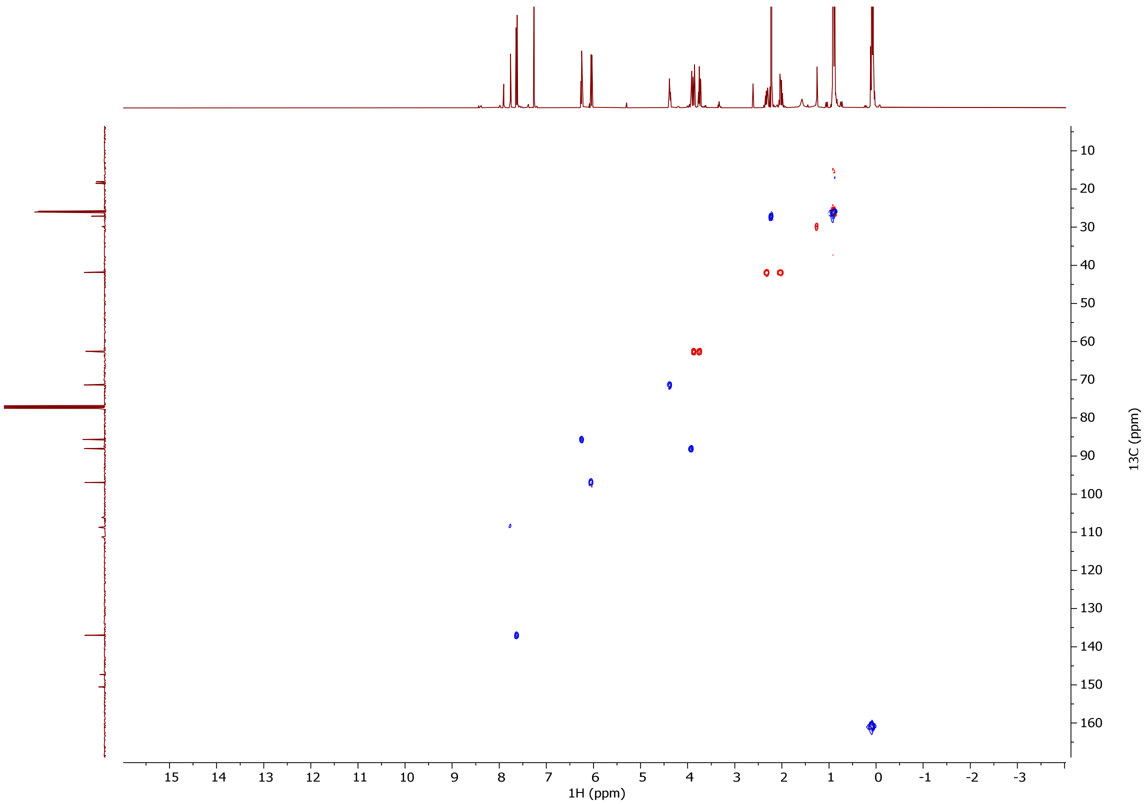

Figure S145 - ^{1}H-^{13}C HSQC NMR spectrum of **34** in $CDCl_3$

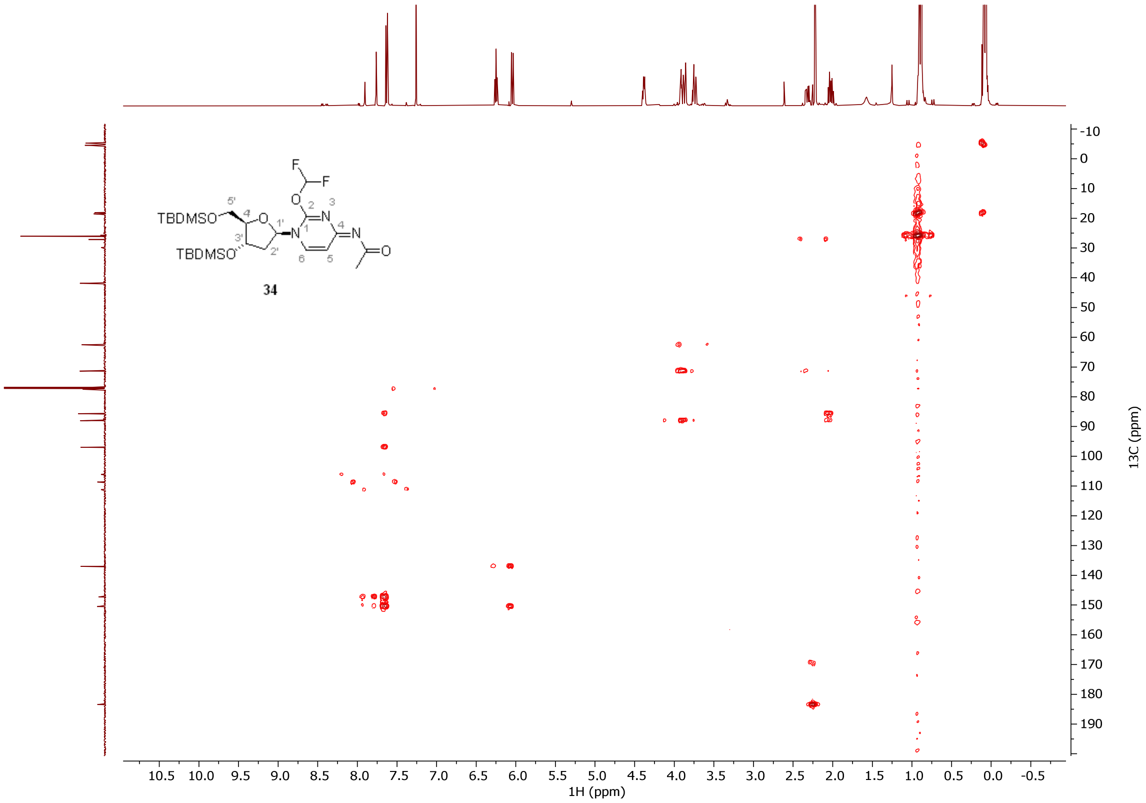

Figure S146 - ^{1}H-^{13}C HMBC NMR spectrum of **34** in $CDCl_3$

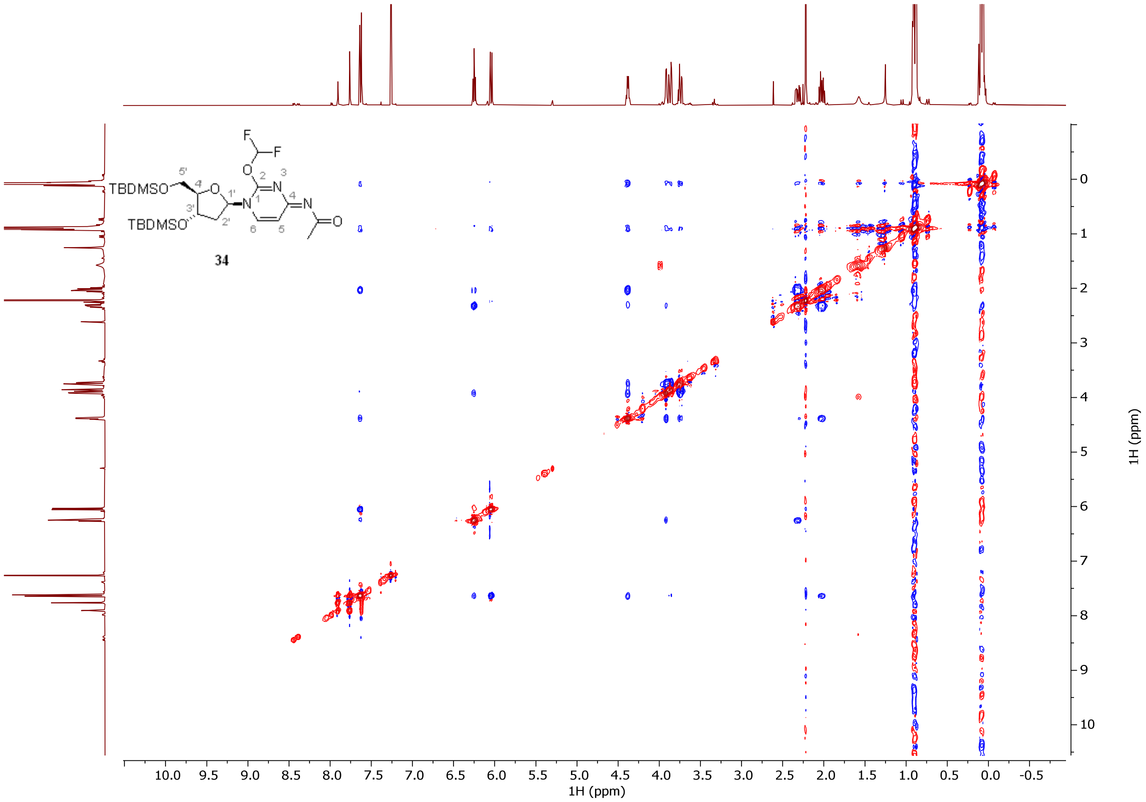

Figure S147 - ^{1}H-^{1}H NOESY NMR spectrum of **34** in $CDCl_3$

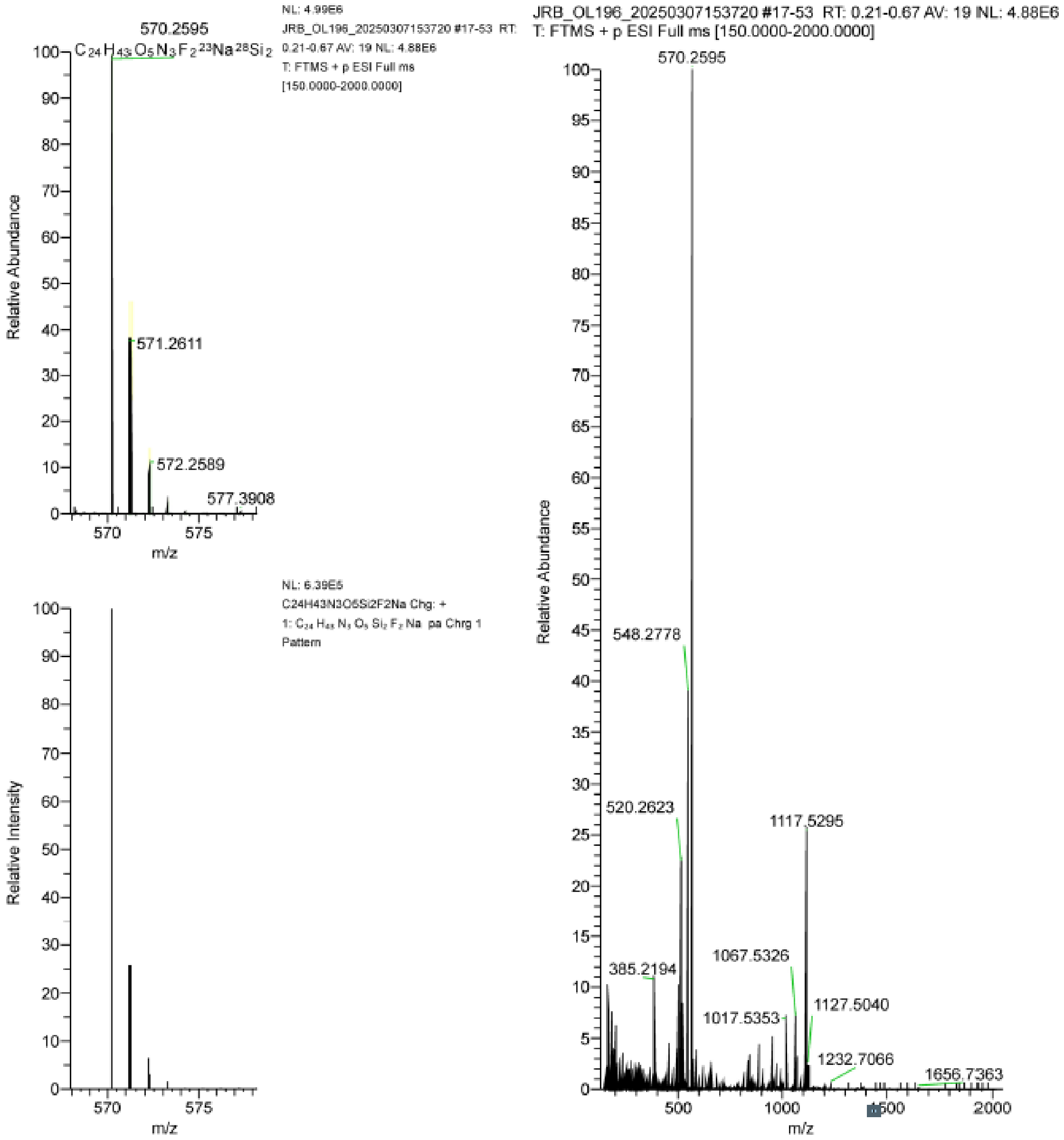

Peak Mass	Display Formula	S Fit	RDB	Delta [ppm]	Theo. mass	Rank	Combined Score	# Matched Iso.	# Missed Iso.	MS Co
570.2595	$C_{24}H_{43}O_5N_3F_2{}^{23}Na^{28}Si_2$	54.2769272224261	5.50	-1.13	570.26015	1	97.39	5	0	99.79

Figure S148 - HRMS spectrum of **34**

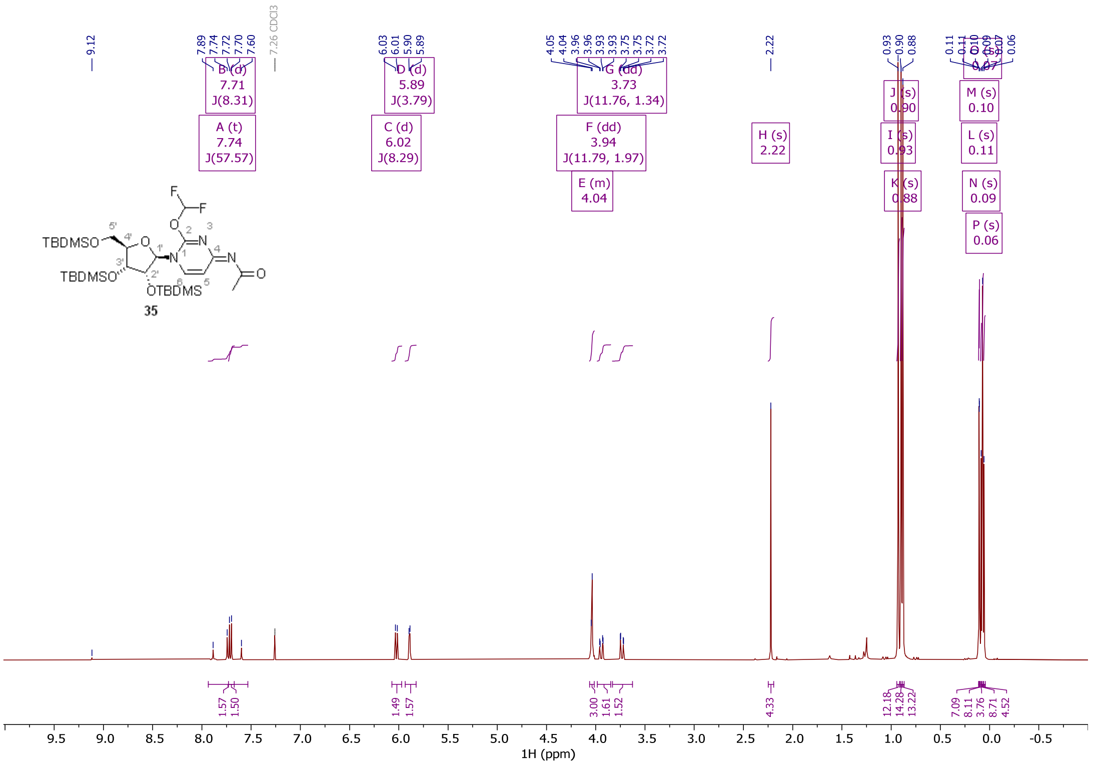

Figure S149 - ^{1}H NMR (400 MHz) spectrum of **35** in $CDCl_3$

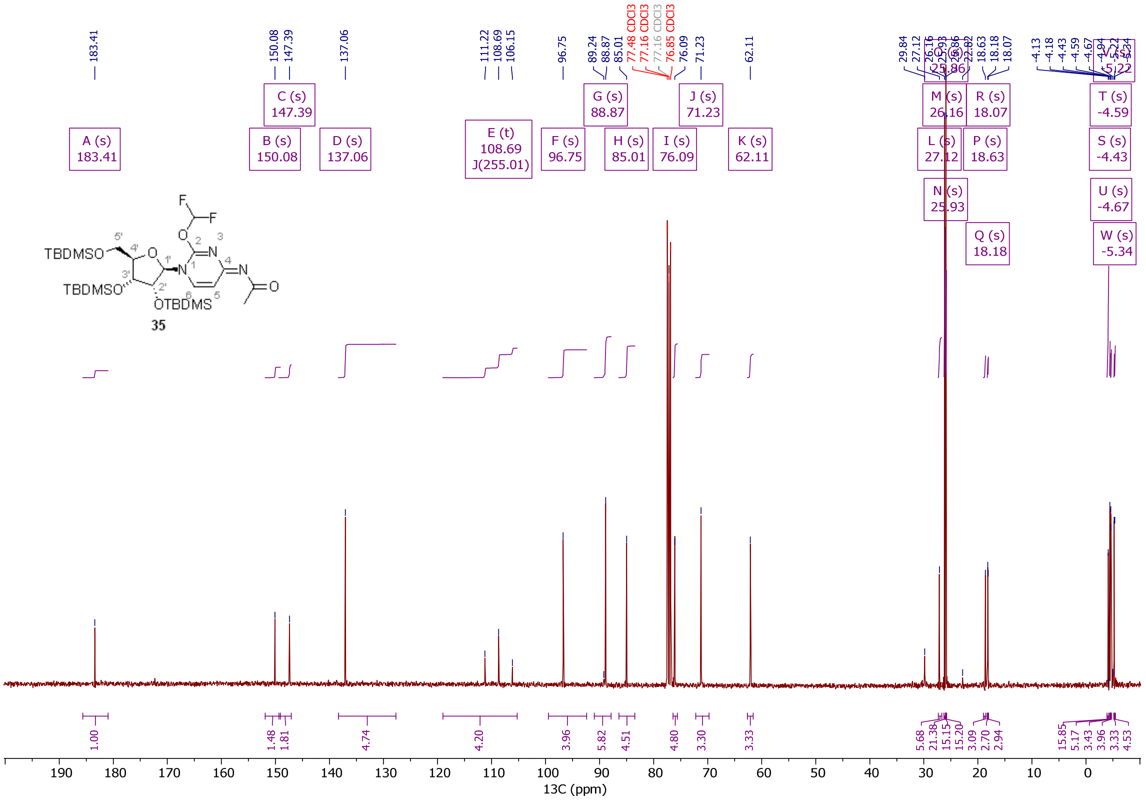

Figure S150 - ^{13}C NMR (101 MHz) spectrum of **35** in $CDCl_3$

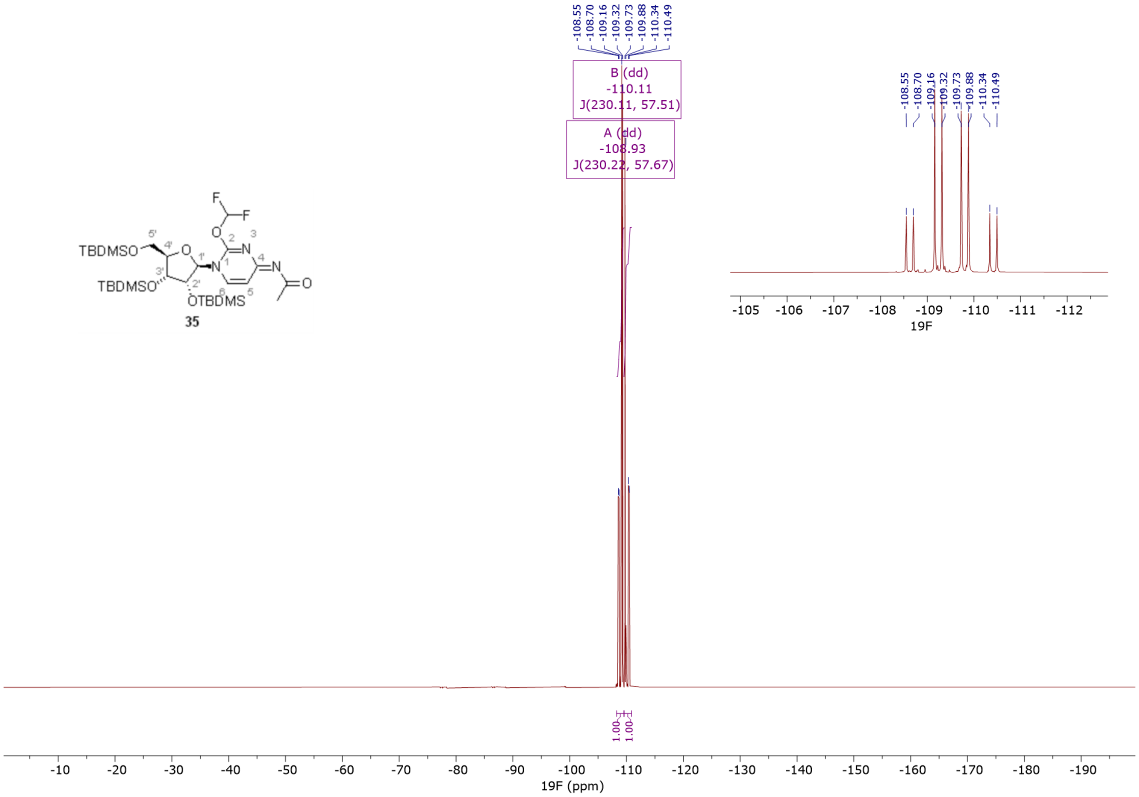

Figure S151 - ^{19}F NMR (376 MHz) spectrum of **35** in $CDCl_3$

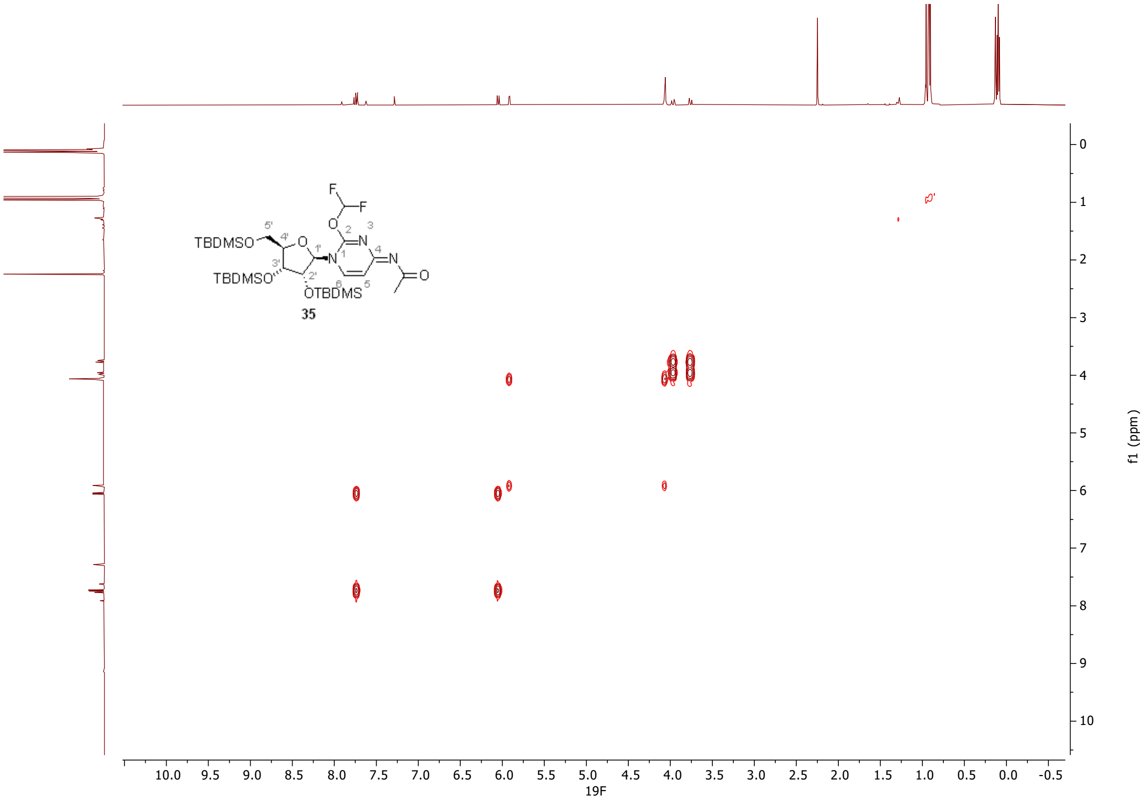

Figure S152 - ^{1}H-^{1}H COSY NMR spectrum of **35** in $CDCl_3$

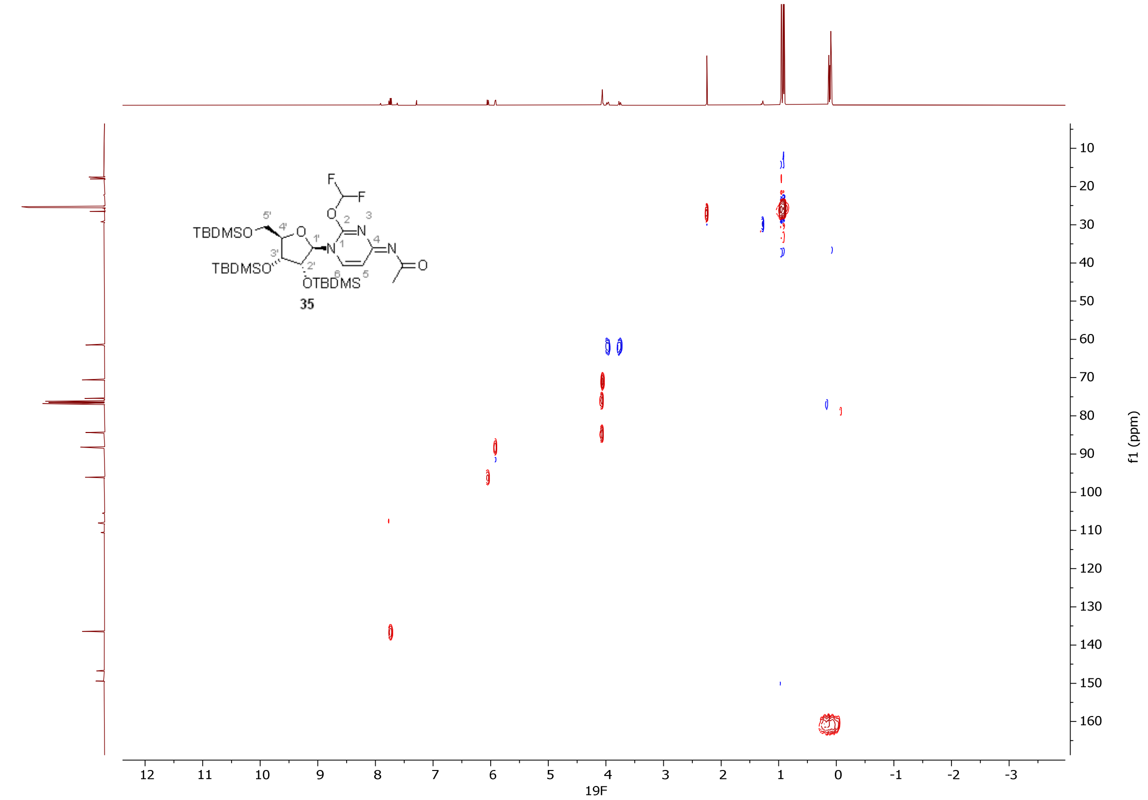

Figure S153 - ^{1}H-^{13}C HSQC NMR spectrum of **35** in $CDCl_3$

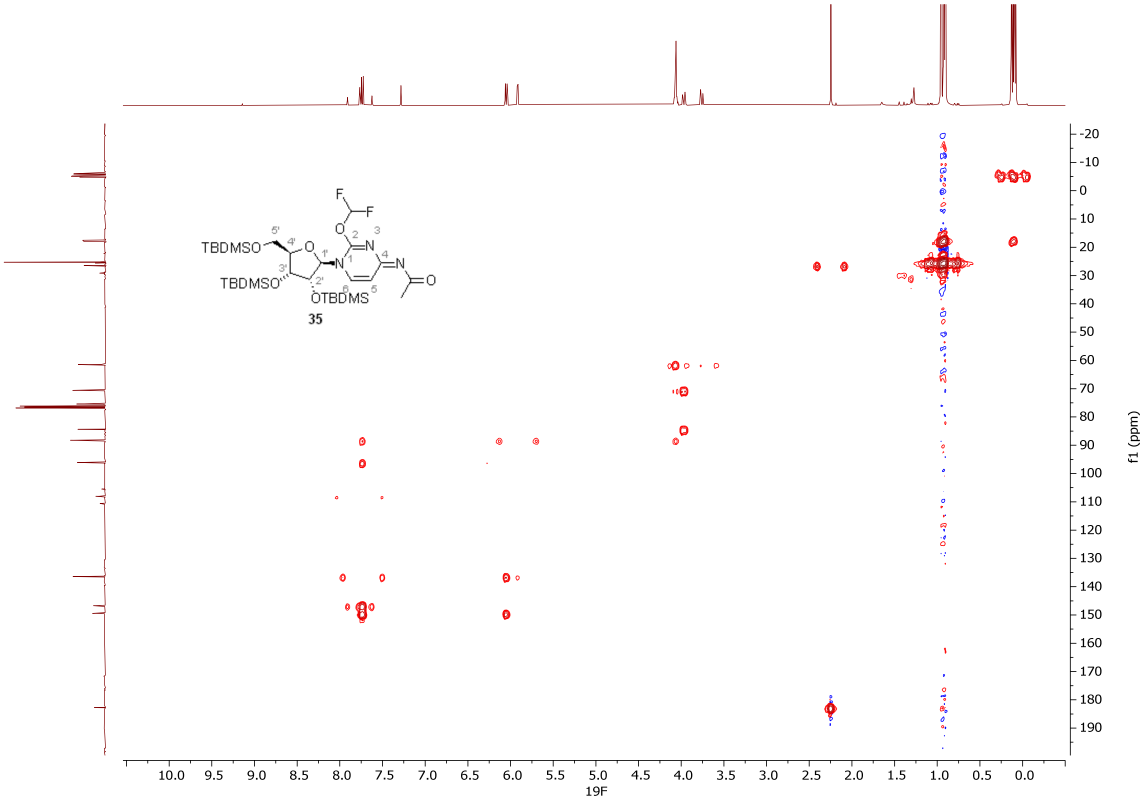

Figure S154 - ^{1}H-^{13}C HMBC NMR spectrum of **35** in $CDCl_3$

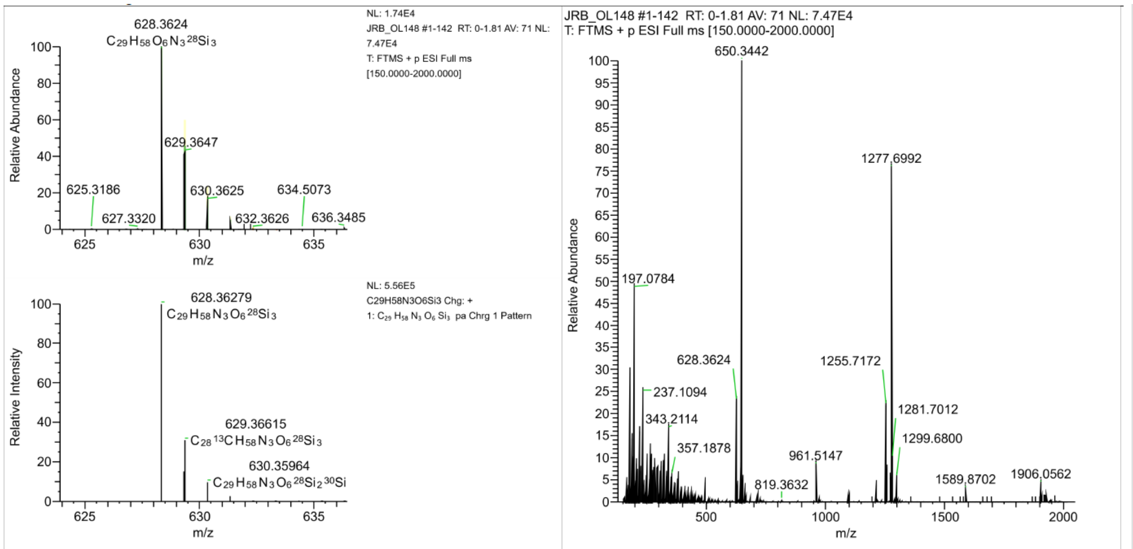

Peak Mass	Display For...	S Fit	RDB	Delta [ppm]	Theo. mass	Rank	Combined S...	# Matched I...	# Missed Iso.	MS Cov. [%]	Pattern Cov....	MSMS Matc...
628.3624	$C_{28}H_{52}ON_{10}{}^{28}Si_3$	48.4156377740183	11.00	-0.69	628.36279	1	96.88	5	0	99.57	99.34	(Collection)
628.3624	$C_{29}H_{58}O_6N_3{}^{28}Si_3$	36.2660276574621	5.50	-0.70	628.36279	2	96.24	5	1	99.57	99.17	(Collection)

Figure S155 - HRMS spectrum of **35**

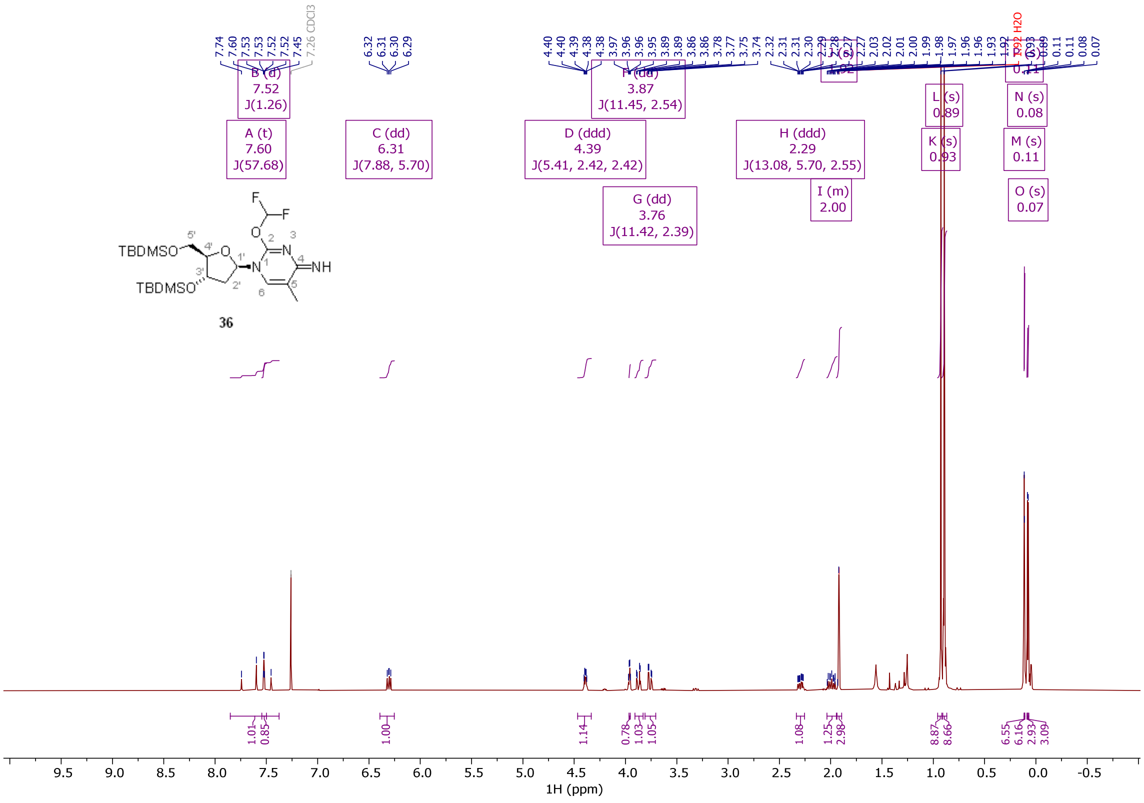

Figure S156 - ^{1}H NMR (400 MHz) spectrum of **36** in $CDCl_3$

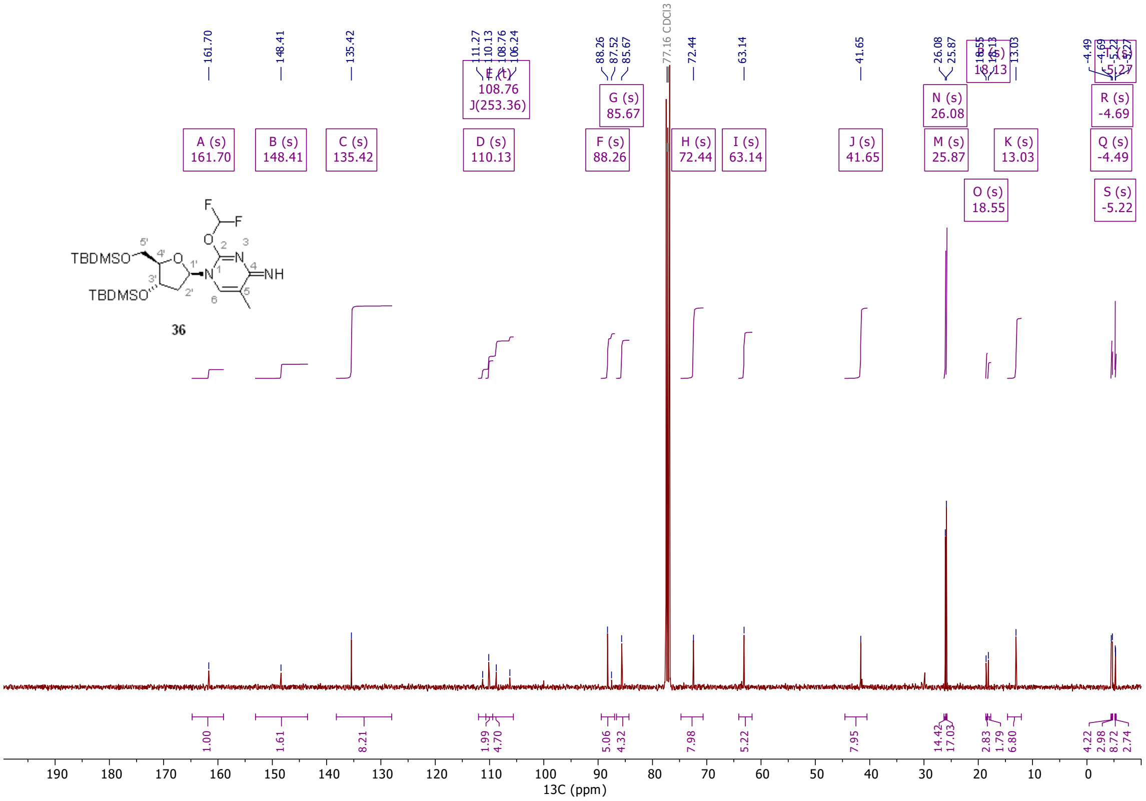

Figure S157 - ^{13}C NMR (101 MHz) spectrum of **36** in $CDCl_3$

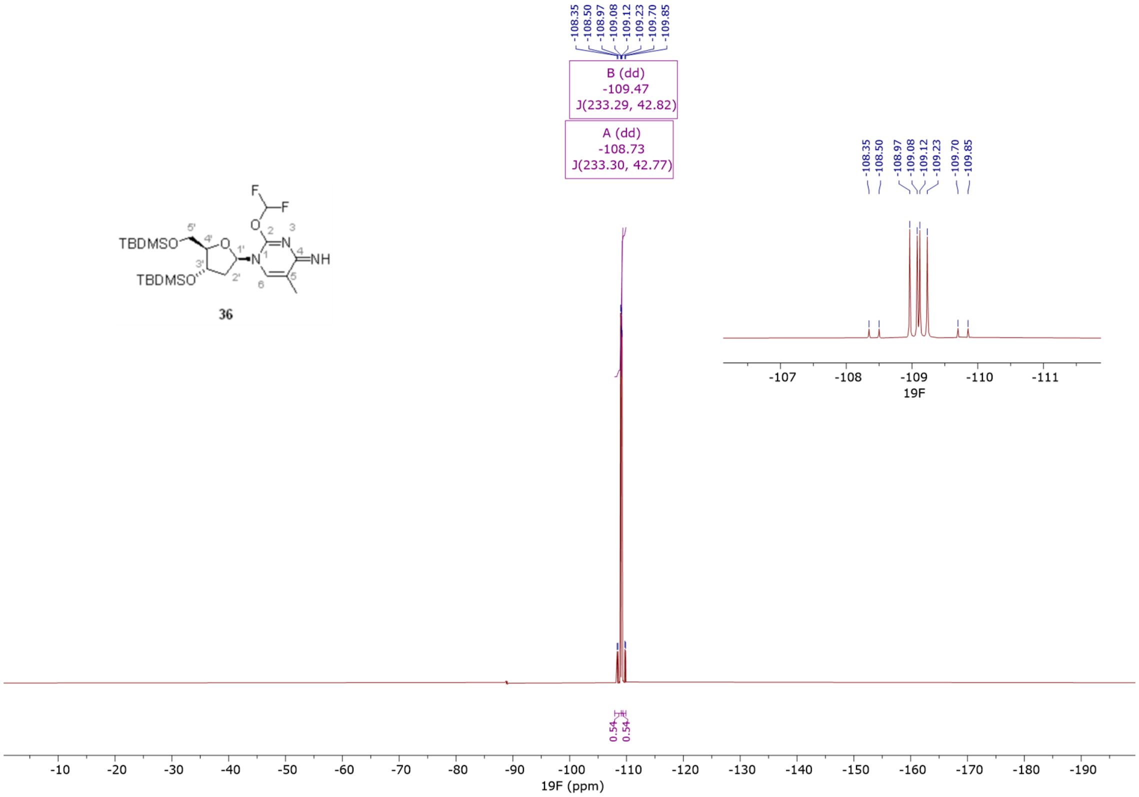

Figure S158 - ^{19}F NMR (376 MHz) spectrum of **36** in $CDCl_3$

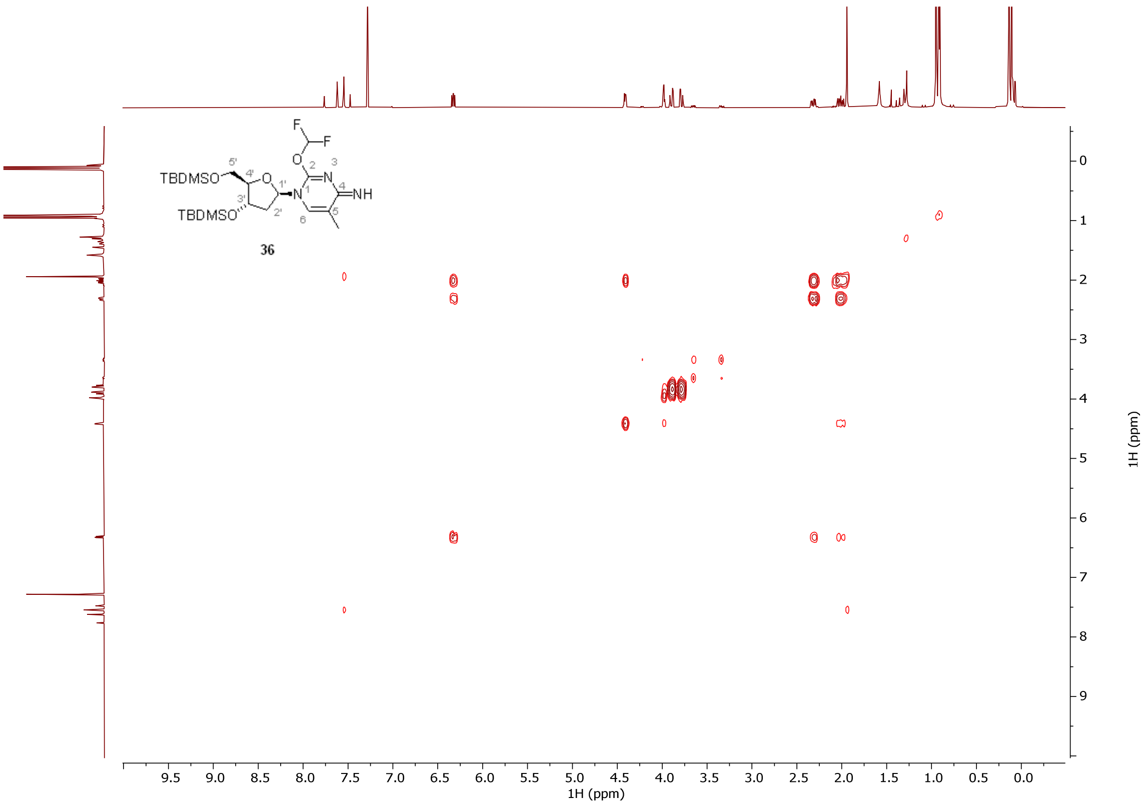

Figure S159 - ^{1}H-^{1}H COSY NMR spectrum of **36** in $CDCl_3$

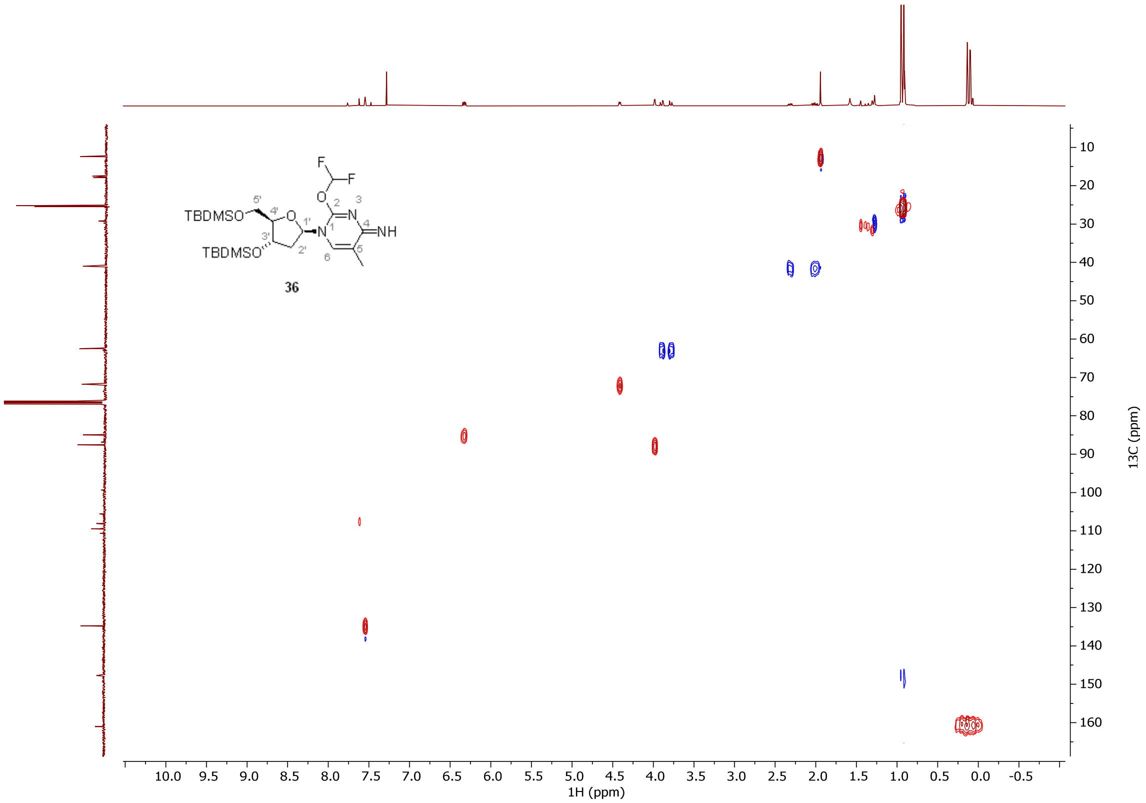

Figure S160 - ^{1}H-^{13}C HSQC NMR spectrum of **36** in $CDCl_3$

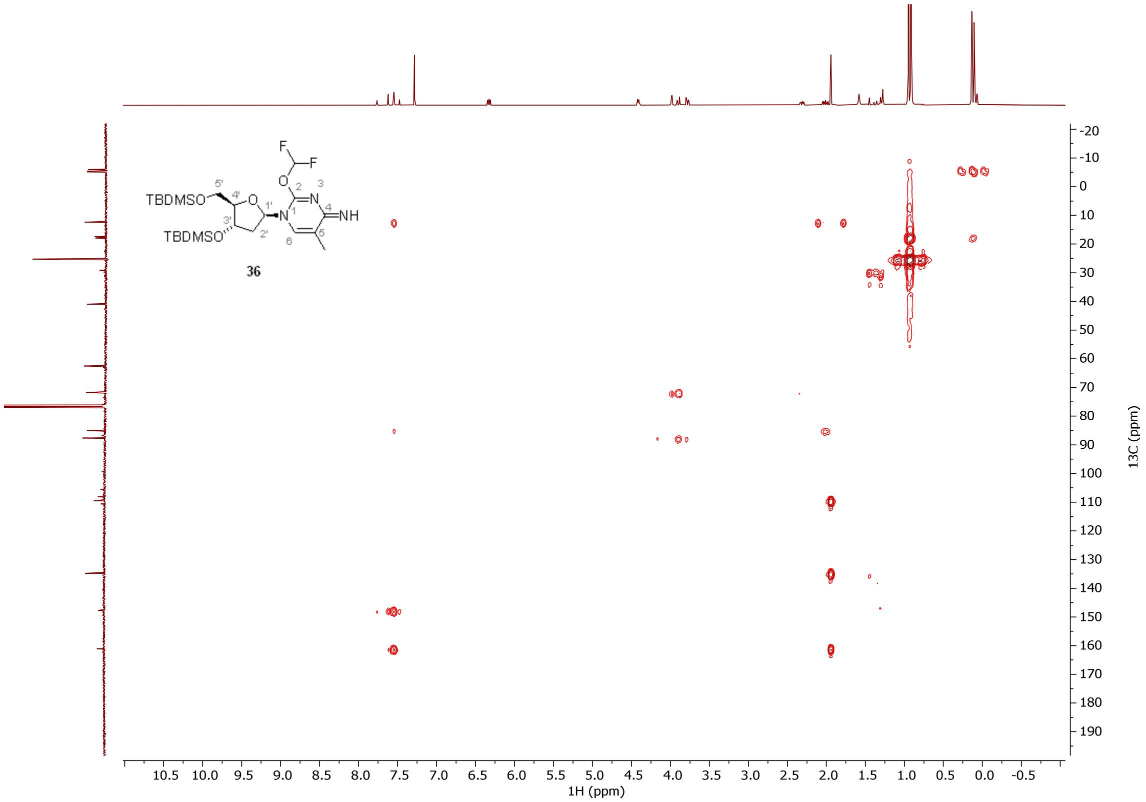

Figure S161 - ^{1}H-^{13}C HMBC NMR spectrum of **36** in $CDCl_3$

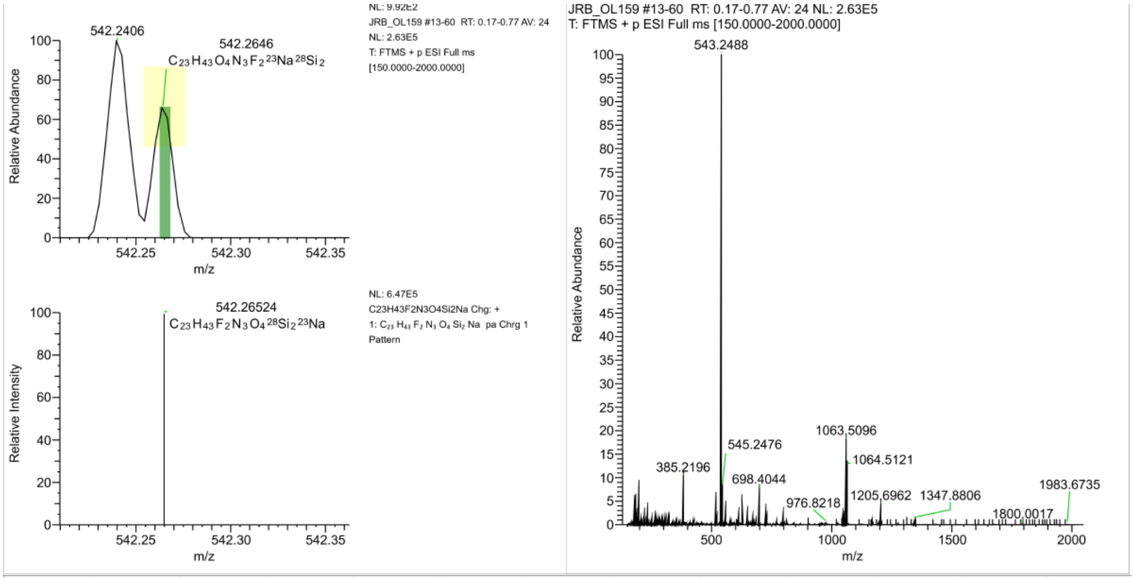

Peak Mass	Display For...	S Fit	RDB	Delta [ppm]	Theo. mass	Rank	Combined S...	# Matched I...	# Missed Iso.	MS Cov. [%]	Pattern Cov....	MSMS Matc...
542.2646	$C_{23}H_{43}O_4N_3F_2{}^{23}Na^{28}Si_2$	17.7416521885253	4.50	-1.22	542.26524	1	1.11	1	2	0.18	69.92	(Collection)
542.2646	$C_{21}H_{41}O_3N_6F_2{}^{23}Na^{28}Si_2$	17.7121955222066	5.00	1.25	542.26389	2	1.11	1	2	0.18	71.05	(Collection)
542.2646	$C_{24}H_{39}N_7F_2{}^{23}Na^{28}Si_2$	12.9695281188229	9.50	-3.69	542.26657	3	0.86	1	2	0.18	69.16	(Collection)
542.2646	$C_{25}H_{45}O_5F_2{}^{23}Na^{28}Si_2$	12.942497955728	4.00	-3.70	542.26658	4	0.85	1	2	0.18	68.92	(Collection)
542.2646	$C_{20}H_{45}O_7N_2F_2{}^{23}Na^{28}Si_2$	12.886174332698	0.00	3.72	542.26256	5	0.85	1	2	0.18	71.89	(Collection)
542.2646	$C_{19}H_{39}O_2N_9F_2{}^{23}Na^{28}Si_2$	12.8589548525202	5.50	3.73	542.26255	6	0.85	1	2	0.18	71.9	(Collection)

Figure S162 -HRMS spectrum of **36**

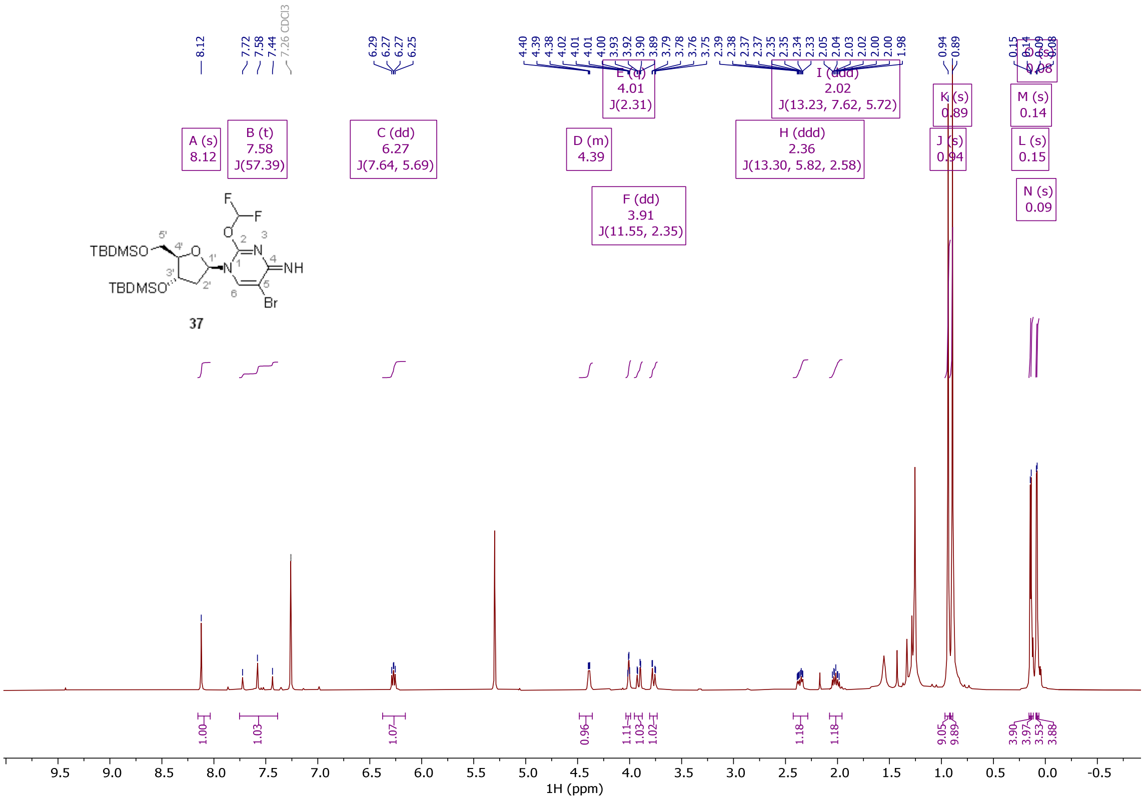

Figure S163 - ^{1}H NMR (400 MHz) spectrum of **37** in $CDCl_3$

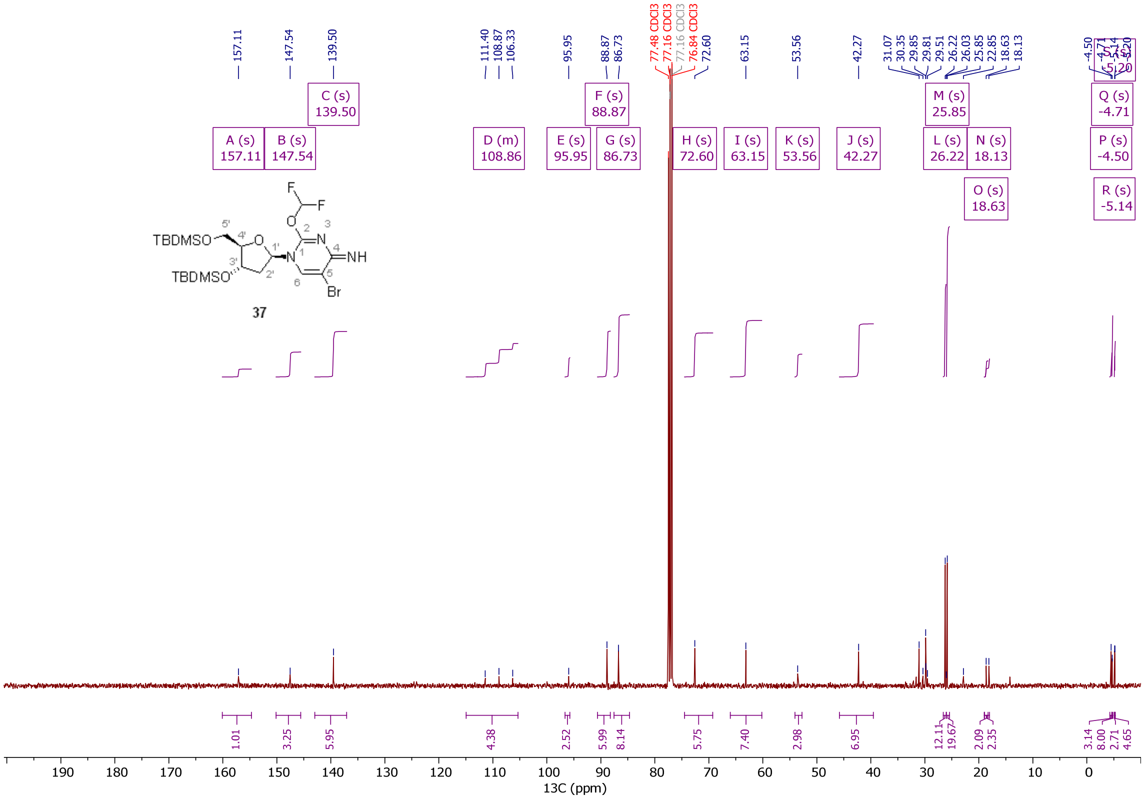

Figure S164 - ^{13}C NMR (101 MHz) spectrum of **37** in $CDCl_3$

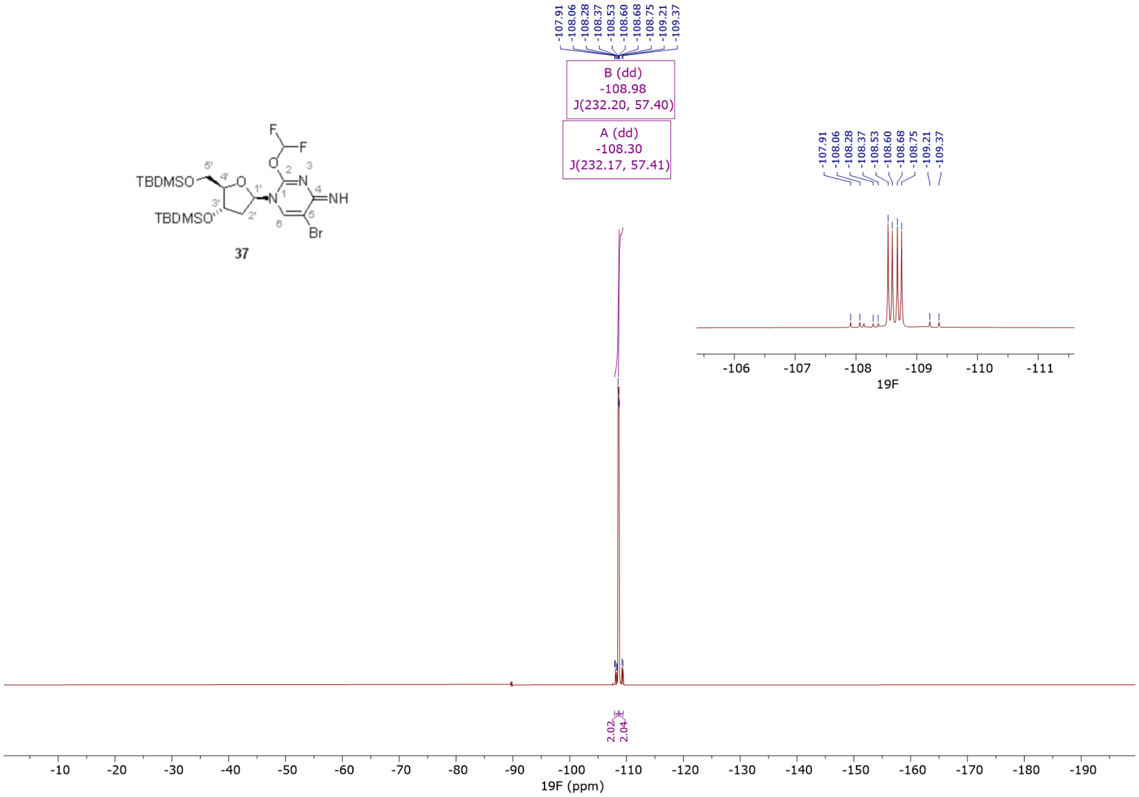

Figure S165 - ^{19}F NMR (376 MHz) spectrum of **37** in $CDCl_3$

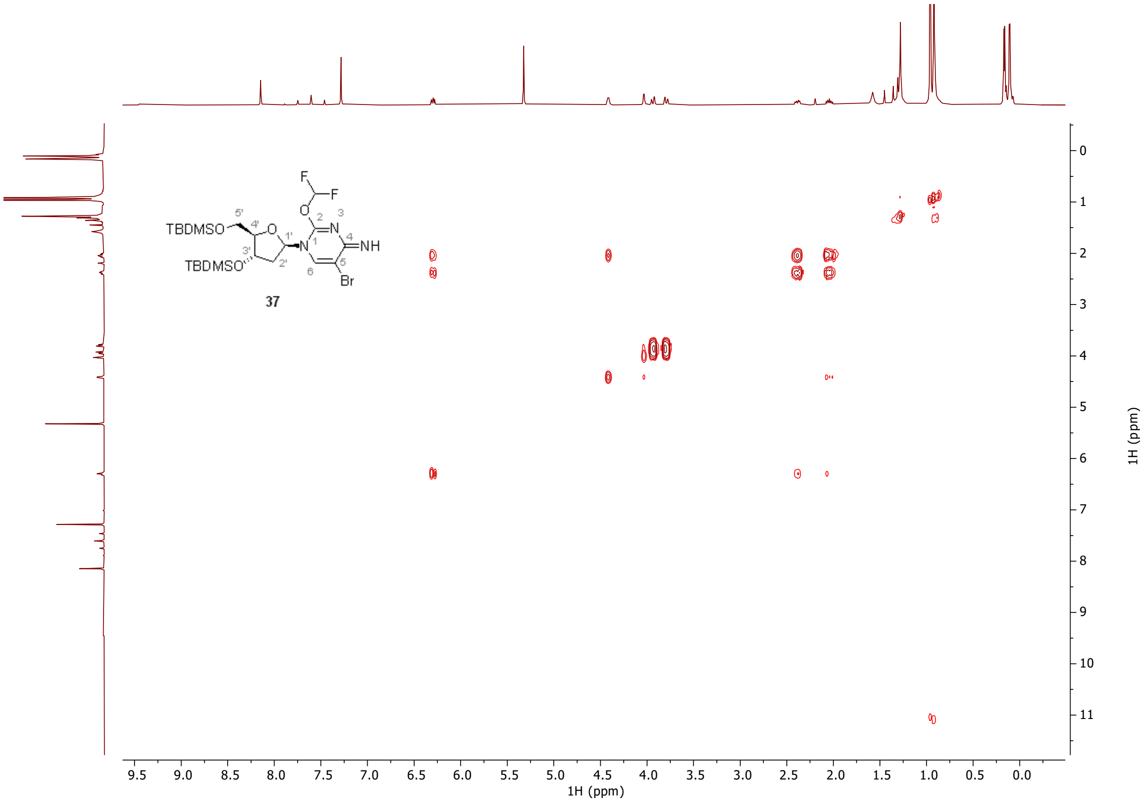

Figure S166 - ^{1}H-^{1}H COSY NMR spectrum of **37** in $CDCl_3$

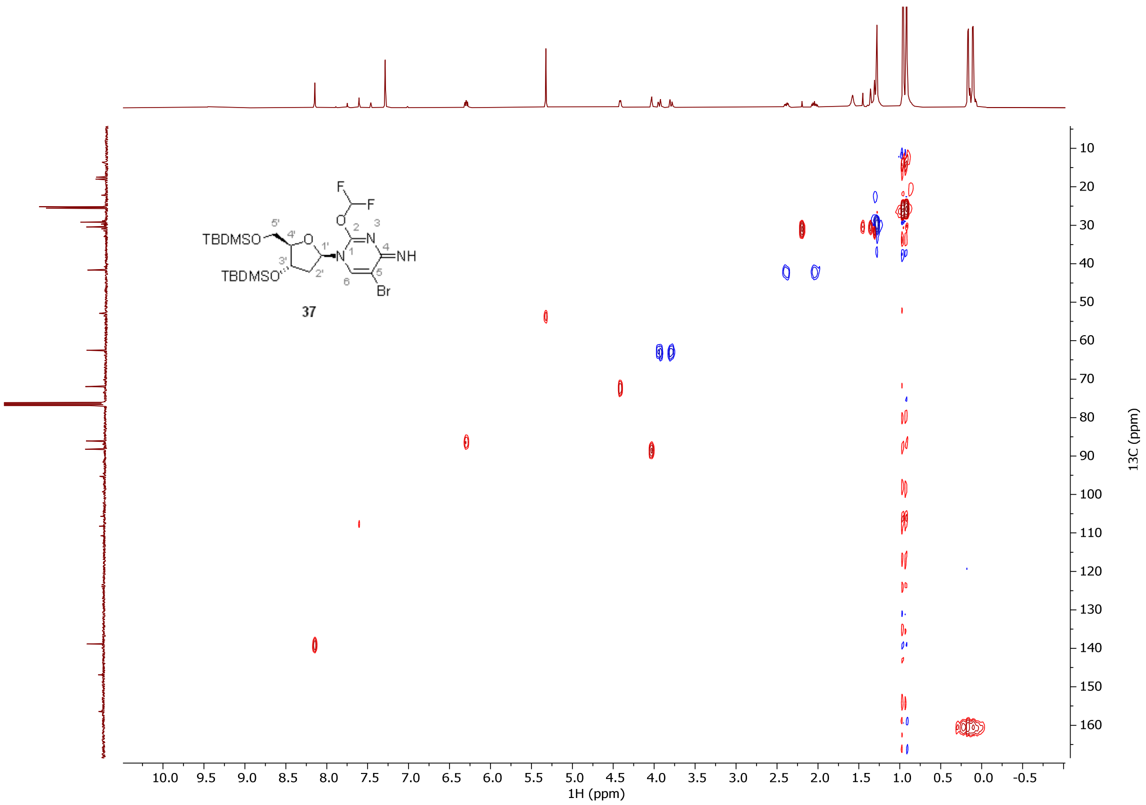

Figure S167 - ^{1}H-^{13}C HSQC NMR spectrum of **37** in $CDCl_3$

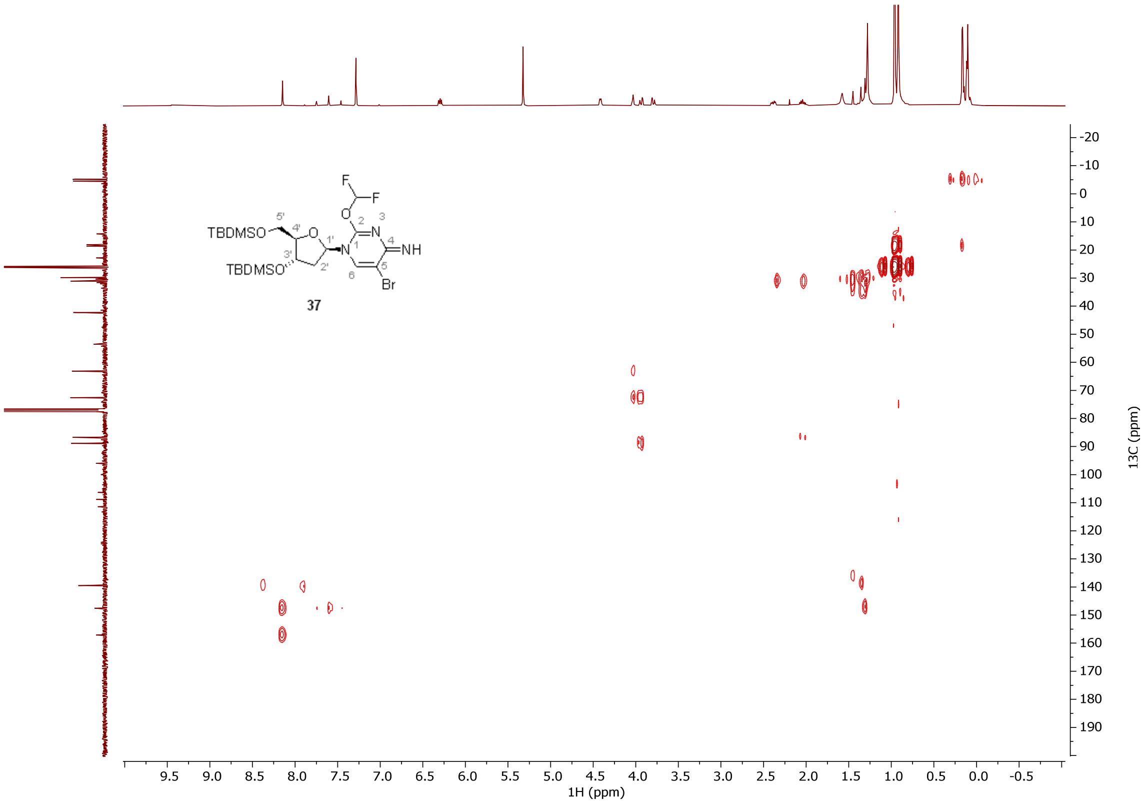

Figure S168 - ^{1}H-^{13}C HMBC NMR spectrum of **37** in $CDCl_3$

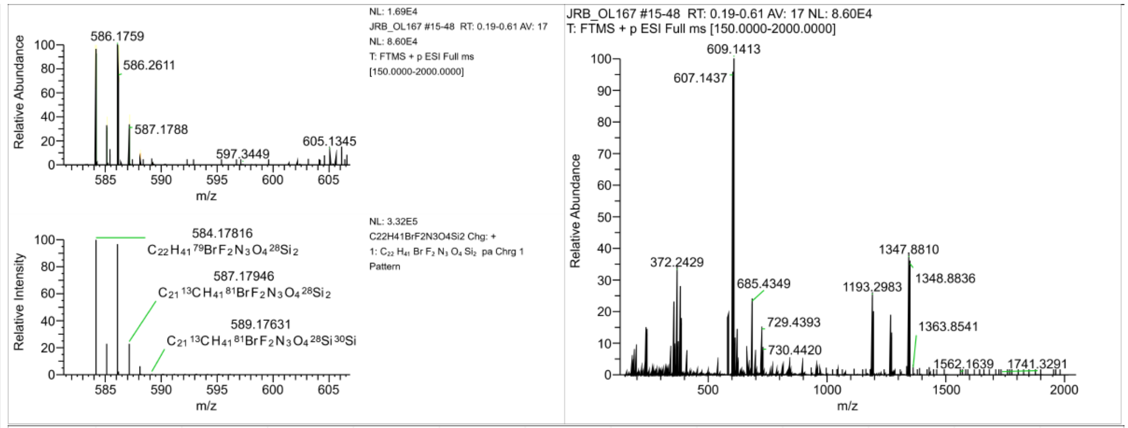

Peak Mass	Display For...	S Fit	RDB	Delta [ppm]	Theo. mass	Rank	Combined S...	# Matched I...	# Missed Iso.	MS Cov. [%]	Pattern Cov....	MSMS Matc...
584.1777	$C_{22}H_{41}O_4N_3{}^{79}BrF_2{}^{28}Si_2$	40.3963503920418	4.50	-0.84	584.17816	1	83.17	6	0	85.55	95.54	(Collection)
584.1777	$C_{23}H_{37}N_7{}^{79}BrF_2{}^{28}Si_2$	34.0853017791608	9.50	-3.13	584.17949	2	82.84	6	0	85.55	95.53	(Collection)
584.1777	$C_{20}H_{39}O_3N_6{}^{79}BrF_2{}^{28}Si_2$	33.8391279322266	5.00	1.46	584.17681	3	82.82	6	0	85.55	95.63	(Collection)
584.1777	$C_{24}H_{43}O_5{}^{79}BrF_2{}^{28}Si_2$	33.3358727449569	4.00	-3.14	584.17950	4	82.8	6	0	85.55	95.4	(Collection)
584.1777	$C_{19}H_{43}O_7N_2{}^{79}BrF_2{}^{28}Si_2$	12.3176936983145	0.00	3.75	584.17548	5	62.99	6	0	65.81	73.54	(Collection)
584.1777	$C_{18}H_{37}O_2N_9{}^{79}BrF_2{}^{28}Si_2$	12.1841197270049	5.50	3.76	584.17547	6	62.99	6	0	65.81	73.37	(Collection)
584.1777	$C_{20}H_{34}O_{10}N_4F_2{}^{28}Si_2$	20.6649813645165	7.00	0.10	584.17760	7	40.79	2	3	41.91	90.4	(Collection)
584.1777	$C_{22}H_{36}O_{11}NF_2{}^{28}Si_2$	19.8346196909368	6.50	-2.20	584.17895	8	40.75	2	3	41.91	90.1	(Collection)
584.1777	$C_{21}H_{30}O_6N_8F_2{}^{28}Si_2$	19.7055842034453	12.00	-2.19	584.17894	9	40.74	2	3	41.91	90.46	(Collection)
584.1777	$C_{18}H_{32}O_9N_7F_2{}^{28}Si_2$	19.3456205595464	7.50	2.40	584.17626	10	40.72	2	2	41.91	90.84	(Collection)

Figure S169 - HRMS spectrum of **37**

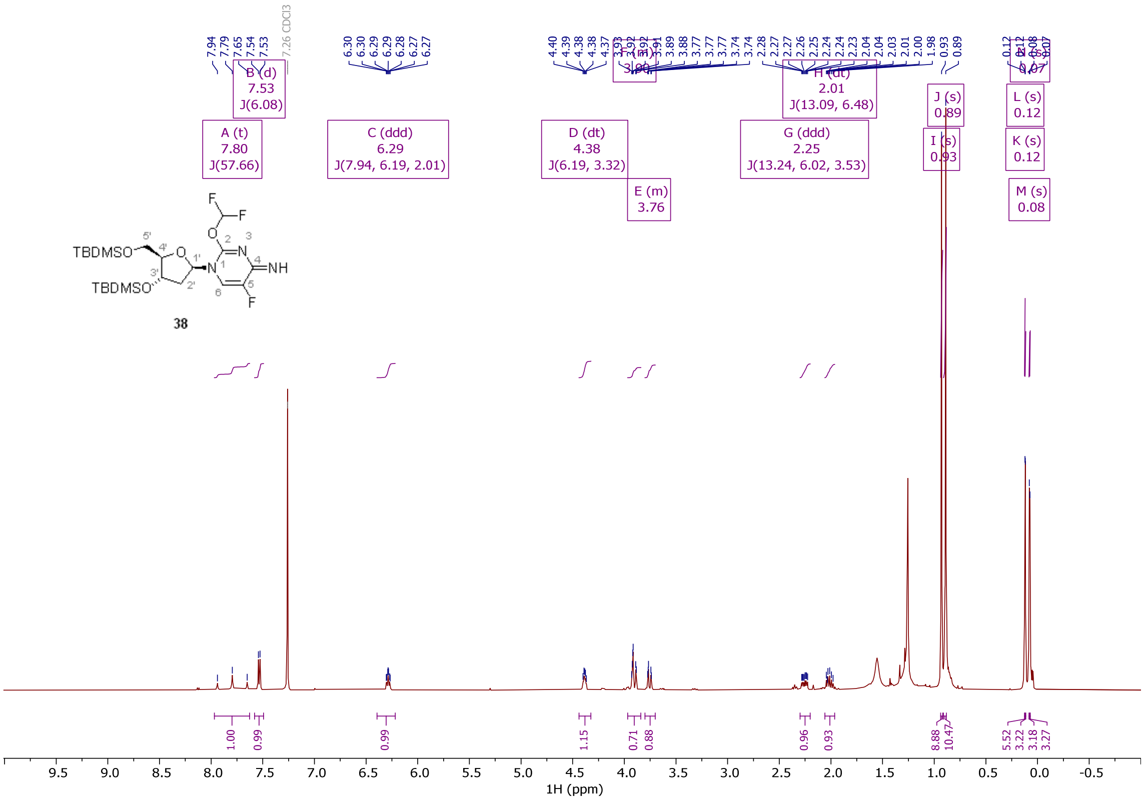

Figure S170 - ^{1}H NMR (400 MHz) spectrum of **38** in $CDCl_3$

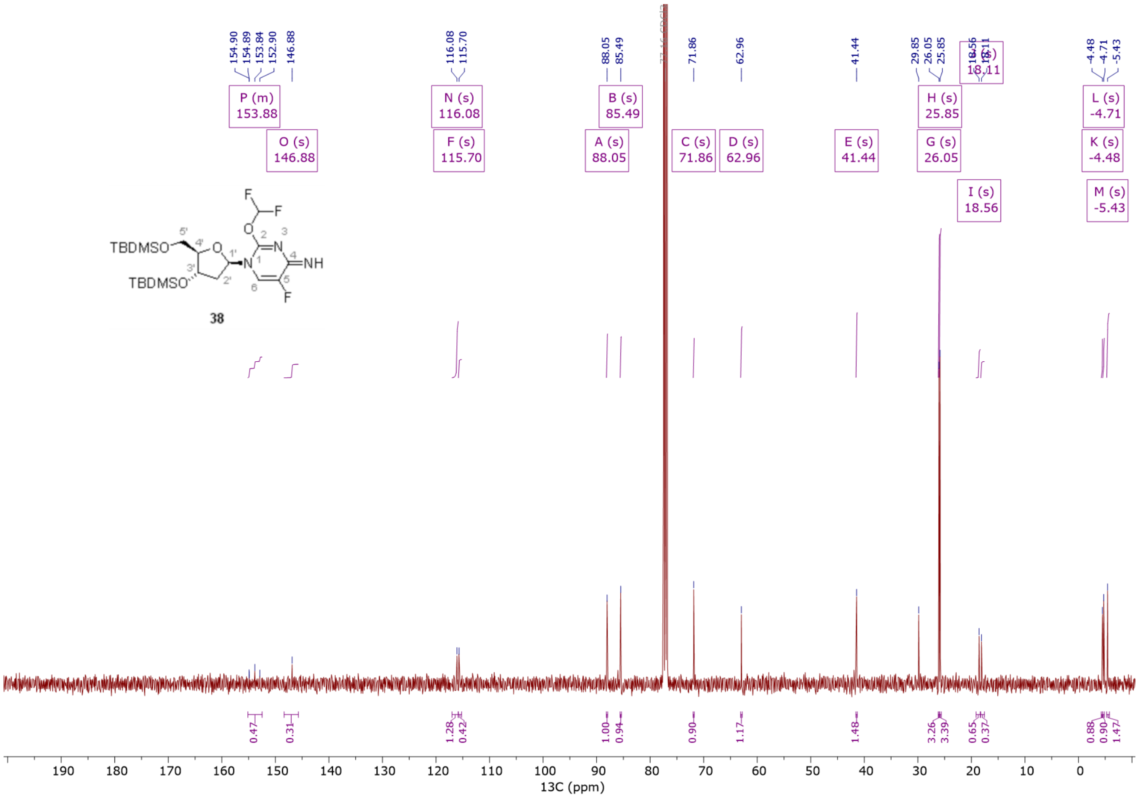

Figure S171 - ^{13}C NMR (101 MHz) spectrum of **38** in $CDCl_3$

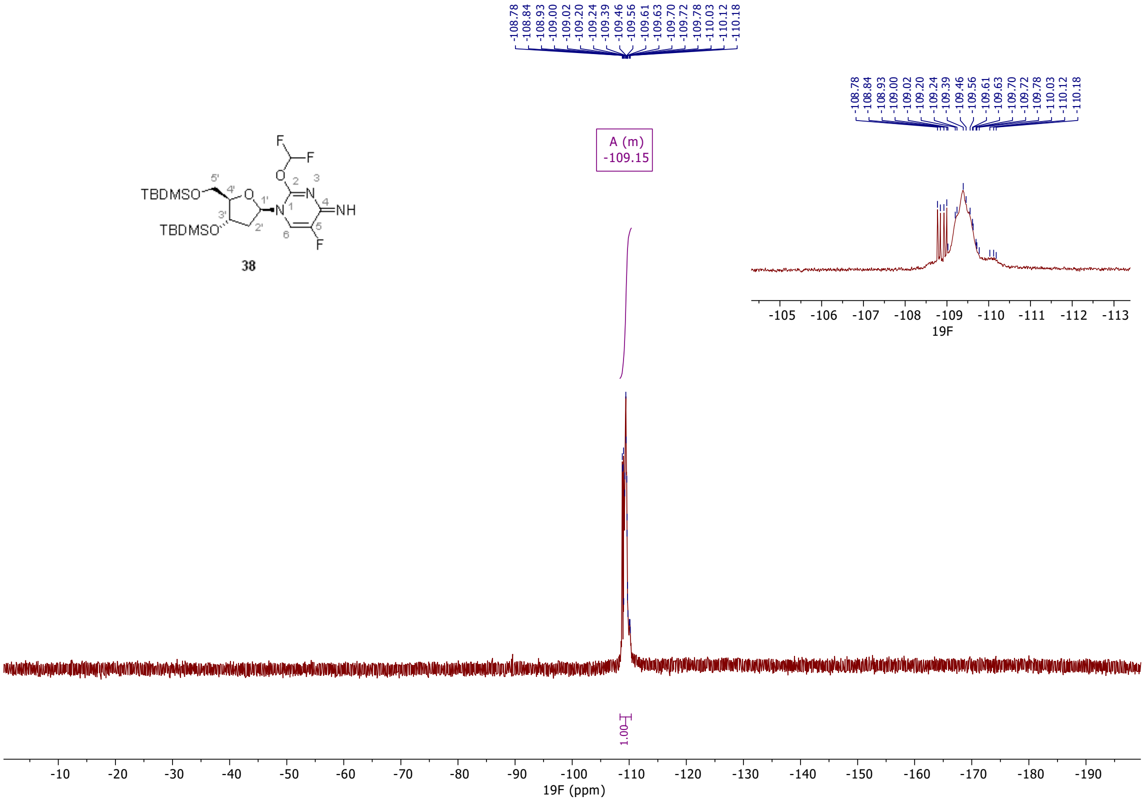

Figure S172 - ^{19}F NMR (376 MHz) spectrum of **38** in $CDCl_3$

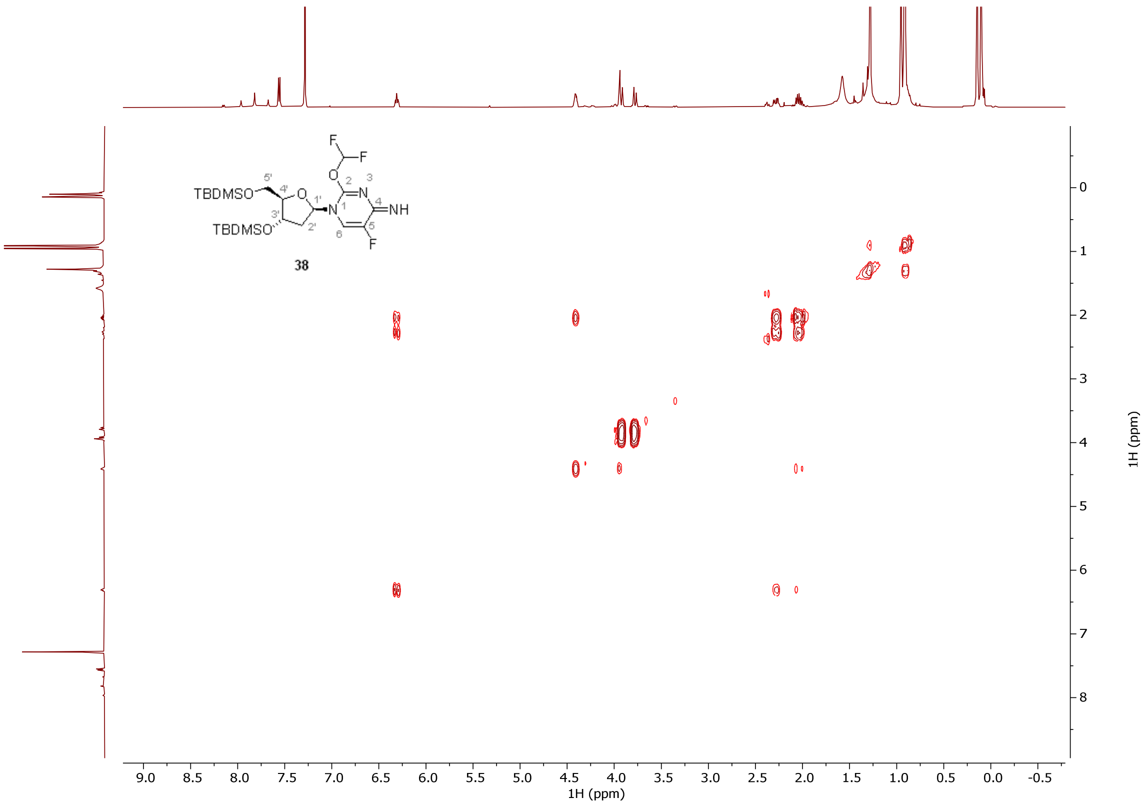

Figure S173 - ^{1}H-^{1}H COSY NMR spectrum of **38** in $CDCl_3$

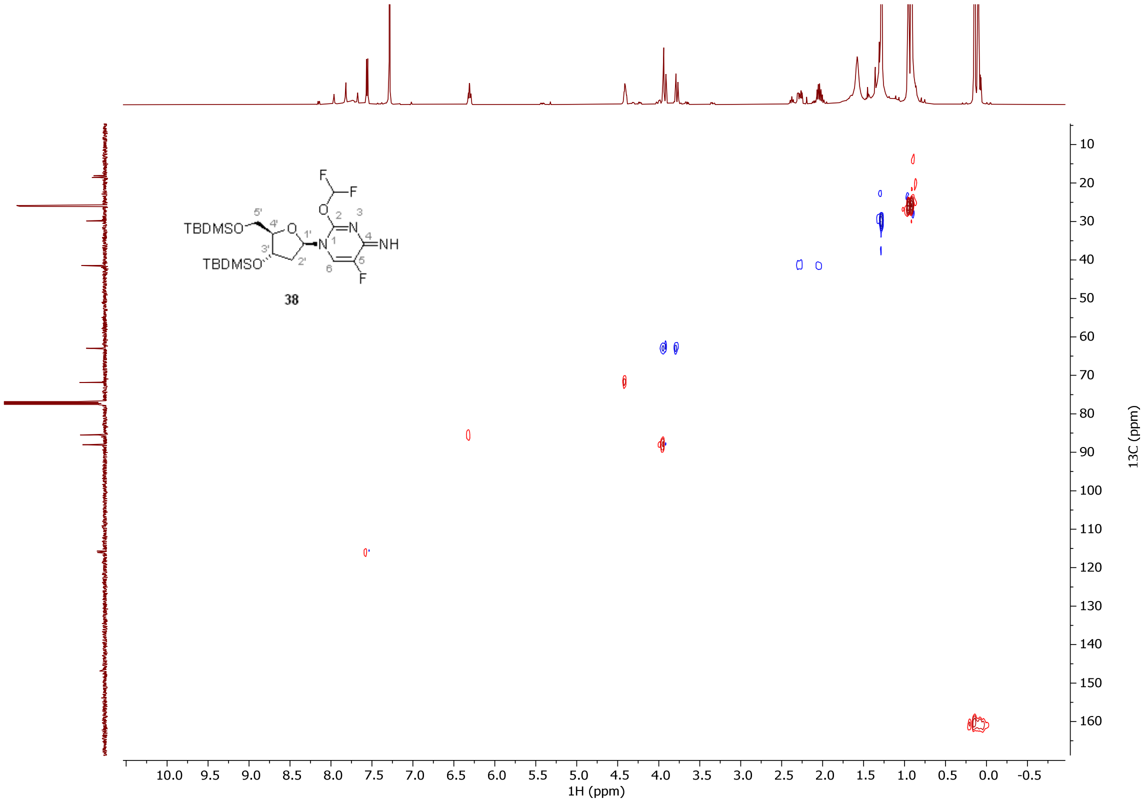

Figure S174 - ^{1}H-^{13}C HSQC NMR spectrum of **38** in $CDCl_3$

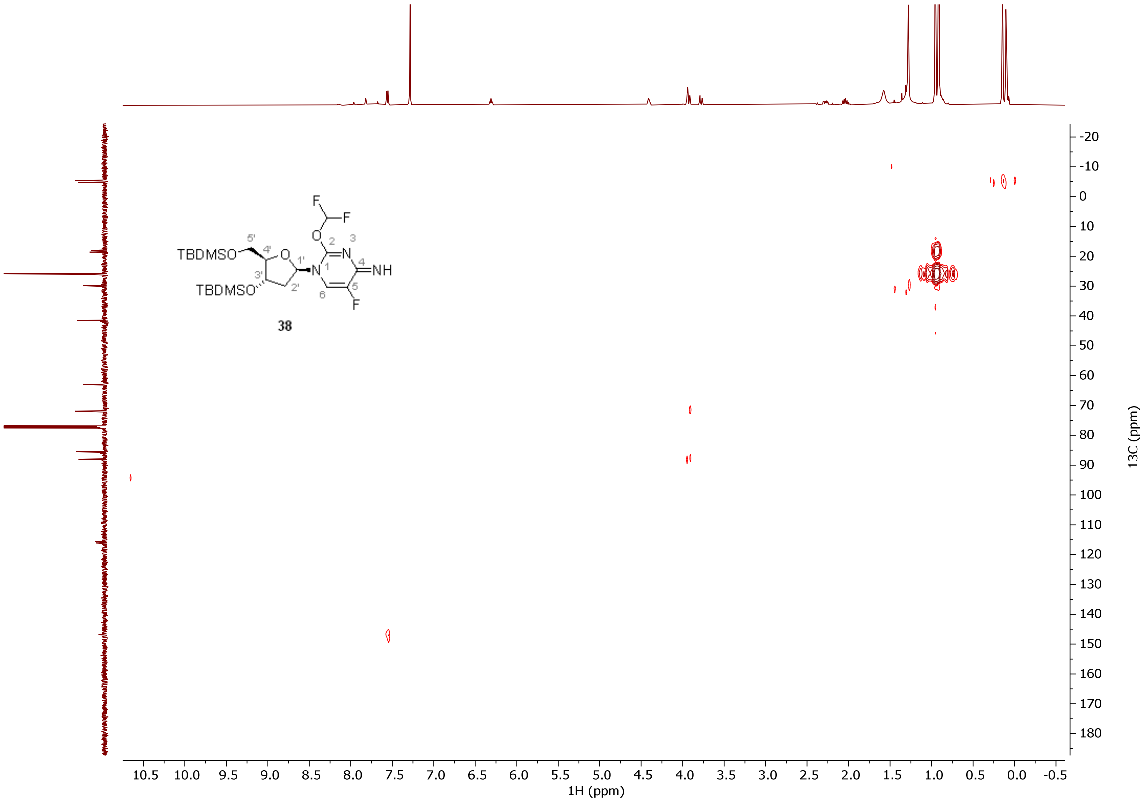

Figure S175 - ^{1}H-^{13}C HMBC NMR spectrum of **38** in $CDCl_3$

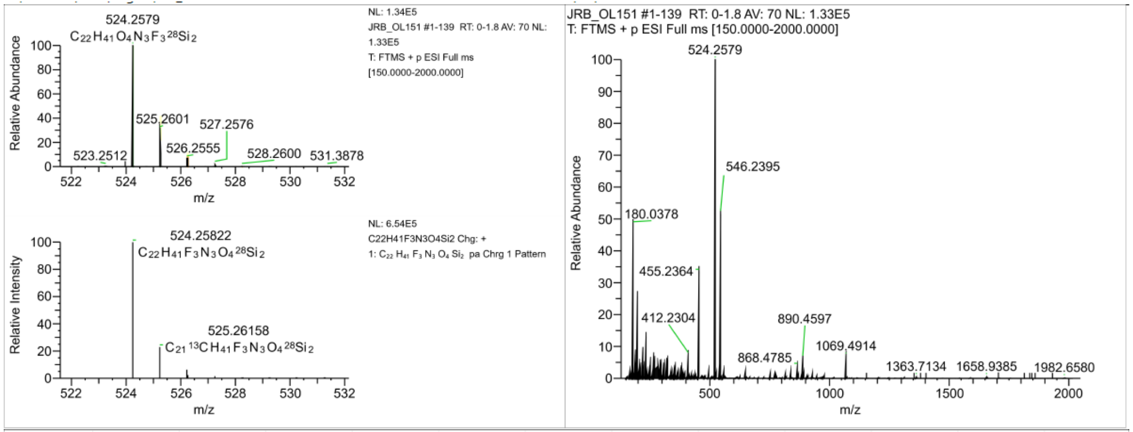

Peak Mass	Display For...	S Fit	RDB	Delta [ppm]	Theo. mass	Rank	Combined S...	# Matched I...	# Missed Iso.	MS Cov. [%]	Pattern Cov....	MSMS Matc...
524.2579	$C_{23}H_{38}O_2N_6F_2{}^{28}Si_2$	27.4119426658998	9.00	4.14	524.25574	1	95.82	5	0	99.62	99.76	(Collection)
524.2579	$C_{21}H_{35}O_4N_9F{}^{28}Si$	23.922165614315	9.50	3.67	524.25598	2	94.42	5	0	98.34	99.06	(Collection)
524.2579	$C_{22}H_{41}O_4N_3F_3{}^{28}Si_2$	30.6454986227713	4.50	-0.60	524.25822	3	91.29	5	0	94.66	93.15	(Collection)
524.2579	$C_{23}H_{37}O_5N_6F{}^{28}Si$	34.5843974870862	9.00	1.11	524.25732	4	90.39	5	0	93.49	94.4	(Collection)
524.2579	$C_{22}H_{41}O_9N_2F{}^{28}Si$	27.7088925967594	4.00	3.66	524.25599	5	90.02	5	0	93.49	94.15	(Collection)
524.2579	$C_{22}H_{31}O_2N_{10}F_3$	23.3917734163519	11.00	0.19	524.25781	6	89.69	2	2	93.37	96.96	(Collection)
524.2579	$C_{23}H_{37}O_7N_3F_3$	23.1118130177744	5.50	0.18	524.25781	7	89.68	3	1	93.37	96.07	(Collection)
524.2579	$C_{21}H_{44}O_9N_2{}^{28}Si_2$	22.4909260250729	3.00	-0.16	524.25799	8	89.64	5	0	93.37	91.17	(Collection)
524.2579	$C_{21}H_{34}O_7N_9$	21.1388964710206	9.50	0.64	524.25757	9	89.57	3	1	93.37	96.24	(Collection)
524.2579	$C_{22}H_{40}O_5N_6{}^{28}Si_2$	18.6552372746429	8.00	-2.71	524.25932	10	89.44	5	0	93.37	91.19	(Collection)

Figure S176 - HRMS spectrum of **38**

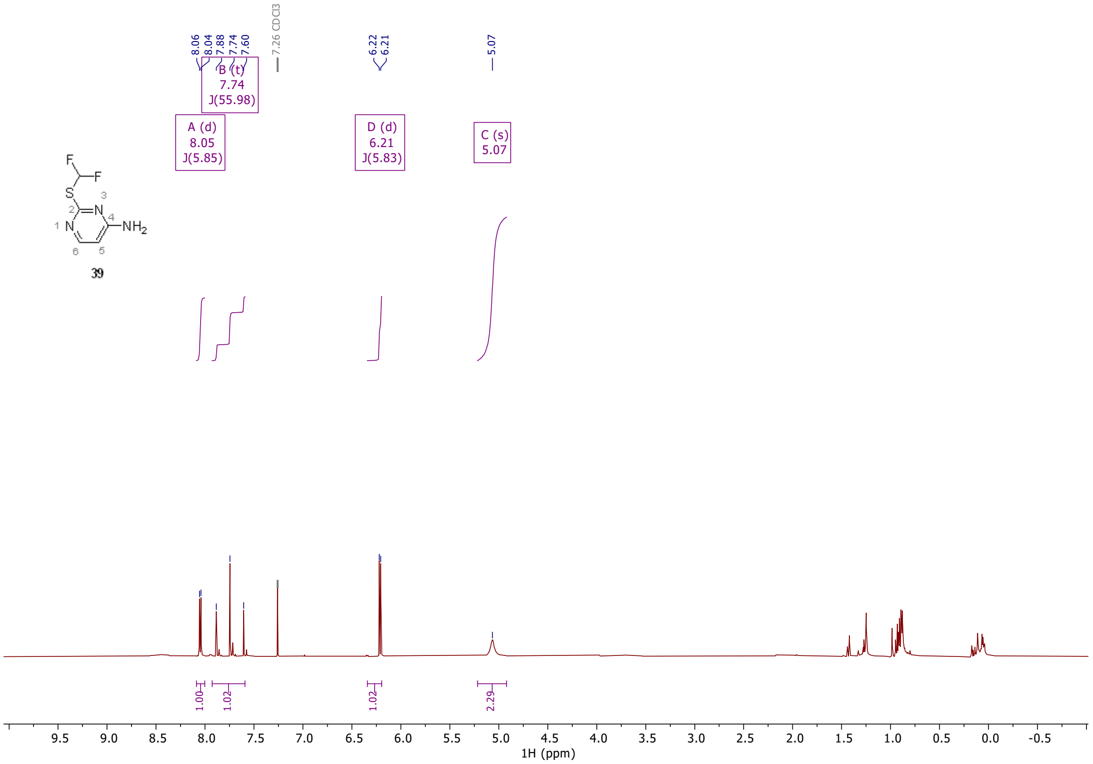

Figure S177 - ^{1}H NMR (400 MHz) spectrum of **39** in $CDCl_3$

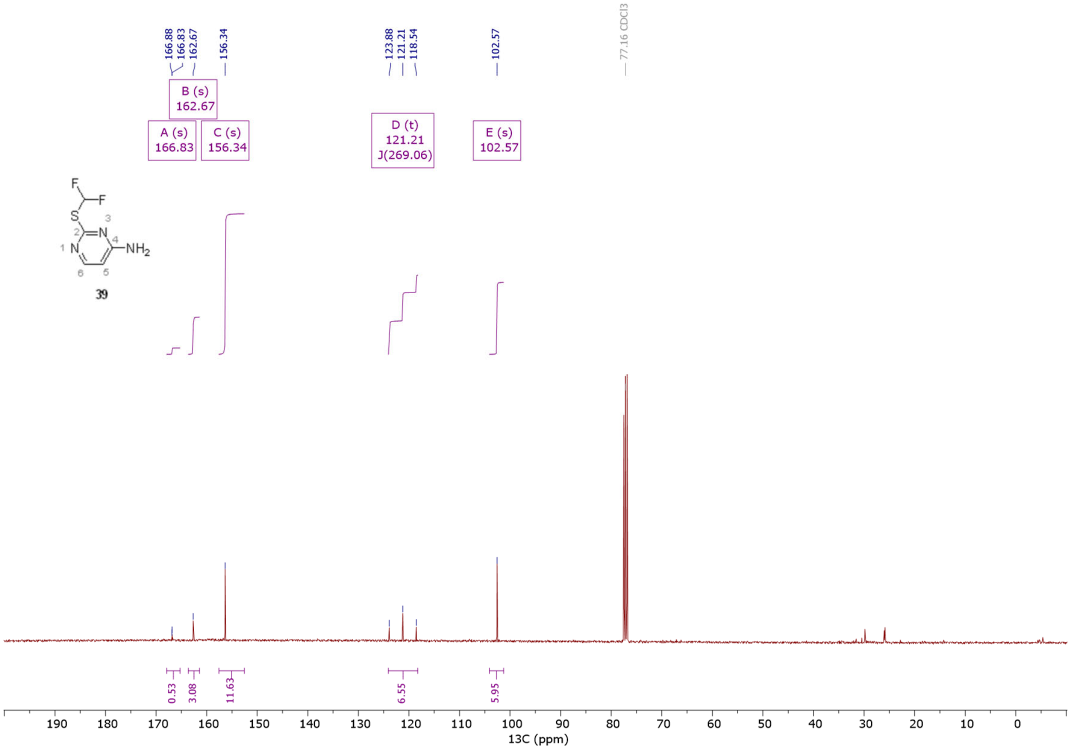

Figure S178 - ^{13}C NMR (101 MHz) spectrum of **39** in $CDCl_3$

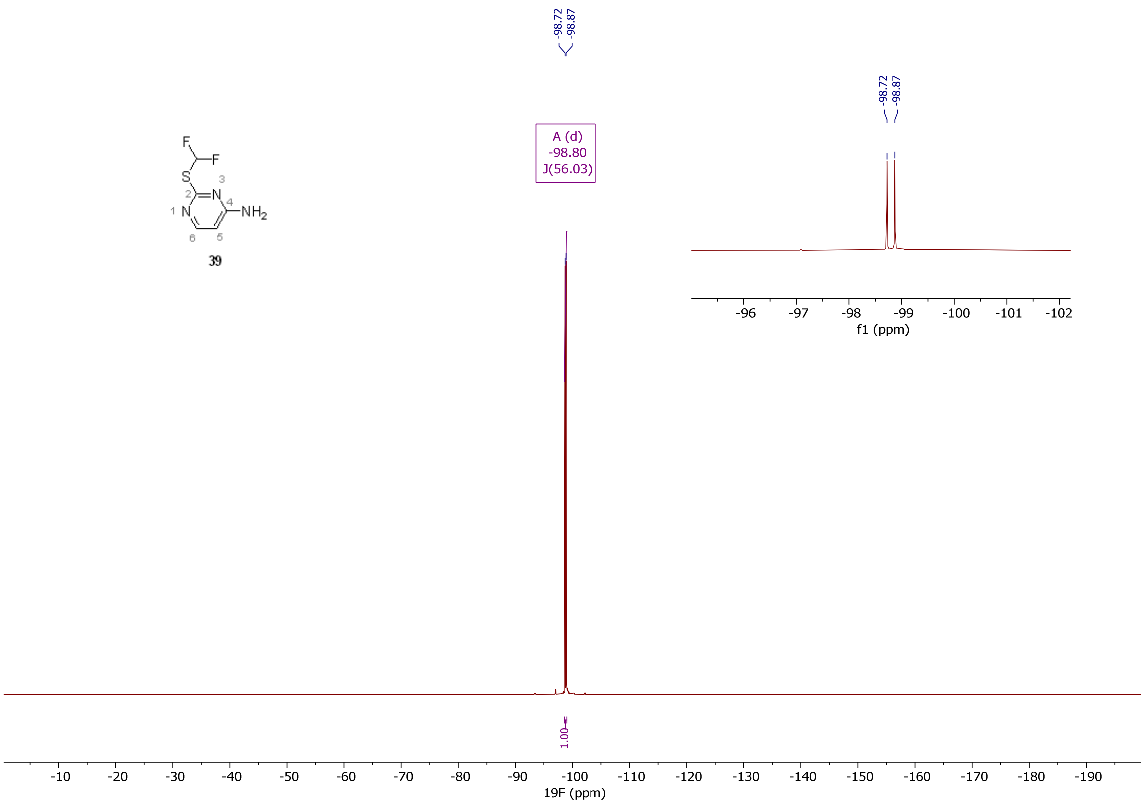

Figure S179 - ^{19}F NMR (376 MHz) spectrum of **39** in $CDCl_3$

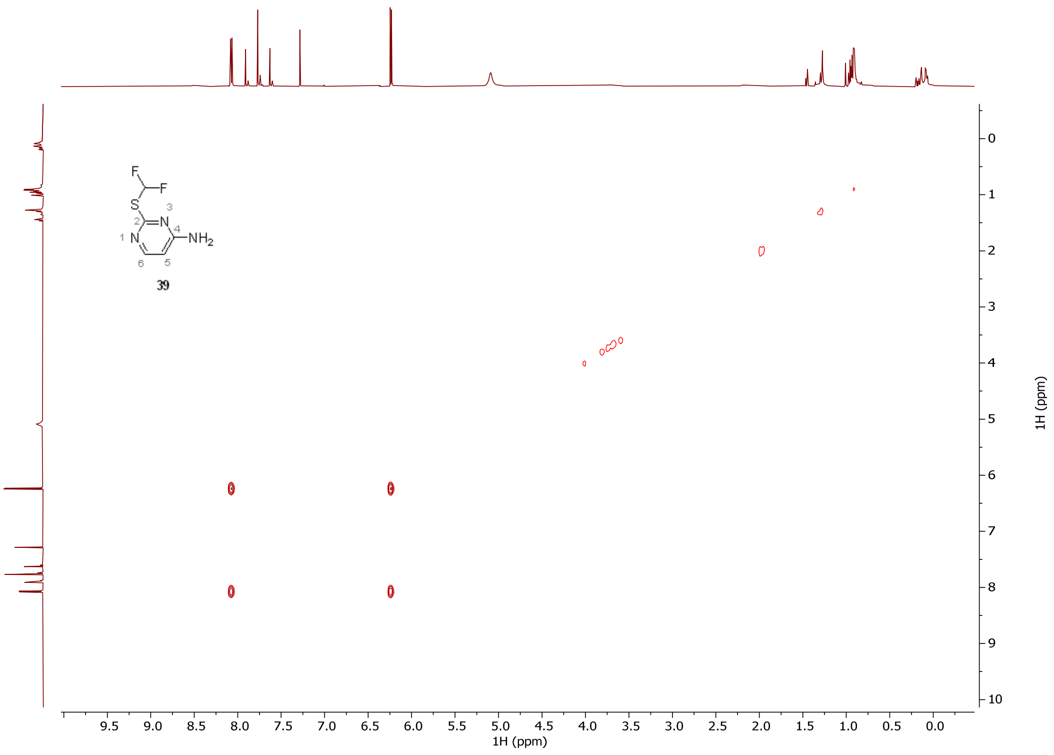

Figure S180 - ^{1}H-^{1}H COSY NMR spectrum of **39** in $CDCl_3$

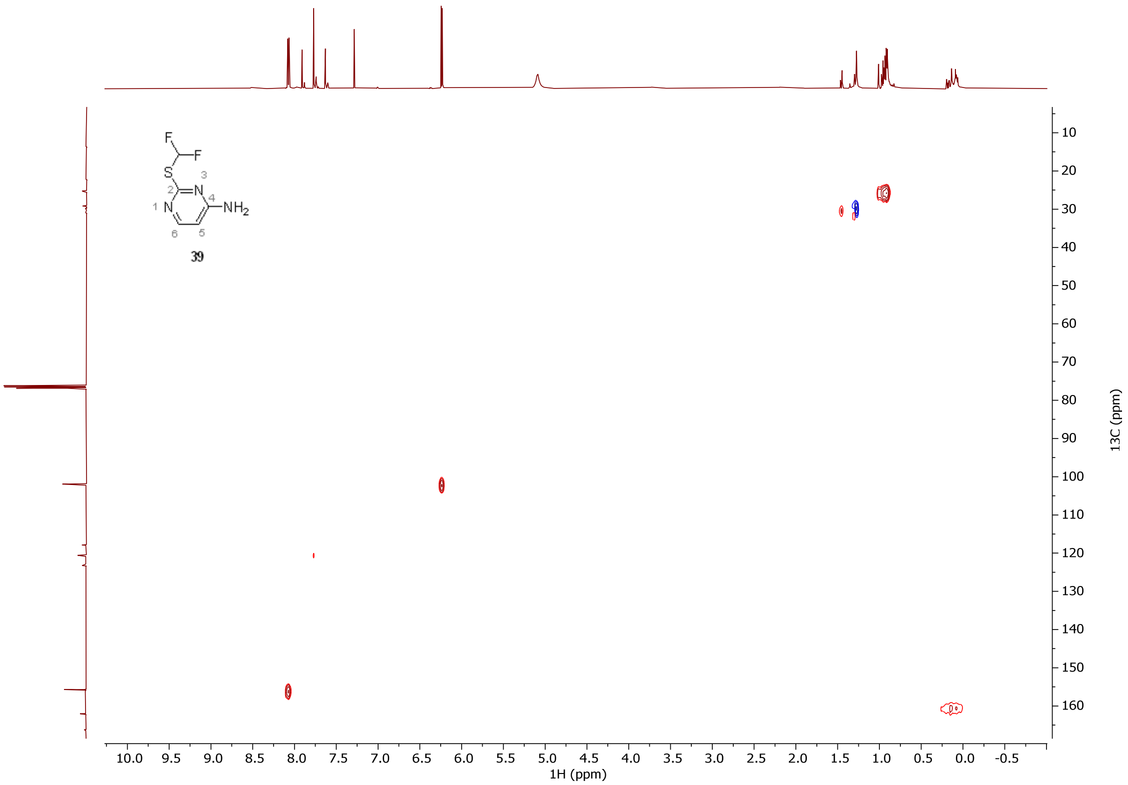

Figure S181 - ^{1}H-^{13}C HSQC NMR spectrum of **39** in $CDCl_3$

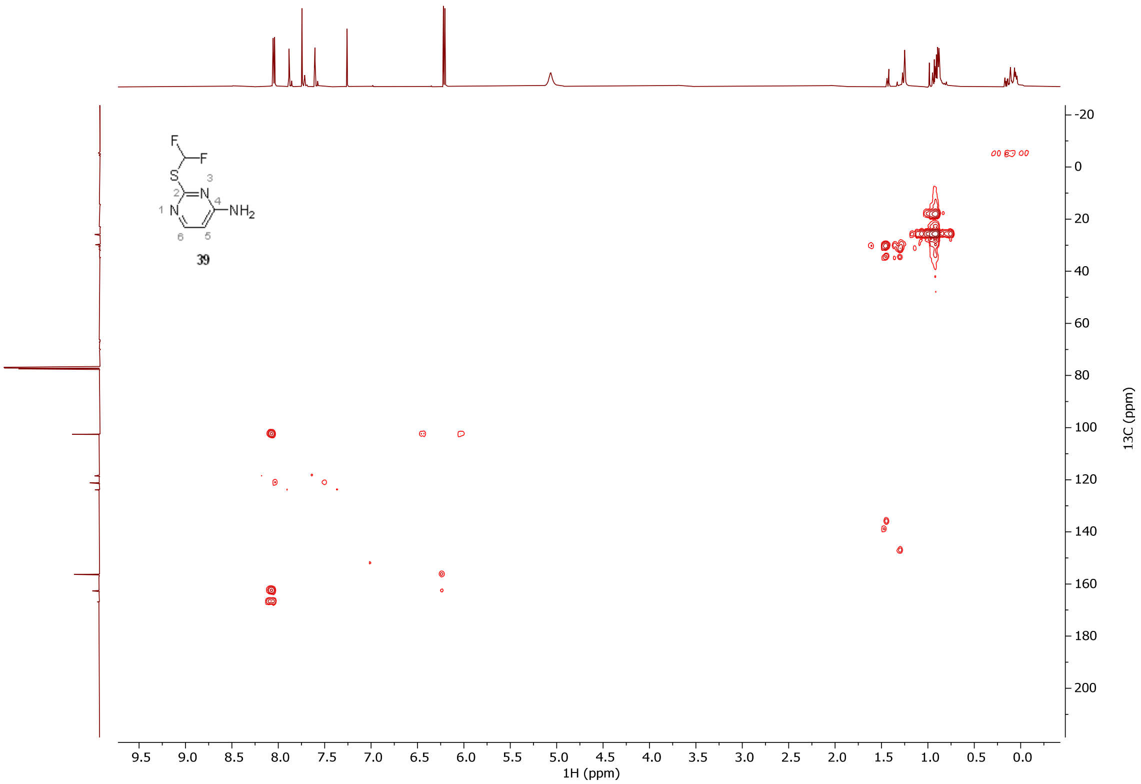

Figure S182 - ^{1}H-^{13}C HMBC NMR spectrum of **39** in $CDCl_3$

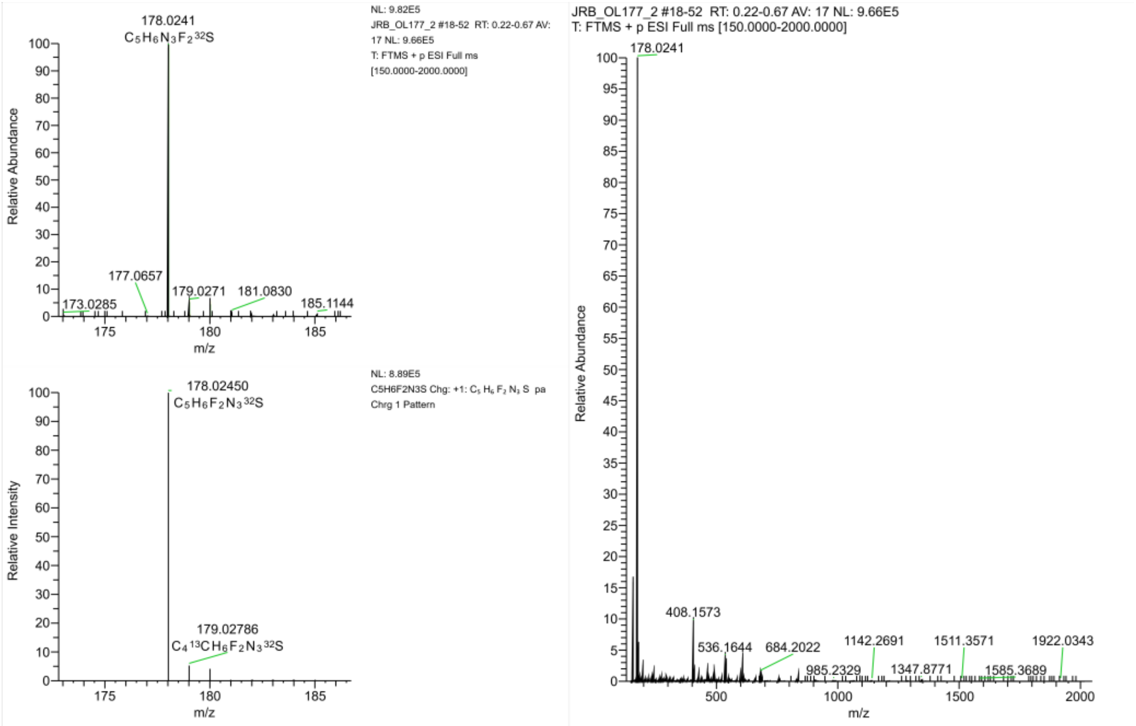

Peak Mass	Display For...	S Fit	RDB	Delta [ppm]	Theo. mass	Rank	Combined S...	# Matched I...	# Missed Iso.	MS Cov. [%]	Pattern Cov....	MSMS Matc...
178.0241	$C_5H_6N_3F_2{}^{32}S$	24.01992016 38469	3.50	-2.28	178.02450	1	94.64	7	0	98.56	98.1	(Collection)

Figure S183 - HRMS spectrum of **39**

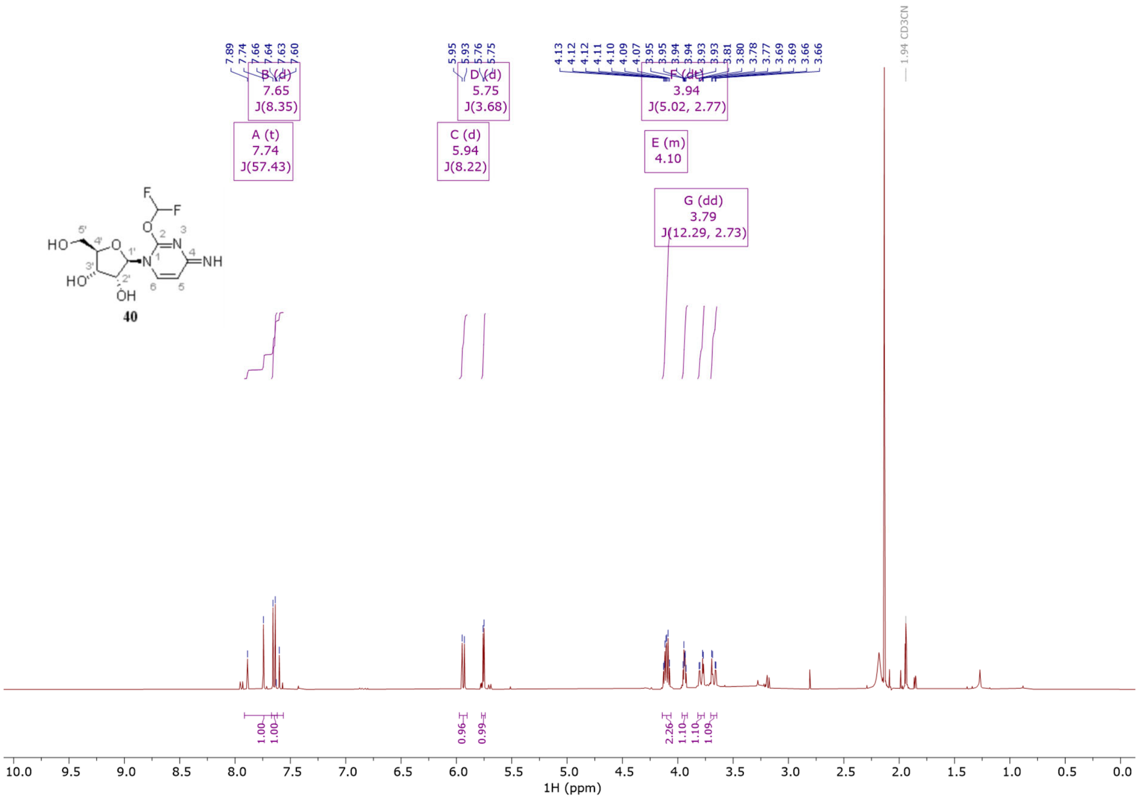

Figure S184 - ^{1}H NMR (400 MHz) spectrum of **40** in CD_3CN

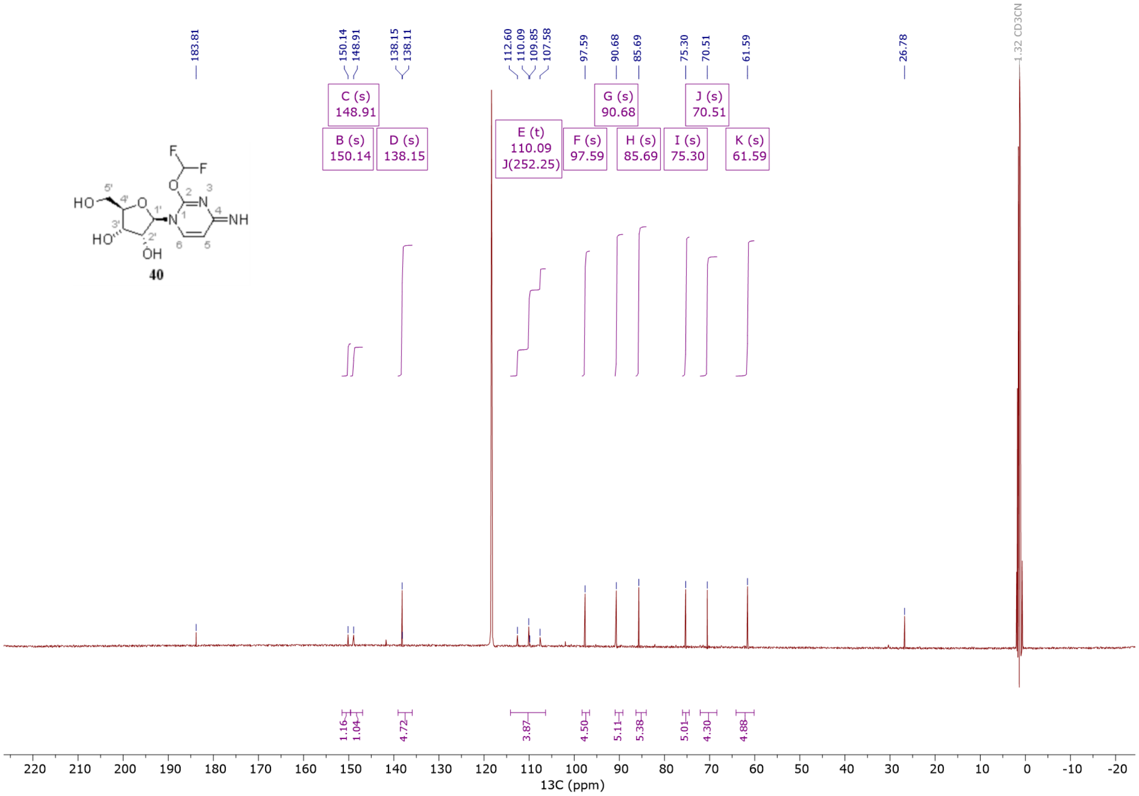

Figure S185 - ^{13}C NMR (101 MHz) spectrum of **40** in CD_3CN

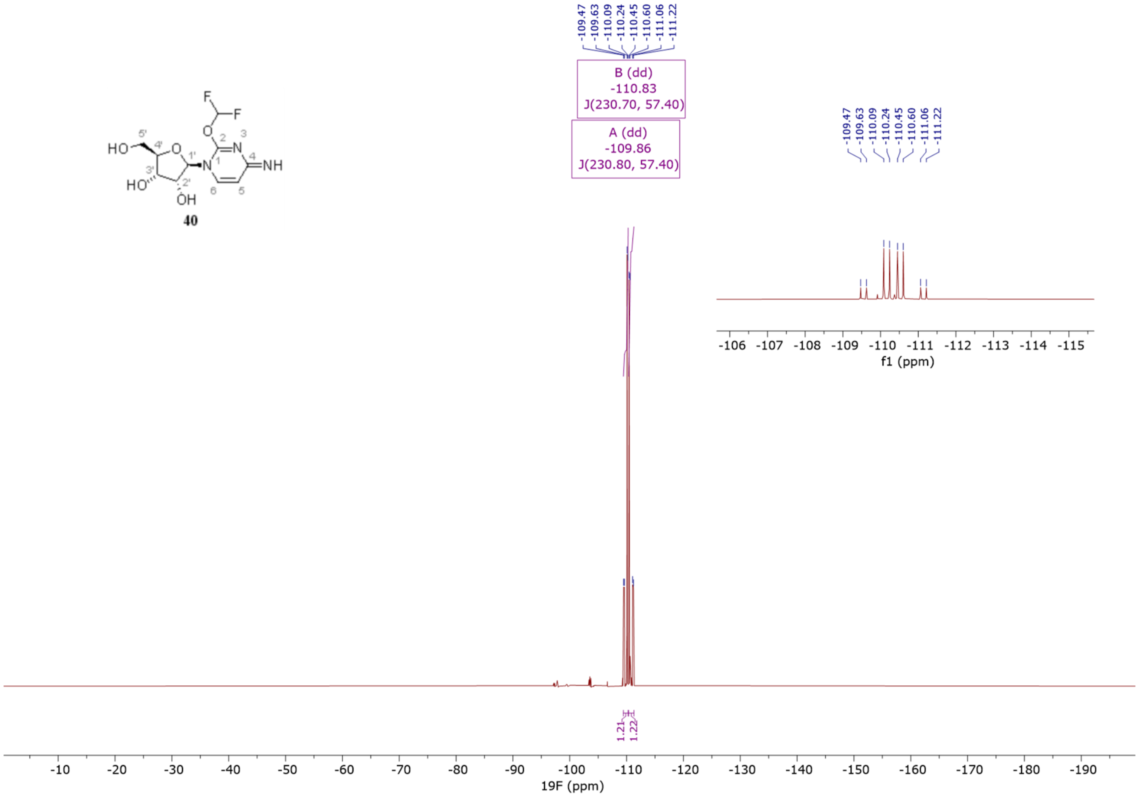

Figure S186 - ^{19}F NMR (376 MHz) spectrum of **40** in CD_3CN

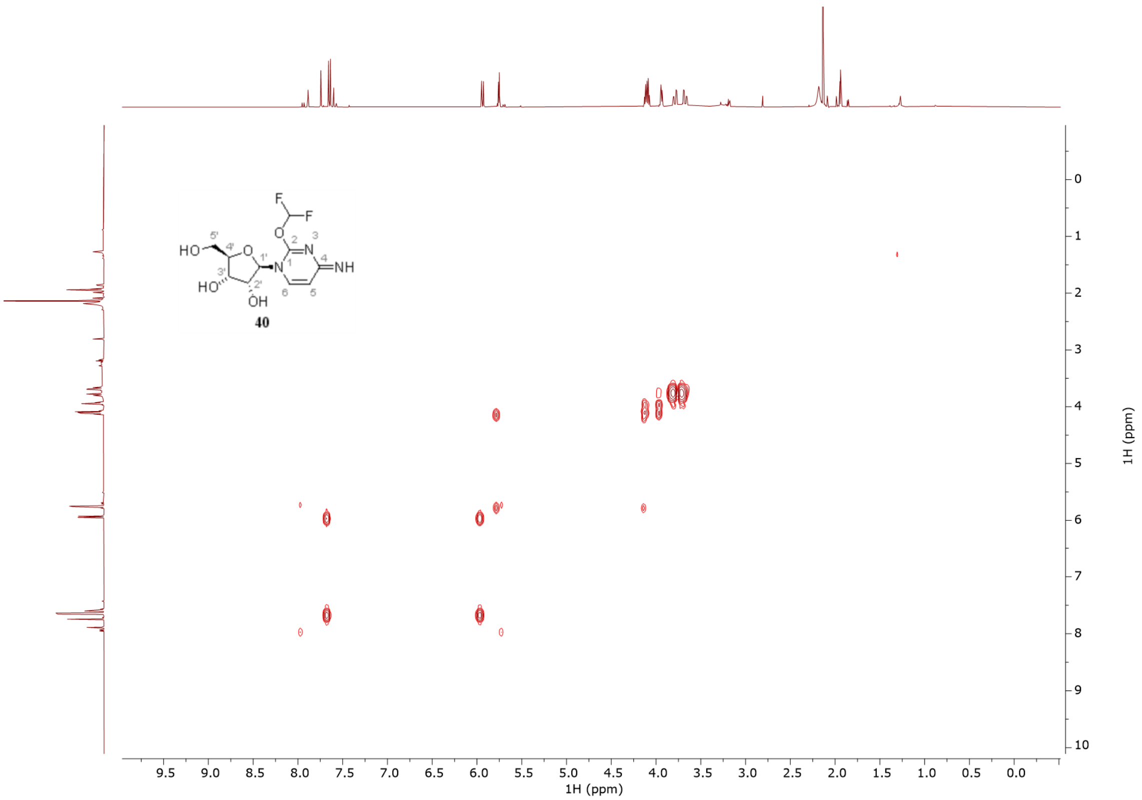

Figure S187 - ^{1}H-^{1}H COSY NMR spectrum of **40** in CD_3CN

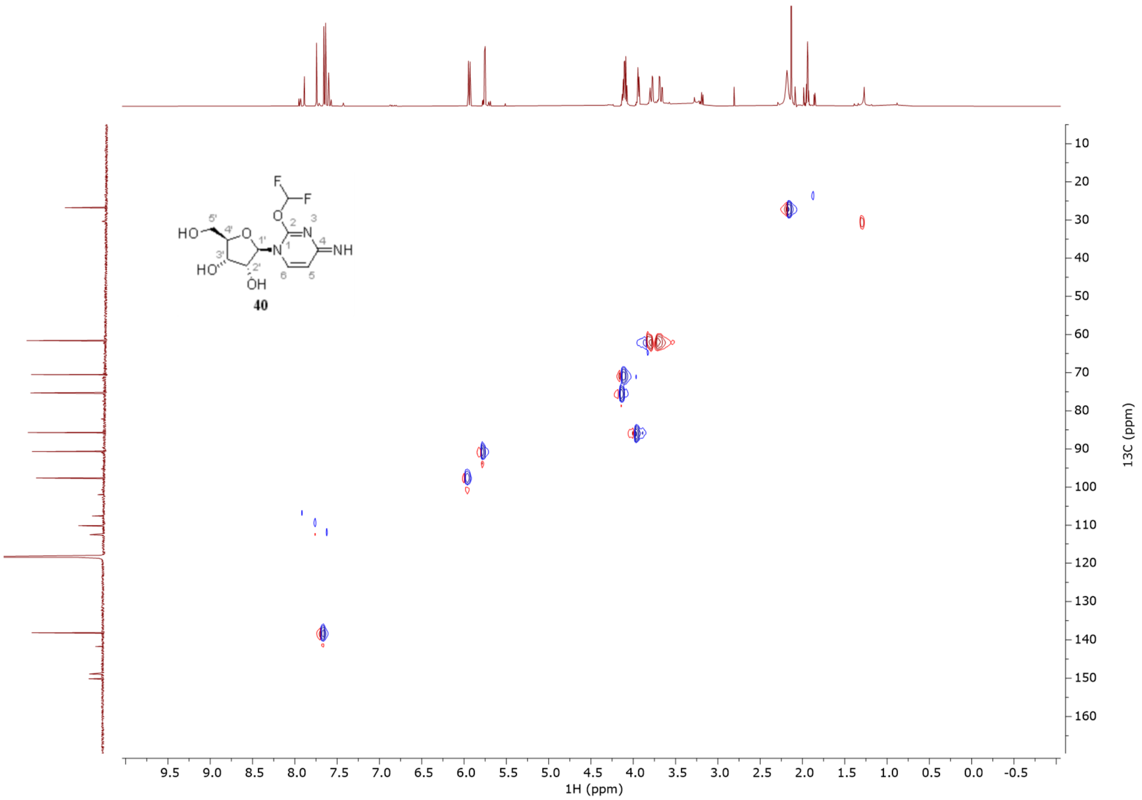

Figure S188 - ^{1}H-^{13}C HSQC NMR spectrum of **40** in CD_3CN

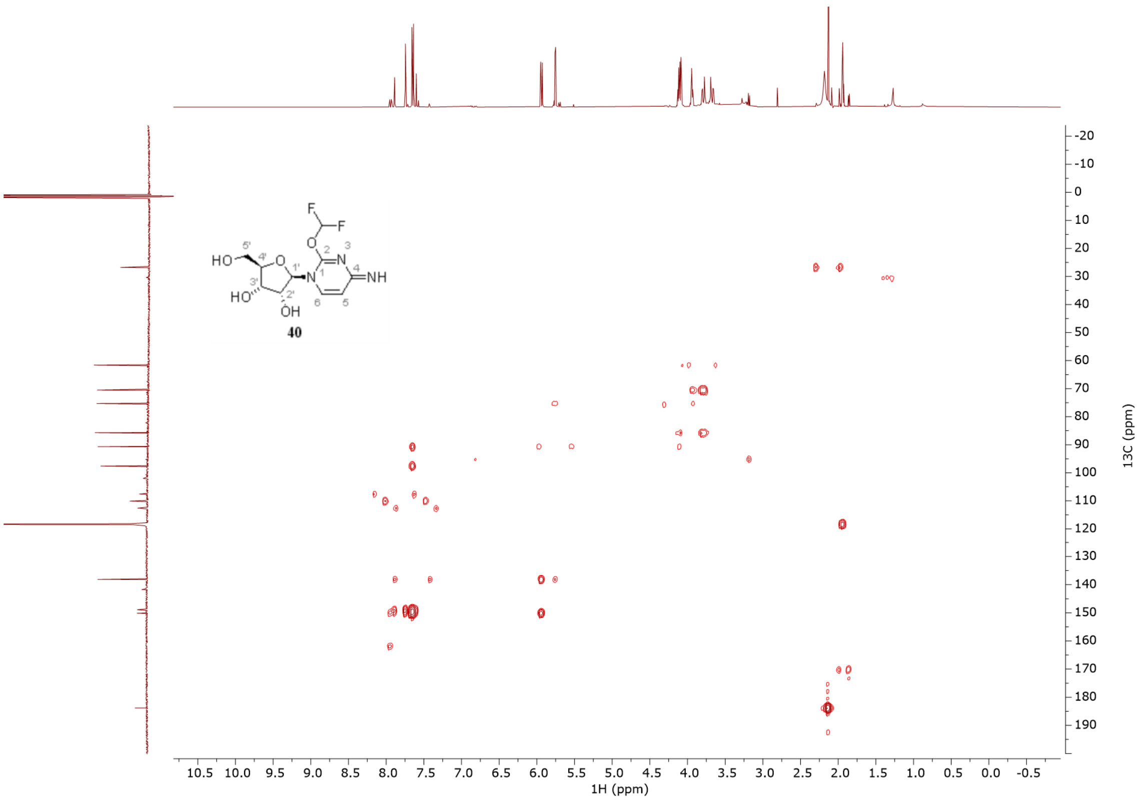

Figure S189 - ^{1}H-^{13}C HMBC NMR spectrum of **40** in CD_3CN

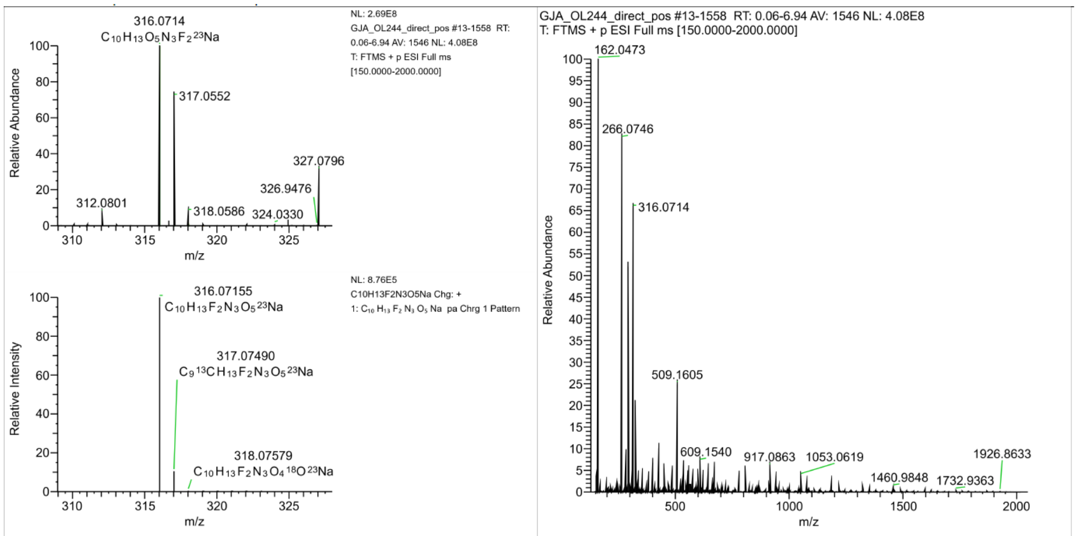

Peak Mass	Display For...	S Fit	RDB	Delta [ppm]	Theo. mass	Rank	Combined S...	# Matched I...	# Missed Iso.	MS Cov. [%]	Pattern Cov....	MSMS Matc...
316.0714	$C_8H_{11}O_4N_6F_2$ ^{23}Na	48.04792123 15247	5.00	3.80	316.07021	1	57.14	3	0	57.65	100	(Collection)
316.0714	$C_{10}H_{13}O_5N_3F_2$ ^{23}Na	39.74386756 26642	4.50	-0.45	316.07155	2	56.7	4	0	57.65	99.89	(Collection)
316.0714	$C_9H_7N_{10}F_2$ ^{23}Na	21.27490160 65634	10.00	-0.43	316.07154	3	55.19	2	3	57.07	95.95	(Collection)
316.0714	$C_{11}H_9ON_7F_2$ ^{23}Na	9.847633350 21953	9.50	-4.68	316.07289	4	49.3	3	0	51.49	88.67	(Collection)
316.0714	$C_{12}H_{15}O_6F_2$ ^{23}Na	7.246176283 16728	4.00	-4.70	316.07289	5	49.16	4	0	51.49	86.83	(Collection)

Figure S190 - HRMS spectrum of **40**

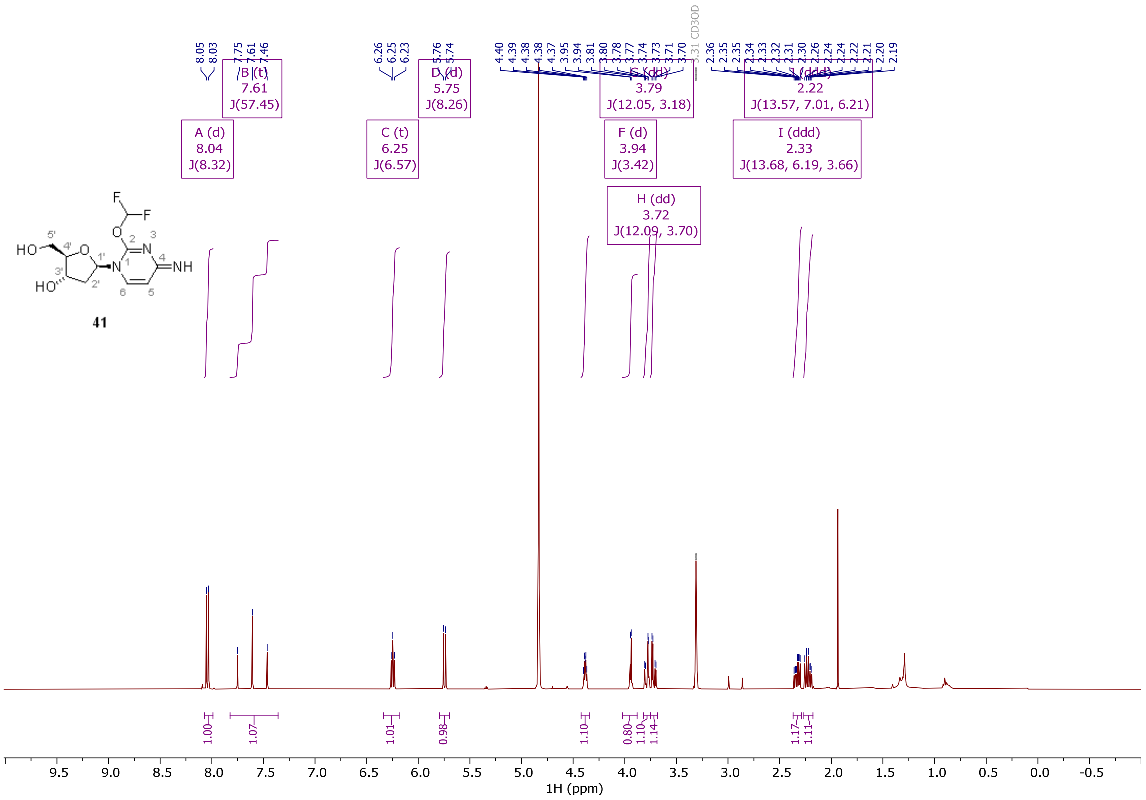

Figure S191 - ^{1}H NMR (400 MHz) spectrum of **41** in CD$_3$OD

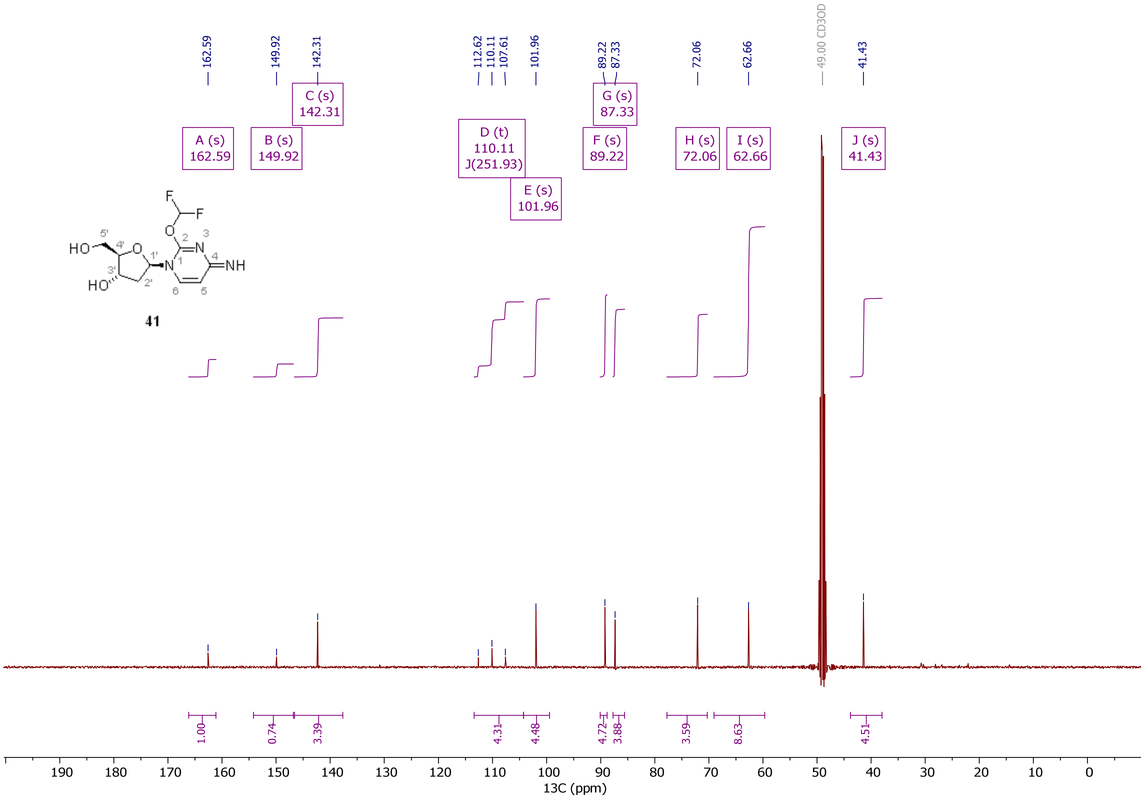

Figure S192 - ^{13}C NMR (101 MHz) spectrum of **41** in CD$_3$OD

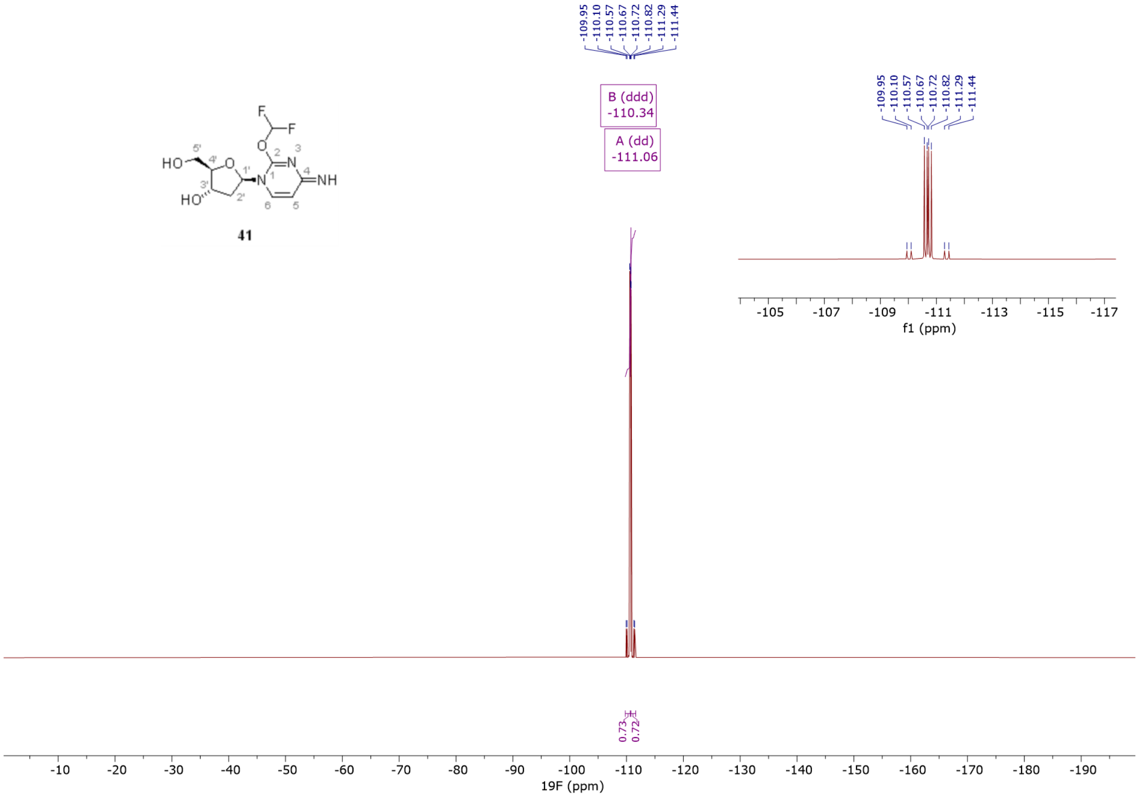

Figure S193 - ^{19}F NMR (376 MHz) spectrum of **41** in CD_3OD

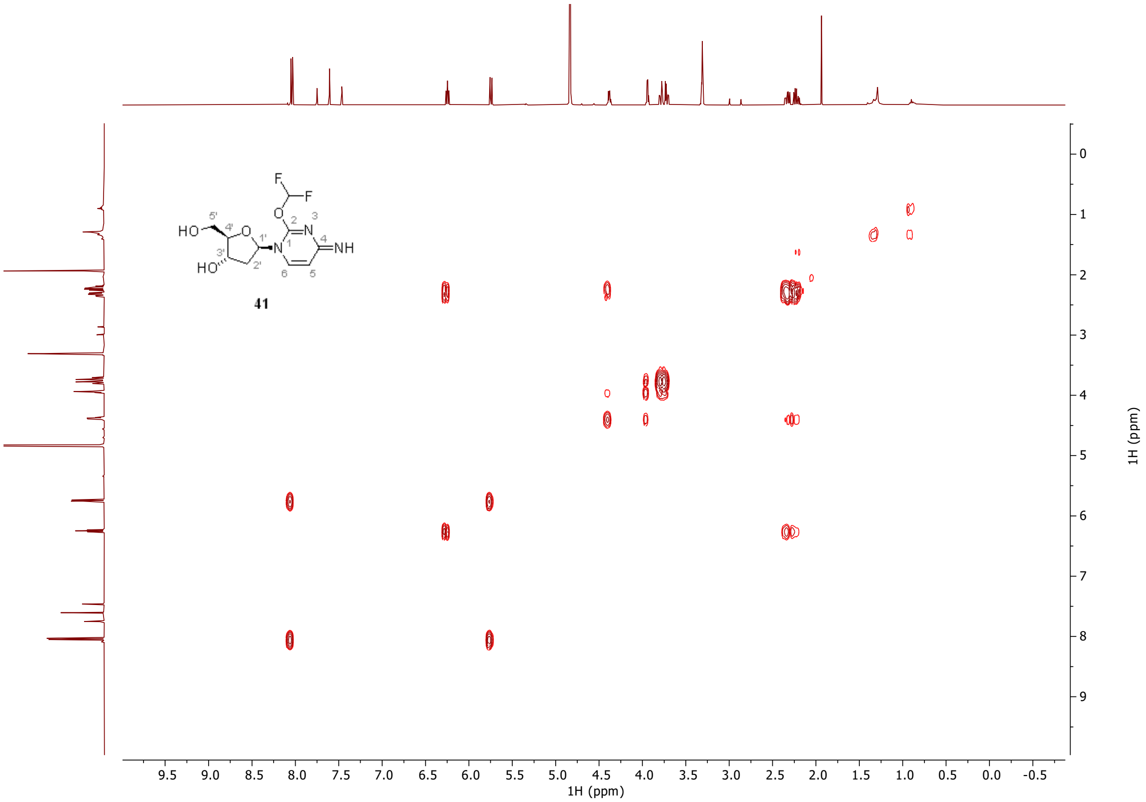

Figure S194 - ^{1}H-^{1}H COSY NMR spectrum of **41** in CD_3OD

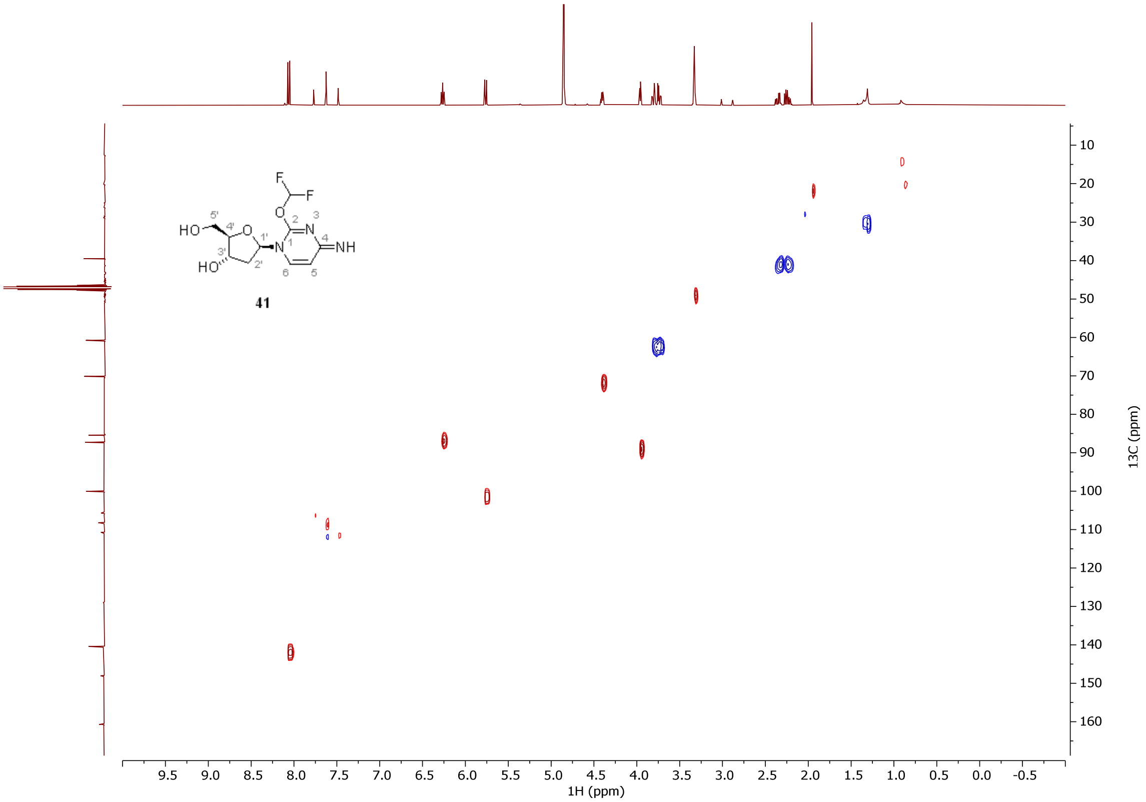

Figure S195 - ^{1}H-^{13}C HSQC NMR spectrum of **41** in CD_3OD

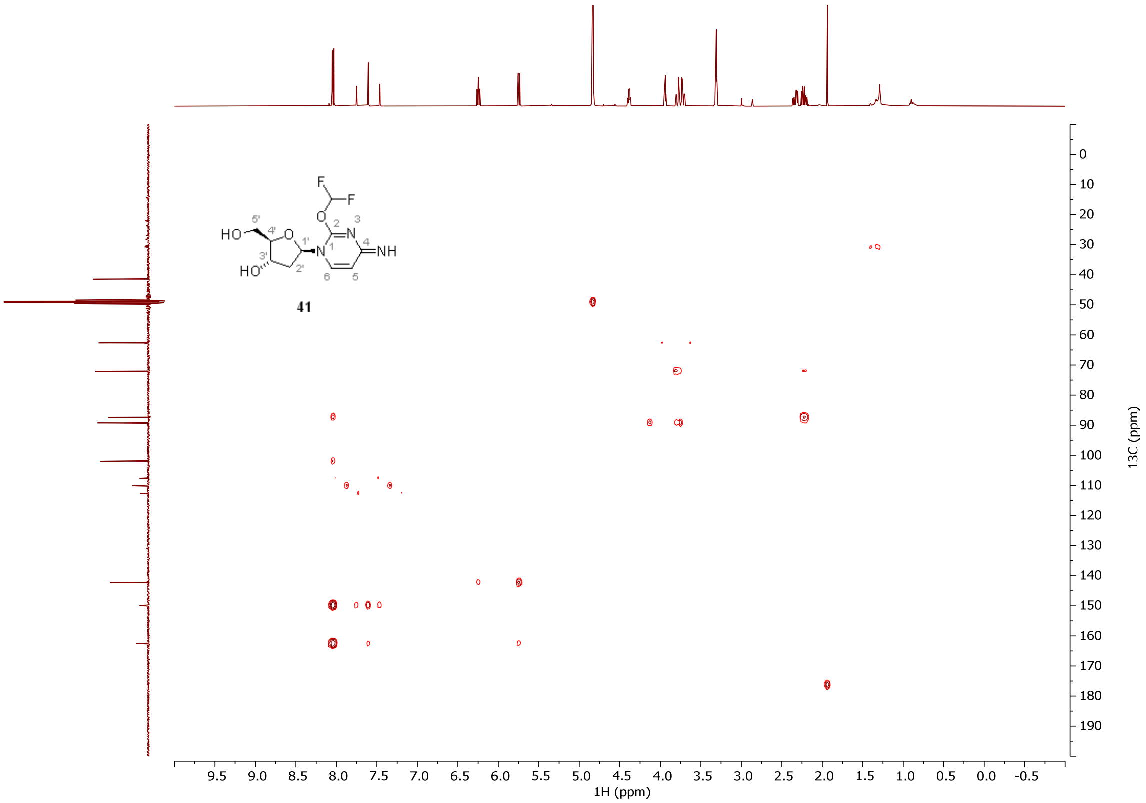

Figure S196 - ^{1}H-^{13}C HMBC NMR spectrum of **41** in CD_3OD

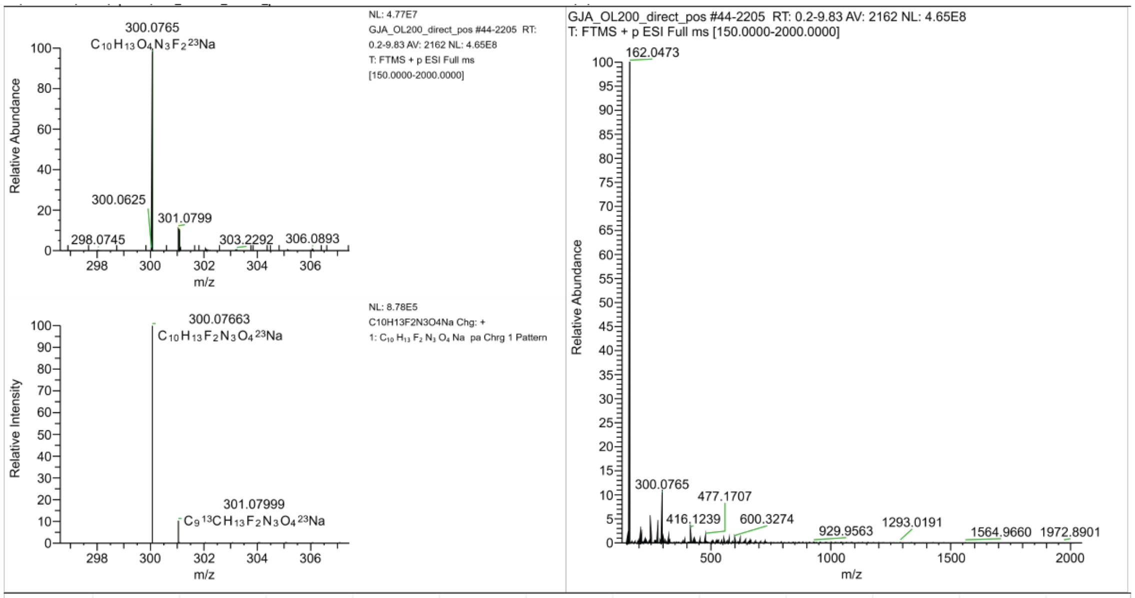

Peak Mass	Display For...	S Fit	RDB	Delta [ppm]	Theo. mass	Rank	Combined S...	# Matched I...	# Missed Iso.	MS Cov. [%]	Pattern Cov....	MSMS Matc...
300.0765	$C_{10}H_{13}O_4N_3F_2{}^{23}Na$	71.4672116594765	4.50	-0.48	300.07663	1	94.25	3	0	95.52	100	(Collection)
300.0765	$C_8H_{11}O_3N_6F_2{}^{23}Na$	12.0837969335841	5.00	3.99	300.07529	2	81.58	3	0	85.44	91.19	(Collection)
300.0765	$C_{11}H_9N_7F_2{}^{23}Na$	6.62031376714166	9.50	-4.94	300.07797	3	81.29	3	1	85.44	88.55	(Collection)
300.0765	$C_{12}H_{15}O_5F_2{}^{23}Na$	6.57459271039736	4.00	-4.96	300.07798	4	81.29	4	0	85.44	87.03	(Collection)

Figure S197 - HRMS spectrum of **41**

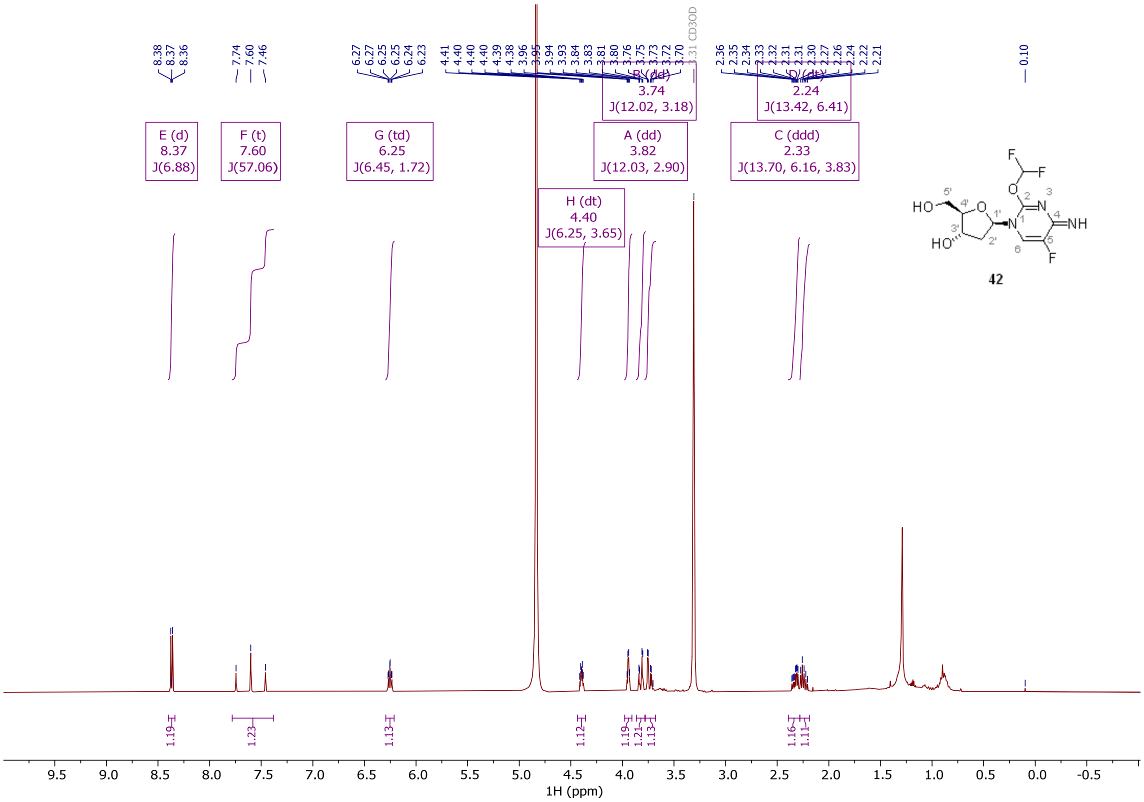

42

Figure S198 - ^{1}H NMR (400 MHz) spectrum of **42** in CD_3OD

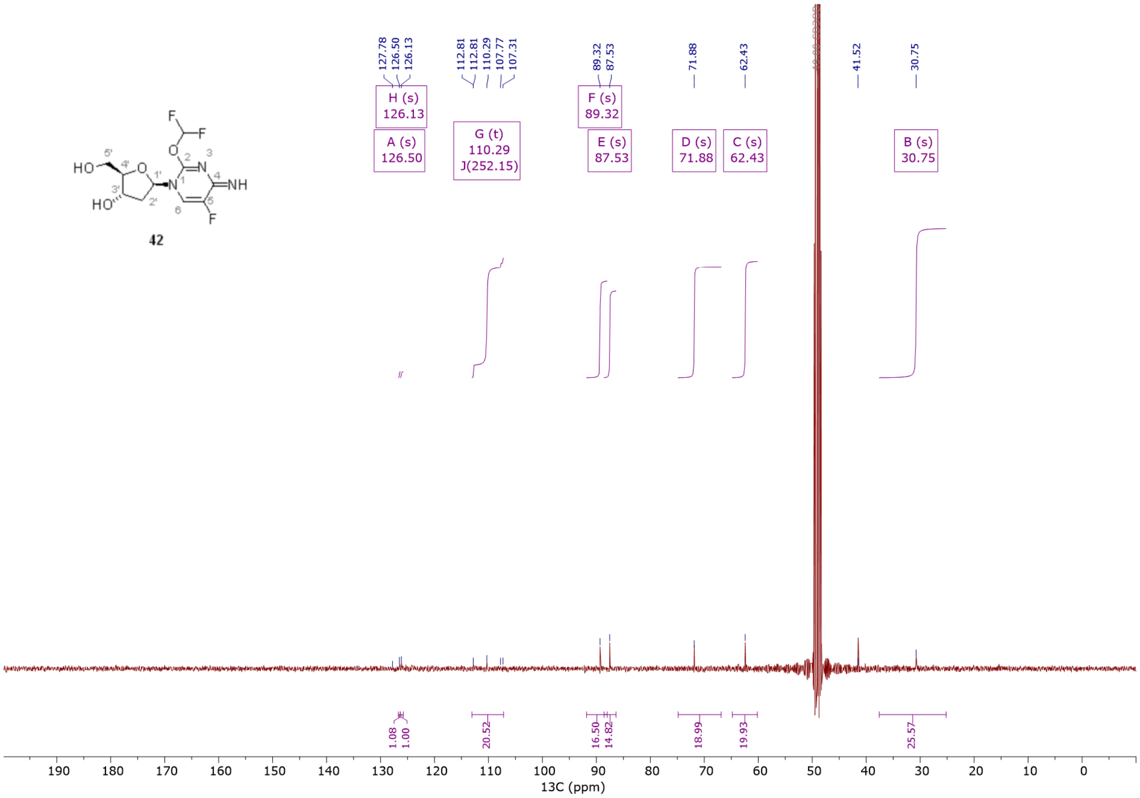

Figure S199 - ^{13}C NMR (101 MHz) spectrum of **42** in CD_3OD

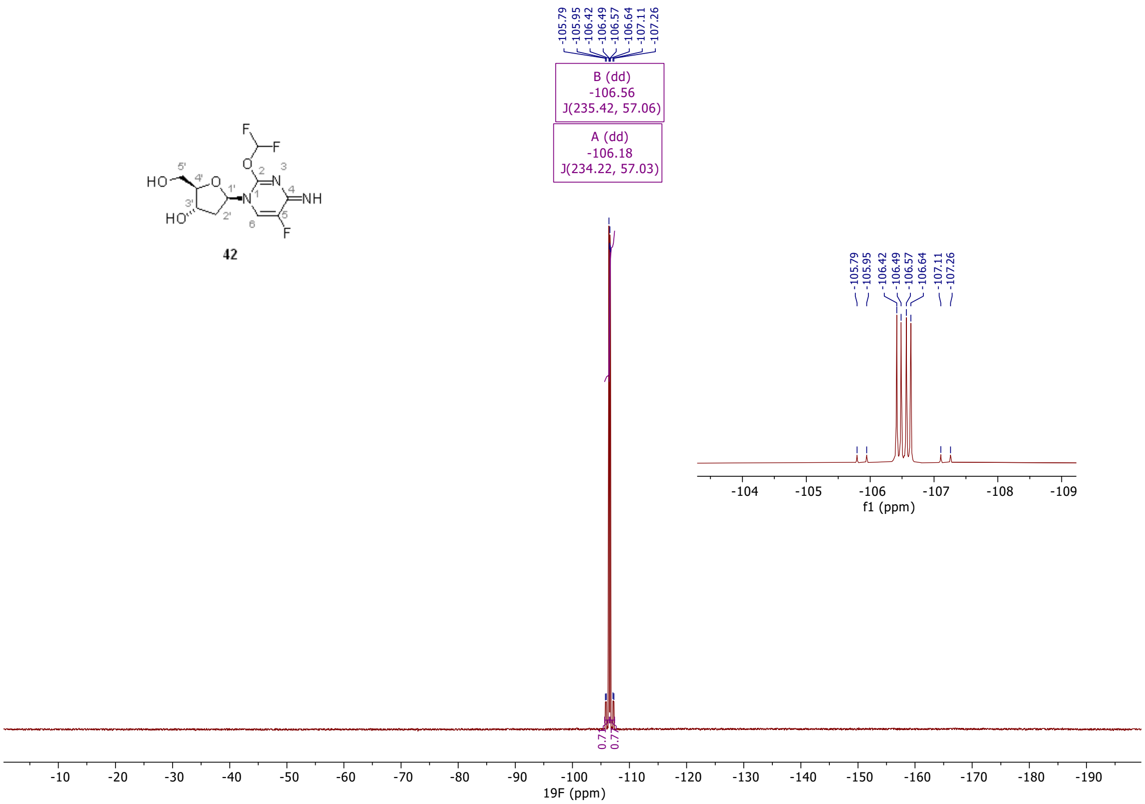

Figure S200 - ^{19}F NMR (376 MHz) spectrum of **42** in CD_3OD

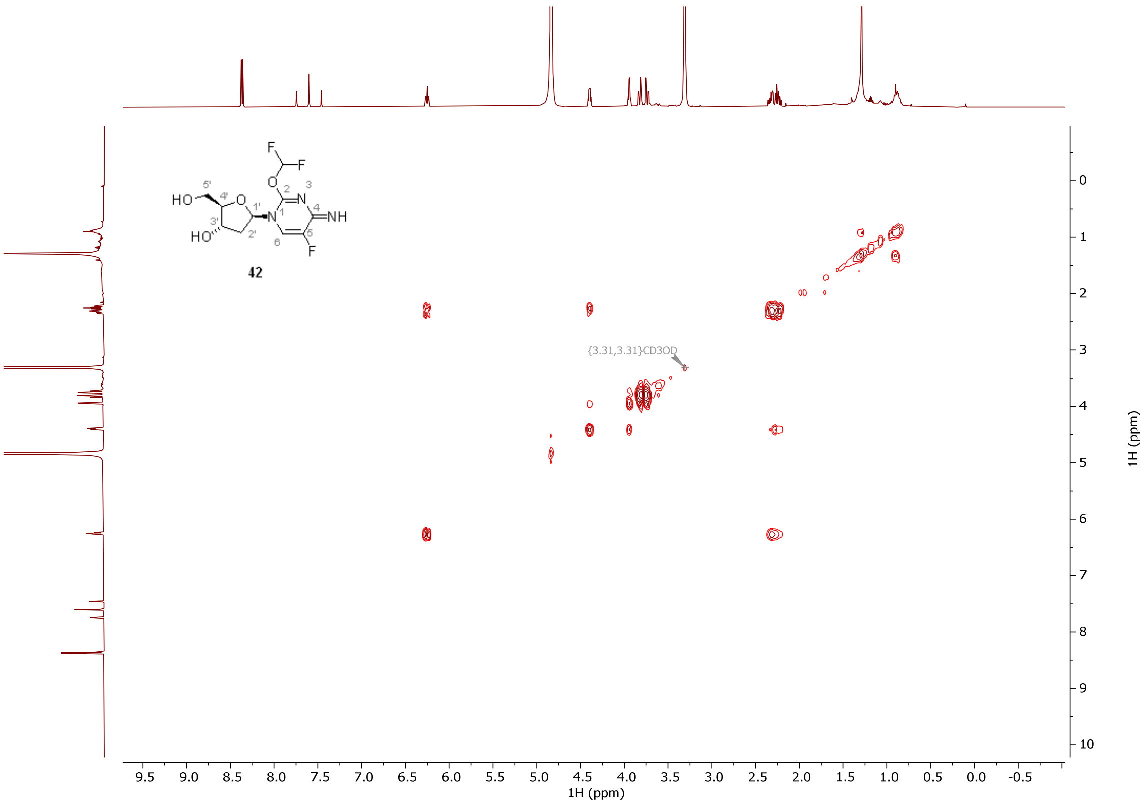

Figure S201 - ^{1}H-^{1}H COSY NMR spectrum of **42** in CD_3OD

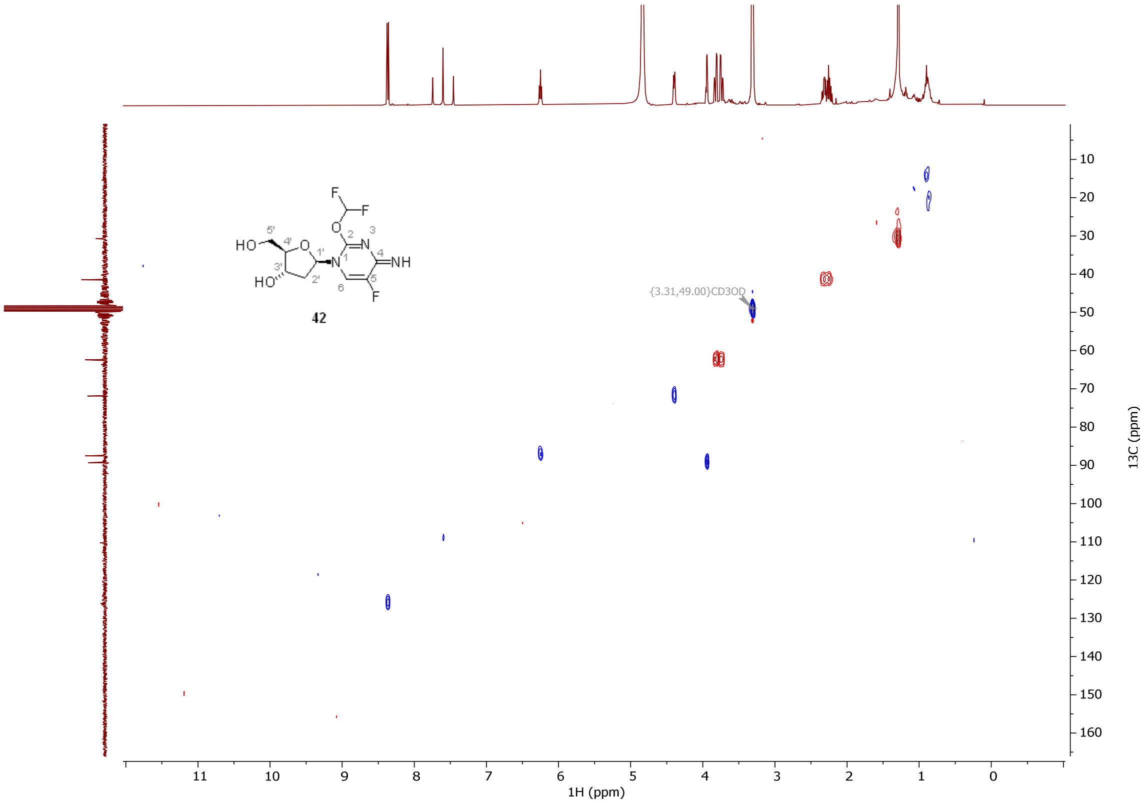

Figure S202 - ^{1}H-^{13}C HSQC NMR spectrum of **42** in CD_3OD

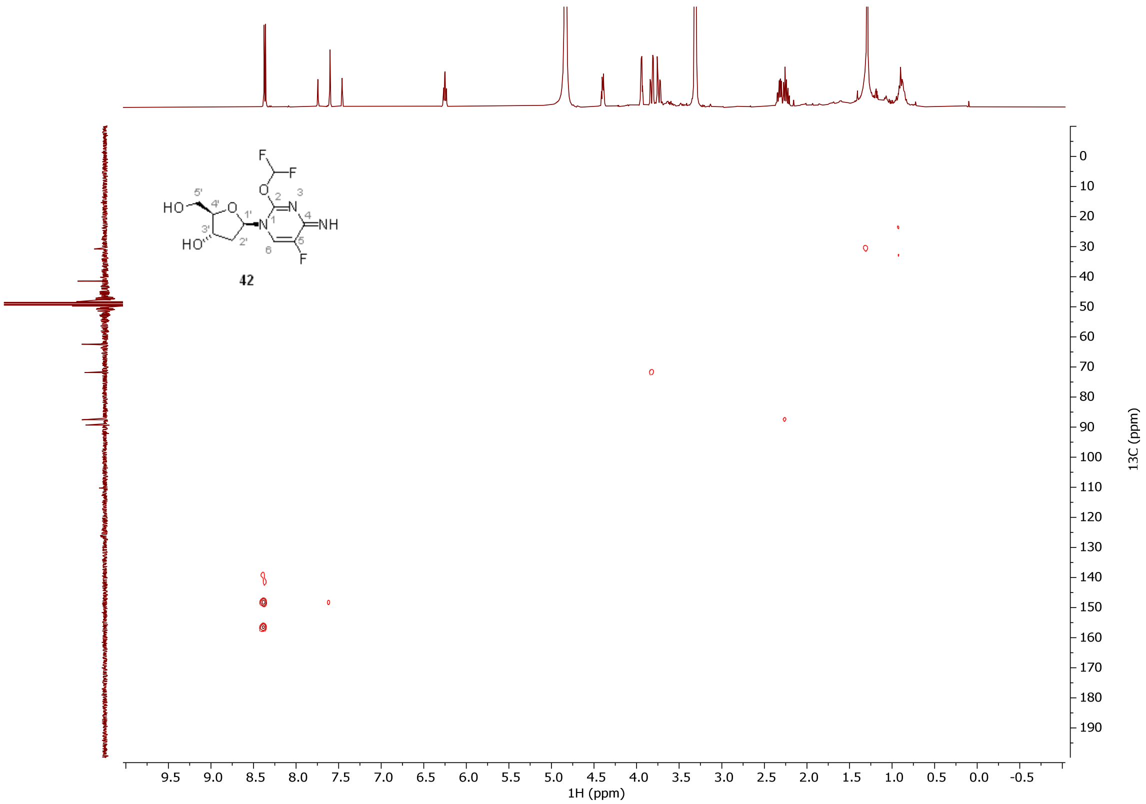

Figure S203 - ^{1}H-^{13}C HMBC NMR spectrum of **42** in CD_3OD

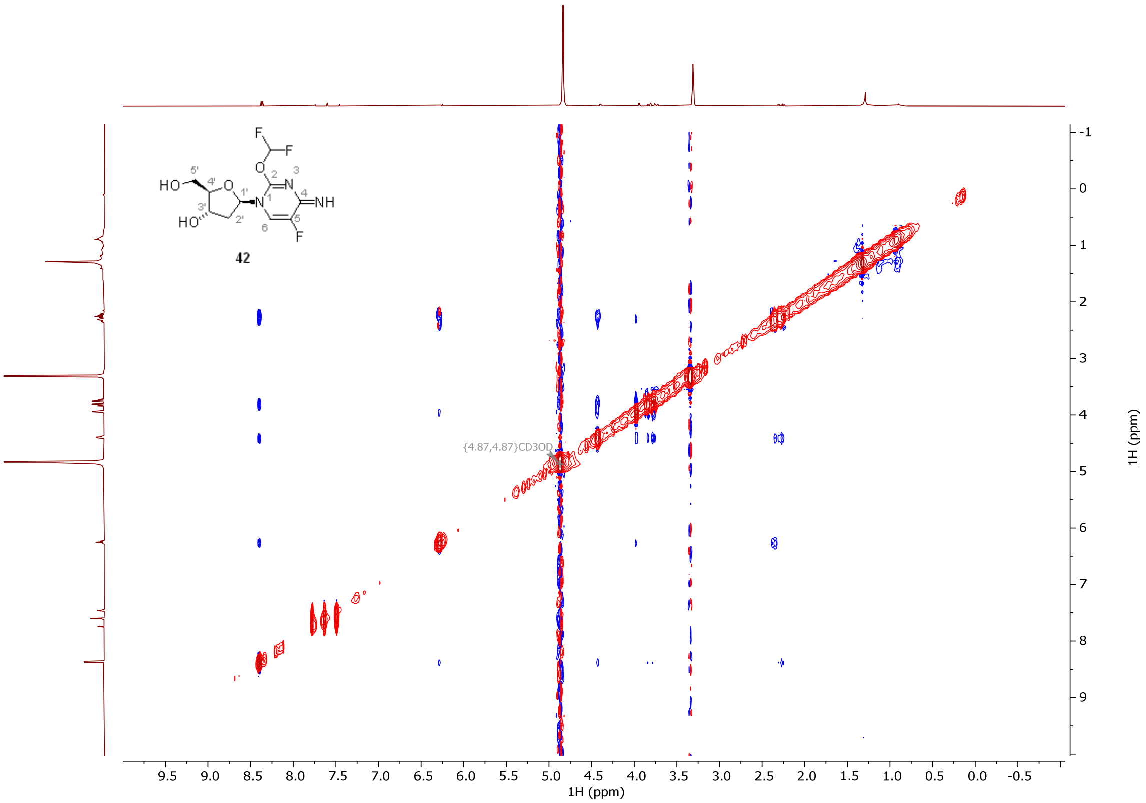

Figure S204 - ^{1}H-^{1}H NOESY NMR spectrum of **42** in CD_3OD

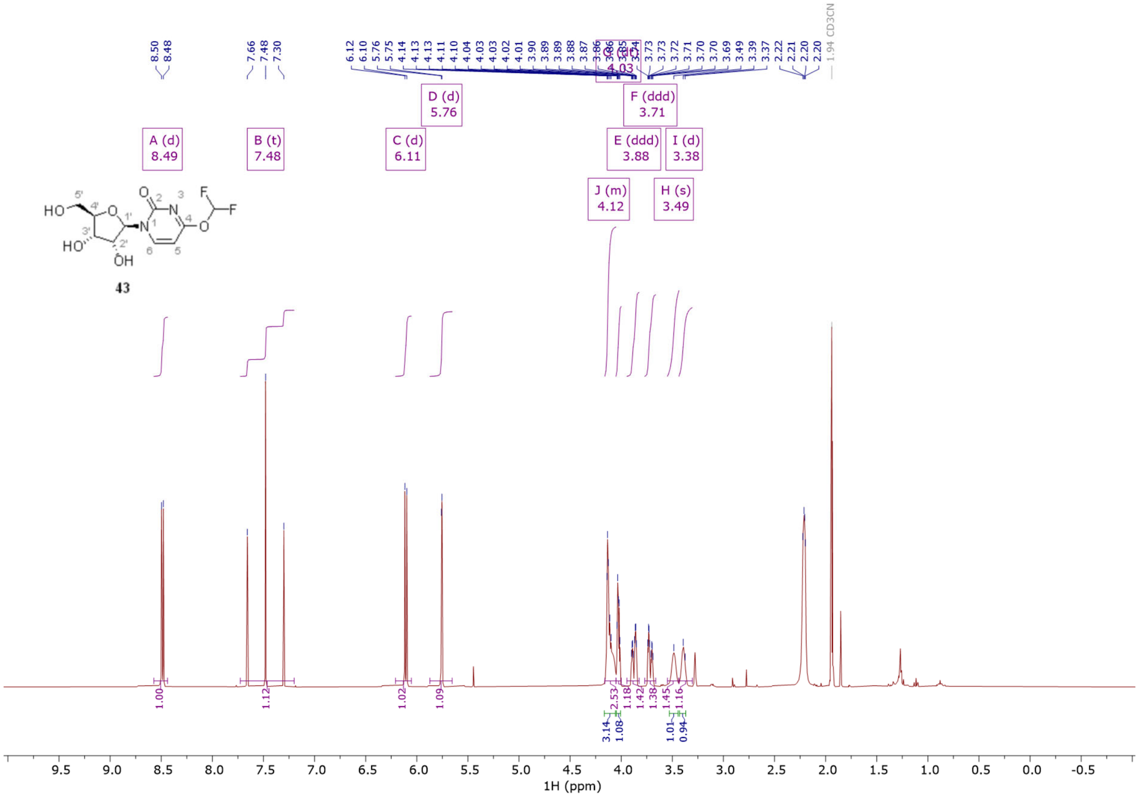

Figure S205 - ^{1}H NMR (400 MHz) spectrum of **43** in CD_3CN

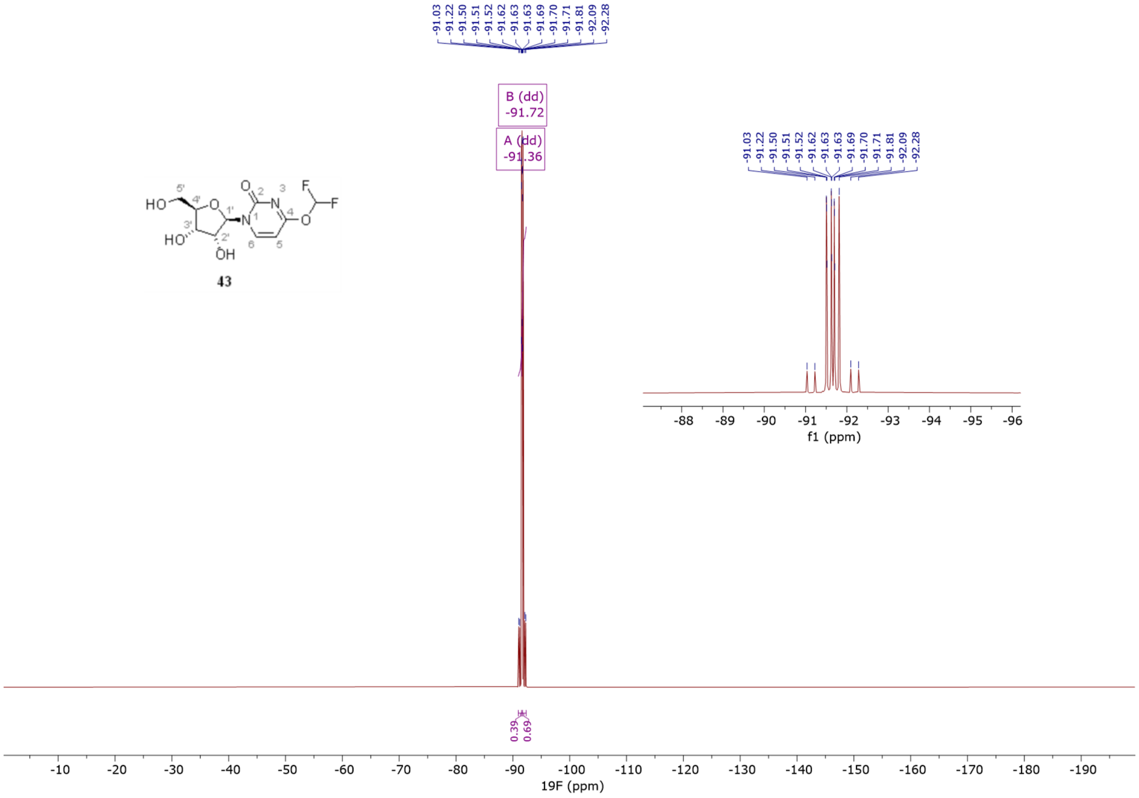

Figure S206 - ^{19}F NMR (376 MHz) spectrum of **43** in CD_3CN

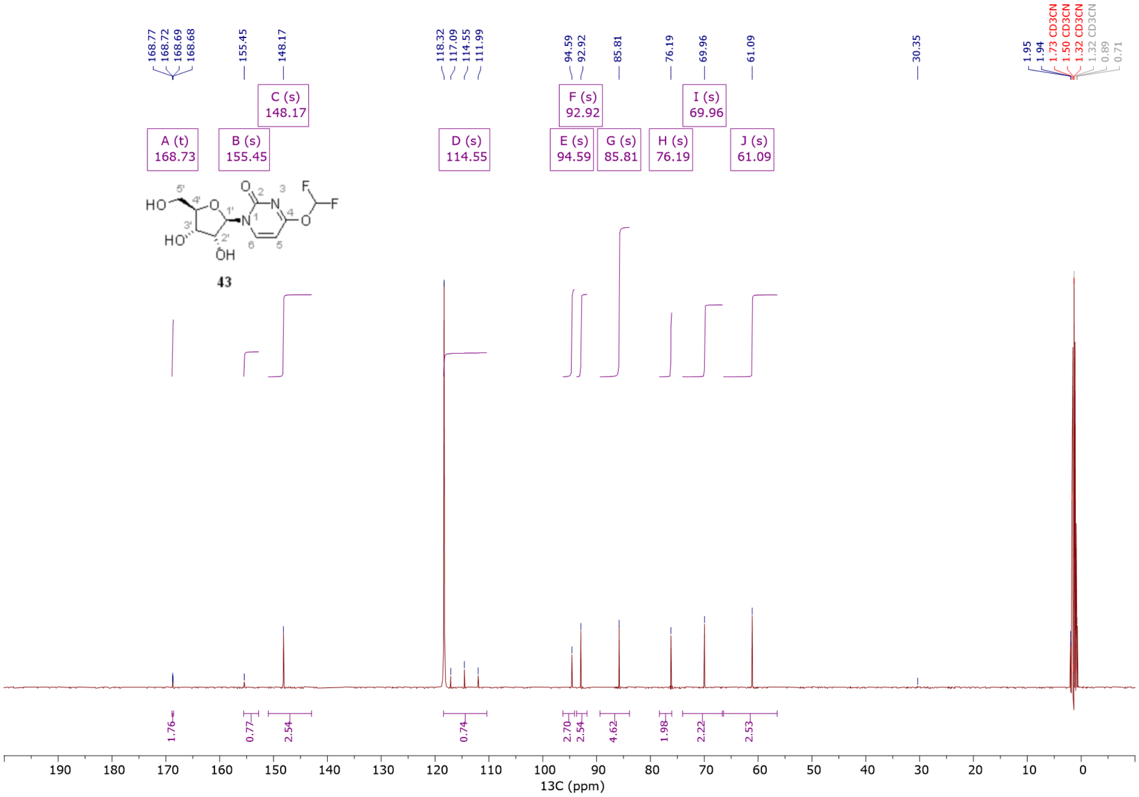

Figure S207 - ^{13}C NMR (101 MHz) spectrum of **43** in CD_3CN

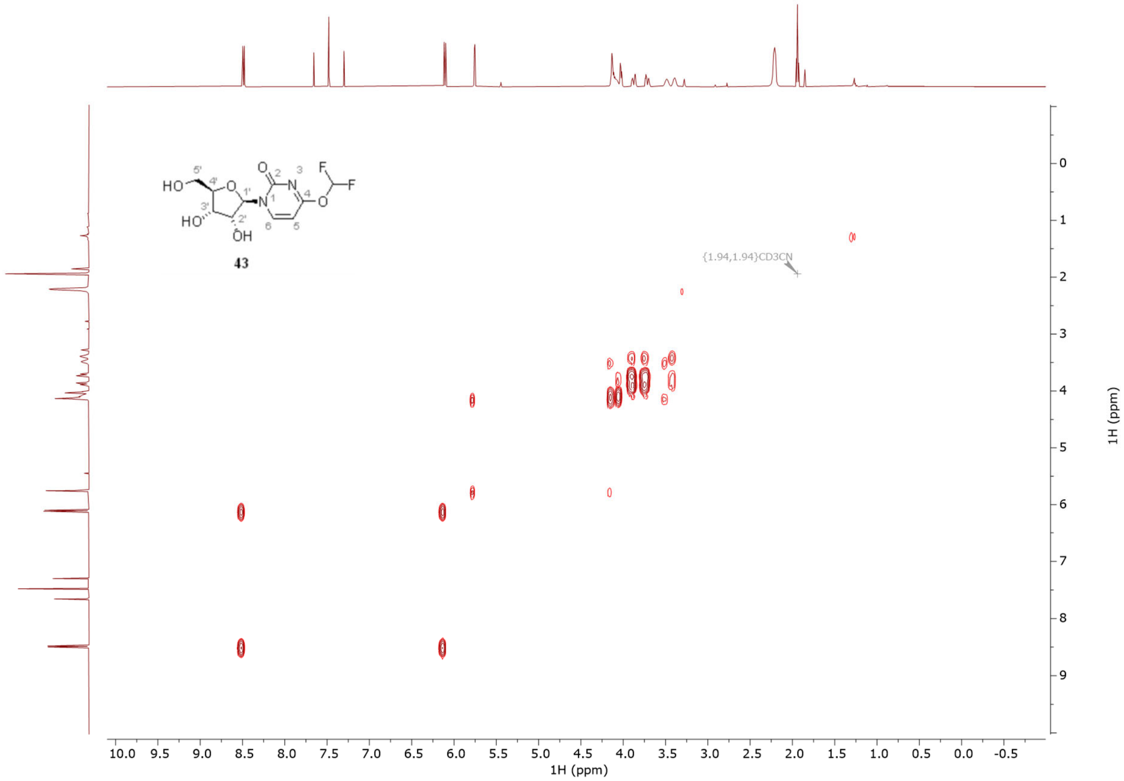

Figure S208 - ^{1}H-^{1}H COSY NMR spectrum of **43** in CD_3CN

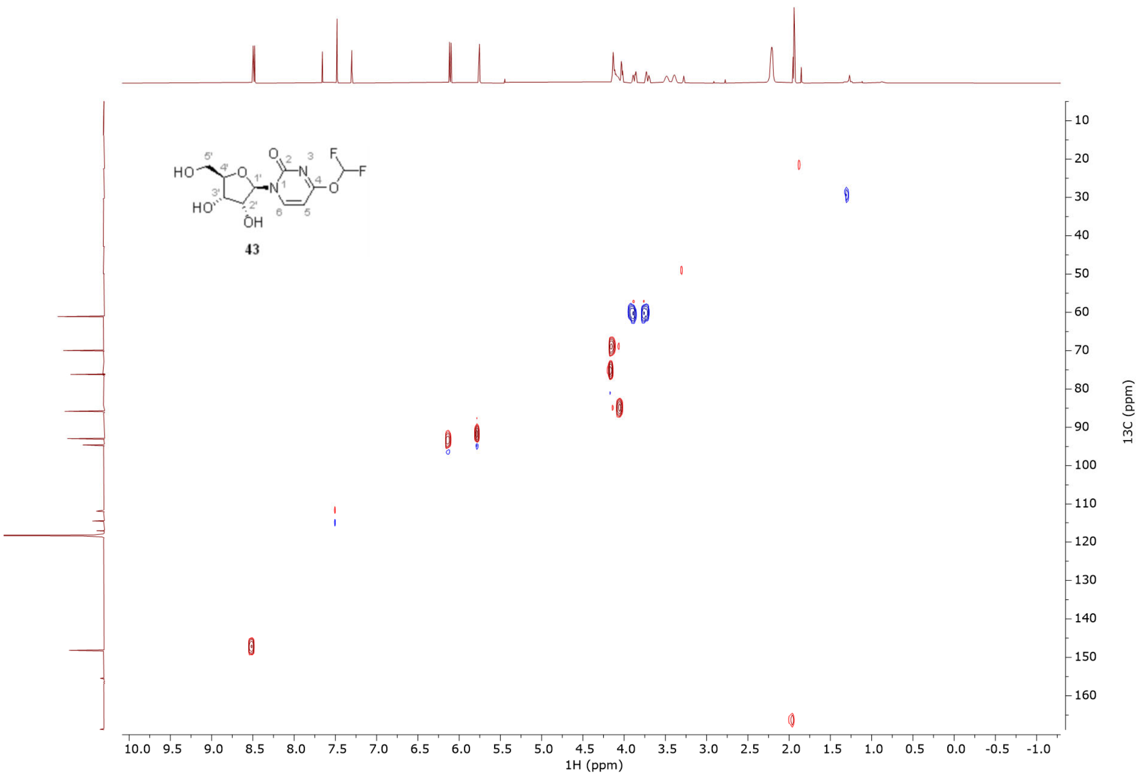

Figure S209 - ^{1}H-^{13}C HSQC NMR spectrum of **43** in CD_3CN

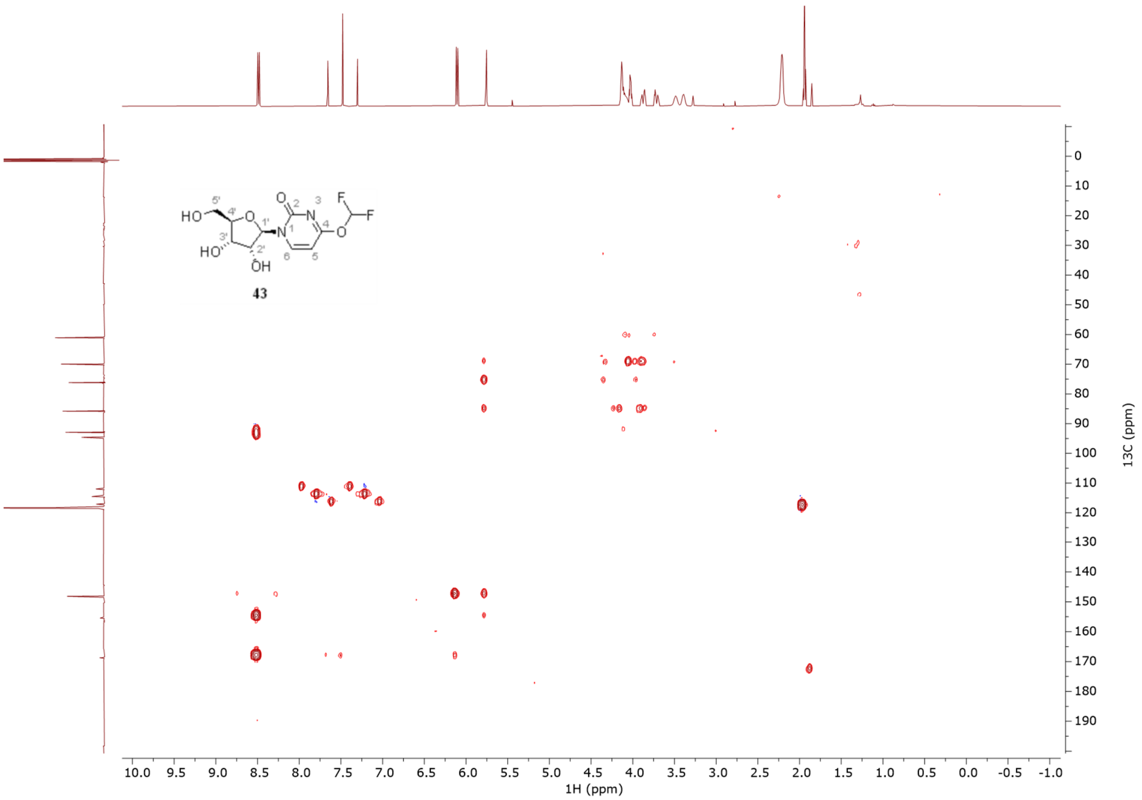

Figure S210 - ^{1}H-^{13}C HMBC NMR spectrum of **43** in CD_3CN

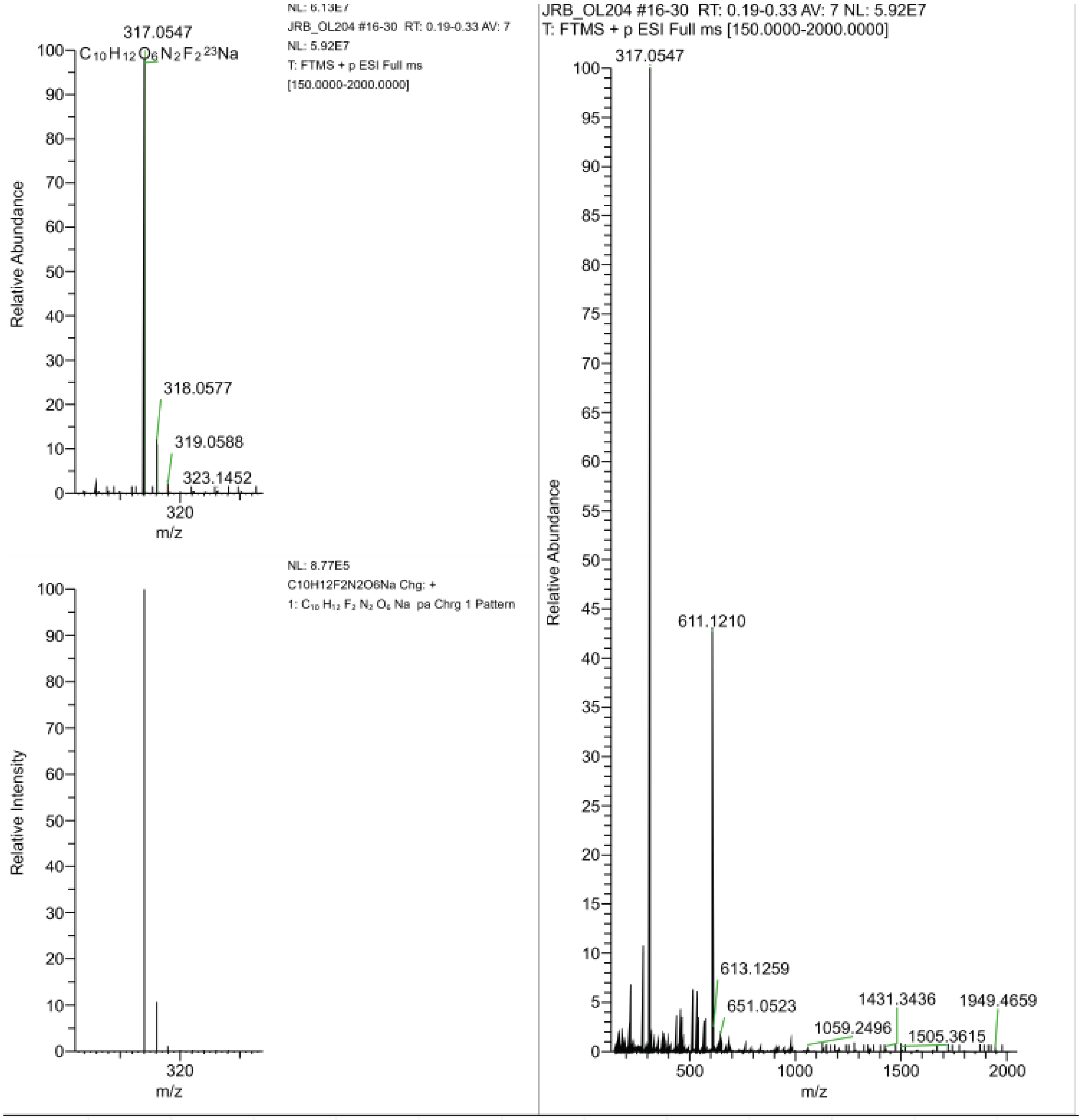

Peak Mass	Display...	S Fit	RDB	Delta [p...	Theo. m...	Rank	Combin...	# Match...	# Misse...	MS Cov....	Pattern...	MSMS...
317.0547	$C_8H_{10}O_5N_5F_2{}^{23}Na$	54.0911252462868	5.00	1.42	317.05422	1	97.07	3	0	99.46	100	(Collection)
317.0547	$C_9H_6ON_9F_2{}^{23}Na$	28.9765303342268	10.00	-2.80	317.05556	2	94.49	2	1	98.13	99.48	(Collection)
317.0547	$C_{10}H_{12}O_6N_2F_2{}^{23}Na$	21.8947376778744	4.50	-2.81	317.05556	3	94.12	4	0	98.13	98.45	(Collection)

Figure S211 - HRMS spectrum of **43**

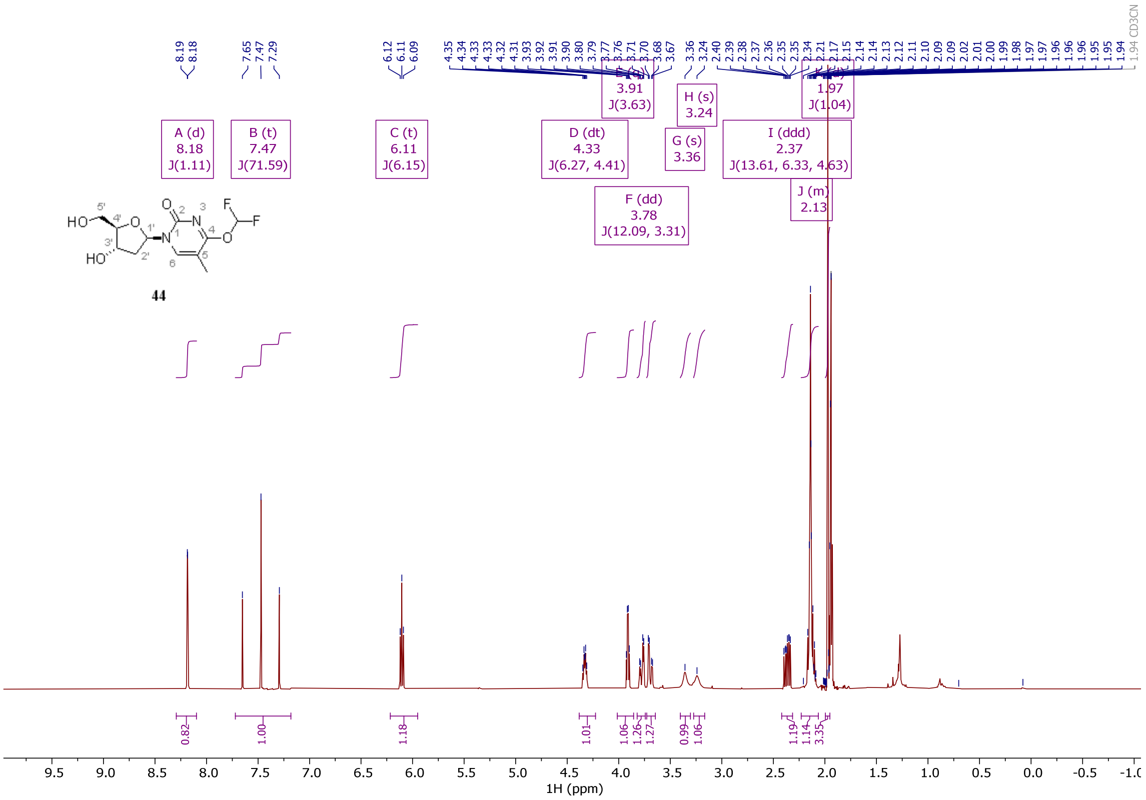

Figure S212 - ^{1}H NMR (400 MHz) spectrum of **44** in CD_3CN

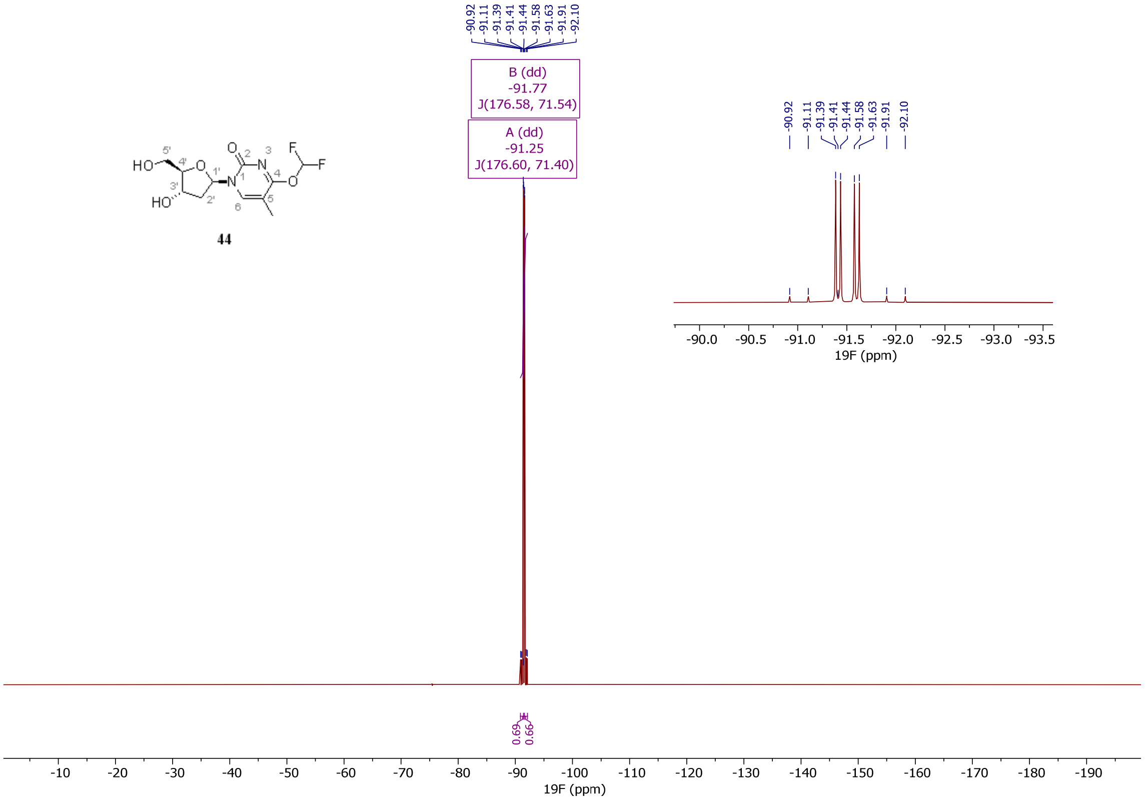

Figure S213 - ^{19}F NMR (376 MHz) spectrum of **44** in CD_3CN

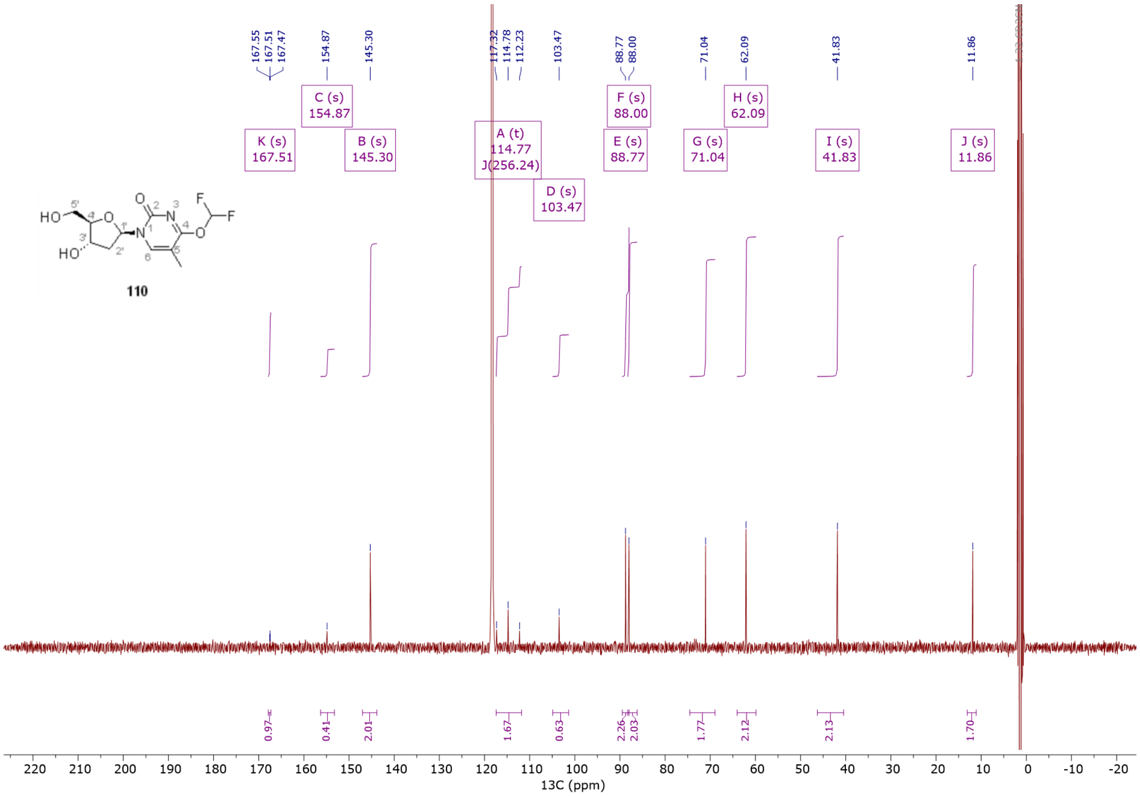

Figure S214 - ^{13}C NMR (101 MHz) spectrum of **44** in CD_3CN

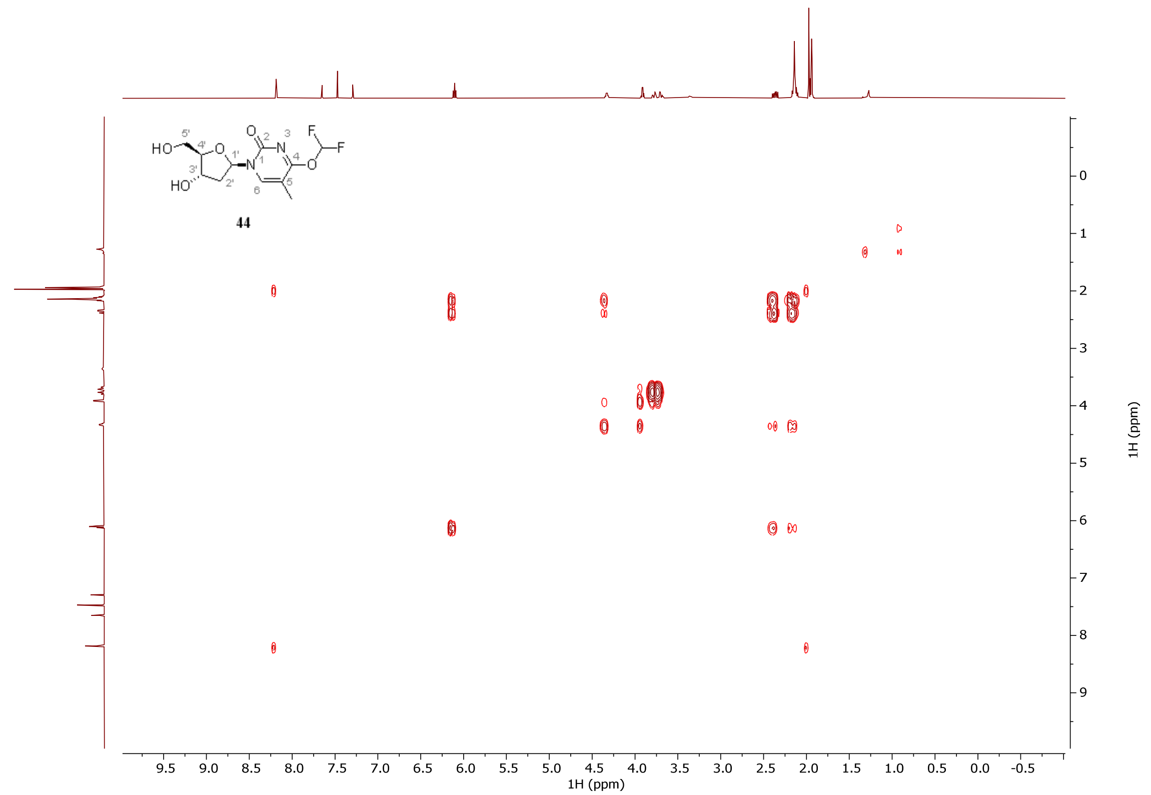

Figure S215 - ^{1}H-^{1}H COSY NMR spectrum of **44** in CD_3CN

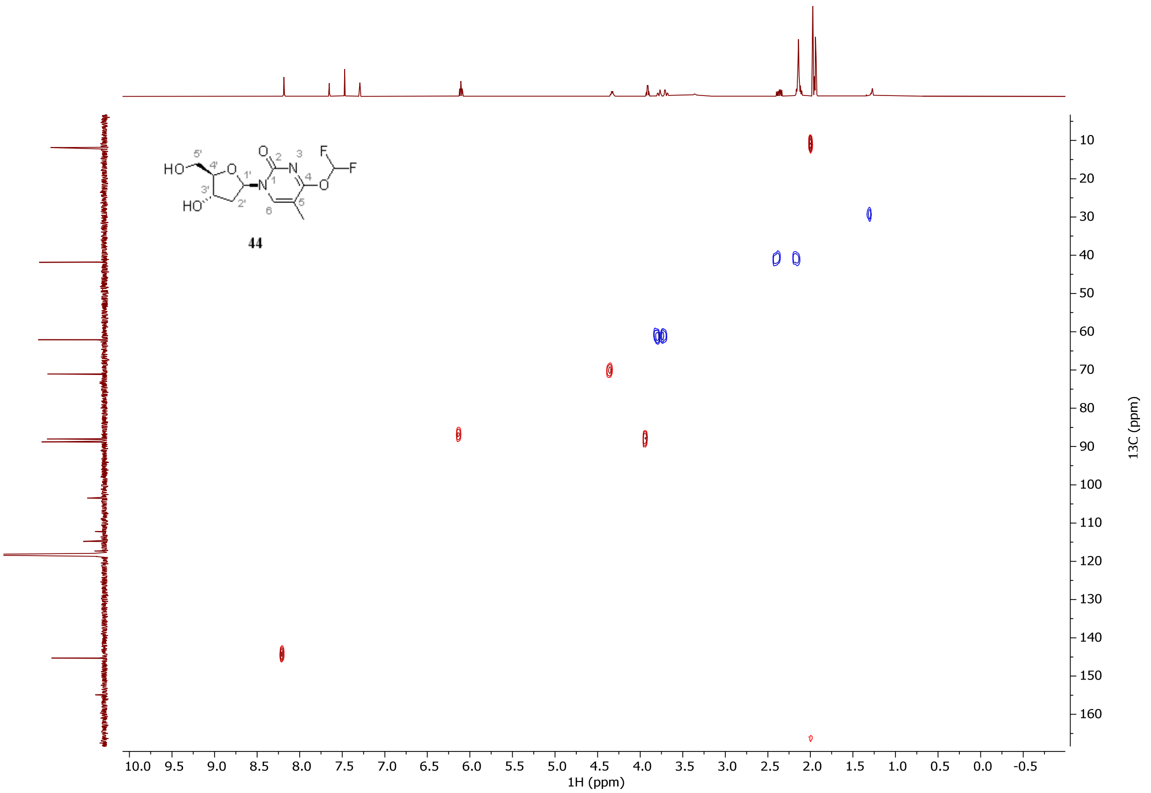

Figure S216 - ^{1}H-^{13}C HSQC NMR spectrum of **44** in CD_3CN

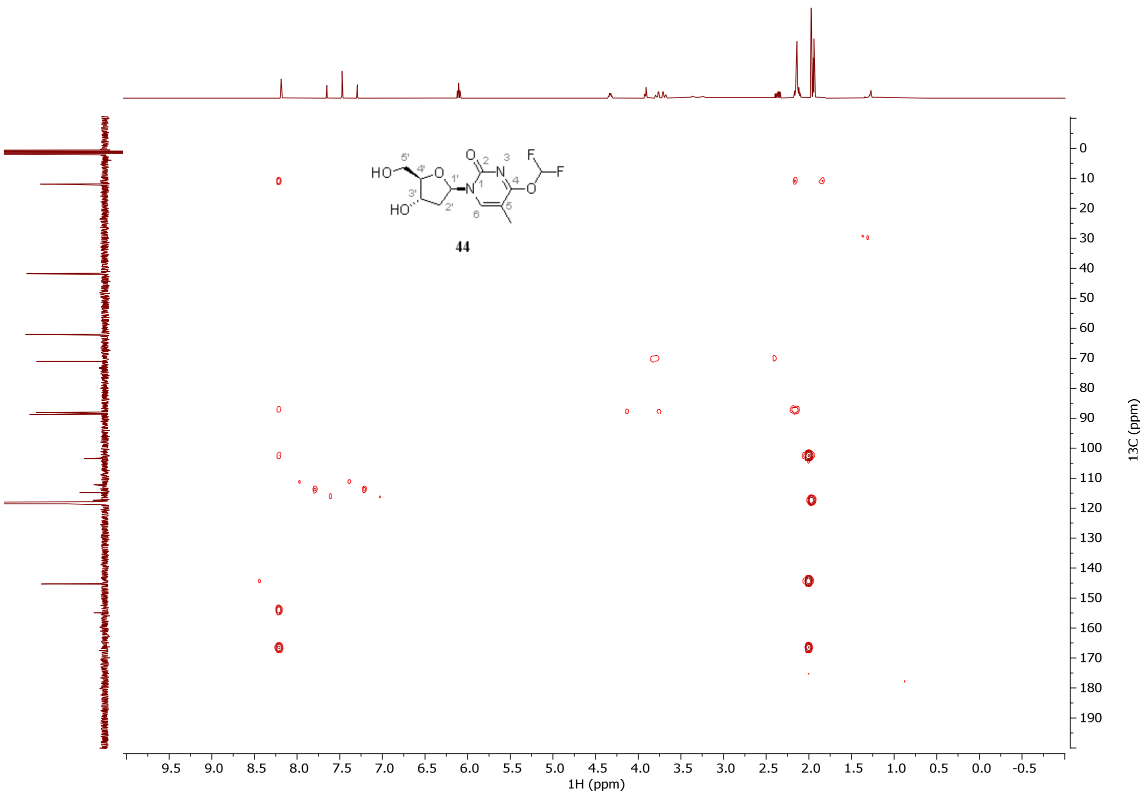

Figure S217 - ¹H-¹³C HMBC NMR spectrum of **44** in CD_3CN

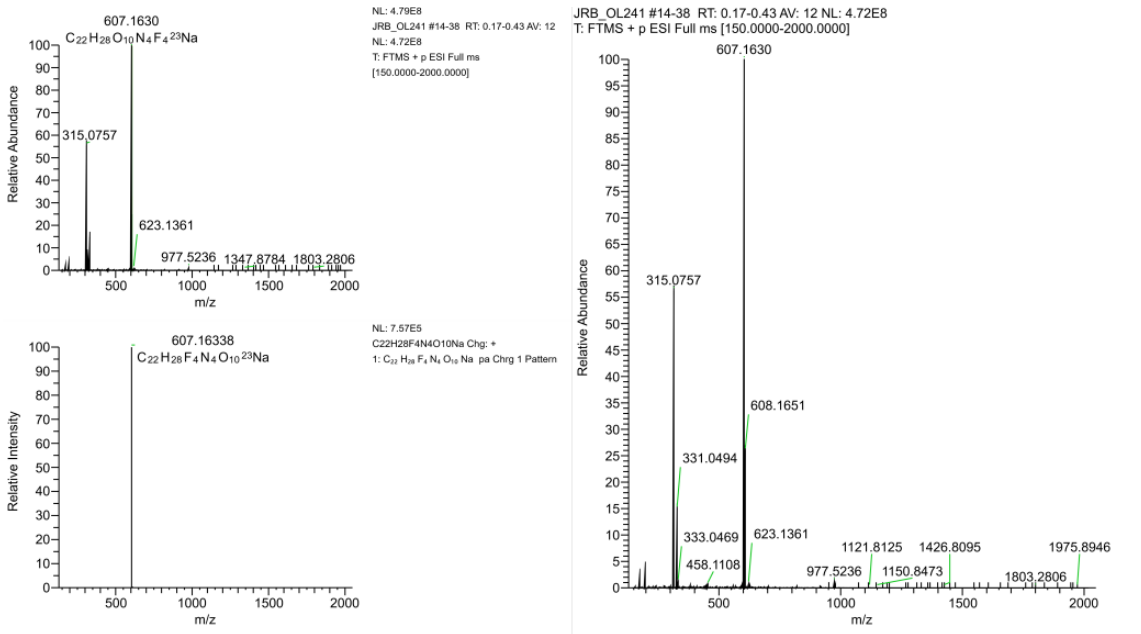

Peak Mass	Display For...	S Fit	RDB	Delta [ppm]	Theo. mass	Rank	Combined S...	# Matched I...	# Missed Iso.	MS Cov. [%]	Pattern Cov....	MSMS Matc.
607.1630	$C_{25}H_{27}O_9N_4F_3{}^{23}Na$	77.4558623250954	12.50	1.23	607.16223	1	98.64	4	0	99.81	100	(Collection)
607.1630	$C_{22}H_{28}O_{10}N_4F_4{}^{23}Na$	72.6068037773758	8.50	-0.65	607.16338	2	98.38	4	0	99.81	100	(Collection)
607.1630	$C_{24}H_{30}O_{11}NF_4{}^{23}Na$	21.3894650206523	8.00	-2.87	607.16472	3	95.15	5	0	99.25	99.28	(Collection)
607.1630	$C_{19}H_{29}O_{11}N_4F_5{}^{23}Na$	29.7324005453115	4.50	-2.54	607.16452	4	77.42	4	0	80.07	82.63	(Collection)

Figure S218 – HRMS spectrum of **44**
